# Supplementary figures and images for: Freshwater sponge hosts and their green algae symbionts: a tractable model to understand intracellular symbiosis (part 1 of 2)
Source: PeerJ. 2021 Feb 11;9:e10654. doi: 10.7717/peerj.10654 (PMC7882143; doi:10.7717/peerj.10654)

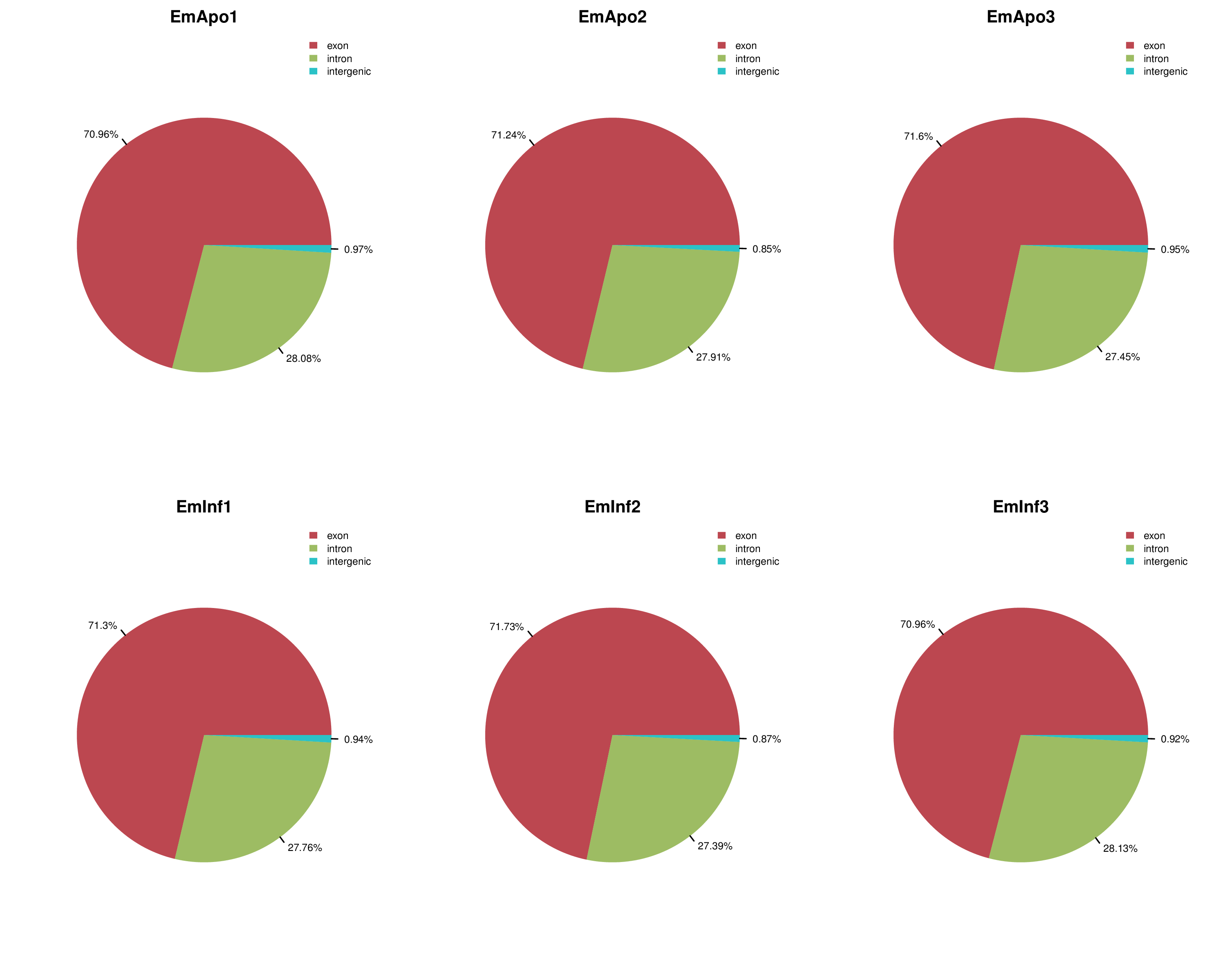

Supplement: Supplemental Information 1 — Red (largest portion in all cases) shows exonic, green intronic, and blue intergenically E. muelleri mapped proportion of reads per sample, with percentages as indicated on figure. [file peerj-09-10654-s001.png]

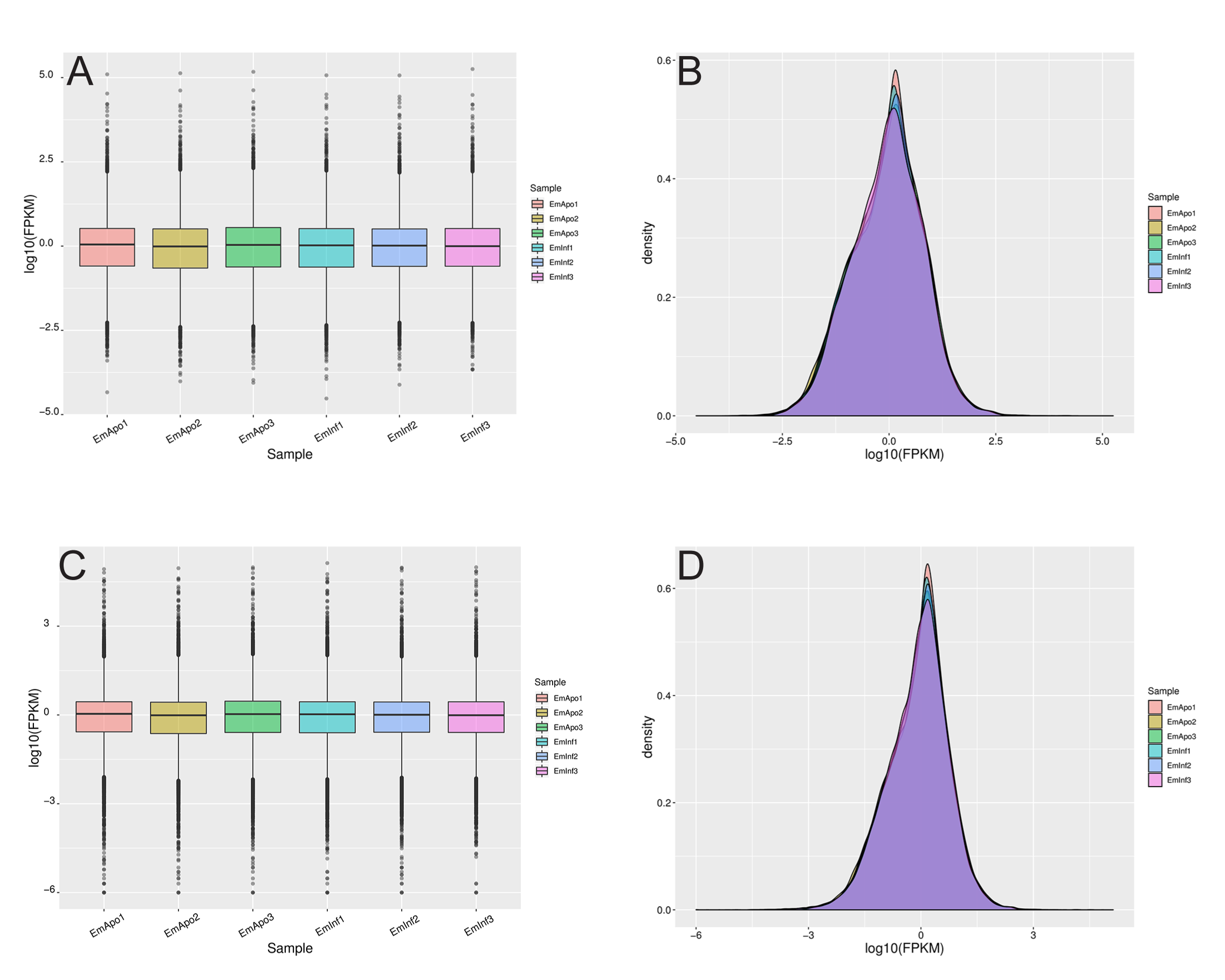

Supplement: Supplemental Information 2 — (A) Boxplot showing distribution of sponge gene expression values (log10(FPKM)) for each sample (aposymbiotic and 24 h post-infection, triplicate samples) in RNASeq dataset. Expression values are similar. (B) Density plot of log10(FPKM) values of protein coding genes. (C) Boxplot showing distribution of transcript expression values. (D) Density plot of log10(FPKM) values of transcripts. [file peerj-09-10654-s002.png]

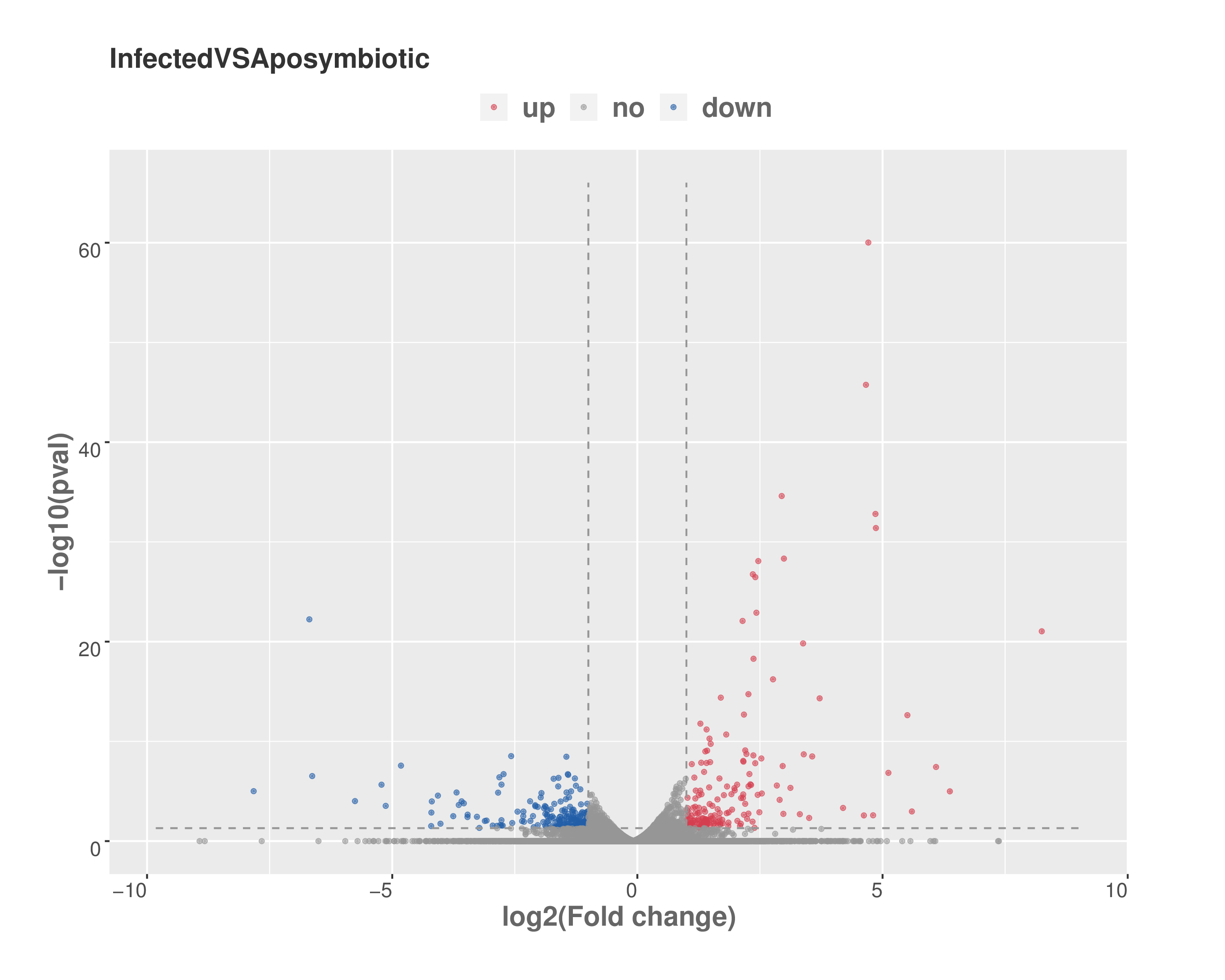

Supplement: Supplemental Information 3 — Scatter plot showing statistical significant versus fold change between aposymbiotic and symbiotic E. muelleri. Horizontal axis shows log 2 fold change value and vertical axis is the mean expression value of log 10 ( q-value). Red dots represent up-regulated genes, blue dots represent down-regulated, and grey dots represent genes that are not statistically significant for regulation. [file peerj-09-10654-s003.png]

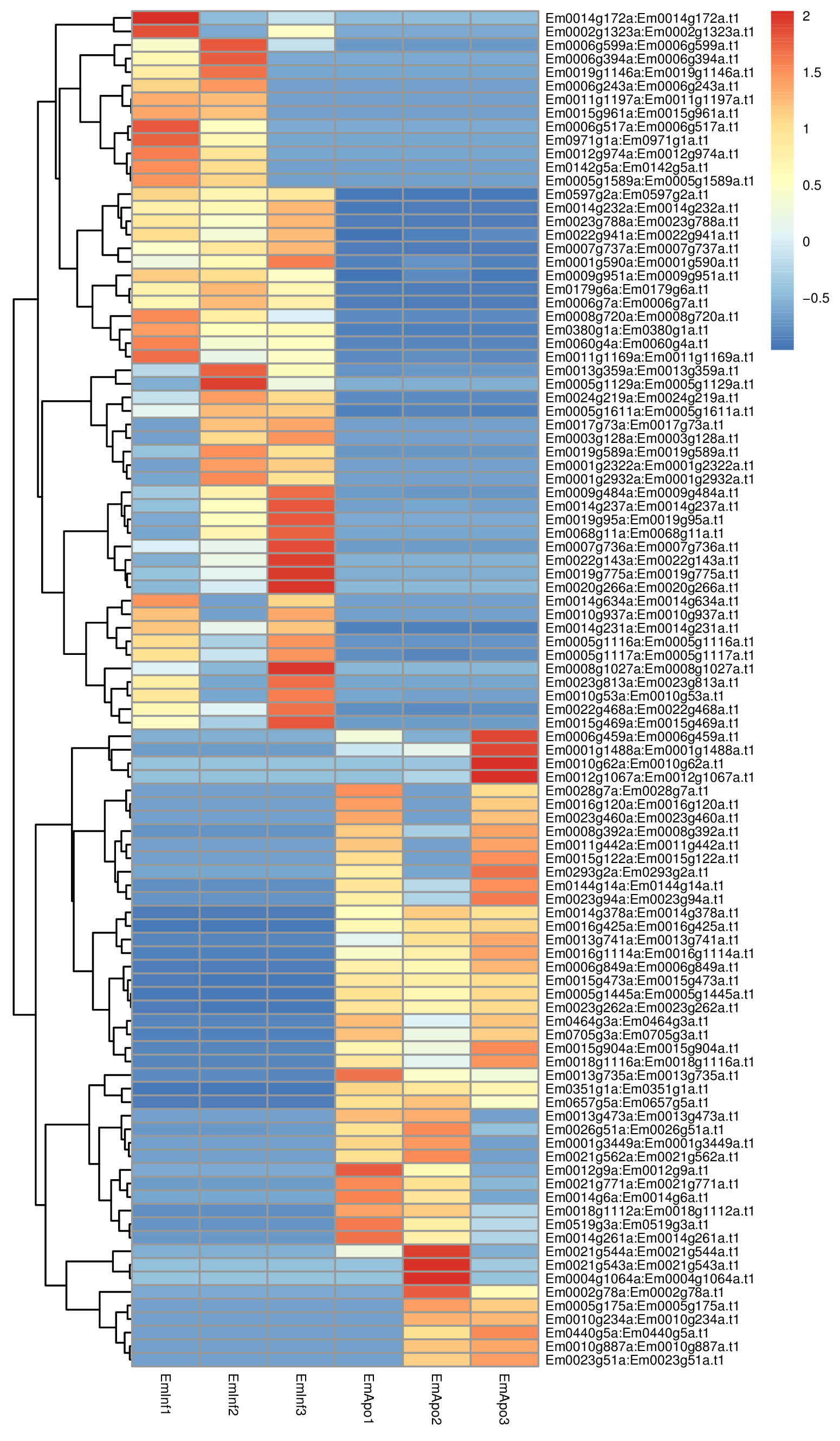

Supplement: Supplemental Information 4 — Relative expression of differentially expressed sponge transcripts (scale at right) are shown comparing triplicate samples for aposymbiotic and 24 h post-infected sponges. Gene IDs are provided at the right of each expression profile. [file peerj-09-10654-s004.png]

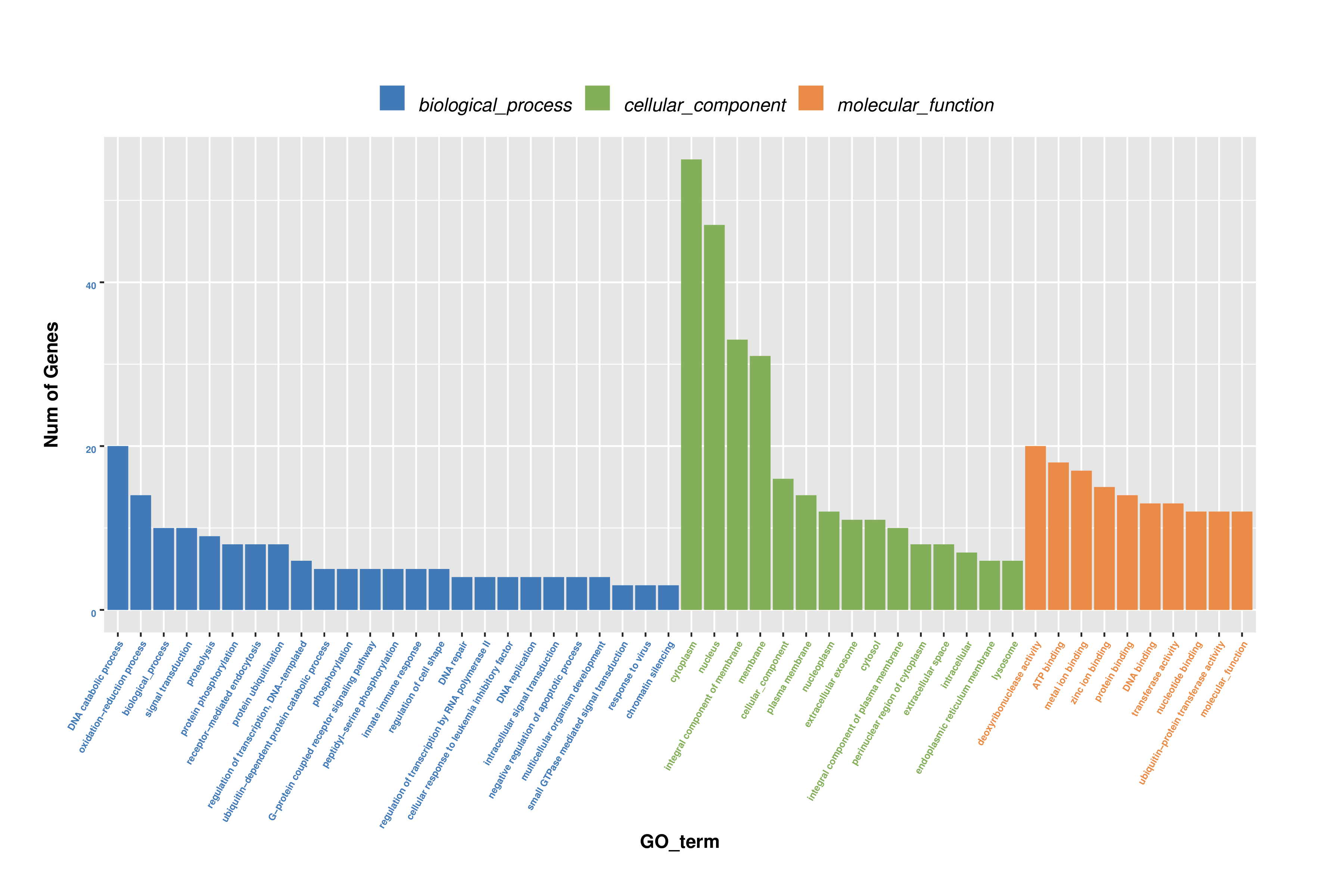

Supplement: Supplemental Information 5 — Enrichment for three GO ontologies, biological process, cellular component, and molecular function are provided. [file peerj-09-10654-s005.png]

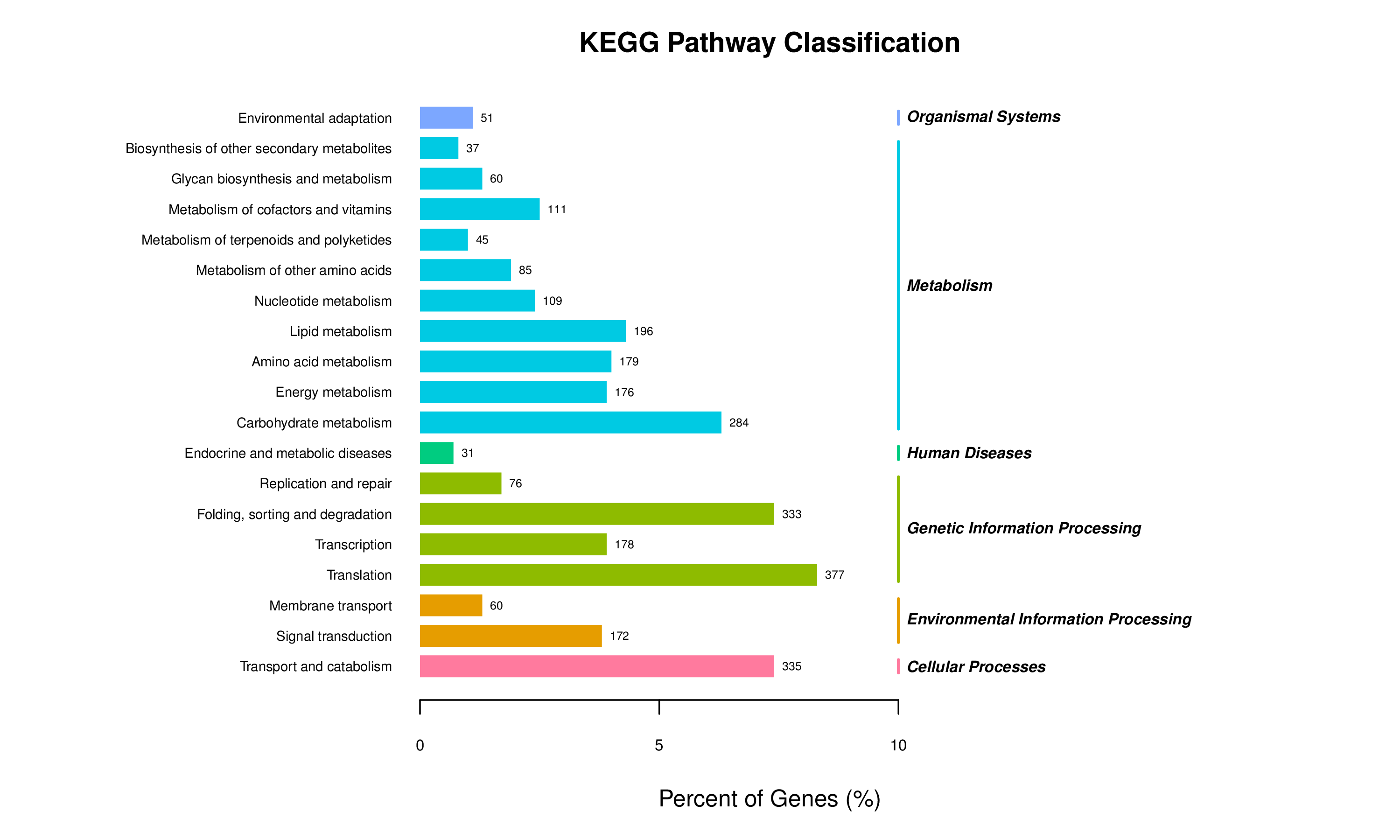

Supplement: Supplemental Information 6 — Categories of KEGG pathways for genes expressed in both sponge host and algal symbiont are shown for pathways with highest numbers of genes represented. [file peerj-09-10654-s006.png]

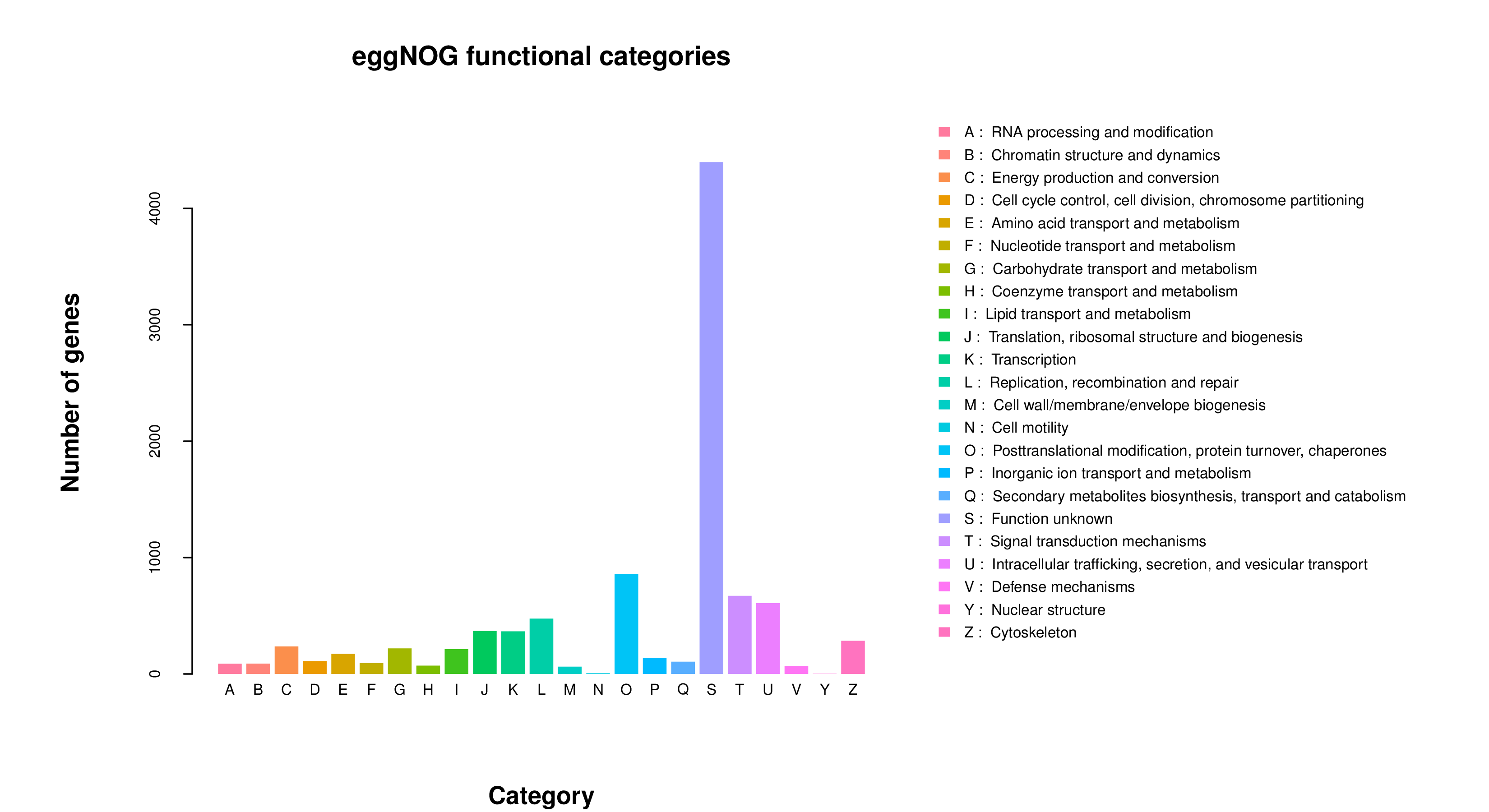

Supplement: Supplemental Information 7 — Numbers of sponge and algal genes predicted in each of 23 eggNOG functional categories. [file peerj-09-10654-s007.png]

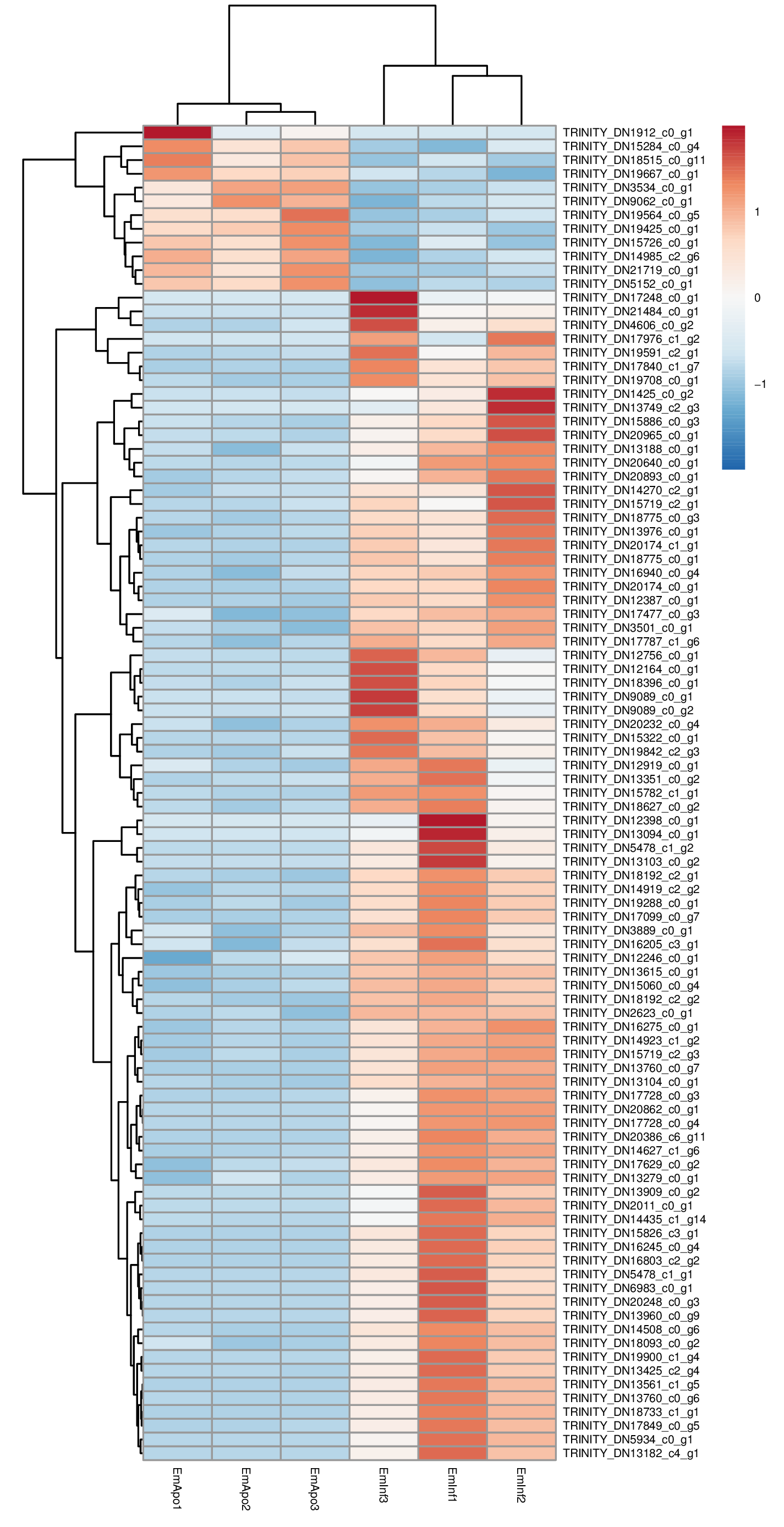

Supplement: Supplemental Information 8 — Relative expression of differentially expressed genes (scale at right) comparing triplicate samples for aposymbiotic and 24 h post-infected sponges from the de novo assembly analysis. Transcripts from sponge host and algal symbiont are represented. [file peerj-09-10654-s008.png]

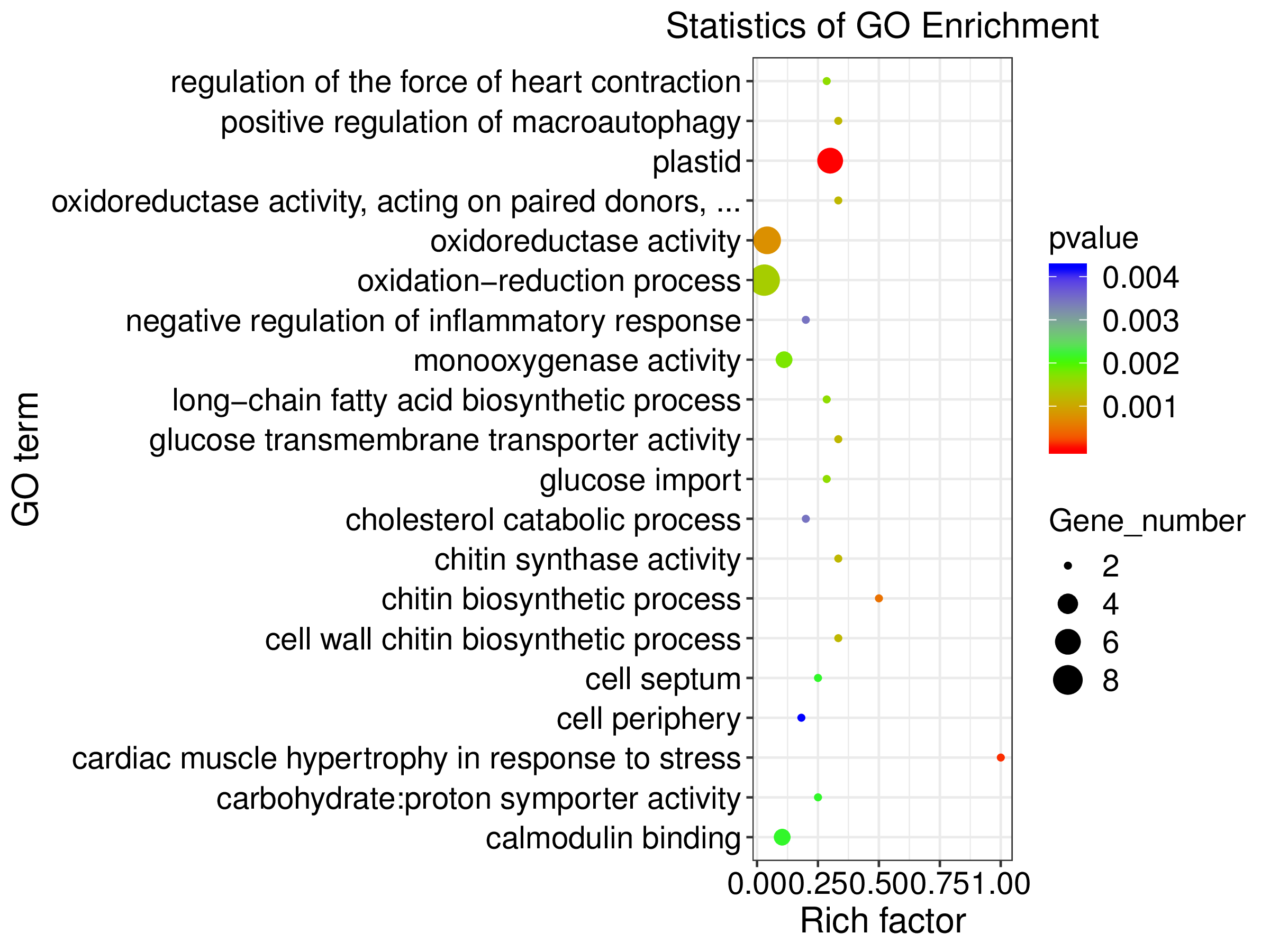

Supplement: Supplemental Information 9 — Statistics of gene ontology (GO) enrichment across most represented GO categories from de novo transcriptome assembly analysis for host and symbiont expressed genes. Enrichment is reported across the differential gene expression analysis for aposymbiotic and 24 h post-infection E muelleri. Size of dots correspond to gene number while colors correspond to p values. Scales are given on the right. [file peerj-09-10654-s009.png]

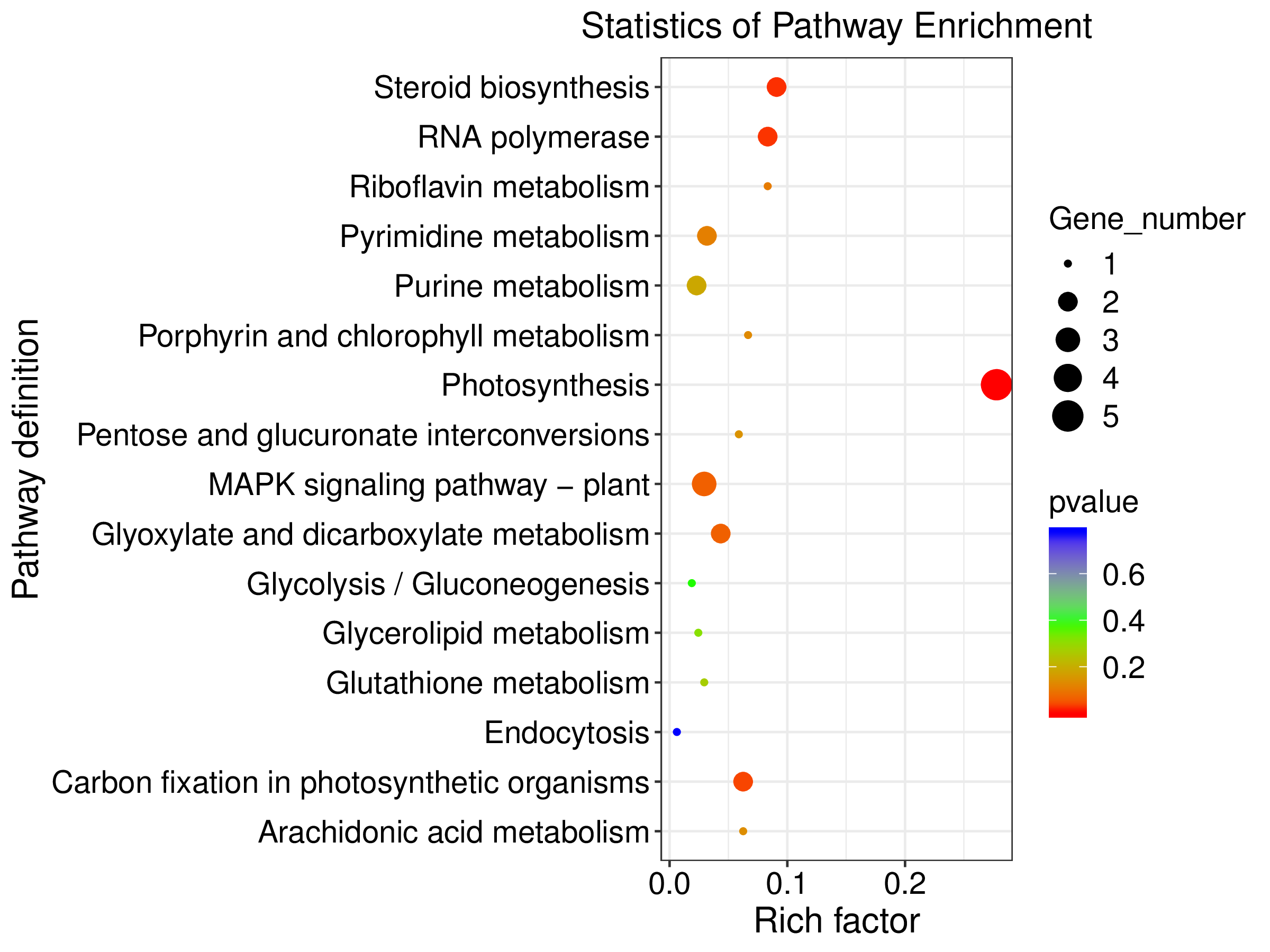

Supplement: Supplemental Information 10 — Statistics of KEGG pathway enrichment across most represented KEGG pathways from de novo transcriptome assembly analysis for host and symbiont expressed genes. Enrichment is reported across the differential gene expression analysis for aposymbiotic and 24 h post-infection E muelleri. Size of dots correspond to gene number while colors correspond to p values. Scales are given on the right. [file peerj-09-10654-s010.png]

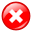

Supplement: Supplemental Information 24 [file peerj-09-10654-s024.zip › EmApo1_Clean_Data1.fq_fastqc/Icons/error.png]

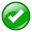

Supplement: Supplemental Information 24 [file peerj-09-10654-s024.zip › EmApo1_Clean_Data1.fq_fastqc/Icons/tick.png]

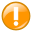

Supplement: Supplemental Information 24 [file peerj-09-10654-s024.zip › EmApo1_Clean_Data1.fq_fastqc/Icons/warning.png]

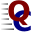

Supplement: Supplemental Information 24 [file peerj-09-10654-s024.zip › EmApo1_Clean_Data1.fq_fastqc/Icons/fastqc_icon.png]

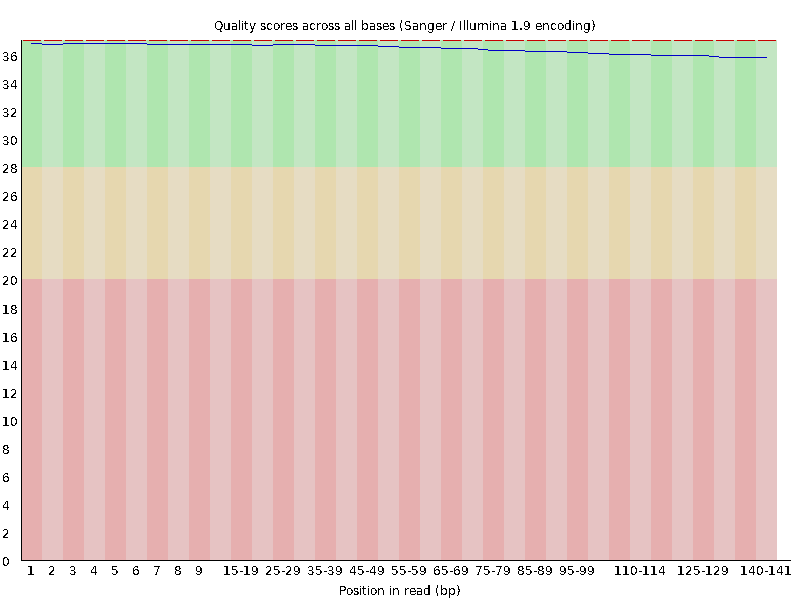

Supplement: Supplemental Information 24 [file peerj-09-10654-s024.zip › EmApo1_Clean_Data1.fq_fastqc/Images/per_base_quality.png]

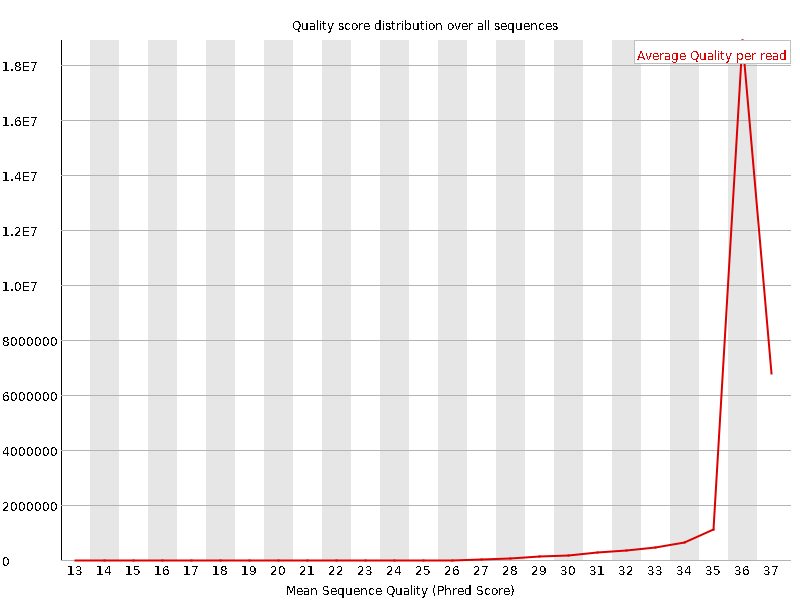

Supplement: Supplemental Information 24 [file peerj-09-10654-s024.zip › EmApo1_Clean_Data1.fq_fastqc/Images/per_sequence_quality.png]

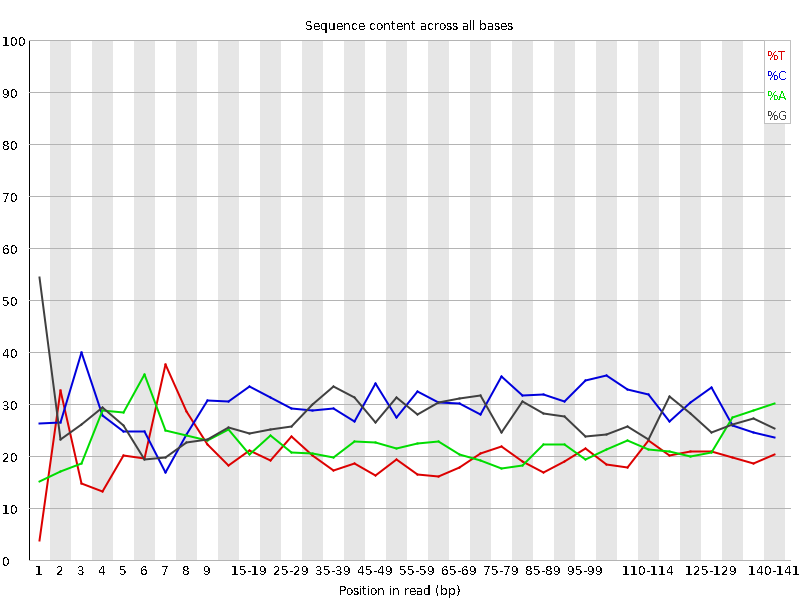

Supplement: Supplemental Information 24 [file peerj-09-10654-s024.zip › EmApo1_Clean_Data1.fq_fastqc/Images/per_base_sequence_content.png]

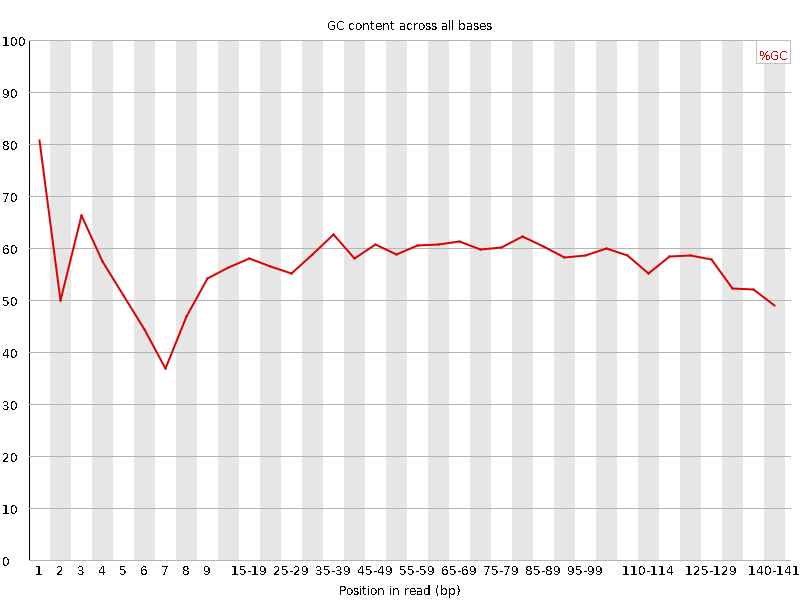

Supplement: Supplemental Information 24 [file peerj-09-10654-s024.zip › EmApo1_Clean_Data1.fq_fastqc/Images/per_base_gc_content.png]

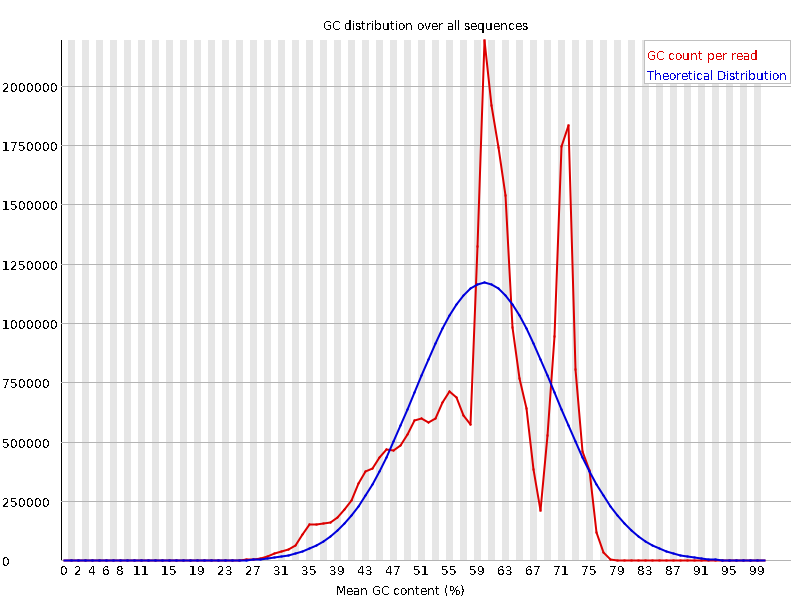

Supplement: Supplemental Information 24 [file peerj-09-10654-s024.zip › EmApo1_Clean_Data1.fq_fastqc/Images/per_sequence_gc_content.png]

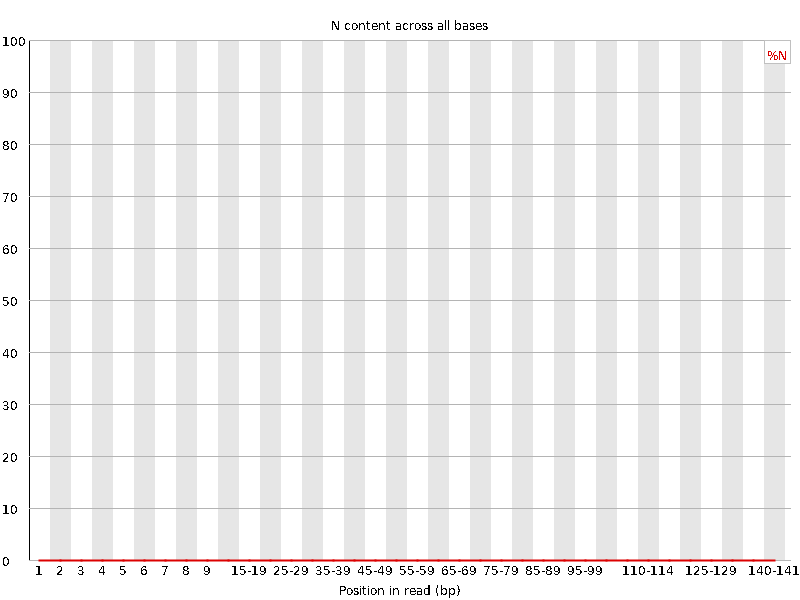

Supplement: Supplemental Information 24 [file peerj-09-10654-s024.zip › EmApo1_Clean_Data1.fq_fastqc/Images/per_base_n_content.png]

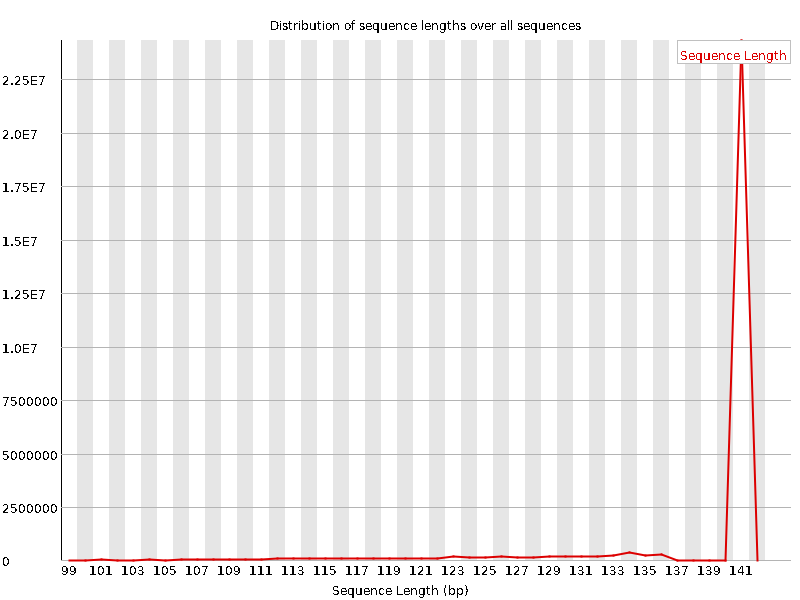

Supplement: Supplemental Information 24 [file peerj-09-10654-s024.zip › EmApo1_Clean_Data1.fq_fastqc/Images/sequence_length_distribution.png]

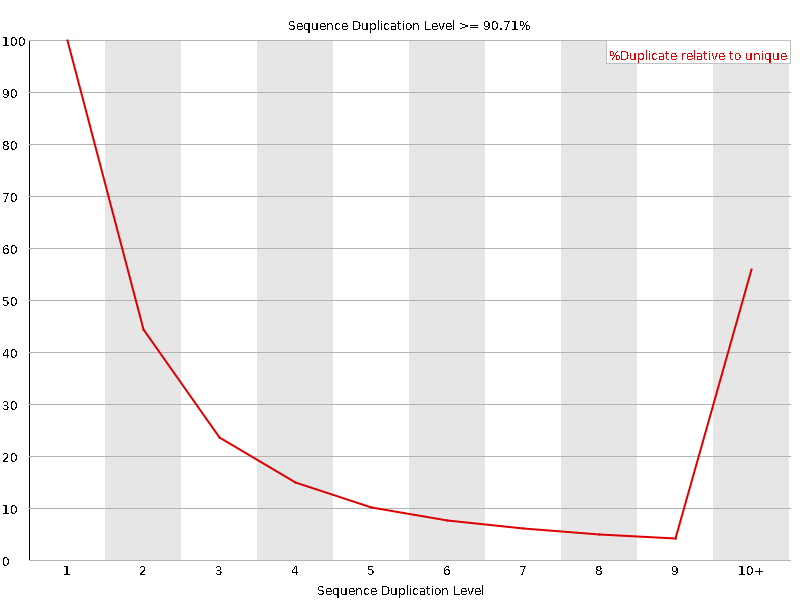

Supplement: Supplemental Information 24 [file peerj-09-10654-s024.zip › EmApo1_Clean_Data1.fq_fastqc/Images/duplication_levels.png]

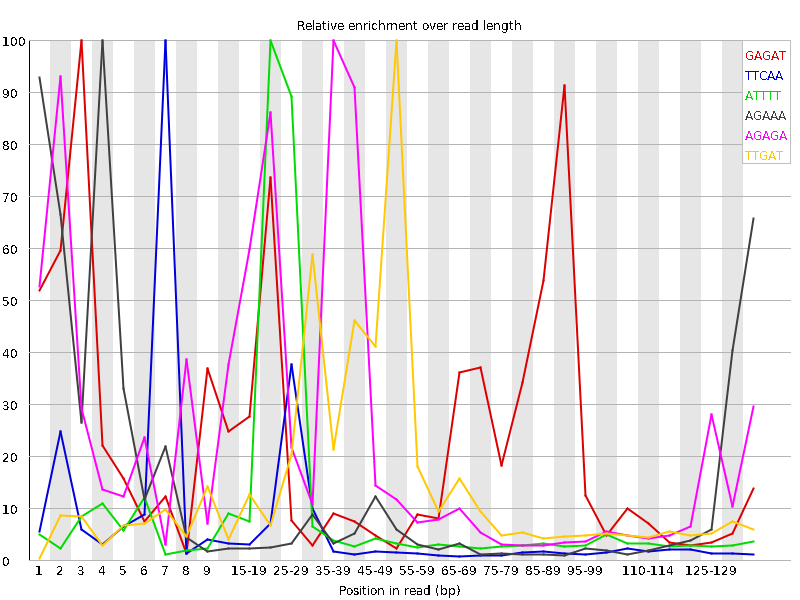

Supplement: Supplemental Information 24 [file peerj-09-10654-s024.zip › EmApo1_Clean_Data1.fq_fastqc/Images/kmer_profiles.png]

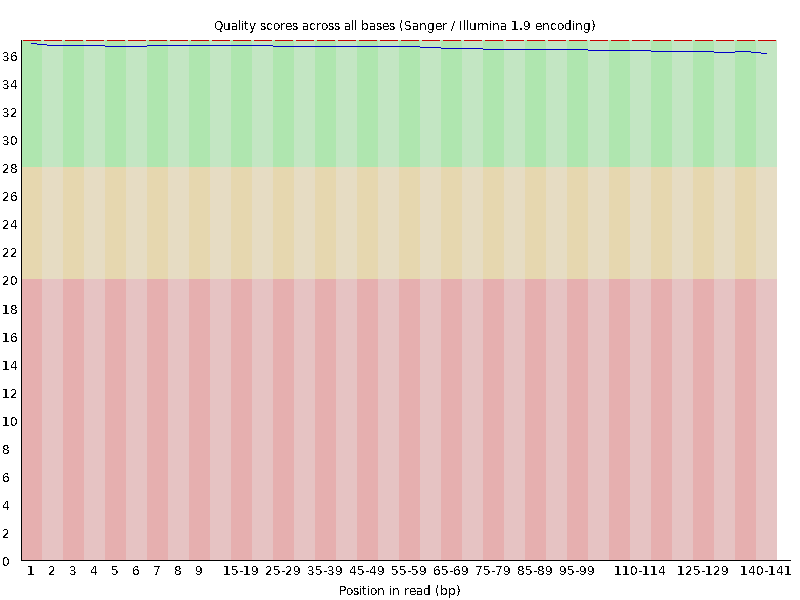

Supplement: Supplemental Information 25 [file peerj-09-10654-s025.zip › EmApo1_Clean_Data2.fq_fastqc/Images/per_base_quality.png]

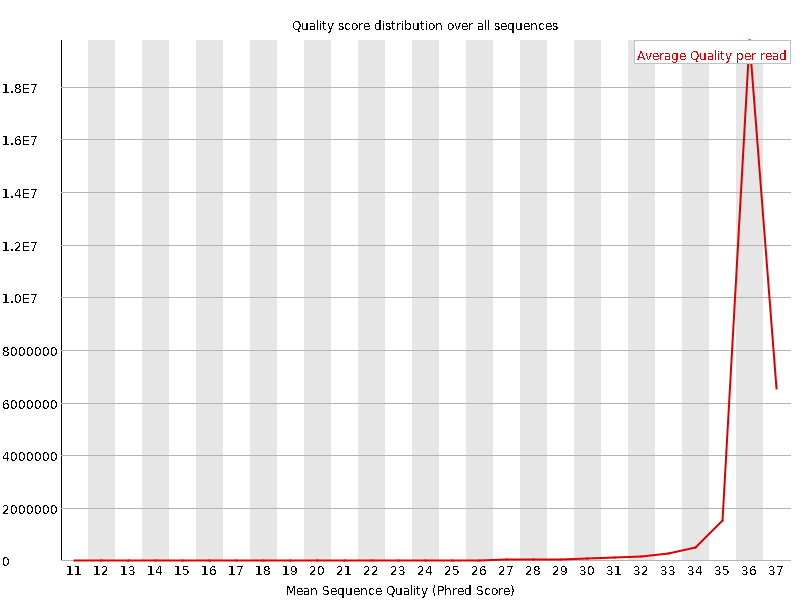

Supplement: Supplemental Information 25 [file peerj-09-10654-s025.zip › EmApo1_Clean_Data2.fq_fastqc/Images/per_sequence_quality.png]

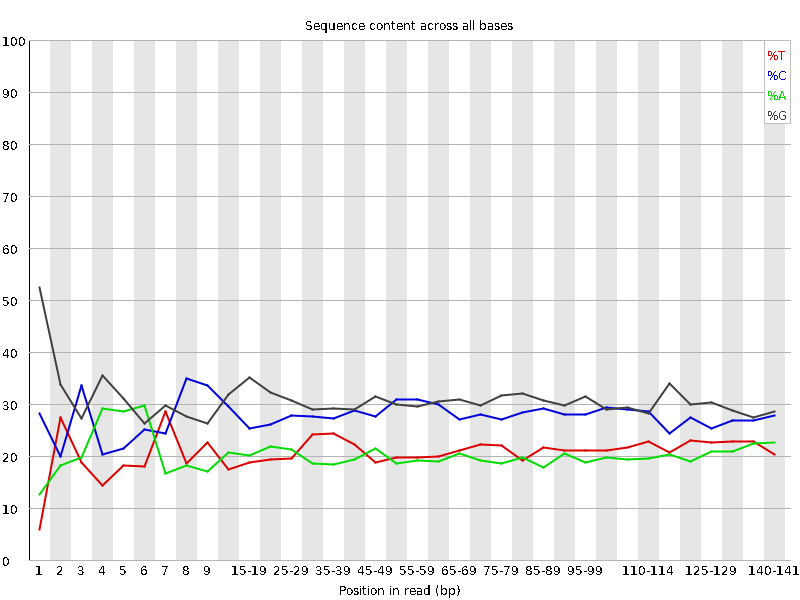

Supplement: Supplemental Information 25 [file peerj-09-10654-s025.zip › EmApo1_Clean_Data2.fq_fastqc/Images/per_base_sequence_content.png]

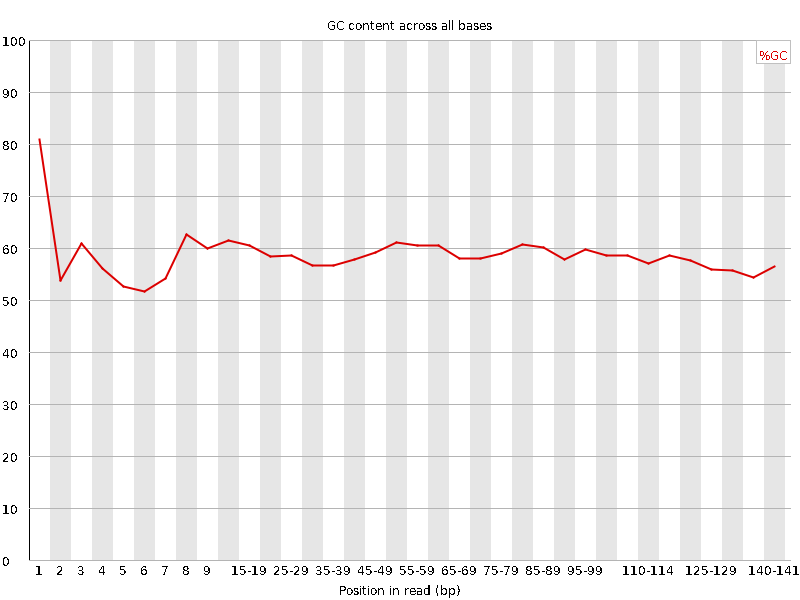

Supplement: Supplemental Information 25 [file peerj-09-10654-s025.zip › EmApo1_Clean_Data2.fq_fastqc/Images/per_base_gc_content.png]

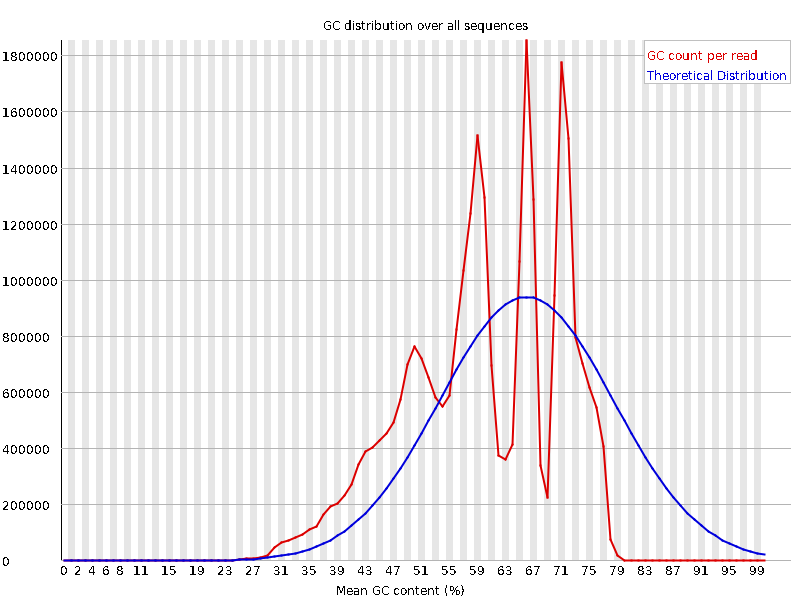

Supplement: Supplemental Information 25 [file peerj-09-10654-s025.zip › EmApo1_Clean_Data2.fq_fastqc/Images/per_sequence_gc_content.png]

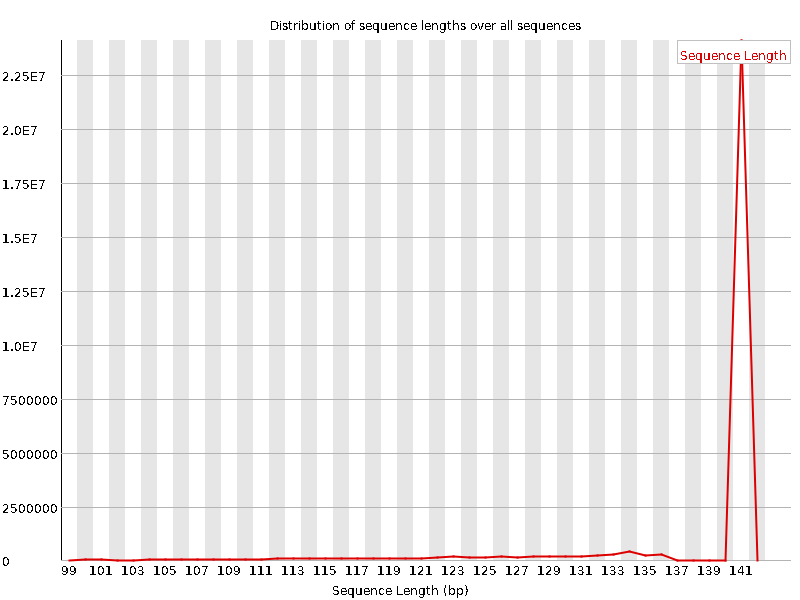

Supplement: Supplemental Information 25 [file peerj-09-10654-s025.zip › EmApo1_Clean_Data2.fq_fastqc/Images/sequence_length_distribution.png]

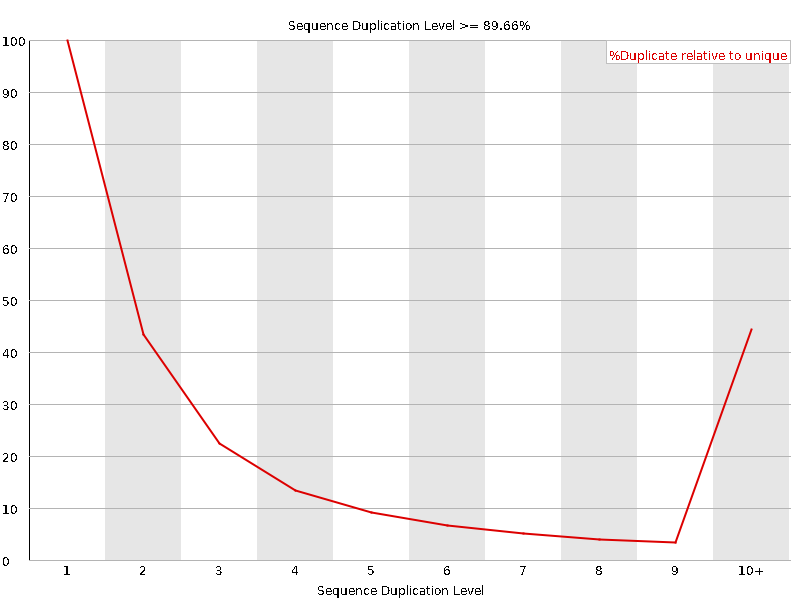

Supplement: Supplemental Information 25 [file peerj-09-10654-s025.zip › EmApo1_Clean_Data2.fq_fastqc/Images/duplication_levels.png]

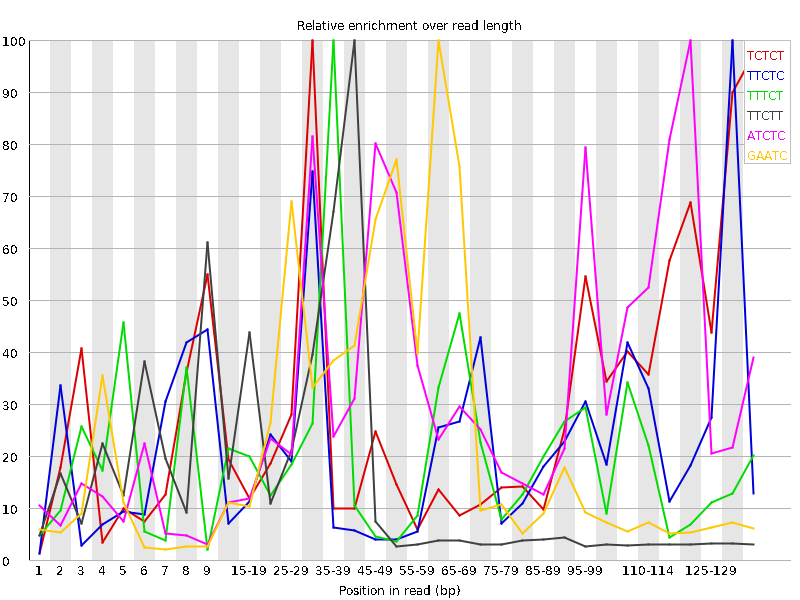

Supplement: Supplemental Information 25 [file peerj-09-10654-s025.zip › EmApo1_Clean_Data2.fq_fastqc/Images/kmer_profiles.png]

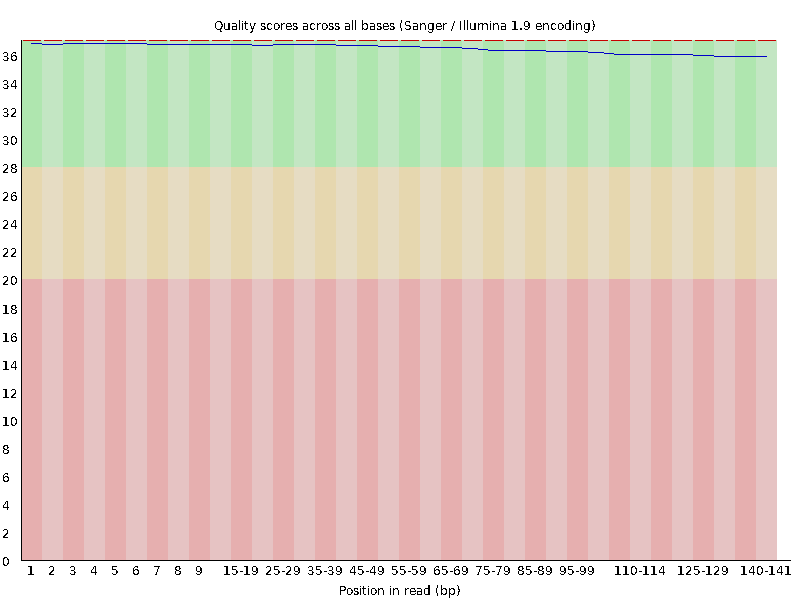

Supplement: Supplemental Information 26 [file peerj-09-10654-s026.zip › EmApo2_Clean_Data1.fq_fastqc/Images/per_base_quality.png]

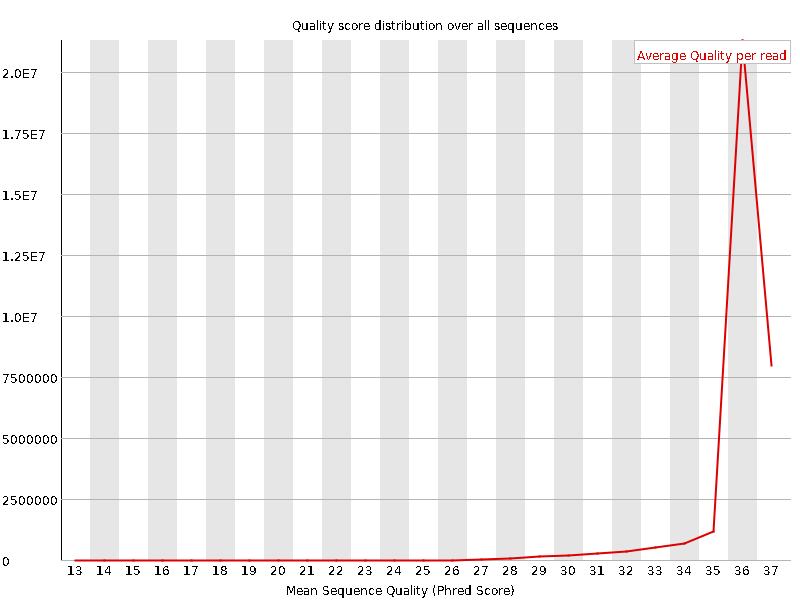

Supplement: Supplemental Information 26 [file peerj-09-10654-s026.zip › EmApo2_Clean_Data1.fq_fastqc/Images/per_sequence_quality.png]

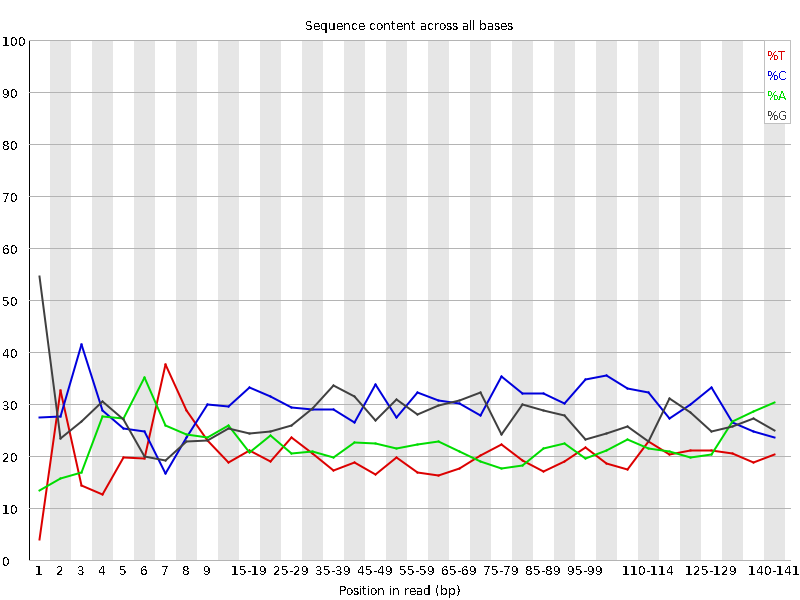

Supplement: Supplemental Information 26 [file peerj-09-10654-s026.zip › EmApo2_Clean_Data1.fq_fastqc/Images/per_base_sequence_content.png]

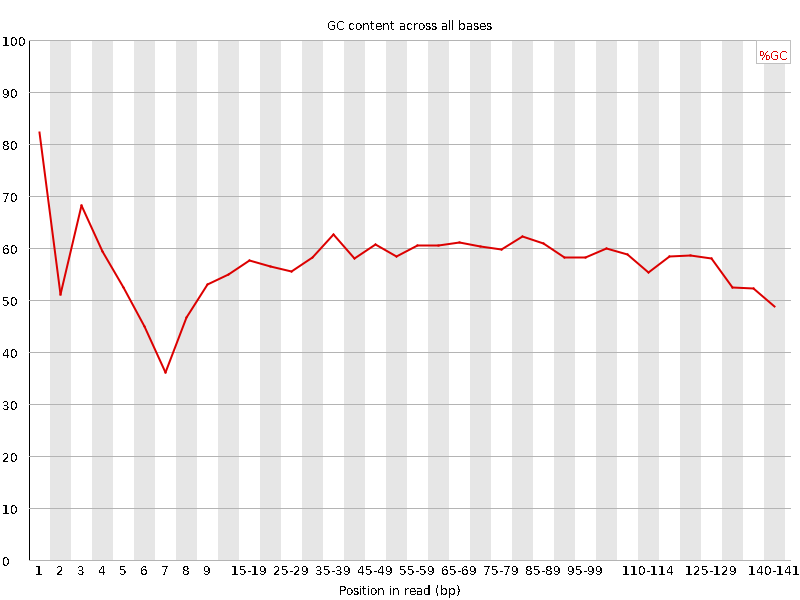

Supplement: Supplemental Information 26 [file peerj-09-10654-s026.zip › EmApo2_Clean_Data1.fq_fastqc/Images/per_base_gc_content.png]

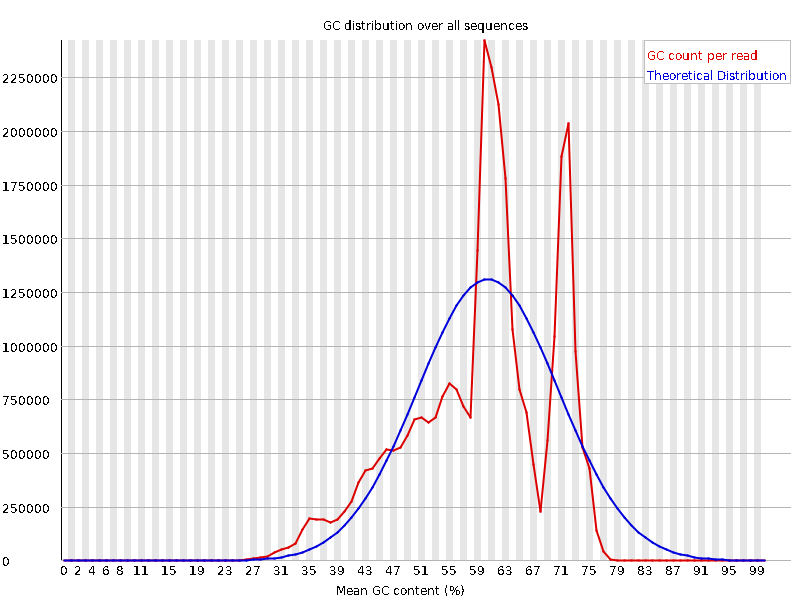

Supplement: Supplemental Information 26 [file peerj-09-10654-s026.zip › EmApo2_Clean_Data1.fq_fastqc/Images/per_sequence_gc_content.png]

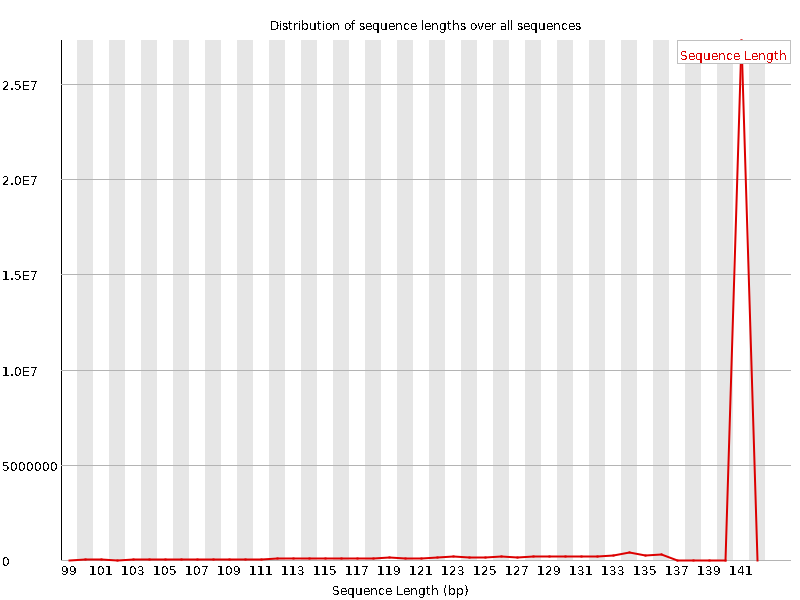

Supplement: Supplemental Information 26 [file peerj-09-10654-s026.zip › EmApo2_Clean_Data1.fq_fastqc/Images/sequence_length_distribution.png]

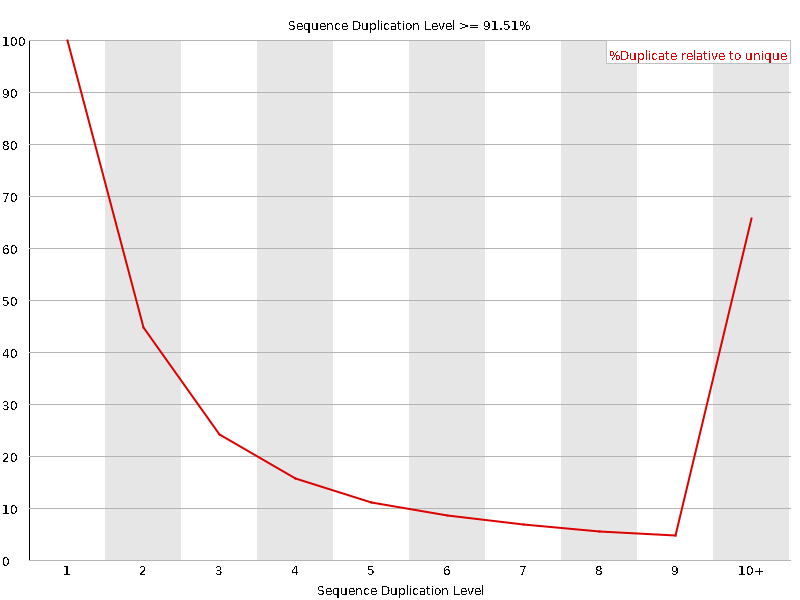

Supplement: Supplemental Information 26 [file peerj-09-10654-s026.zip › EmApo2_Clean_Data1.fq_fastqc/Images/duplication_levels.png]

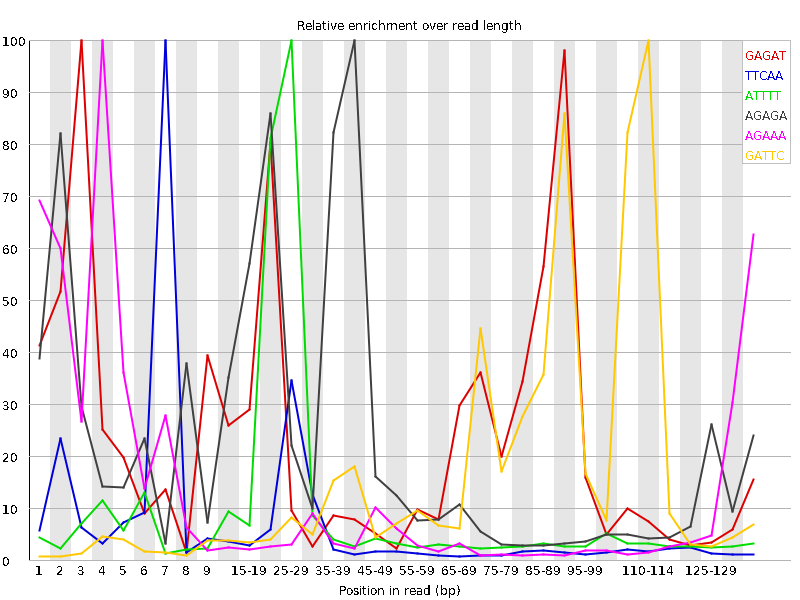

Supplement: Supplemental Information 26 [file peerj-09-10654-s026.zip › EmApo2_Clean_Data1.fq_fastqc/Images/kmer_profiles.png]

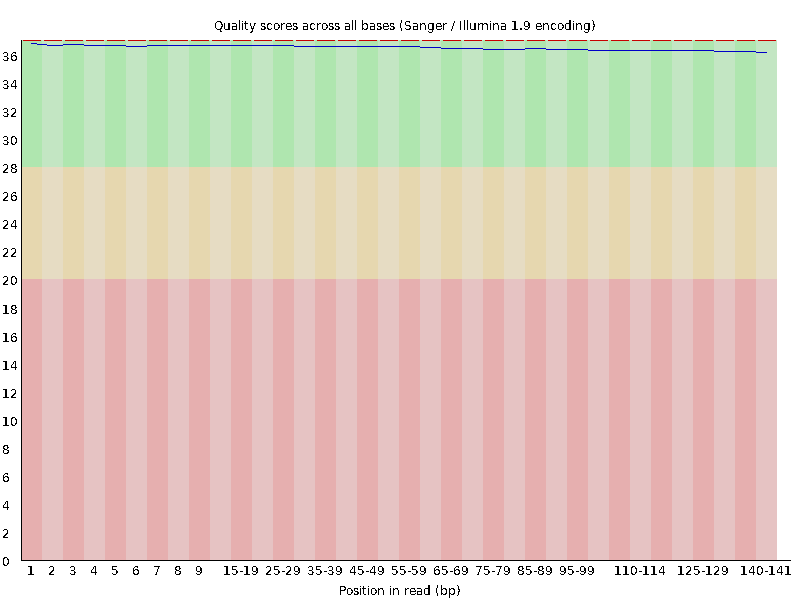

Supplement: Supplemental Information 27 [file peerj-09-10654-s027.zip › EmApo2_Clean_Data2.fq_fastqc/Images/per_base_quality.png]

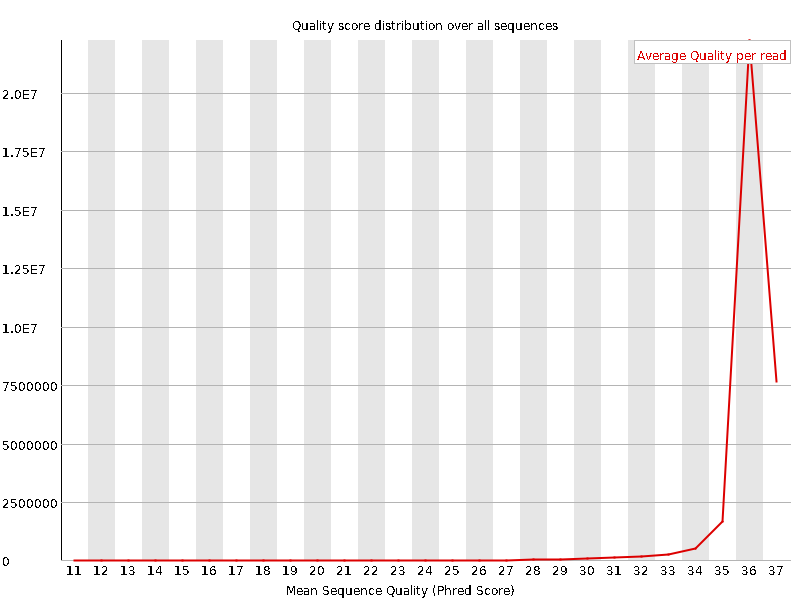

Supplement: Supplemental Information 27 [file peerj-09-10654-s027.zip › EmApo2_Clean_Data2.fq_fastqc/Images/per_sequence_quality.png]

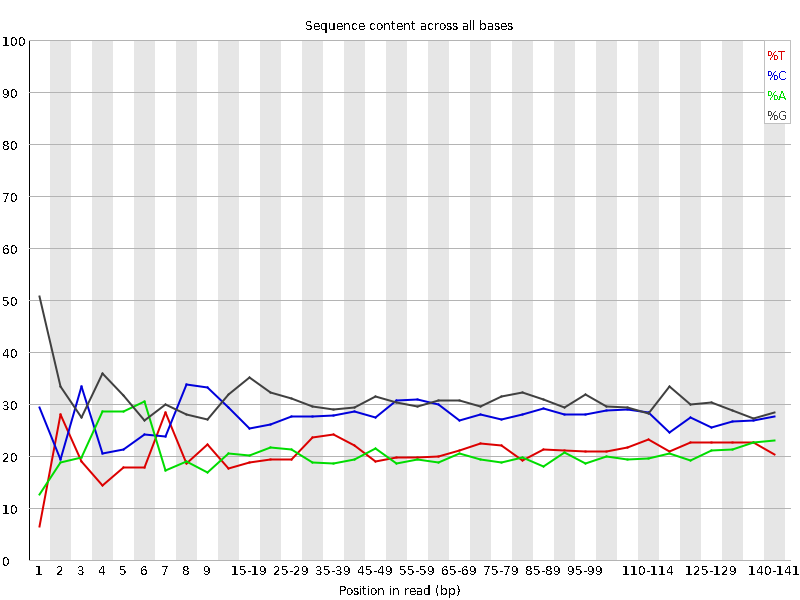

Supplement: Supplemental Information 27 [file peerj-09-10654-s027.zip › EmApo2_Clean_Data2.fq_fastqc/Images/per_base_sequence_content.png]

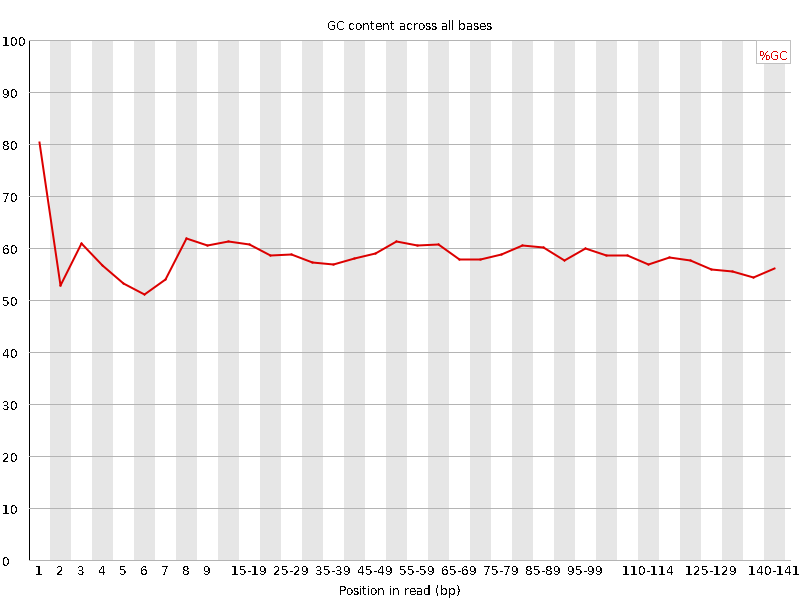

Supplement: Supplemental Information 27 [file peerj-09-10654-s027.zip › EmApo2_Clean_Data2.fq_fastqc/Images/per_base_gc_content.png]

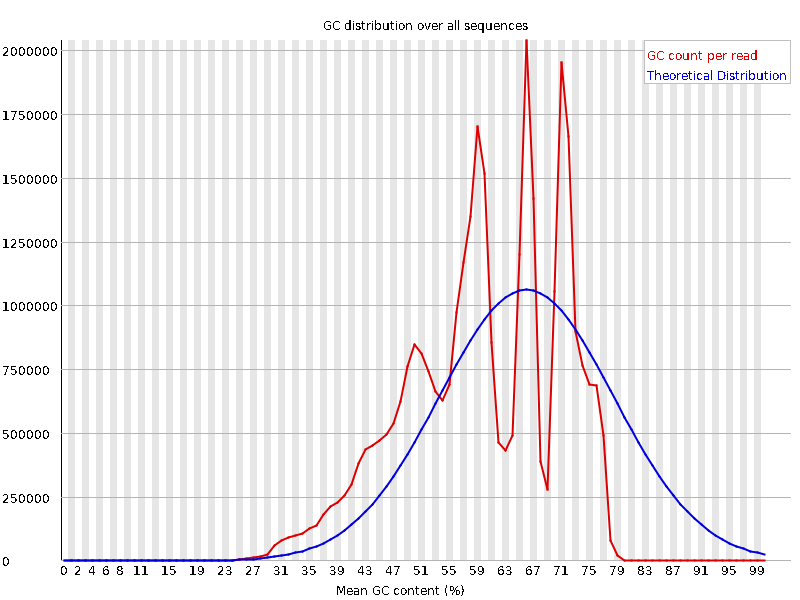

Supplement: Supplemental Information 27 [file peerj-09-10654-s027.zip › EmApo2_Clean_Data2.fq_fastqc/Images/per_sequence_gc_content.png]

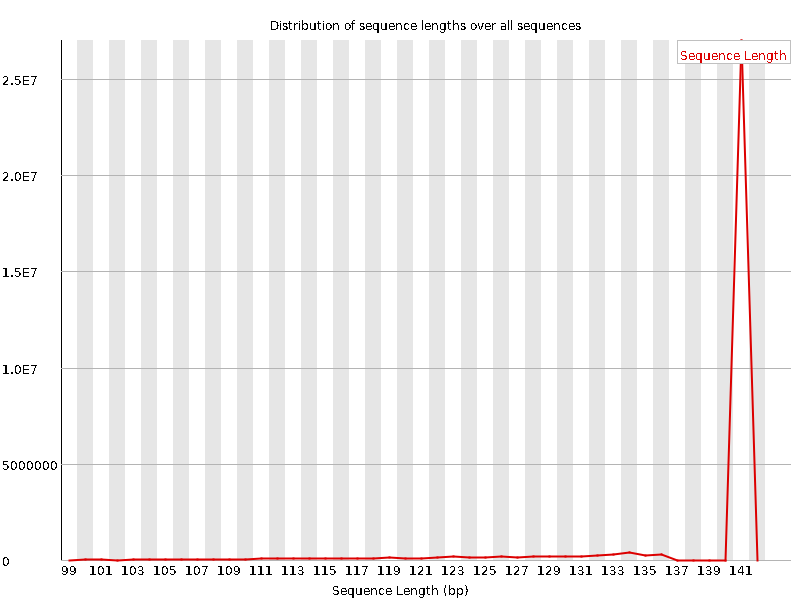

Supplement: Supplemental Information 27 [file peerj-09-10654-s027.zip › EmApo2_Clean_Data2.fq_fastqc/Images/sequence_length_distribution.png]

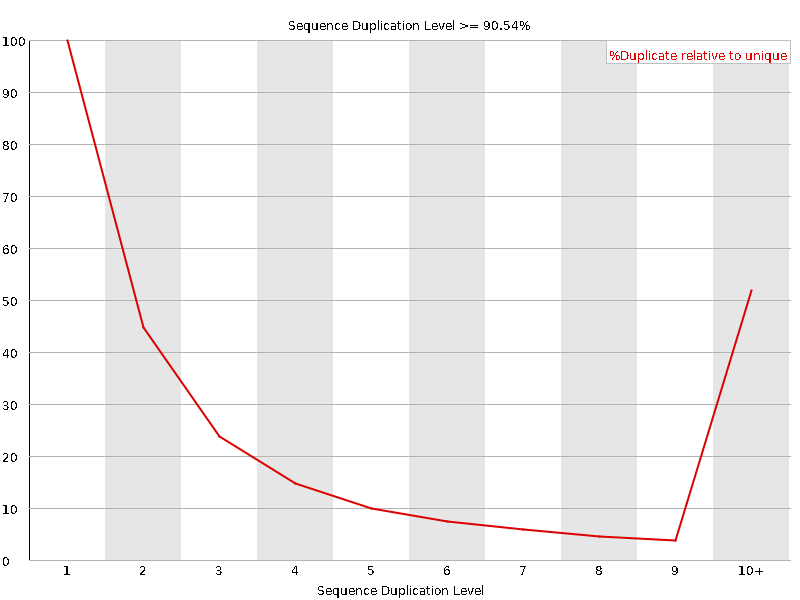

Supplement: Supplemental Information 27 [file peerj-09-10654-s027.zip › EmApo2_Clean_Data2.fq_fastqc/Images/duplication_levels.png]

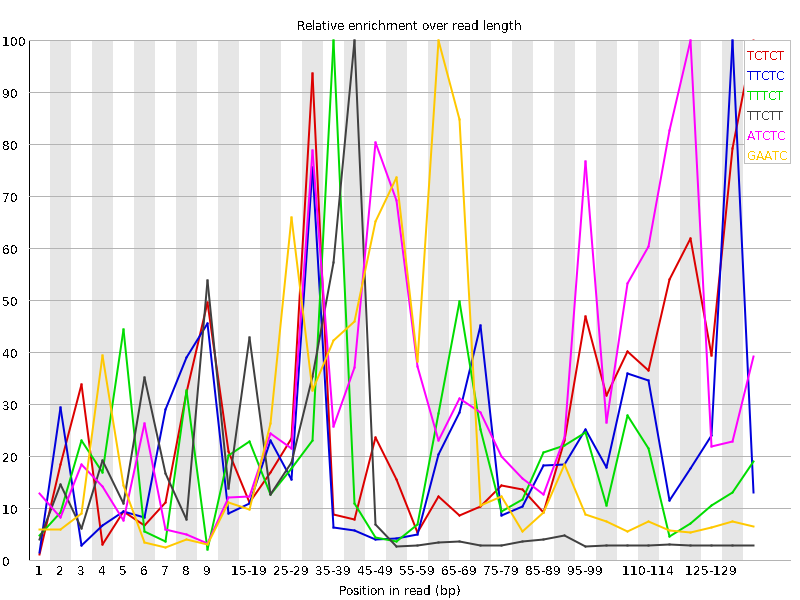

Supplement: Supplemental Information 27 [file peerj-09-10654-s027.zip › EmApo2_Clean_Data2.fq_fastqc/Images/kmer_profiles.png]

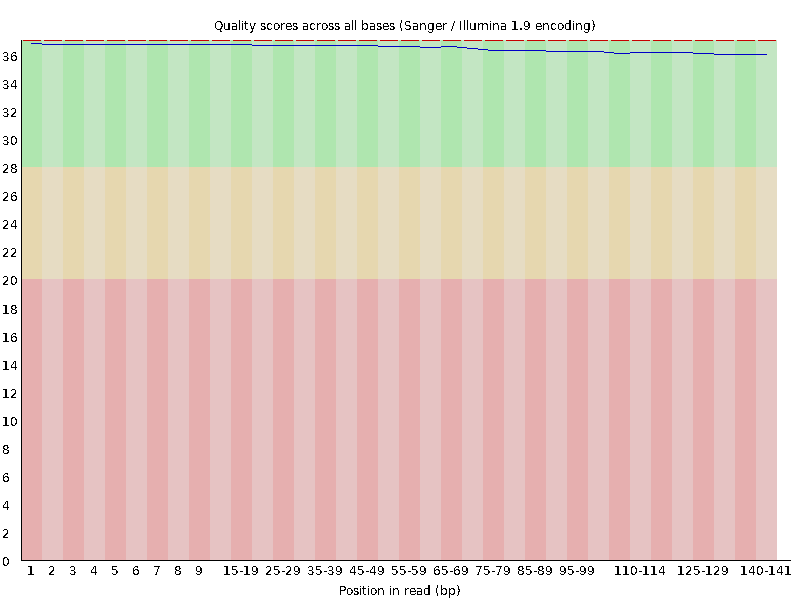

Supplement: Supplemental Information 28 [file peerj-09-10654-s028.zip › EmApo3_Clean_Data1.fq_fastqc/Images/per_base_quality.png]

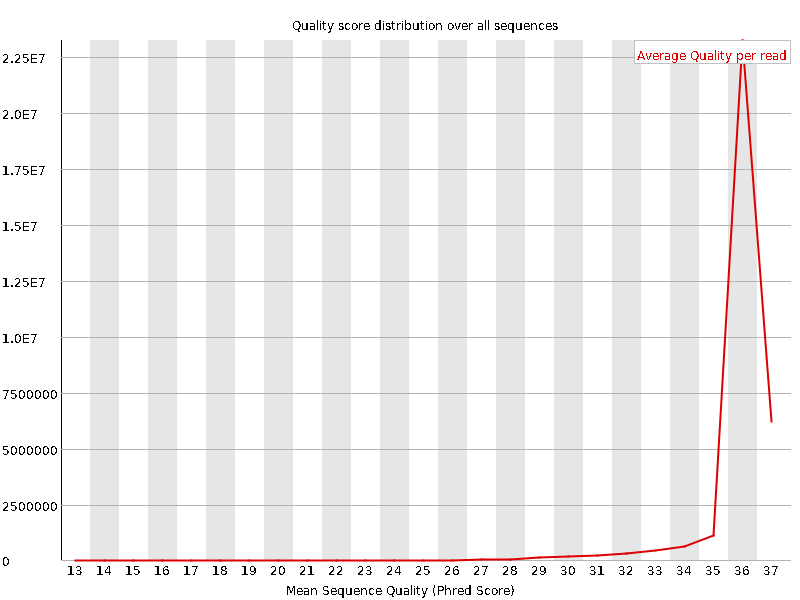

Supplement: Supplemental Information 28 [file peerj-09-10654-s028.zip › EmApo3_Clean_Data1.fq_fastqc/Images/per_sequence_quality.png]

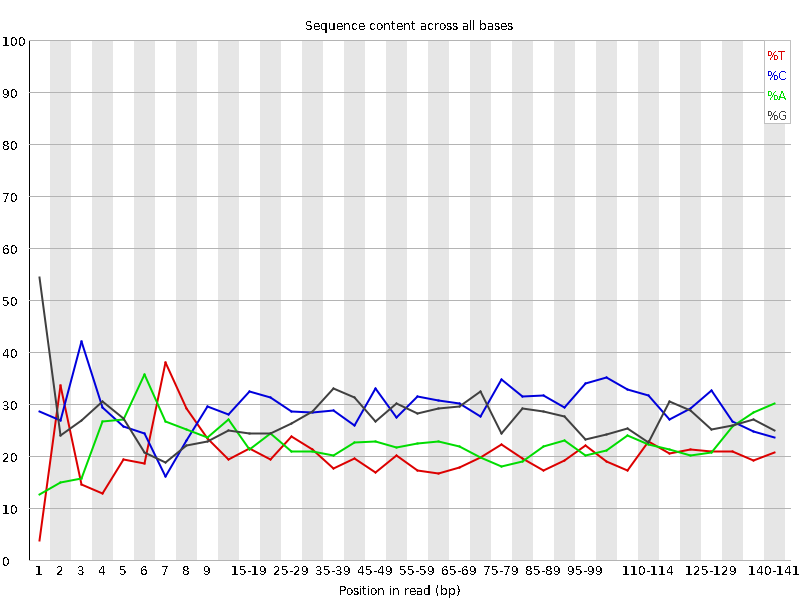

Supplement: Supplemental Information 28 [file peerj-09-10654-s028.zip › EmApo3_Clean_Data1.fq_fastqc/Images/per_base_sequence_content.png]

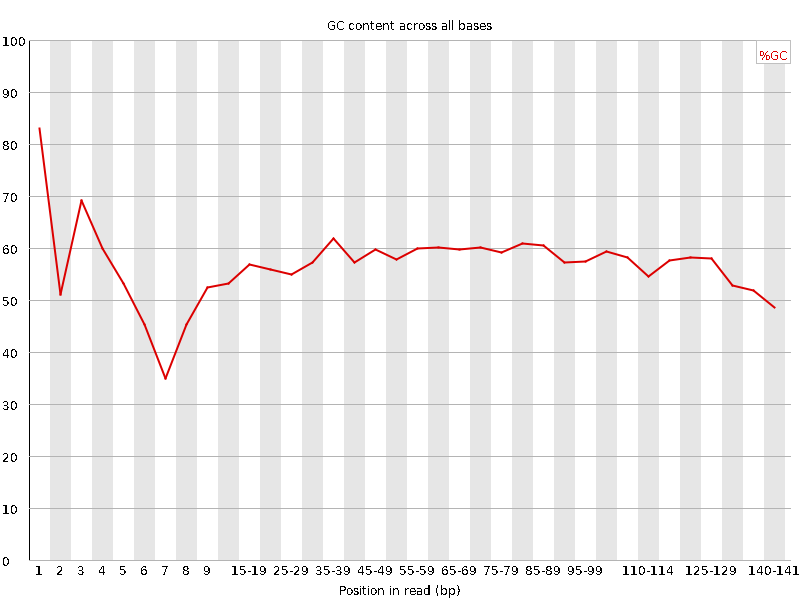

Supplement: Supplemental Information 28 [file peerj-09-10654-s028.zip › EmApo3_Clean_Data1.fq_fastqc/Images/per_base_gc_content.png]

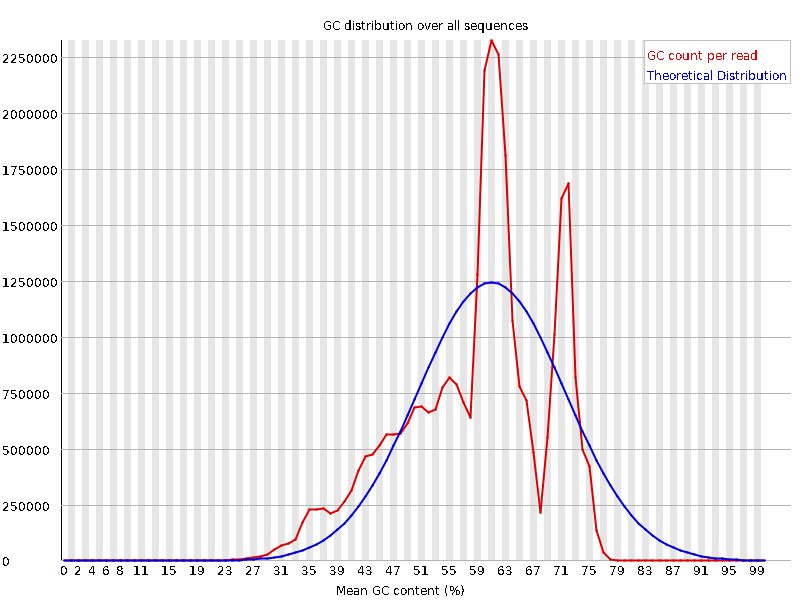

Supplement: Supplemental Information 28 [file peerj-09-10654-s028.zip › EmApo3_Clean_Data1.fq_fastqc/Images/per_sequence_gc_content.png]

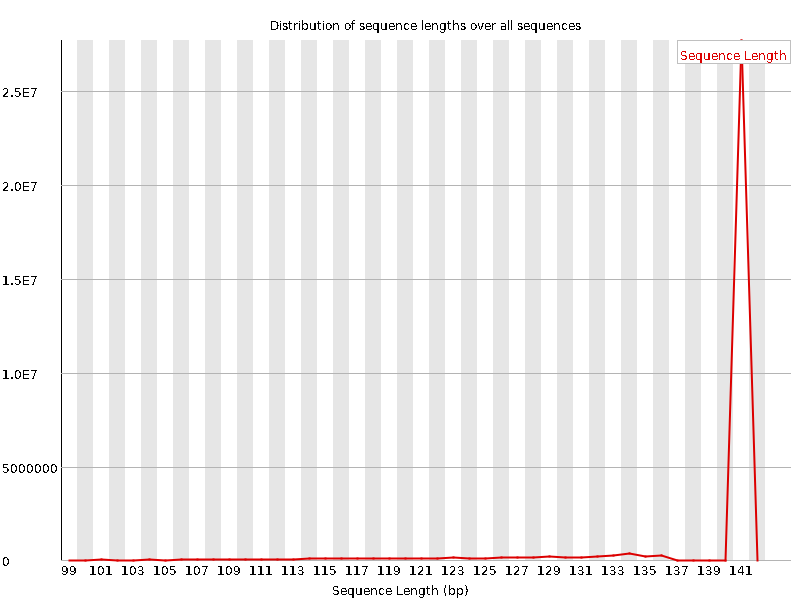

Supplement: Supplemental Information 28 [file peerj-09-10654-s028.zip › EmApo3_Clean_Data1.fq_fastqc/Images/sequence_length_distribution.png]

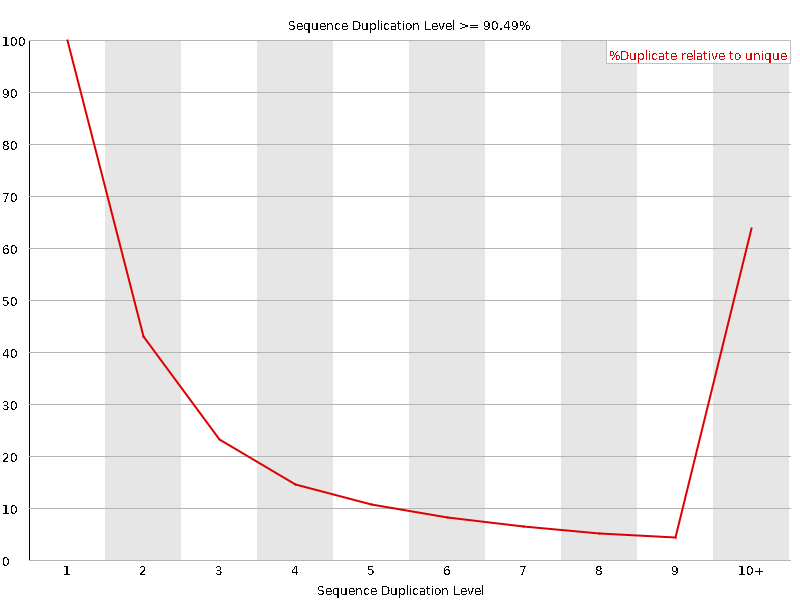

Supplement: Supplemental Information 28 [file peerj-09-10654-s028.zip › EmApo3_Clean_Data1.fq_fastqc/Images/duplication_levels.png]

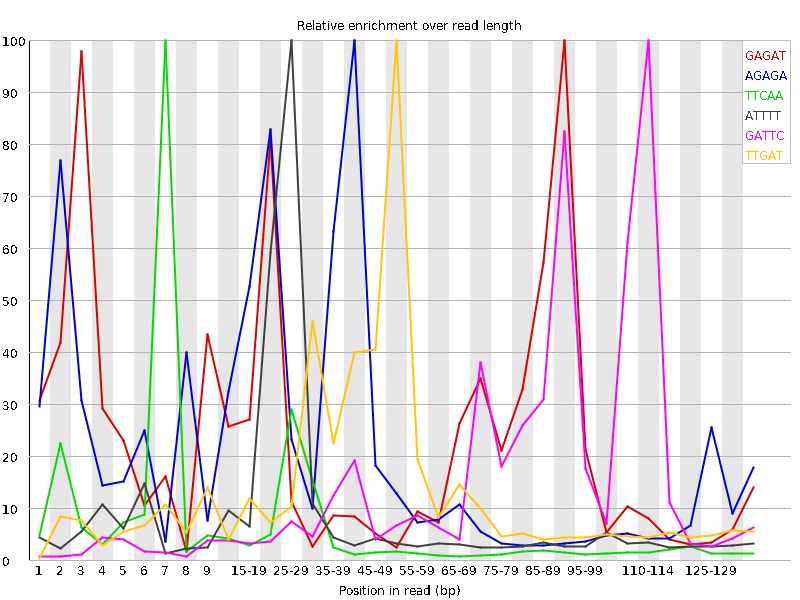

Supplement: Supplemental Information 28 [file peerj-09-10654-s028.zip › EmApo3_Clean_Data1.fq_fastqc/Images/kmer_profiles.png]

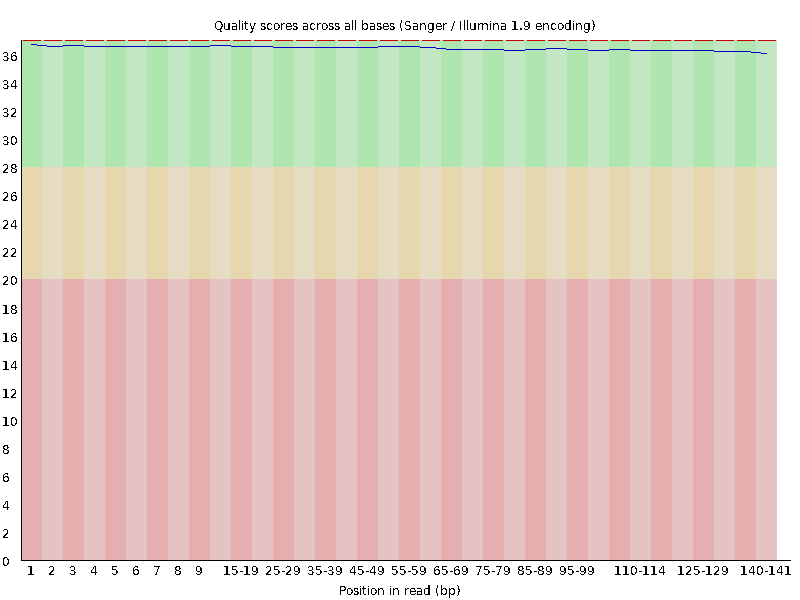

Supplement: Supplemental Information 29 [file peerj-09-10654-s029.zip › EmApo3_Clean_Data2.fq_fastqc/Images/per_base_quality.png]

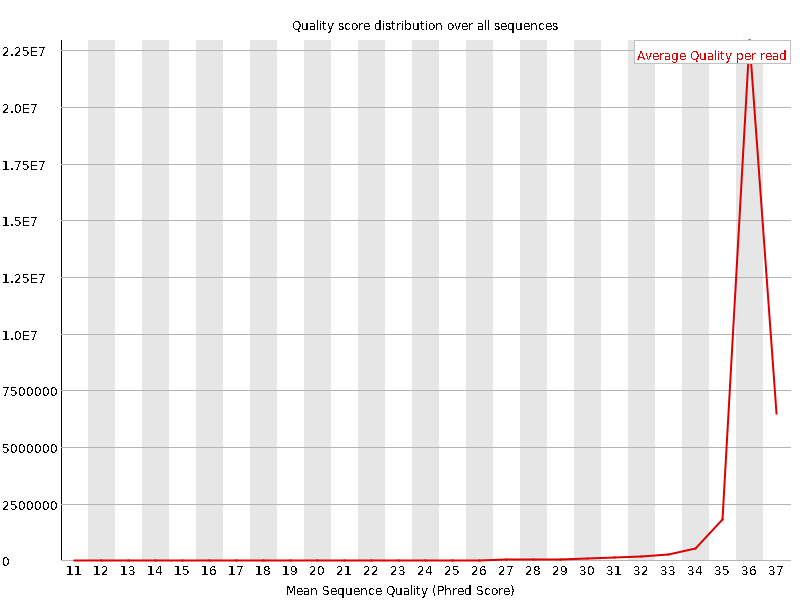

Supplement: Supplemental Information 29 [file peerj-09-10654-s029.zip › EmApo3_Clean_Data2.fq_fastqc/Images/per_sequence_quality.png]

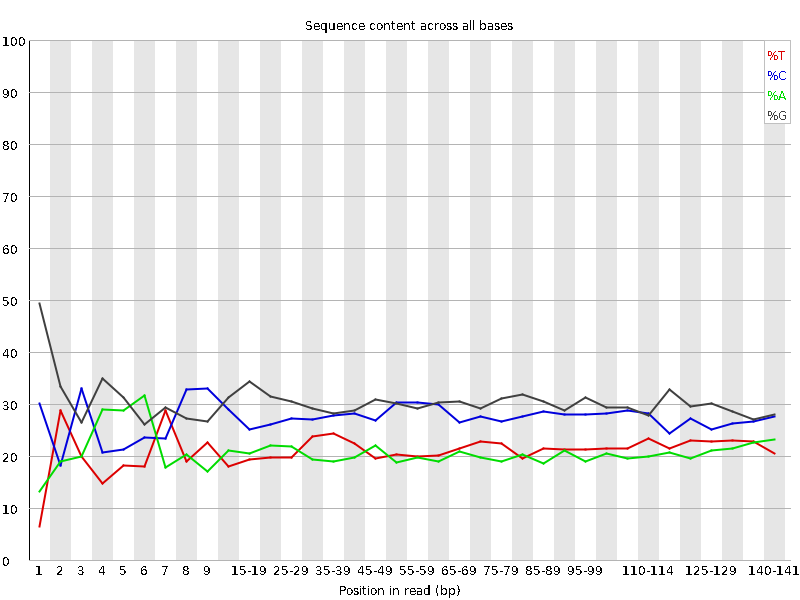

Supplement: Supplemental Information 29 [file peerj-09-10654-s029.zip › EmApo3_Clean_Data2.fq_fastqc/Images/per_base_sequence_content.png]

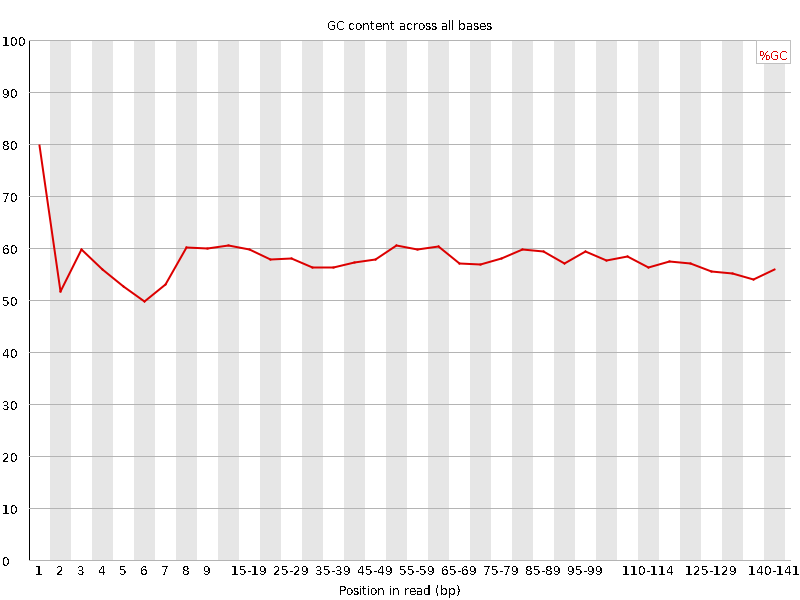

Supplement: Supplemental Information 29 [file peerj-09-10654-s029.zip › EmApo3_Clean_Data2.fq_fastqc/Images/per_base_gc_content.png]

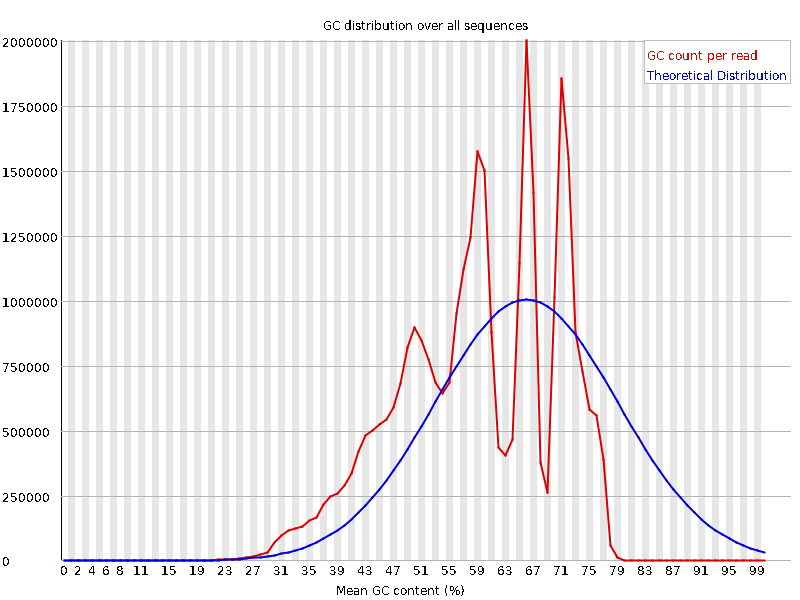

Supplement: Supplemental Information 29 [file peerj-09-10654-s029.zip › EmApo3_Clean_Data2.fq_fastqc/Images/per_sequence_gc_content.png]

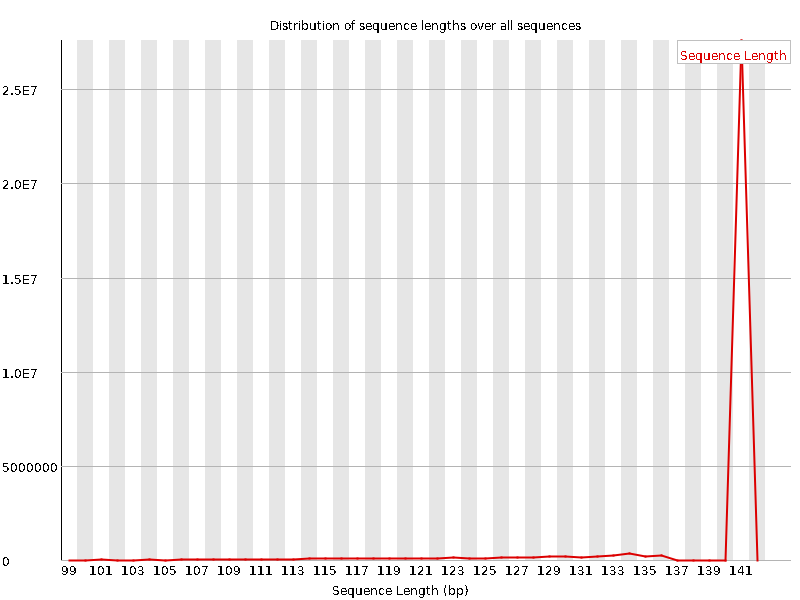

Supplement: Supplemental Information 29 [file peerj-09-10654-s029.zip › EmApo3_Clean_Data2.fq_fastqc/Images/sequence_length_distribution.png]

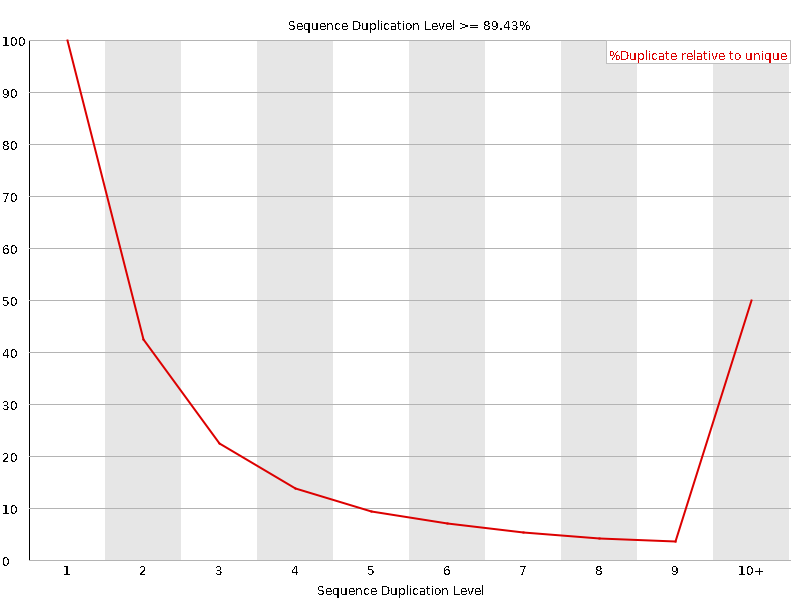

Supplement: Supplemental Information 29 [file peerj-09-10654-s029.zip › EmApo3_Clean_Data2.fq_fastqc/Images/duplication_levels.png]

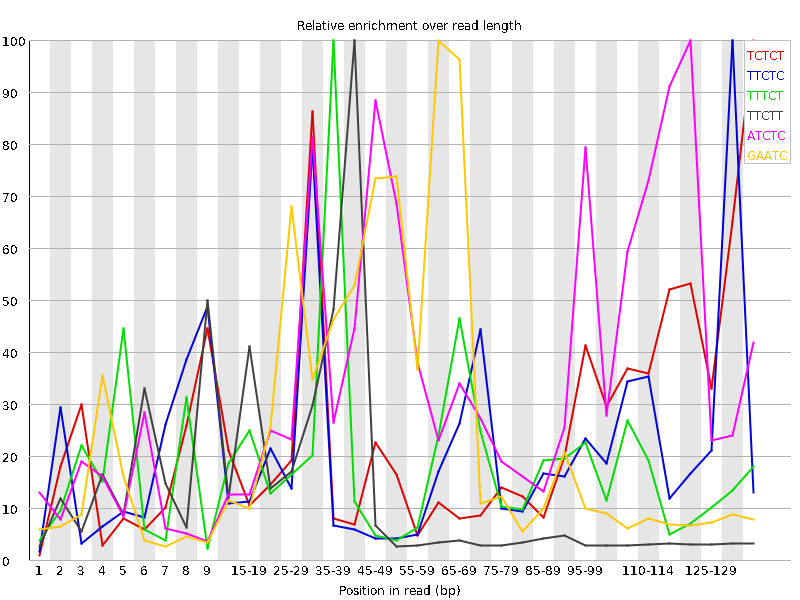

Supplement: Supplemental Information 29 [file peerj-09-10654-s029.zip › EmApo3_Clean_Data2.fq_fastqc/Images/kmer_profiles.png]

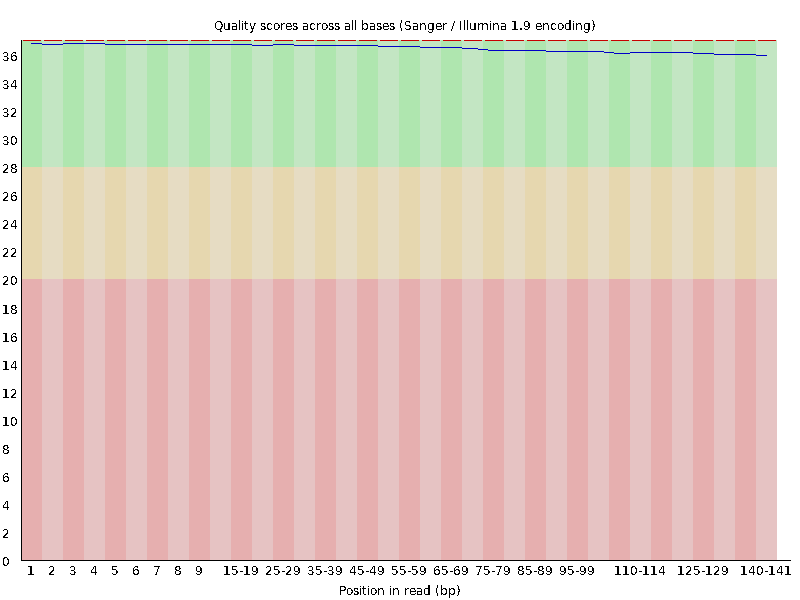

Supplement: Supplemental Information 30 [file peerj-09-10654-s030.zip › EmInf1_Clean_Data1.fq_fastqc/Images/per_base_quality.png]

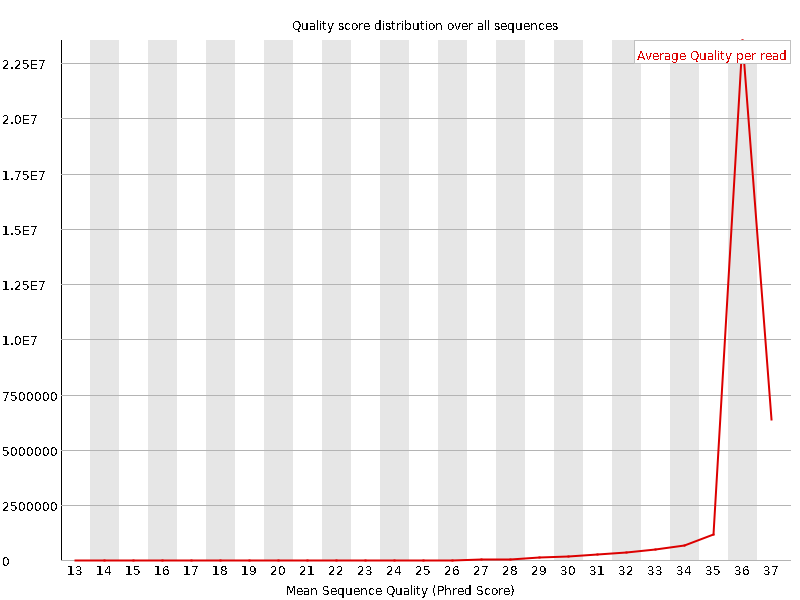

Supplement: Supplemental Information 30 [file peerj-09-10654-s030.zip › EmInf1_Clean_Data1.fq_fastqc/Images/per_sequence_quality.png]

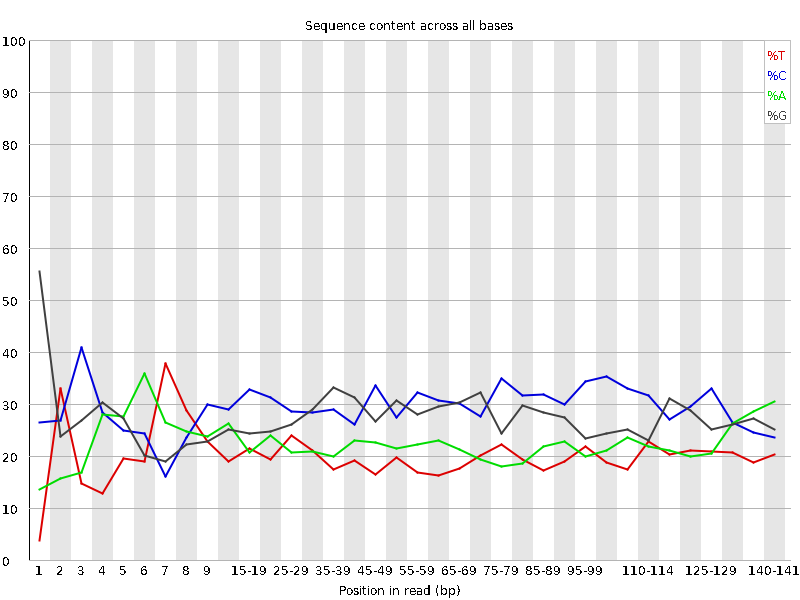

Supplement: Supplemental Information 30 [file peerj-09-10654-s030.zip › EmInf1_Clean_Data1.fq_fastqc/Images/per_base_sequence_content.png]

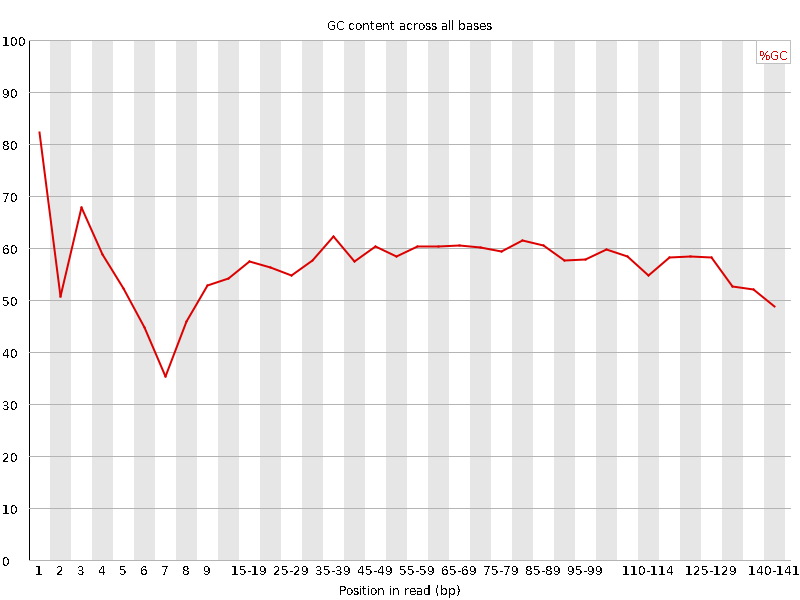

Supplement: Supplemental Information 30 [file peerj-09-10654-s030.zip › EmInf1_Clean_Data1.fq_fastqc/Images/per_base_gc_content.png]

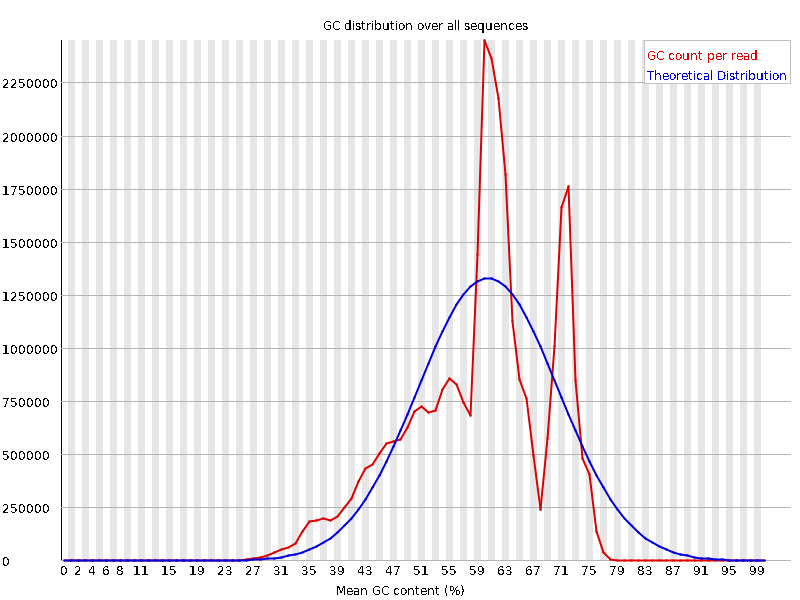

Supplement: Supplemental Information 30 [file peerj-09-10654-s030.zip › EmInf1_Clean_Data1.fq_fastqc/Images/per_sequence_gc_content.png]

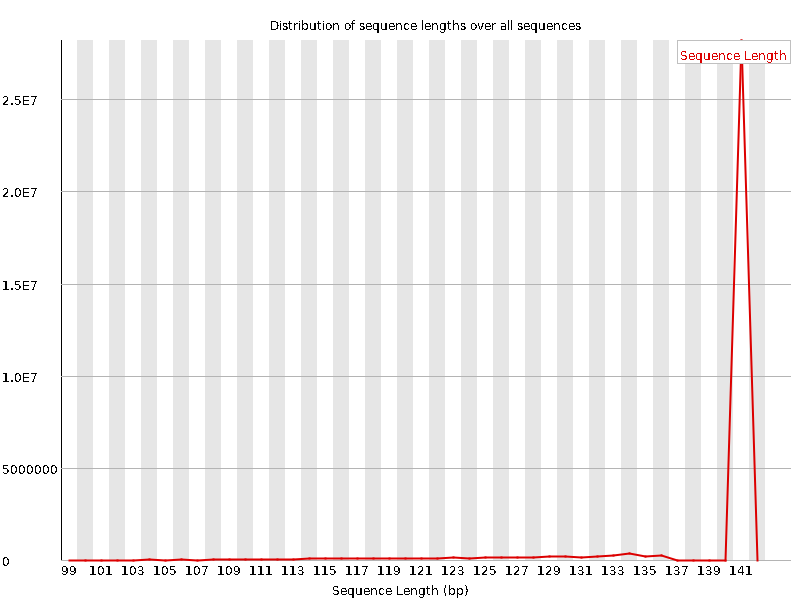

Supplement: Supplemental Information 30 [file peerj-09-10654-s030.zip › EmInf1_Clean_Data1.fq_fastqc/Images/sequence_length_distribution.png]

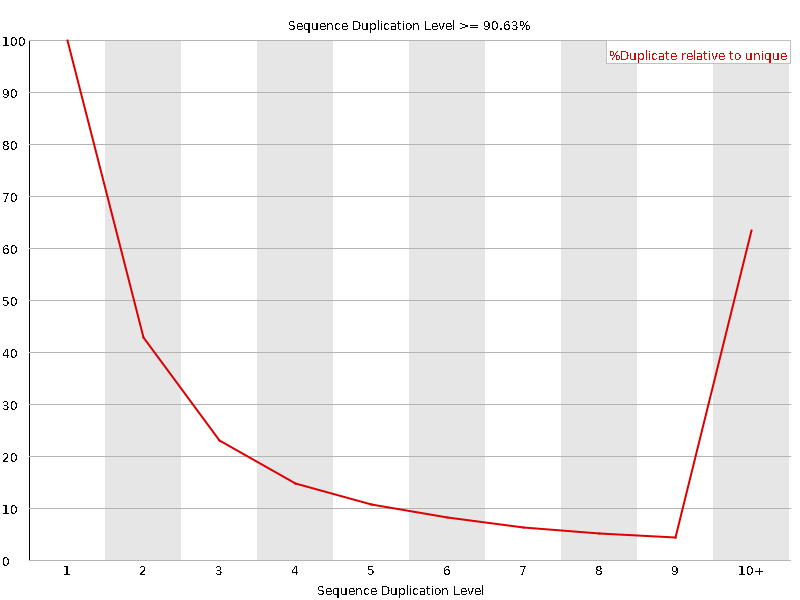

Supplement: Supplemental Information 30 [file peerj-09-10654-s030.zip › EmInf1_Clean_Data1.fq_fastqc/Images/duplication_levels.png]

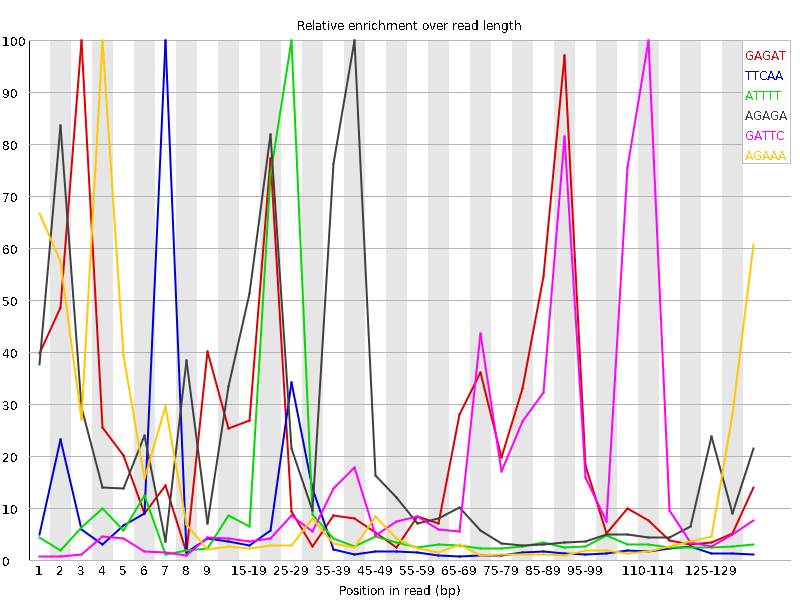

Supplement: Supplemental Information 30 [file peerj-09-10654-s030.zip › EmInf1_Clean_Data1.fq_fastqc/Images/kmer_profiles.png]

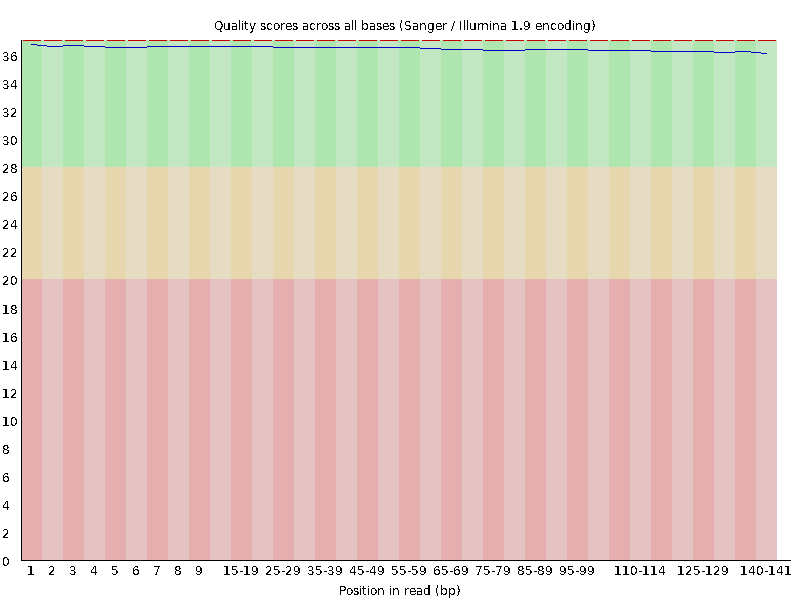

Supplement: Supplemental Information 31 [file peerj-09-10654-s031.zip › EmInf1_Clean_Data2.fq_fastqc/Images/per_base_quality.png]

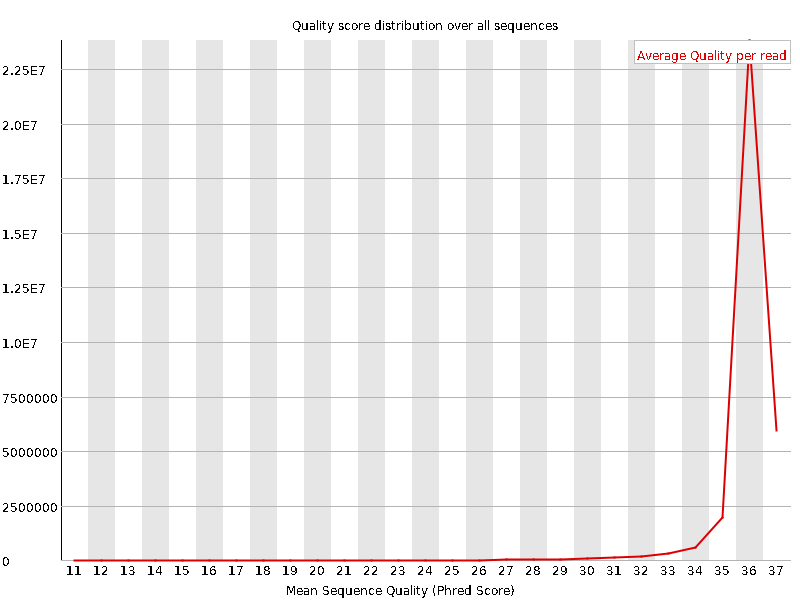

Supplement: Supplemental Information 31 [file peerj-09-10654-s031.zip › EmInf1_Clean_Data2.fq_fastqc/Images/per_sequence_quality.png]

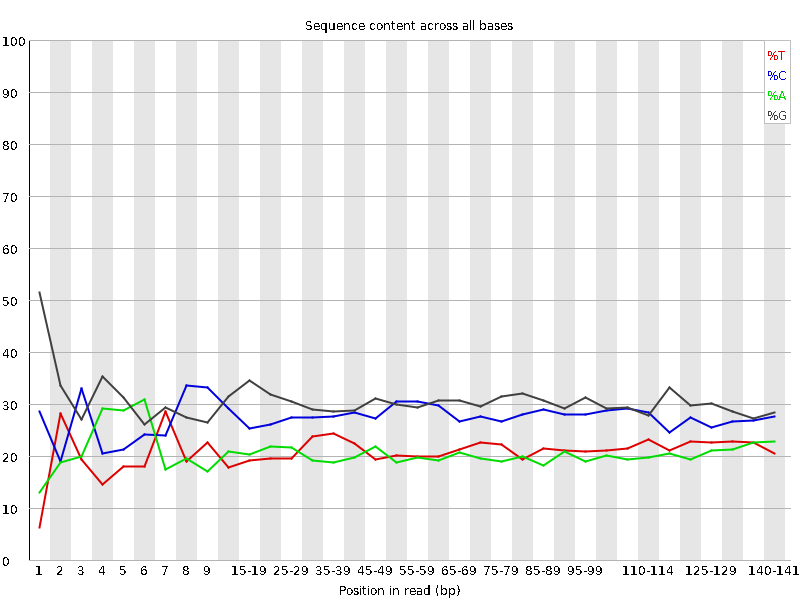

Supplement: Supplemental Information 31 [file peerj-09-10654-s031.zip › EmInf1_Clean_Data2.fq_fastqc/Images/per_base_sequence_content.png]

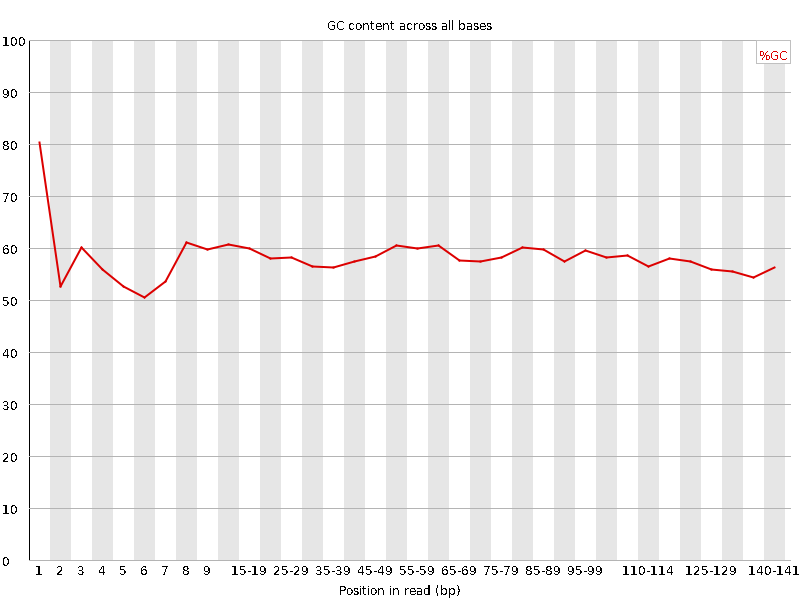

Supplement: Supplemental Information 31 [file peerj-09-10654-s031.zip › EmInf1_Clean_Data2.fq_fastqc/Images/per_base_gc_content.png]

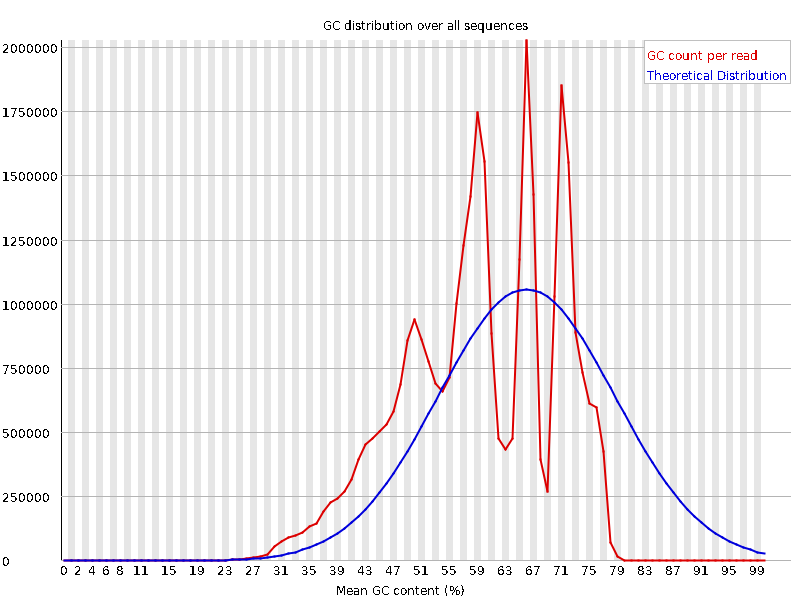

Supplement: Supplemental Information 31 [file peerj-09-10654-s031.zip › EmInf1_Clean_Data2.fq_fastqc/Images/per_sequence_gc_content.png]

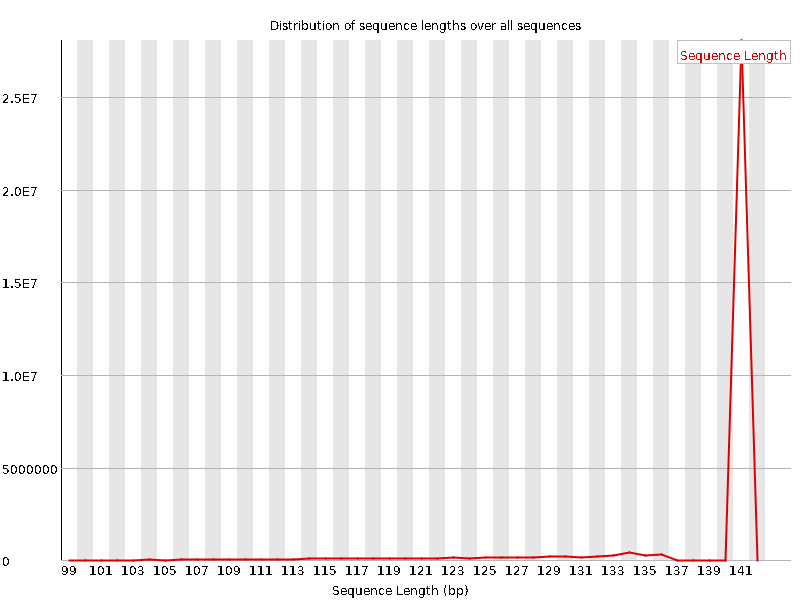

Supplement: Supplemental Information 31 [file peerj-09-10654-s031.zip › EmInf1_Clean_Data2.fq_fastqc/Images/sequence_length_distribution.png]

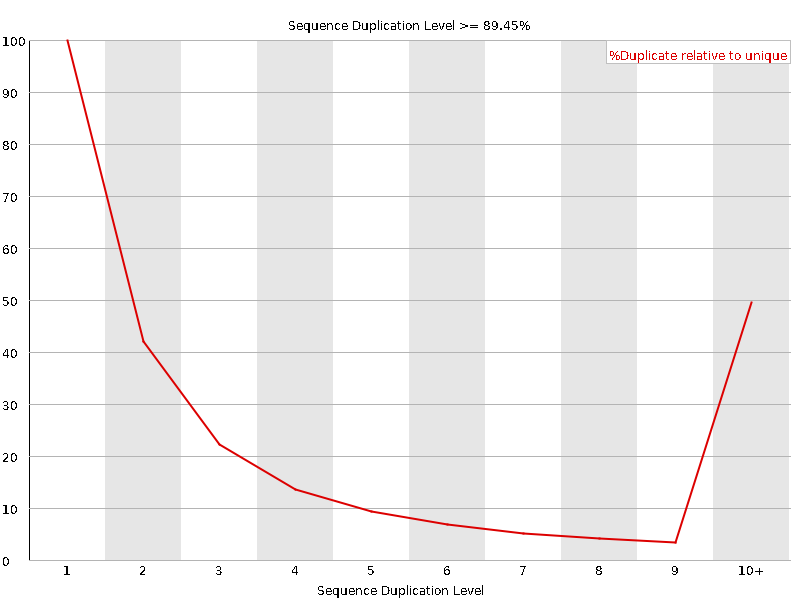

Supplement: Supplemental Information 31 [file peerj-09-10654-s031.zip › EmInf1_Clean_Data2.fq_fastqc/Images/duplication_levels.png]

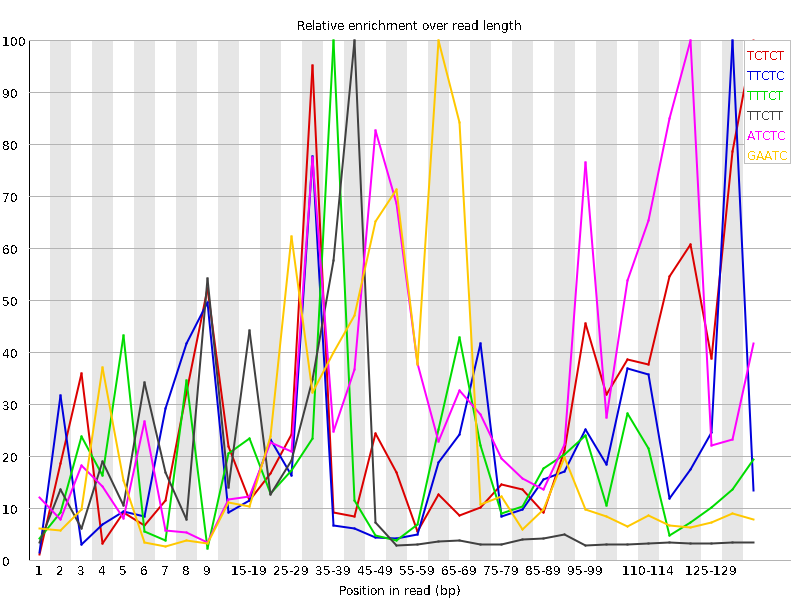

Supplement: Supplemental Information 31 [file peerj-09-10654-s031.zip › EmInf1_Clean_Data2.fq_fastqc/Images/kmer_profiles.png]

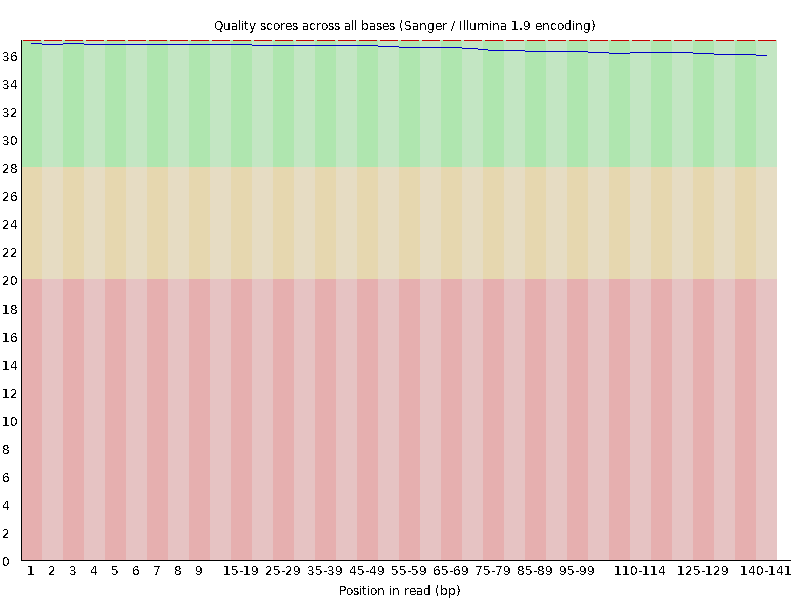

Supplement: Supplemental Information 32 [file peerj-09-10654-s032.zip › EmInf2_Clean_Data1.fq_fastqc/Images/per_base_quality.png]

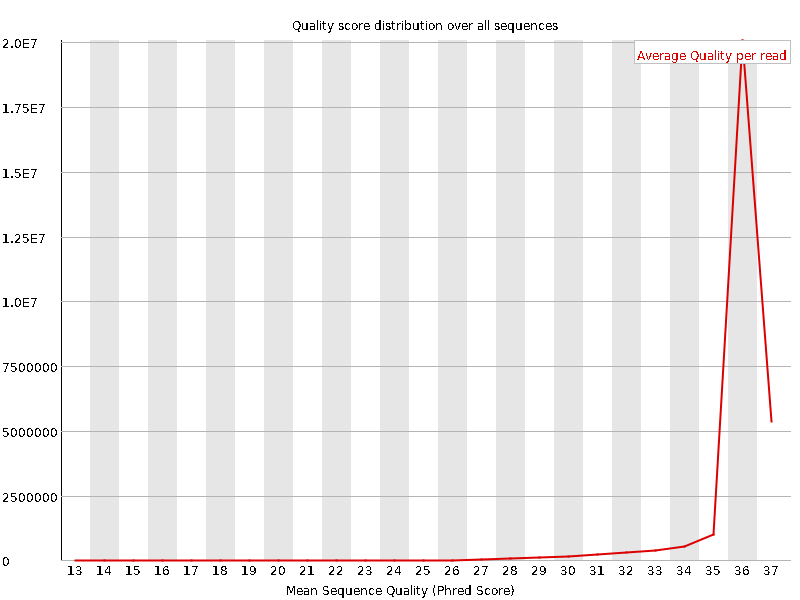

Supplement: Supplemental Information 32 [file peerj-09-10654-s032.zip › EmInf2_Clean_Data1.fq_fastqc/Images/per_sequence_quality.png]

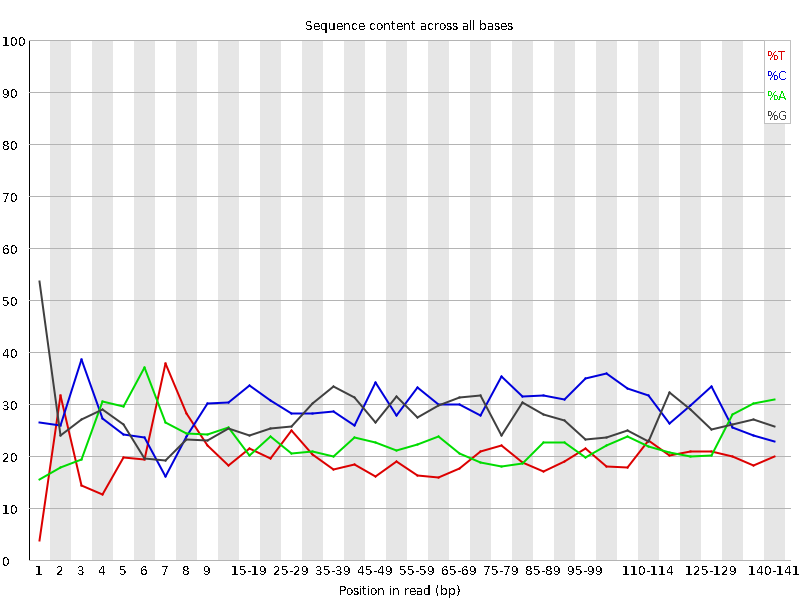

Supplement: Supplemental Information 32 [file peerj-09-10654-s032.zip › EmInf2_Clean_Data1.fq_fastqc/Images/per_base_sequence_content.png]

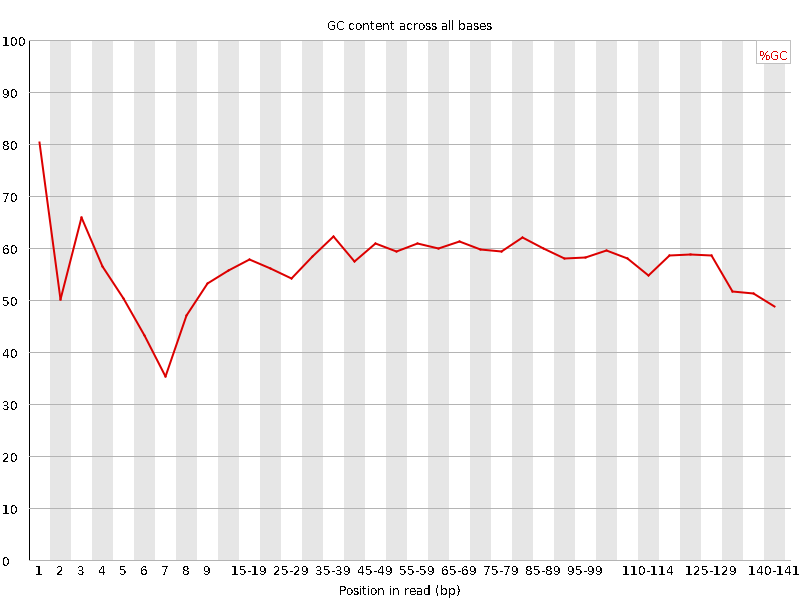

Supplement: Supplemental Information 32 [file peerj-09-10654-s032.zip › EmInf2_Clean_Data1.fq_fastqc/Images/per_base_gc_content.png]

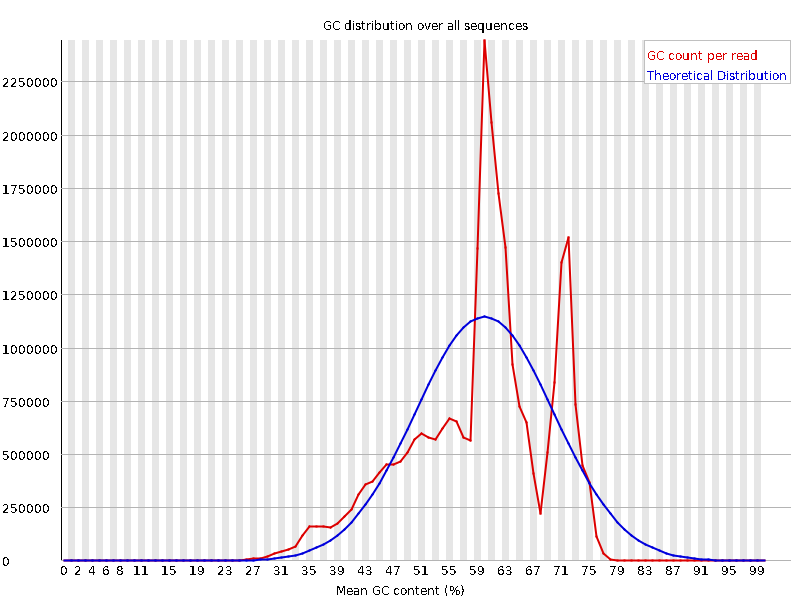

Supplement: Supplemental Information 32 [file peerj-09-10654-s032.zip › EmInf2_Clean_Data1.fq_fastqc/Images/per_sequence_gc_content.png]

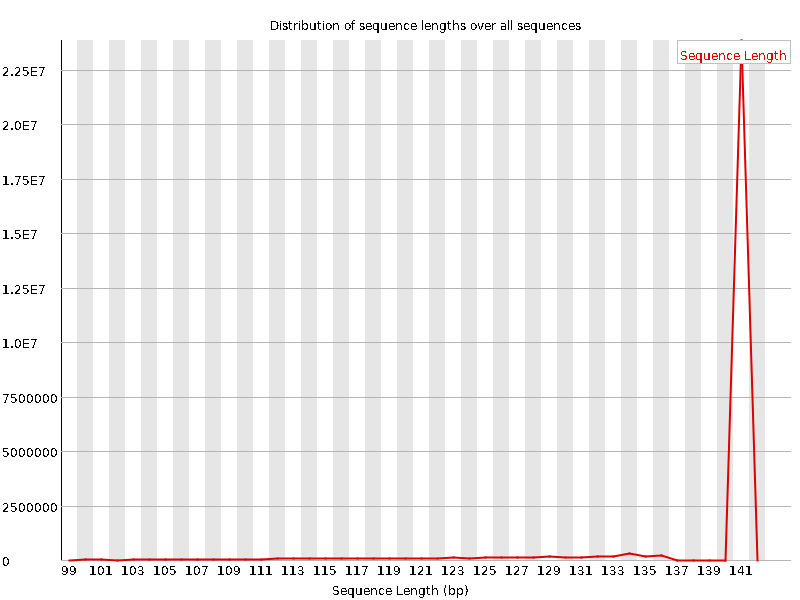

Supplement: Supplemental Information 32 [file peerj-09-10654-s032.zip › EmInf2_Clean_Data1.fq_fastqc/Images/sequence_length_distribution.png]

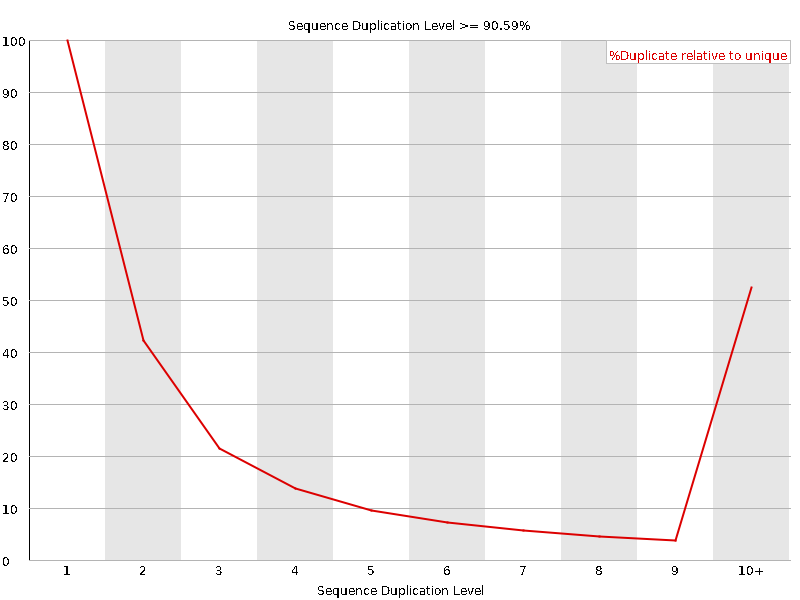

Supplement: Supplemental Information 32 [file peerj-09-10654-s032.zip › EmInf2_Clean_Data1.fq_fastqc/Images/duplication_levels.png]

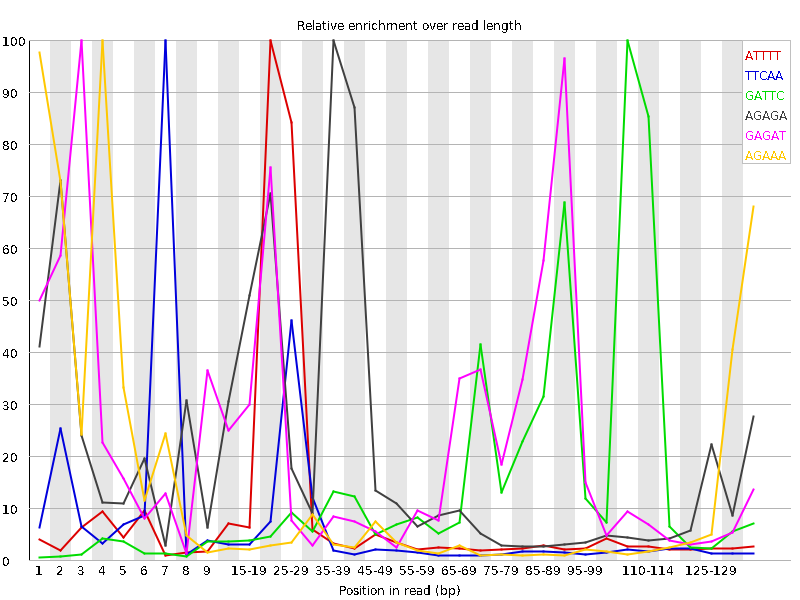

Supplement: Supplemental Information 32 [file peerj-09-10654-s032.zip › EmInf2_Clean_Data1.fq_fastqc/Images/kmer_profiles.png]

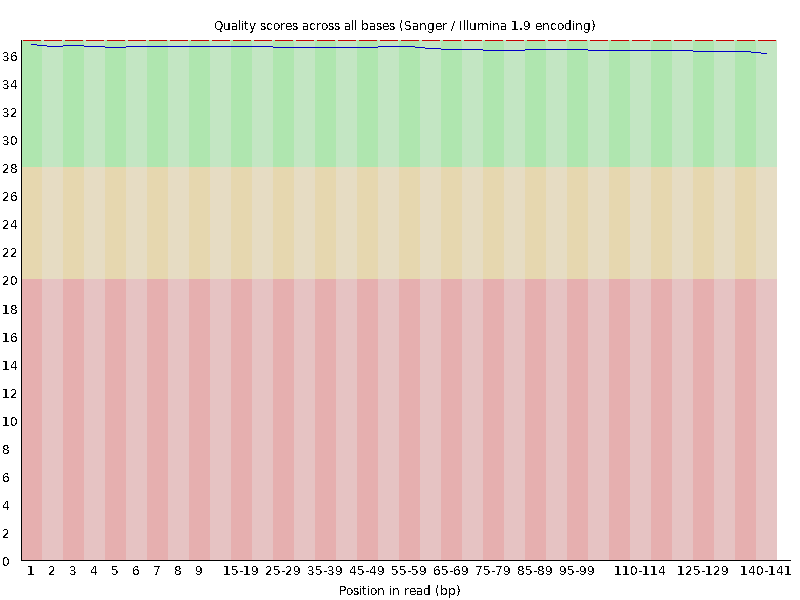

Supplement: Supplemental Information 33 [file peerj-09-10654-s033.zip › EmInf2_Clean_Data2.fq_fastqc/Images/per_base_quality.png]

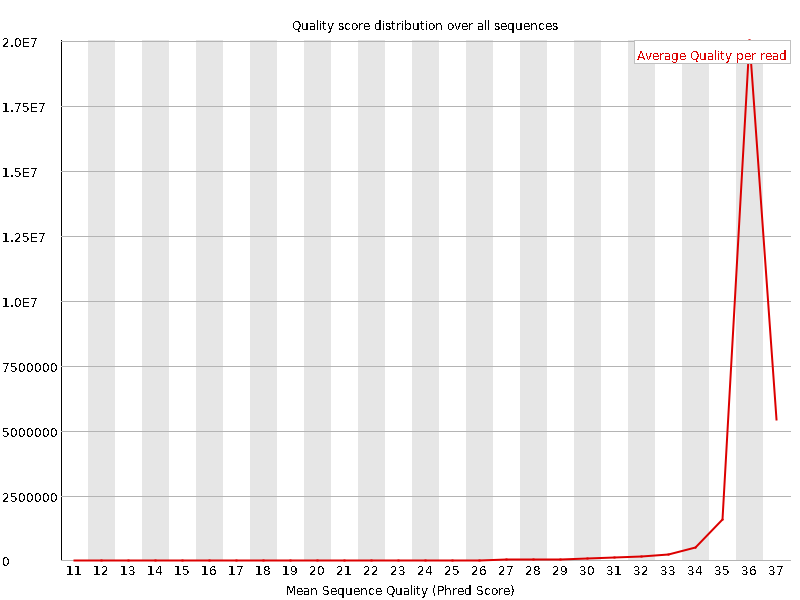

Supplement: Supplemental Information 33 [file peerj-09-10654-s033.zip › EmInf2_Clean_Data2.fq_fastqc/Images/per_sequence_quality.png]

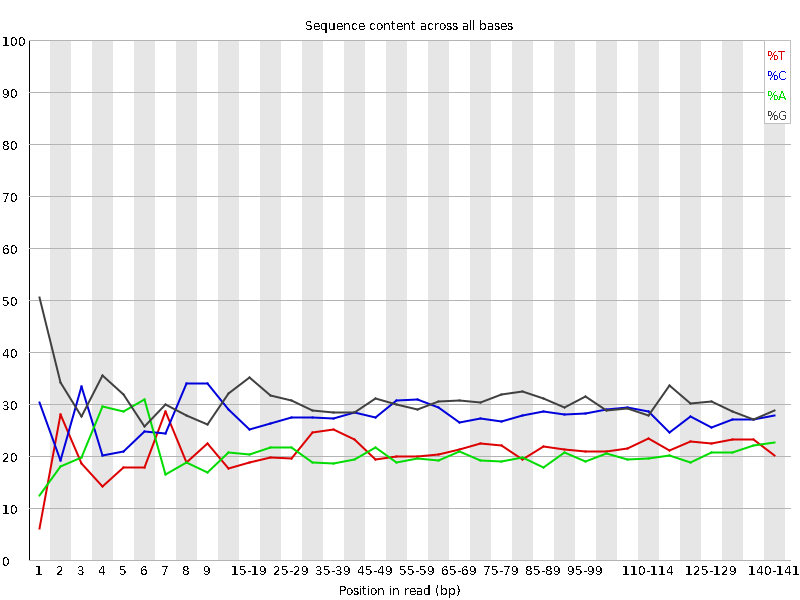

Supplement: Supplemental Information 33 [file peerj-09-10654-s033.zip › EmInf2_Clean_Data2.fq_fastqc/Images/per_base_sequence_content.png]

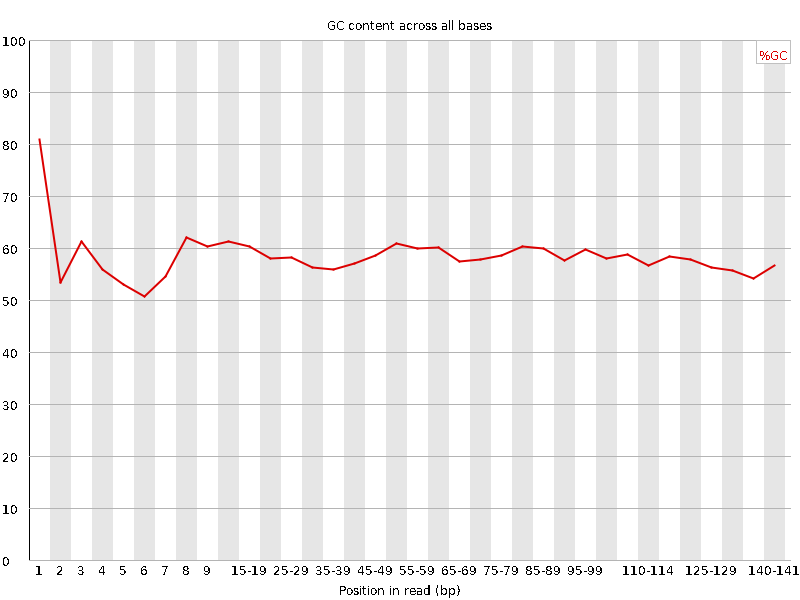

Supplement: Supplemental Information 33 [file peerj-09-10654-s033.zip › EmInf2_Clean_Data2.fq_fastqc/Images/per_base_gc_content.png]

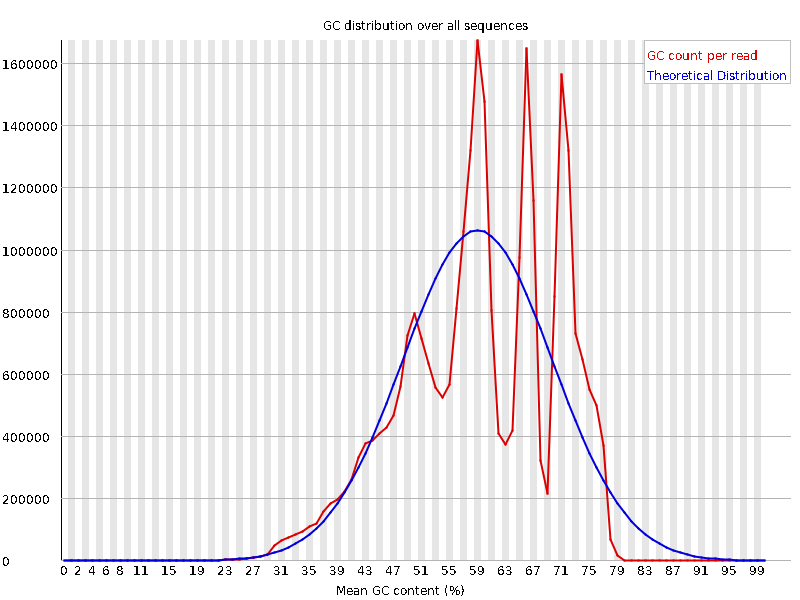

Supplement: Supplemental Information 33 [file peerj-09-10654-s033.zip › EmInf2_Clean_Data2.fq_fastqc/Images/per_sequence_gc_content.png]

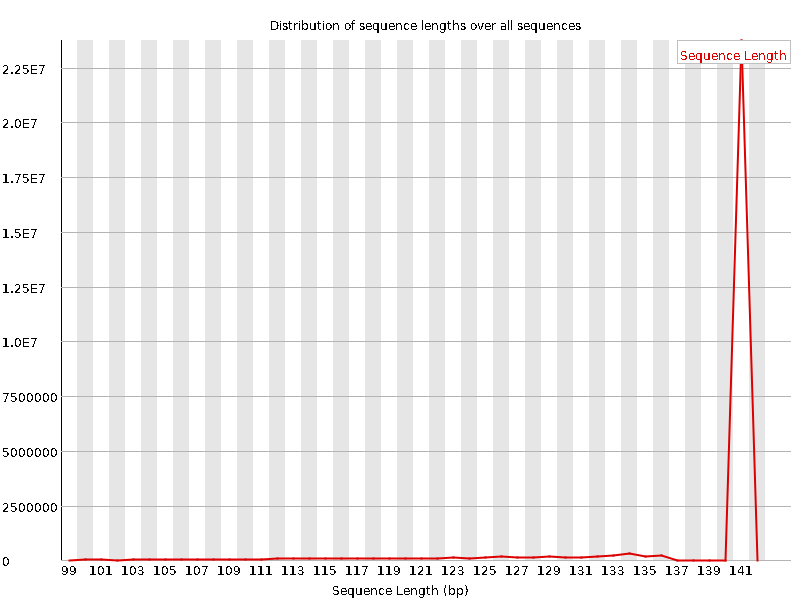

Supplement: Supplemental Information 33 [file peerj-09-10654-s033.zip › EmInf2_Clean_Data2.fq_fastqc/Images/sequence_length_distribution.png]

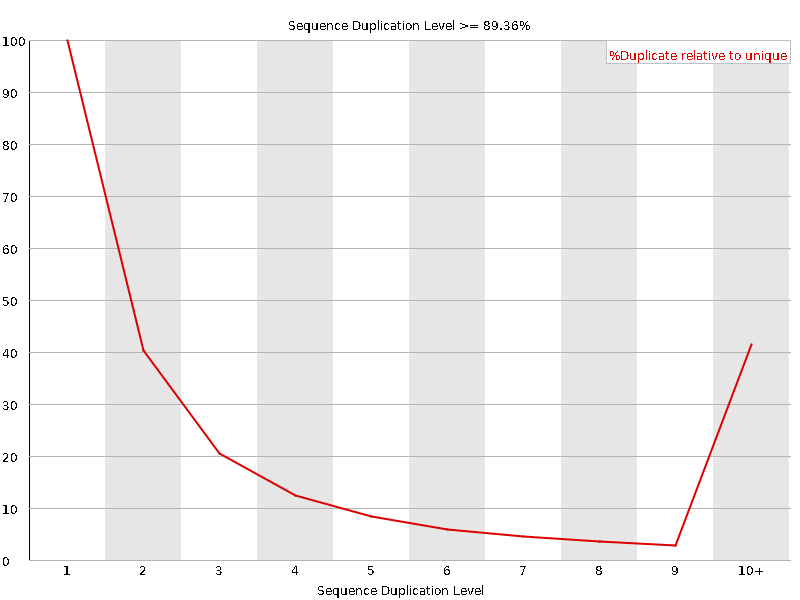

Supplement: Supplemental Information 33 [file peerj-09-10654-s033.zip › EmInf2_Clean_Data2.fq_fastqc/Images/duplication_levels.png]

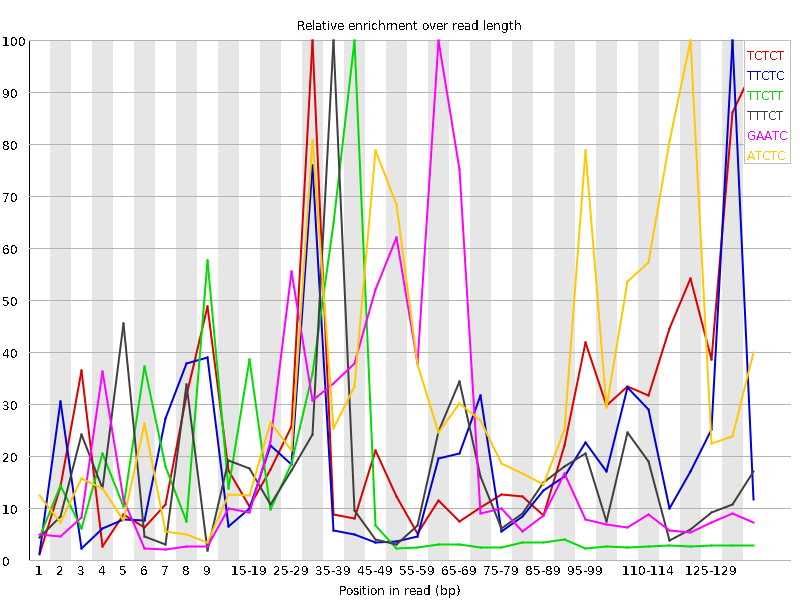

Supplement: Supplemental Information 33 [file peerj-09-10654-s033.zip › EmInf2_Clean_Data2.fq_fastqc/Images/kmer_profiles.png]

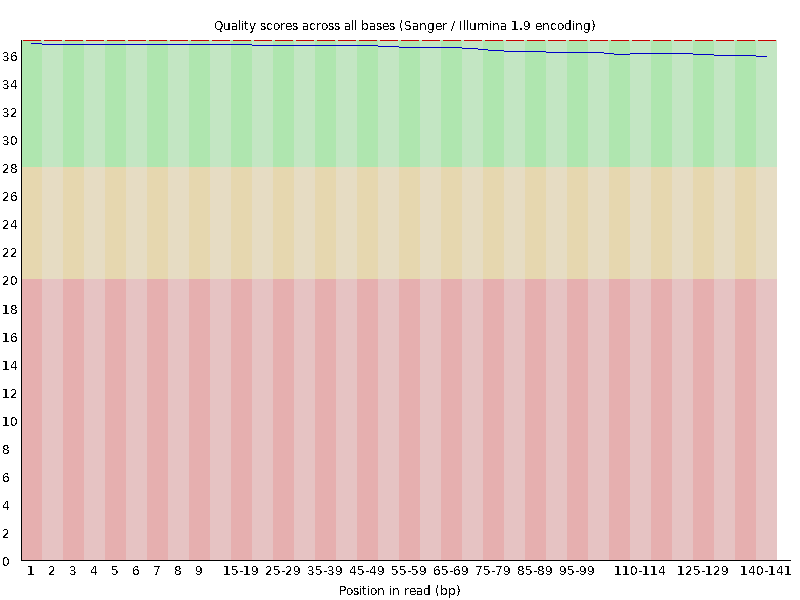

Supplement: Supplemental Information 34 [file peerj-09-10654-s034.zip › EmInf3_Clean_Data1.fq_fastqc/Images/per_base_quality.png]

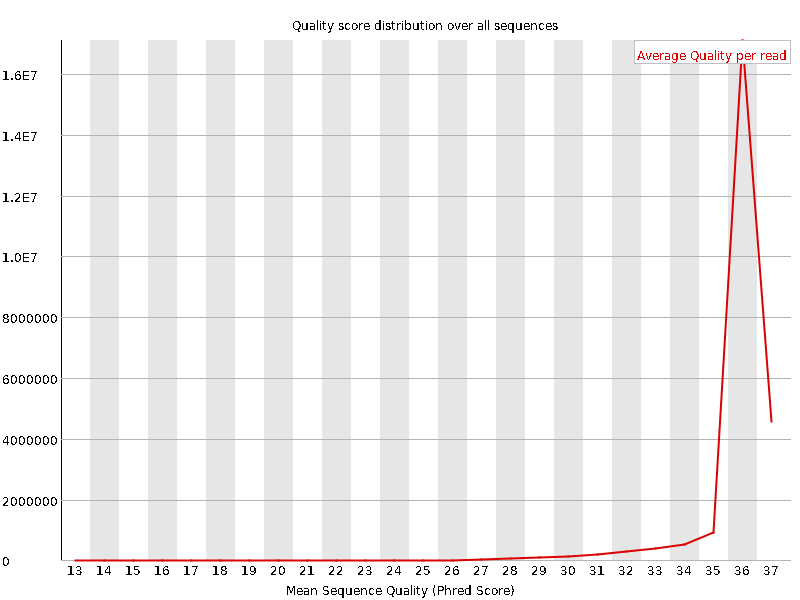

Supplement: Supplemental Information 34 [file peerj-09-10654-s034.zip › EmInf3_Clean_Data1.fq_fastqc/Images/per_sequence_quality.png]

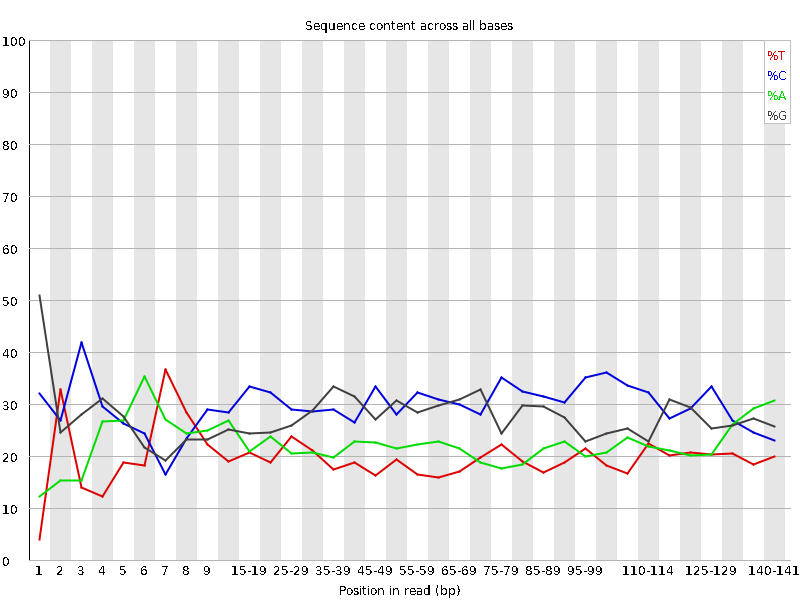

Supplement: Supplemental Information 34 [file peerj-09-10654-s034.zip › EmInf3_Clean_Data1.fq_fastqc/Images/per_base_sequence_content.png]

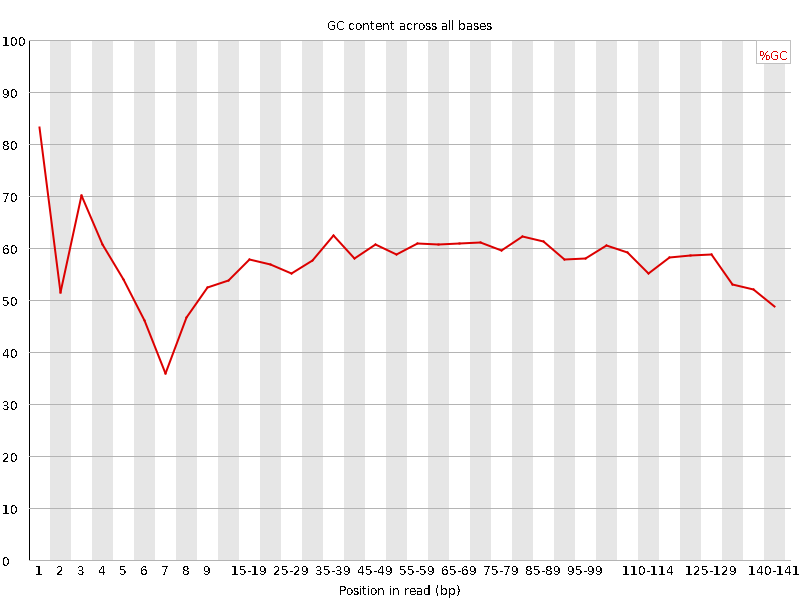

Supplement: Supplemental Information 34 [file peerj-09-10654-s034.zip › EmInf3_Clean_Data1.fq_fastqc/Images/per_base_gc_content.png]

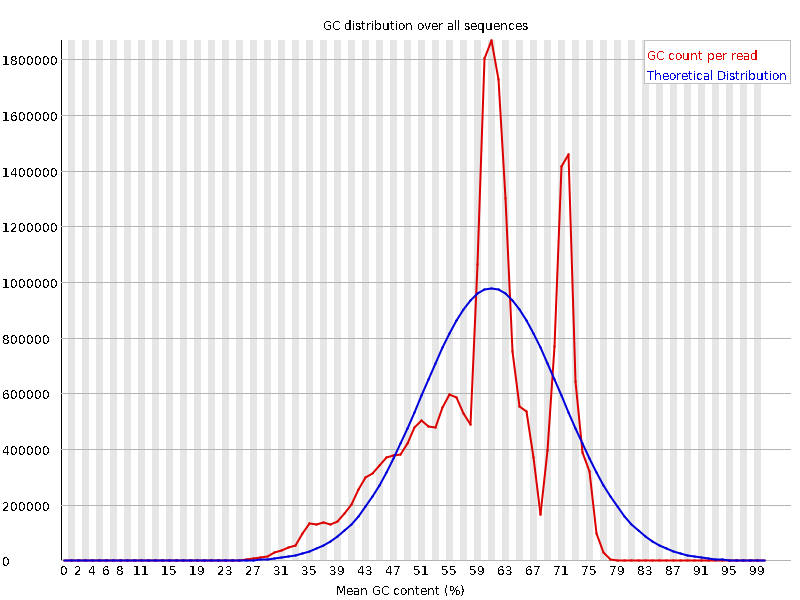

Supplement: Supplemental Information 34 [file peerj-09-10654-s034.zip › EmInf3_Clean_Data1.fq_fastqc/Images/per_sequence_gc_content.png]
