# Supplementary material for: Freshwater sponge hosts and their green algae symbionts: a tractable model to understand intracellular symbiosis
Source: PeerJ. 2021 Feb 11;9:e10654. doi: 10.7717/peerj.10654 (PMC7882143; doi:10.7717/peerj.10654)
Supplement: Supplemental Information 11 [file peerj-09-10654-s011.docx]

**Sequences of sponge-derived *Chlorella-like* algae**

18S

ATTCTTGGATTTATGAAAGACGAACTACTGCGAAAGCATTTGCCAAGGATGTTTTCATTAATCAAGAACGAAAGTTGGGGGCTCGAAGACGATTAGATACCGTCCTAGTCTCAACCATAAACGATGCCGACTAGGGATCGGCGGATGTTTCTTCGATGACTCCGCCGGCACCTTATGAGAAATCAAAGTTTTTGGGTTCCGGGGGGAGTATGGTCGCAAGGCTGAAACTTAAAGGAATTGACGGAAGGGCACCACCAGGCGTGGAGCCTGCGGCTTAATTTGACTCAACACGGGAAAACTTACCAGGTCCAGACATAGTGAGGATTGACAGATTGAGAGCTCTTTCTTGATTCTATGGGTGGTGGTGCATGGCCGTTCTTAGTTGGTGGGTTGCCTTGTCAGGTTGATTCCGGTAACGAACGAGACCTCAGCCTGCTAAATAGTCACGGTTGGCTCGCCAGCCGGCGGACTTCTTAGAGGGACTATTGGCGACTAGCCAATGGAAGCATGAGGCAATAACAGGTCTGTGATGCCCTTAGATGTTCTGGGCCGCACGCGCGCTACACTGATGCATTCAACGAGCCTAGCCTTGGCCGAGAGGCCCGGGTAATCTTTGAAACTGCATCGTGATGGGGATAGATTATTGCAATTATTAATCTTCAAC

5’ region of SSU ITS

TTAGGGTTCGATTCCGGAGAGGGAGCCTGAGAAACGGCTACCACATCCAAGGAAGGCAGCAGGCGCGCAAATTACCCAATCCTGACACAGGGAGGTAGTGACAATAAATAACAATACTGGGCCTTTTCAGGTCTGGTAATTGGAATGAGTACAATCTAAACCCCTTAACGAGGATCAATTGGAGGGCAAGTCTGGTGCCAGCAGCCGCGGTAATTCCAGCTCCAATAGCGTATATTTAAGTTGCTGCAGTTAAAAAGCTCGTAGTTGGATTTCGGGTGGGGCCTGCCGGTCCGCCGTTTCGGTGTGCACTGGCAGGGCCCACCTTGTTGCCGGGGACGGGCTCCTGGGCTTCACTGTCCGGGACTCGGAGTCGGCGCTGTTACTTTGAGTAAATTAGAGTGTTCAAAGCAGGCCTACGCTCTGAATACATTAGCATGGAATAACACGATAGGACTCTGGCCTATCCTGTTGGTCTGTAGGACCGGAGTAATGATTAAGAGGGACAGTCGGGGGCATTCGTATTTCATTGTCAGAGGTGAA

3’ region of SSU ITS

AGGTGAACCTGCGGAAGGATCATTGAATCGATCGAATCCACTCTGTGAACCAAACGTCCCCCCTTGGGTGCGGGCTTCGGTCTGCCCCAAGGCGTCGGTTCCCTGGCTGGGGTCTTCGGACCGCAGTTAGGTCCGGCGGGCGCGCCCTCTGGCGTGTCGGCCCTCGTGGCTGCCGCCAGTTGGGTTCGCTGGAAATTGTATCCAACTCAACCCACCCCAAACCACAACTTATACTGAAGCAATCGGTGAGTGCACTCTGGTGCCTCGCTCTAACCAAAGACAACTCTCAACAACGGATATCTTGGCTCCCGTATCGATGAAGAACGCAGCGAAATGCGATACGTAGTGTGAATTGCAGAATTCCGTGAACCATCGAATCTTTGAACGCAAATTGCGCCCAAGGCTTCGGCCGAGGGCATGTCTGCCTCAGCGTCGGCTTACCCCCTCGCTCCCCCTCTCCTTTGGAGTGGGTGAACGGATCTGGTTTTCCCGGCTACGTGCTTCTGCACGCCCGGGTTGACTGAAGTGTAGAGGCTTGAGCATGGACCCCGTTTGTAGGGCAATGGCTTGGTAGGTAGCTTAGCTACACCGCCTGCCGTGGTCCGAGGGGACTTTGCTGGCGGCCCAGCAGGAATTCGGGTGTTGGGTTTCCCACCCCGAAAGCTTCAAACCTTCGACCTGAGCTCAGGCAAGACTACCCGCTGAACTTAAGCATATCAATAAGCGGAGGAAAAGAAACTAACTAGGATGCCCTTAGTAACGGCGAGCGAACCGGGCAAAGCCCAACTTGAAAATCTCCAGCCTCCGGCTGGCGAATTGTAGTCTAGAGAAGTGCTCTCTGCCTCAGCCTGTTCCCAAGTCCCCTGGAAAGGGGCGTCAGAGAGGGTGAGAACCCCGTTGGGATCGGATCCTGAGGCTCC

23S

AGCCGGCGACTTAGAAAACGTGGCAAGGTTAAGGAAATGTATCCGGAGCCGAAGCGAAAGCAAGTCTGAATAGGGCGCGTAAGTCATTTTTTCTAGACCCGAACCCGGGTGATCTAACCATGACCAGGATGAAGCTTGGGTGACACCAAGTGAAGGTCCGAACCGACCGATGTTGAAAAATCGGCGGATGAGTTGTGGTTAGCGGTGAAATACCAGTCGAACTCGGAGCTAGCTGGTTCTCCCCGAAATGCGTTGAGGCGCAGCGGTTCATAAGGCTGTCTAGGGGTAAAGCACTGTTTCGGTGCGGGCTGCGAAAGCGGTACCAAATCGTGGCAAACTCTGAATACTAGATATGCTATTTATGGGCCAGTGAGACGGTGGGGGATAAGCTTCATCGTCGAGAGGGAAACAGCCCAGATCACTAGCTAAGGCCCCAAAATGATCGTTAAGTGACAAAGGAGGTGAGAATGCTGAAACAACCAGGAGGTTTGCTTAGAAGCAGCCACCCTTTAAAGAGTGCGTA

16S

TGCAGTCGTACGAATGAATTTTGGCTTGCCAAAATTTAATGAGTGGCGGACGGGTGAGTAACACGTAAGAACCTACCTTTTGGAGAGGGACAACCATTGGAAACGATGGCTAATACCTCGTAATGCTGATAAGTTAAATGATGAACAATCGCCAAAAGATGGGCTTGCGGCTGATTAGCTAGTTGGTGGGGTAAAGGCTTACCAAGGCGATGATCAGTAGCTGGTCTGAGAGGATGATCAGCCACACTGGGACTGAGACACGGCCCAACTCCTACGGGAGGCAGCAGTGAGGAATTTTCCGCAATGGGCGAAAGCCTGACGGAGCAATGCCGCGTGAAGGATGAATGCCTATGGGTTGTAAACTTCTTTTCTCAGAGAAGAATTCTTGACGGTATCTGAGGAATAAGCATCGGCTAACTCTGTGCCGCAGCCGCGGTAAGACAGAGGATGCAAGCGTTATCCGGAATGATTGGGCGTAAAGCGTCTGTAGTGGCTTAAAAAGKCTTAAGTCTGTCAAAGATCAGGCKTAACCCTGGGCCGGCAGGAGAAACTCTTATGCTAGAGTTTGGTAGGGGCAGAGGGAATTCCCGGTGGAGCGGTGAAATGCGTAGAGATCGGGAGGAACACCAAAGGCGAAAGCACTCTGCTGGGCCACAACTGACACTGAGAGACGAAAGCGAGGGGAGCAAAAGGGATTAGATACCCCTGTAGTCCTCGCCGTAAACGATGGATACTAGATGTTGGATAGGTTAAAGCATTCAGTATCGTAGCTAACGCGTGAAGTATCCCGCCTGGGGAGTATGCTCGCAAGAGGAAACTCAAAGGAATTACGGGGGCCCGCACAAGCGGTGGAGCATGTGGTTTAATTCGATGCAACGCGAAGAACCTTACCAGGACTTGACATGCCACTTTTTCCCTGAAAGGGGAAGTTCCCGAGTGGACACAGGTGGTGCATGGCTGTCGTCAGCTCGTGTCTGAGATGTTGGGTTAAGTCCCGCAACGAGCGCAACCCTTGTTTTGAATTGCCATTATTGGGAAATTCAAAAGACTGCCGGTGACAAGCCGGAGGAAGGTGAGGATGACGTCAAGTCAGCATGCCCCTTACGTCCTGGGCGACACACGTGCTACAATGGCCGGGACAAAGAGATGCAAACCCGCGAGGGCTAGCCAACCTCAAAAACCCGGTTTYARTTTGGATTGCAGGTTGCAAATCGCATGCMTGAARTTGGAATCGCTAGTAATCGCAGGTCAGCCATACTGCGGTGAATACGTTCCCGGGCCTTGTACACACCGCCCGTCACACCATGGGAGCTGGTATGCCCAAGTCGTTACCCCAACC

Chloroplast CS1_4

TCGGCTGATTTGTTGGTGGGGTAAAGGCTTACCAAGGCGATGATCAGTAGCTGGTCTGAGAGGATGATCAGCCACACTGGGACTGAGACACGGCCCAGACTCCTACGGGAGGCAGCAGTGAGGAATTTTCCGCAATGGGCGAAAGCCTGACGGAGCAATGCCGCGTGAAGGATGAAGGCCTATGGGTTGTAAACTTCTTTTCTCAGAGAAGAATTCTTGACGGTATCTGAGGAATAAGCATCGGCTAACTCTGTGCCAGCAGCCGCGGTAAGACAGAGGATGCAAGCGTTATCCGGAATGATTGGGCGTAAAGCGTCTGTAGGTGGCTTAAAAAGTCTTCTGTCAAAGATCAGGGCTTAACCCTGGGCCGGCAGGAGAAACTCTTAGGCTAGAGTTTGGTAGGGGCAGAGGGAATTCCCGGTGGAGCGGTGAAATGCGTAGAGATCGGGAGGAACACCAAAGGCGAAAGCACTCTGCTGGGCCACAACTGACACTGAGAGACGAAAGCGAGGGGAGCAAAAGGGATTAGATACCCCTGTAGTCCTCGCCGTAAACGATGGATACTAGATGTTGGATAGGTTAAAGCATTCAGTATCGTAGCTAACGCGTGAAGTATCCCGCCTGGGGAGTATGCTCGCAAGAGTGAAACTCAAAGGAATTGACGGGGGCCCGCACAAGCGGTGGAGCATGTGGTTTAATTCGATGCAACGCGAAGAACCTTACCAGGACTTGACATGCCACTTTTTCCCTGAAAGGGGAAGTTCCCGAGTGGACACAGGTGGTGCATGGCTGTCGTCAGCTCGTGTCTTGAGATGTTGGGTTAAGTCCCGCAACGAGCGCAACCCTTGTTTTGAATTGCCATTATTGGGAAATTCAAAAGACTGCCGGTGATAAGCCCGGAGGAAA

Nuclear NS1_4

CTTGTCTCAAAGATTAAGCCATGCATGTCTAAGTATAAACTGCTTTATACTGTGAAACTGCGAATGGCTCATTAAATCAGTTATAGTTTATTTGATGGTACCTACTACTCGGATACCCGTAGTAAATCTAGAGCTAATACGTGCGTAAATCCCGACTTCTGGAAGGGACGTATTTATTAGATAAAAGGCCGACCGGGCTCTGCCCGACTCGCGGTGAATCATGATAACTTCACGAATCGCATGGCCTCGTGCCGGCGATGTTTCATTCAAATTTCTGCCCTATCAACTTTCGATGGTAGGATAGAGGCCTACCATGGTGGTAACGGGTGACGGAGGATTAGGGTTCGATTCCGGAGAGGGAGCCTGAGAAACGGCTACCACATCCAAGGAAGGCAGCAGGCGCGCAAATTACCCAATCCTGACACAGGGAGGTAGTGACAATAAATAACAATACTGGGCCTTTTCAGGTCTGGTAATTGGAATGAGTACAATCTAAACCCCTTAACGAGGATCAATTGGAGGGCAAGTCTGGTG
