# Supplementary material for: Freshwater sponge hosts and their green algae symbionts: a tractable model to understand intracellular symbiosis
Source: PeerJ. 2021 Feb 11;9:e10654. doi: 10.7717/peerj.10654 (PMC7882143; doi:10.7717/peerj.10654)
Supplement: Supplemental Information 20 [file peerj-09-10654-s020.docx]

| **Sample (Em=*Ephydatia muelleri*, Apo=Aposymbiotic, Inf=Infected w/*Chlorella*)** | **Raw Data** | | **Valid Data, post-cleaning** | | **Valid Ratio (reads)** | **Q20%** | **Q30%** | **GC content%** |
| --- | --- | --- | --- | --- | --- | --- | --- | --- |
|  | **Reads** | **Bases** | **Reads** | **Bases** |  |  |  |  |
| EmApo1 | 60283236 | 9.04G | 58633932 | 8.80G | 97.26 | 99.97 | 98.85 | 58 |
| EmApo2 | 68523296 | 10.28G | 66301024 | 9.95G | 96.76 | 99.98 | 98.91 | 58 |
| EmApo3 | 67325704 | 10.10G | 65723824 | 9.86G | 97.62 | 99.98 | 99.01 | 57.50 |
| EmInf1 | 68823034 | 10.32G | 67022240 | 10.05G | 97.38 | 99.98 | 98.97 | 57.50 |
| EmInf2 | 58540960 | 8.78G | 57009984 | 8.55G | 97.38 | 99.98 | 98.93 | 57.50 |
| EmInf3 | 50279878 | 7.54G | 49084330 | 7.36G | 97.62 | 99.98 | 98.98 | 58 |

**Supplementary Table 1:** Quality control and read statistics for RNASeq analysis
