# Supplementary material for: Freshwater sponge hosts and their green algae symbionts: a tractable model to understand intracellular symbiosis
Source: PeerJ. 2021 Feb 11;9:e10654. doi: 10.7717/peerj.10654 (PMC7882143; doi:10.7717/peerj.10654)
Supplement: Supplemental Information 21 [file peerj-09-10654-s021.docx]

| **Sample** | **Valid reads** | **Mapped reads** | **Unique Mapped reads** | **Multi Mapped reads** | **PE Mapped reads** |
| --- | --- | --- | --- | --- | --- |
| EmApo1 | 58633932 | 34032135 (58.04%) | 20755364 (35.40%) | 13276771 (22.64%) | 25537776 (43.55%) |
| EmApo2 | 66301024 | 38263555 (57.71%) | 23776612 (35.86%) | 14486943 (21.85%) | 28420820 (42.87%) |
| EmApo3 | 65723824 | 38019603 (57.85%) | 23156155 (35.23%) | 14863448 (22.62%) | 28242584 (42.97%) |
| EmInf1 | 67022240 | 38916119 (58.06%) | 24038142 (35.87%) | 14877977 (22.20%) | 28924232 (43.16%) |
| EmInf2 | 57009984 | 32788797 (57.51%) | 19945968 (34.99%) | 12842829 (22.53%) | 24113818 (42.30%) |
| EmInf3 | 49084330 | 27733054 (56.50%) | 18170773 (37.02%) | 9562281 (19.48%) | 20723346 (42.22%) |

**Supplementary Table 2:** Statistics related to mapping of reads to reference genome.
