# Supplementary material for: Freshwater sponge hosts and their green algae symbionts: a tractable model to understand intracellular symbiosis
Source: PeerJ. 2021 Feb 11;9:e10654. doi: 10.7717/peerj.10654 (PMC7882143; doi:10.7717/peerj.10654)
Supplement: Supplemental Information 22 [file peerj-09-10654-s022.docx]

| **Sample** | **Exp gene** | **Min.** | **1st Qu.** | **Median** | **Mean** | **3rd Qu.** | **Max.** | **Sd.** | **Sum.** |
| --- | --- | --- | --- | --- | --- | --- | --- | --- | --- |
| EmApo1 | 31847 | 0.00 | 0.26 | 1.12 | 12.68 | 3.34 | 125118.01 | 746.18 | 403838.20 |
| EmApo2 | 31347 | 0.00 | 0.22 | 0.98 | 13.02 | 3.25 | 134930.49 | 810.76 | 408061.24 |
| EmApo3 | 32095 | 0.00 | 0.24 | 1.08 | 14.09 | 3.56 | 148405.68 | 878.32 | 452189.52 |
| EmInf1 | 32080 | 0.00 | 0.24 | 1.05 | 12.56 | 3.31 | 117241.97 | 707.45 | 402873.56 |
| EmInf2 | 31189 | 0.00 | 0.25 | 1.03 | 12.53 | 3.24 | 116363.40 | 705.07 | 390669.78 |
| EmInf3 | 30141 | 0.00 | 0.25 | 0.99 | 14.51 | 3.33 | 179180.41 | 1059.65 | 437297.48 |

| **Sample** | **Exp transcript** | **Min.** | **1st Qu.** | **Median** | **Mean** | **3rd Qu.** | **Max.** | **Sd.** | **Sum.** |
| --- | --- | --- | --- | --- | --- | --- | --- | --- | --- |
| EmApo1 | 53156 | 0.00 | 0.27 | 1.09 | 12.33 | 2.80 | 84772.13 | 584.81 | 655645.59 |
| EmApo2 | 52753 | 0.00 | 0.24 | 0.98 | 11.07 | 2.70 | 91019.25 | 529.70 | 584038.05 |
| EmApo3 | 53928 | 0.00 | 0.26 | 1.07 | 13.45 | 2.92 | 97543.26 | 705.36 | 725585.33 |
| EmInf1 | 53834 | 0.00 | 0.25 | 1.06 | 13.01 | 2.79 | 135428.30 | 734.67 | 700453.41 |
| EmInf2 | 52258 | 0.00 | 0.26 | 1.02 | 12.70 | 2.75 | 95322.56 | 712.51 | 663540.65 |
| EmInf3 | 50468 | 0.00 | 0.26 | 0.98 | 13.98 | 2.79 | 97963.54 | 738.30 | 705577.07 |

**Supplemental Table 3:** Summary statistics of raw reads mapped to genes and transcripts
