# Supplementary material for: Freshwater sponge hosts and their green algae symbionts: a tractable model to understand intracellular symbiosis
Source: PeerJ. 2021 Feb 11;9:e10654. doi: 10.7717/peerj.10654 (PMC7882143; doi:10.7717/peerj.10654)
Supplement: Supplemental Information 23 [file peerj-09-10654-s023.docx]

| **Sample** | **0-0.1 FI** | **0.1-0.3 FI** | **0.3-3.57 FI** | **3.57-15 FI** | **15-60 FI** | **>60 FI** |
| --- | --- | --- | --- | --- | --- | --- |
| EmApo1 | 4276 (13.43%) | 4419 (13.88%) | 15570 (48.89%) | 5825 (18.29%) | 1427 (4.48%) | 330 (1.04%) |
| EmApo2 | 4730 (15.09%) | 4483 (14.30%) | 14777 (47.14%) | 5589 (17.83%) | 1410 (4.50%) | 358 (1.14%) |
| EmApo3 | 4594 (14.31%) | 4391 (13.68%) | 15100 (47.05%) | 6006 (18.71%) | 1601 (4.99%) | 403 (1.26%) |
| EmInf1 | 4690 (14.62%) | 4408 (13.74%) | 15391 (47.98%) | 5735 (17.88%) | 1494 (4.66%) | 362 (1.13%) |
| EmInf2 | 4278 (13.72%) | 4338 (13.91%) | 15295 (49.04%) | 5556 (17.81%) | 1387 (4.45%) | 335 (1.07%) |
| EmInf3 | 4051 (13.44%) | 4282 (14.21%) | 14621 (48.51%) | 5458 (18.11%) | 1398 (4.64%) | 331 (1.10%) |

| **Sample** | **0-0.1 FI** | **0.1-0.3 FI** | **0.3-3.57 FI** | **3.57-15 FI** | **15-60 FI** | **>60 FI** |
| --- | --- | --- | --- | --- | --- | --- |
| EmApo1 | 7088 (13.33%) | 7039 (13.24%) | 28344 (53.32%) | 8413 (15.83%) | 1868 (3.51%) | 404 (0.76%) |
| EmApo2 | 7801 (14.79%) | 7184 (13.62%) | 27397 (51.93%) | 8106 (15.37%) | 1831 (3.47%) | 434 (0.82%) |
| EmApo3 | 7528 (13.96%) | 7125 (13.21%) | 27914 (51.76%) | 8803 (16.32%) | 2057 (3.81%) | 501 (0.93%) |
| EmInf1 | 7572 (14.07%) | 7282 (13.53%) | 28046 (52.10%) | 8587 (15.95%) | 1917 (3.56%) | 430 (0.80%) |
| EmInf2 | 7215 (13.81%) | 6914 (13.23%) | 27736 (53.08%) | 8214 (15.72%) | 1771 (3.39%) | 408 (0.78%) |
| EmInf3 | 6814 (13.50%) | 6977 (13.82%) | 26459 (52.43%) | 8029 (15.91%) | 1792 (3.55%) | 397 (0.79%) |

**Supplemental Table 4:** Statistics of FPKM Intervals of gene and transcript expression
