# Supplementary material for: Freshwater sponge hosts and their green algae symbionts: a tractable model to understand intracellular symbiosis
Source: PeerJ. 2021 Feb 11;9:e10654. doi: 10.7717/peerj.10654 (PMC7882143; doi:10.7717/peerj.10654)
Supplement: Supplemental Information 24 [file peerj-09-10654-s024.zip › EmApo1_Clean_Data1.fq_fastqc/fastqc_report.html]

EmApo1\_Clean\_Data1.fq.gz FastQC Report


FastQC Report

Tue 10 Sep 2019  
EmApo1\_Clean\_Data1.fq.gz

## Summary

- Basic Statistics
- Per base sequence quality
- Per sequence quality scores
- Per base sequence content
- Per base GC content
- Per sequence GC content
- Per base N content
- Sequence Length Distribution
- Sequence Duplication Levels
- Overrepresented sequences
- Kmer Content

## Basic Statistics

| Measure | Value |
| --- | --- |
| Filename | EmApo1\_Clean\_Data1.fq.gz |
| File type | Conventional base calls |
| Encoding | Sanger / Illumina 1.9 |
| Total Sequences | 29316966 |
| Filtered Sequences | 0 |
| Sequence length | 100-141 |
| %GC | 58 |

## Per base sequence quality

## Per sequence quality scores

## Per base sequence content

## Per base GC content

## Per sequence GC content

## Per base N content

## Sequence Length Distribution

## Sequence Duplication Levels

## Overrepresented sequences

| Sequence | Count | Percentage | Possible Source |
| --- | --- | --- | --- |
| GTCCCATTCAAGTCGTCTACAAGAGATCTTGCCCCGCGGATTGGCCAGCG | 1326142 | 4.52346262570281 | No Hit |
| GCGAGAAAATGAACCGCTCCCTCGGATTTTCAAGGGCCGTAGAGAACGCA | 871716 | 2.973418190681805 | No Hit |
| AGAAAATGAACCGCTCCCTCGGATTTTCAAGGGCCGTAGAGAACGCACCG | 723709 | 2.4685671771082998 | No Hit |
| GAGAAAATGAACCGCTCCCTCGGATTTTCAAGGGCCGTAGAGAACGCACC | 581247 | 1.9826301261870003 | No Hit |
| GCCACCTACAGCCAACAGTCTGAAGCGCAGTCGCGAACCCCGCGCACGGC | 485455 | 1.6558841730075342 | No Hit |
| GTCGTCTACAAGAGATCTTGCCCCGCGGATTGGCCAGCGTTTGATACGCG | 443236 | 1.5118754102999608 | No Hit |
| GCCGTTAGTCGCCTGCCGAATAGCCGCCGACCACGAGGGACGGCGACCAA | 415594 | 1.4175887095547335 | No Hit |
| AAGAGATCTTGCCCCGCGGATTGGCCAGCGTTTGATACGCGCGGTCACCG | 377331 | 1.2870738397690948 | No Hit |
| GTCGCCGTAACAGCACCGCCCGCAACCCACGTTGGCCAGCCCCGGTGAGA | 326235 | 1.1127856818471598 | No Hit |
| CTGCGCTGGCGGGTCGAAGAGACCCTCTCCTCGGTCGCGGGCGCGCTCCG | 326137 | 1.1124514044188611 | No Hit |
| GGCGAGAAAATGAACCGCTCCCTCGGATTTTCAAGGGCCGTAGAGAACGC | 305282 | 1.041315121080401 | No Hit |
| GAAAATGAACCGCTCCCTCGGATTTTCAAGGGCCGTAGAGAACGCACCGG | 289930 | 0.988949538639162 | No Hit |
| ATTCAAGTCGTCTACAAGAGATCTTGCCCCGCGGATTGGCCAGCGTTTGA | 271595 | 0.926408960599811 | No Hit |
| AGCGCAGTCGCGAACCCCGCGCACGGCGGAGGGATGCGCCGGCCTCGCAC | 255739 | 0.8723242370987502 | No Hit |
| CTCTCCTCGGTCGCGGGCGCGCTCCGAACGACGCGGCTATACGTCCCTAA | 204721 | 0.6983021367217876 | No Hit |
| GAGATCTTGCCCCGCGGATTGGCCAGCGTTTGATACGCGCGGTCACCGAA | 199652 | 0.6810118072927465 | No Hit |
| CGGGCGAGAAAATGAACCGCTCCCTCGGATTTTCAAGGGCCGTAGAGAAC | 195720 | 0.6675997782308033 | No Hit |
| GGCCGTTAGTCGCCTGCCGAATAGCCGCCGACCACGAGGGACGGCGACCA | 195617 | 0.667248445831673 | No Hit |
| AGAGATCTTGCCCCGCGGATTGGCCAGCGTTTGATACGCGCGGTCACCGA | 189570 | 0.6466221641079777 | No Hit |
| CTCCTCGGTCGCGGGCGCGCTCCGAACGACGCGGCTATACGTCCCTAACT | 187641 | 0.6400423563611596 | No Hit |
| GCGCTGGCGGGTCGAAGAGACCCTCTCCTCGGTCGCGGGCGCGCTCCGAA | 183392 | 0.6255490421484952 | No Hit |
| GATGAAGCCACCTACAGCCAACAGTCTGAAGCGCAGTCGCGAACCCCGCG | 158921 | 0.5420786039046469 | No Hit |
| GTCTACAAGAGATCTTGCCCCGCGGATTGGCCAGCGTTTGATACGCGCGG | 150102 | 0.5119970463519314 | No Hit |
| AGAAATTTGAATGCACCATCGCCGGCACGAGGCCATGCGATTCGAGCAGT | 133128 | 0.45409883137293267 | No Hit |
| GCAGAAATTTGAATGCACCATCGCCGGCACGAGGCCATGCGATTCGAGCA | 129743 | 0.4425526161199627 | No Hit |
| CCGTAACAGCACCGCCCGCAACCCACGTTGGCCAGCCCCGGTGAGAAATG | 115951 | 0.3955081845781723 | No Hit |
| GTCGGCCGTTAGTCGCCTGCCGAATAGCCGCCGACCACGAGGGACGGCGA | 113474 | 0.3870591520282146 | No Hit |
| CAAGTCGTCTACAAGAGATCTTGCCCCGCGGATTGGCCAGCGTTTGATAC | 111288 | 0.37960271878065416 | No Hit |
| AAAATGAACCGCTCCCTCGGATTTTCAAGGGCCGTAGAGAACGCACCGGA | 105515 | 0.35991104945852853 | No Hit |
| GCCCGCAACCCACGTTGGCCAGCCCCGGTGAGAAATGCGGAAGCGGCGGT | 104641 | 0.35692984055717086 | No Hit |
| GGGCGAGAAAATGAACCGCTCCCTCGGATTTTCAAGGGCCGTAGAGAACG | 103698 | 0.3537132730583376 | No Hit |
| AGATCTTGCCCCGCGGATTGGCCAGCGTTTGATACGCGCGGTCACCGAAG | 102608 | 0.3499952894170563 | No Hit |
| GAAATTTGAATGCACCATCGCCGGCACGAGGCCATGCGATTCGAGCAGTT | 100032 | 0.3412085684446337 | No Hit |
| CGAGAAAATGAACCGCTCCCTCGGATTTTCAAGGGCCGTAGAGAACGCAC | 99781 | 0.3403524089088891 | No Hit |
| GTCAGATGAAGCCACCTACAGCCAACAGTCTGAAGCGCAGTCGCGAACCC | 99328 | 0.3388072285515493 | No Hit |
| CTCGTCCCATTCAAGTCGTCTACAAGAGATCTTGCCCCGCGGATTGGCCA | 92439 | 0.31530888973981824 | No Hit |
| GAAGCCACCTACAGCCAACAGTCTGAAGCGCAGTCGCGAACCCCGCGCAC | 90609 | 0.3090667704154652 | No Hit |
| GTTAGTCGCCTGCCGAATAGCCGCCGACCACGAGGGACGGCGACCAAGCT | 87905 | 0.29984344218975456 | No Hit |
| GCGCAGTCGCGAACCCCGCGCACGGCGGAGGGATGCGCCGGCCTCGCACT | 87580 | 0.2987348690857028 | No Hit |
| GATCTTGCCCCGCGGATTGGCCAGCGTTTGATACGCGCGGTCACCGAAGG | 85944 | 0.2931544826296146 | No Hit |
| GCCTGCGCTGGCGGGTCGAAGAGACCCTCTCCTCGGTCGCGGGCGCGCTC | 85932 | 0.29311355069961875 | No Hit |
| GCGGGAGCTCCGGCCACGAAGGCCTGCGCTGGCGGGTCGAAGAGACCCTC | 85759 | 0.2925234487088466 | No Hit |
| GCCGTAACAGCACCGCCCGCAACCCACGTTGGCCAGCCCCGGTGAGAAAT | 85488 | 0.29159906928977575 | No Hit |
| CAAGAGATCTTGCCCCGCGGATTGGCCAGCGTTTGATACGCGCGGTCACC | 83679 | 0.28542858084291534 | No Hit |
| CCGCAACCCACGTTGGCCAGCCCCGGTGAGAAATGCGGAAGCGGCGGTCG | 81569 | 0.27823138315199464 | No Hit |
| CTTATATTGGTCGGGCTAGGAGCTGAGTCTACTCACAGGCACTATCCCAT | 78231 | 0.2668454846248414 | No Hit |
| CGCGGATTGGCCAGCGTTTGATACGCGCGGTCACCGAAGGCCGCCTACGG | 77712 | 0.26507517865252495 | No Hit |
| CCGCCCGCAACCCACGTTGGCCAGCCCCGGTGAGAAATGCGGAAGCGGCG | 77305 | 0.26368690402683553 | No Hit |
| CCTGACTCTCCAAAGACACCTAATATCTAGGCAGGCGGTCGGCCGCGTAC | 76460 | 0.26080461395630095 | No Hit |
| CATTCAAGTCGTCTACAAGAGATCTTGCCCCGCGGATTGGCCAGCGTTTG | 76141 | 0.25971650681724706 | No Hit |
| TTCAAGTCGTCTACAAGAGATCTTGCCCCGCGGATTGGCCAGCGTTTGAT | 73712 | 0.2514312019872725 | No Hit |
| CCCGCAACCCACGTTGGCCAGCCCCGGTGAGAAATGCGGAAGCGGCGGTC | 71837 | 0.24503558792543537 | No Hit |
| CGTTAGTCGCCTGCCGAATAGCCGCCGACCACGAGGGACGGCGACCAAGC | 68609 | 0.23402489875657667 | No Hit |
| GGCGGGTCGAAGAGACCCTCTCCTCGGTCGCGGGCGCGCTCCGAACGACG | 64537 | 0.22013533051134965 | No Hit |
| AAATGAACCGCTCCCTCGGATTTTCAAGGGCCGTAGAGAACGCACCGGAC | 63825 | 0.21770670266493472 | No Hit |
| CCCATTCAAGTCGTCTACAAGAGATCTTGCCCCGCGGATTGGCCAGCGTT | 63547 | 0.21675844628669966 | No Hit |
| GCCAACAGTCTGAAGCGCAGTCGCGAACCCCGCGCACGGCGGAGGGATGC | 61220 | 0.20882106286168903 | No Hit |
| CCTGCGCTGGCGGGTCGAAGAGACCCTCTCCTCGGTCGCGGGCGCGCTCC | 60907 | 0.207753421687633 | No Hit |
| CGAGATGGCGCCCTCCACCGGAACGCGGGAGCTCCGGCCACGAAGGCCTG | 60376 | 0.20594218378532075 | No Hit |
| CACCCGGTCGCCGTAACAGCACCGCCCGCAACCCACGTTGGCCAGCCCCG | 59130 | 0.2016920850540946 | No Hit |
| GCCACGAAGGCCTGCGCTGGCGGGTCGAAGAGACCCTCTCCTCGGTCGCG | 58519 | 0.19960796761847732 | No Hit |
| GGCCTGCGCTGGCGGGTCGAAGAGACCCTCTCCTCGGTCGCGGGCGCGCT | 57677 | 0.19673591053044165 | No Hit |
| CACCTACAGCCAACAGTCTGAAGCGCAGTCGCGAACCCCGCGCACGGCGG | 55973 | 0.1909235764710441 | No Hit |
| GCTTACAACACCTCGTCCCATTCAAGTCGTCTACAAGAGATCTTGCCCCG | 55764 | 0.19021067869028466 | No Hit |
| CTGCTTACAACACCTCGTCCCATTCAAGTCGTCTACAAGAGATCTTGCCC | 55132 | 0.18805493037717477 | No Hit |
| CTCGGTCGCGGGCGCGCTCCGAACGACGCGGCTATACGTCCCTAACTTCG | 54086 | 0.18448703047921125 | No Hit |
| CCCGGTCGCCGTAACAGCACCGCCCGCAACCCACGTTGGCCAGCCCCGGT | 52952 | 0.1806189630946122 | No Hit |
| GGCAGAAATTTGAATGCACCATCGCCGGCACGAGGCCATGCGATTCGAGC | 52796 | 0.18008684800466734 | No Hit |
| CTCCAAAGACACCTAATATCTAGGCAGGCGGTCGGCCGCGTACGGGGTTC | 49958 | 0.1704064465606707 | No Hit |
| CTACAAGAGATCTTGCCCCGCGGATTGGCCAGCGTTTGATACGCGCGGTC | 49232 | 0.16793006479592737 | No Hit |
| TGACTCTCCAAAGACACCTAATATCTAGGCAGGCGGTCGGCCGCGTACGG | 48117 | 0.16412680630048826 | No Hit |
| CCGGTCGCCGTAACAGCACCGCCCGCAACCCACGTTGGCCAGCCCCGGTG | 47729 | 0.1628033405639588 | No Hit |
| CGCCGTAACAGCACCGCCCGCAACCCACGTTGGCCAGCCCCGGTGAGAAA | 46837 | 0.15976073376760747 | No Hit |
| AGATGAAGCCACCTACAGCCAACAGTCTGAAGCGCAGTCGCGAACCCCGC | 45419 | 0.15492394403977547 | No Hit |
| CTCCACCGGAACGCGGGAGCTCCGGCCACGAAGGCCTGCGCTGGCGGGTC | 45010 | 0.1535288474257534 | No Hit |
| CACCGGAACGCGGGAGCTCCGGCCACGAAGGCCTGCGCTGGCGGGTCGAA | 44499 | 0.1517858294067674 | No Hit |
| GCTCCCTCGGATTTTCAAGGGCCGTAGAGAACGCACCGGACGCCACCAGA | 44320 | 0.15117526145099736 | No Hit |
| AAGCGCAGTCGCGAACCCCGCGCACGGCGGAGGGATGCGCCGGCCTCGCA | 43670 | 0.14895811524289382 | No Hit |
| GTCGGGCTAGGAGCTGAGTCTACTCACAGGCACTATCCCATTACCGCCTG | 43385 | 0.14798598190549458 | No Hit |
| ATCTTGCCCCGCGGATTGGCCAGCGTTTGATACGCGCGGTCACCGAAGGC | 42942 | 0.1464749114898179 | No Hit |
| GGTCGGGCTAGGAGCTGAGTCTACTCACAGGCACTATCCCATTACCGCCT | 41248 | 0.14069668737208346 | No Hit |
| CTACTGCTTACAACACCTCGTCCCATTCAAGTCGTCTACAAGAGATCTTG | 39635 | 0.13519475378182041 | No Hit |
| CCTCGGTCGCGGGCGCGCTCCGAACGACGCGGCTATACGTCCCTAACTTC | 38077 | 0.12988042487070456 | No Hit |
| CTACAGCCAACAGTCTGAAGCGCAGTCGCGAACCCCGCGCACGGCGGAGG | 37822 | 0.12901062135829472 | No Hit |
| CCGAGATGGCGCCCTCCACCGGAACGCGGGAGCTCCGGCCACGAAGGCCT | 37005 | 0.1262238391244169 | No Hit |
| ACCGCCCGCAACCCACGTTGGCCAGCCCCGGTGAGAAATGCGGAAGCGGC | 35685 | 0.12172132682488358 | No Hit |
| GCGCATATGTAGCCCAAAACATTAGGATCATAAGGACCTGACGTCATCCT | 35504 | 0.1211039368807809 | No Hit |
| CTCGGATTTTCAAGGGCCGTAGAGAACGCACCGGACGCCACCAGAAGCGT | 34682 | 0.11830009967607152 | No Hit |
| CTCTACTGCTTACAACACCTCGTCCCATTCAAGTCGTCTACAAGAGATCT | 34241 | 0.11679585124872743 | No Hit |
| CCATTCAAGTCGTCTACAAGAGATCTTGCCCCGCGGATTGGCCAGCGTTT | 34136 | 0.11643769686126455 | No Hit |
| ACAAGAGATCTTGCCCCGCGGATTGGCCAGCGTTTGATACGCGCGGTCAC | 33262 | 0.1134564879599069 | No Hit |
| CAACAGTCTGAAGCGCAGTCGCGAACCCCGCGCACGGCGGAGGGATGCGC | 32814 | 0.11192836257339864 | No Hit |
| ATTCAACCTGGATACAGCCGGACTCCTCCGGCGAACCCATATTGATGCCA | 32810 | 0.11191471859673338 | No Hit |
| CTCGTCCCGGTTCGGGAATATTAACCCGATTCCCTTTCGATGGTGGGTGC | 32638 | 0.11132802760012751 | No Hit |
| GTCGCCTGCCGAATAGCCGCCGACCACGAGGGACGGCGACCAAGCTGCGG | 32252 | 0.11001138385193066 | No Hit |
| GTCTACTTATATTGGTCGGGCTAGGAGCTGAGTCTACTCACAGGCACTAT | 32182 | 0.10977261426028874 | No Hit |
| GGGAAATGTGTCGTTGCGTTCTAGCGTGGATTCTGACTTAGAGGCGTTCA | 31980 | 0.10908359343869349 | No Hit |
| AAGCCACCTACAGCCAACAGTCTGAAGCGCAGTCGCGAACCCCGCGCACG | 31738 | 0.10825813285044572 | No Hit |
| CGCCCGCAACCCACGTTGGCCAGCCCCGGTGAGAAATGCGGAAGCGGCGG | 31675 | 0.10804324021796799 | No Hit |
| CCGTTAGTCGCCTGCCGAATAGCCGCCGACCACGAGGGACGGCGACCAAG | 31573 | 0.10769531881300405 | No Hit |
| GCTGGCGGGTCGAAGAGACCCTCTCCTCGGTCGCGGGCGCGCTCCGAACG | 31528 | 0.10754182407551995 | No Hit |
| GCCAGCGTTTGATACGCGCGGTCACCGAAGGCCGCCTACGGGCCACGGAG | 31417 | 0.1071632037230592 | No Hit |
| CATTATTCAACCTGGATACAGCCGGACTCCTCCGGCGAACCCATATTGAT | 31148 | 0.10624564629232097 | No Hit |
| GGGAGCTCCGGCCACGAAGGCCTGCGCTGGCGGGTCGAAGAGACCCTCTC | 31014 | 0.10578857307403501 | No Hit |
| ACCCGGTCGCCGTAACAGCACCGCCCGCAACCCACGTTGGCCAGCCCCGG | 30838 | 0.10518823810076391 | No Hit |
| GTCGAAGAGACCCTCTCCTCGGTCGCGGGCGCGCTCCGAACGACGCGGCT | 30690 | 0.10468341096414957 | No Hit |
| CAGATGAAGCCACCTACAGCCAACAGTCTGAAGCGCAGTCGCGAACCCCG | 29898 | 0.10198190358442957 | No Hit |
| GCACCGCCCGCAACCCACGTTGGCCAGCCCCGGTGAGAAATGCGGAAGCG | 29553 | 0.10080511059705156 | No Hit |

## Kmer Content

| Sequence | Count | Obs/Exp Overall | Obs/Exp Max | Max Obs/Exp Position |
| --- | --- | --- | --- | --- |
| GAGAT | 12649495 | 4.355422 | 20.619356 | 3 |
| TTCAA | 9380315 | 4.1628065 | 89.22846 | 7 |
| ATTTT | 5375735 | 4.160752 | 38.902546 | 20-24 |
| AGAAA | 10627485 | 4.1012373 | 49.880108 | 4 |
| AGAGA | 13331085 | 4.0858097 | 17.954144 | 35-39 |
| TTGAT | 7277400 | 3.9819334 | 24.236118 | 50-54 |
| GATTC | 11160740 | 3.933599 | 19.238024 | 105-109 |
| GGATT | 9882140 | 3.8225443 | 30.753138 | 20-24 |
| TTTGA | 6976555 | 3.8173213 | 24.408087 | 50-54 |
| AAATG | 8167400 | 3.540892 | 54.00758 | 7 |
| TTCGC | 13655125 | 3.4827034 | 15.376761 | 95-99 |
| AAGAA | 9005365 | 3.4752471 | 52.287292 | 130-134 |
| ATCTT | 6944835 | 3.462384 | 29.730635 | 6 |
| GAGAA | 10954780 | 3.357502 | 37.723034 | 3 |
| TTTCA | 6476555 | 3.22892 | 36.578403 | 25-29 |
| CTTCG | 12618090 | 3.2182107 | 12.450689 | 100-104 |
| TTTTC | 5718875 | 3.2030838 | 31.190311 | 25-29 |
| TGATA | 6247465 | 3.042826 | 21.59371 | 50-54 |
| ATTGG | 7759835 | 3.0016081 | 17.396019 | 40-44 |
| TTGCC | 11679420 | 2.9788053 | 13.804384 | 9 |
| AGAAG | 9623480 | 2.9494753 | 24.89279 | 130-134 |
| TGATT | 5376920 | 2.9420586 | 28.172323 | 105-109 |
| AGATT | 6034125 | 2.9389188 | 21.386759 | 90-94 |
| GATTT | 5256550 | 2.8761964 | 27.95583 | 20-24 |
| TTGGC | 10269325 | 2.8745325 | 13.573366 | 40-44 |
| CGAAG | 12947550 | 2.8716006 | 11.363595 | 65-69 |
| TCCCT | 12095900 | 2.8109593 | 17.217653 | 15-19 |
| AGTTG | 7258840 | 2.8078167 | 16.549257 | 115-119 |
| ACCGG | 17476340 | 2.8048568 | 15.411684 | 75-79 |
| AAAAT | 5135550 | 2.803416 | 66.016235 | 6 |
| CGCTT | 10874480 | 2.7735074 | 18.824451 | 70-74 |
| TGATG | 7138350 | 2.7612095 | 20.436169 | 115-119 |
| GGCCA | 17178970 | 2.7571304 | 7.9190583 | 40-44 |
| ATGAA | 6341275 | 2.7491941 | 52.77289 | 9 |
| GTTTG | 6263705 | 2.721932 | 18.705555 | 50-54 |
| GAAAA | 7046395 | 2.7192638 | 46.74882 | 5 |
| GCGCT | 15077420 | 2.7185144 | 11.106621 | 70-74 |
| AAGAG | 8858690 | 2.715077 | 16.273157 | 1 |
| TCGCG | 15038135 | 2.7114313 | 10.752434 | 95-99 |
| TCTTG | 6842685 | 2.7093682 | 22.63592 | 7 |
| GAGTT | 6960370 | 2.6923645 | 16.04977 | 115-119 |
| ATACG | 8575820 | 2.690471 | 14.397726 | 55-59 |
| GATGG | 9741410 | 2.663834 | 16.819767 | 120-124 |
| TTTAC | 5331145 | 2.6578698 | 25.627638 | 70-74 |
| ACATA | 6690575 | 2.6429431 | 17.800688 | 105-109 |
| TACCG | 11636820 | 2.6418655 | 18.768528 | 75-79 |
| TCCAG | 11522480 | 2.6159074 | 17.241352 | 95-99 |
| CTTTA | 5177310 | 2.5811746 | 25.34808 | 70-74 |
| TCAAG | 8105395 | 2.5428853 | 62.437637 | 8 |
| GAAGA | 8175505 | 2.5056894 | 22.656515 | 135-137 |
| CAACC | 13603970 | 2.504916 | 13.585261 | 85-89 |
| GCTTT | 6324850 | 2.5043314 | 28.77312 | 70-74 |
| CGGTC | 13884790 | 2.503479 | 11.690078 | 60-64 |
| AGCGT | 9808230 | 2.443833 | 13.592815 | 60-64 |
| GATCT | 6923575 | 2.4402115 | 20.797613 | 5 |
| GAAGG | 9876380 | 2.4040232 | 11.045384 | 70-74 |
| CGCGG | 18822330 | 2.3991745 | 9.87254 | 35-39 |
| TTAAG | 4904375 | 2.3886743 | 25.91932 | 125-129 |
| AATGA | 5424390 | 2.3516881 | 52.463634 | 8 |
| TGGCC | 12998820 | 2.3437352 | 13.990872 | 120-124 |
| GATTG | 6042165 | 2.3371904 | 17.90207 | 35-39 |
| CGGAT | 9370800 | 2.3348422 | 19.664158 | 20-24 |
| GTGTG | 7568505 | 2.3250904 | 14.490123 | 135-137 |
| TTCCA | 7234400 | 2.3232455 | 24.295996 | 110-114 |
| AACCT | 8127025 | 2.3231633 | 16.323414 | 100-104 |
| GGTCA | 9313135 | 2.3204741 | 11.930043 | 60-64 |
| GTTAA | 4743860 | 2.3104956 | 26.247585 | 125-129 |
| GGTGA | 8440305 | 2.3080406 | 20.436338 | 115-119 |
| GGAGT | 8395920 | 2.2959037 | 11.456846 | 115-119 |
| GGTCG | 11558460 | 2.287226 | 9.001939 | 9 |
| CCACG | 15524145 | 2.2701964 | 6.690053 | 85-89 |
| CACCG | 15461475 | 2.2610319 | 13.358341 | 45-49 |
| CTCCA | 10903330 | 2.2554388 | 18.83898 | 95-99 |
| CCTAC | 10851705 | 2.24476 | 15.743897 | 90-94 |
| ATTCG | 6347495 | 2.2371724 | 16.31915 | 90-94 |
| CCGAA | 11058960 | 2.234839 | 10.780865 | 65-69 |
| GAACG | 10038610 | 2.226435 | 16.480251 | 40-44 |
| CCAGC | 15167420 | 2.2180302 | 7.663513 | 95-99 |
| GCTCC | 13332330 | 2.1903136 | 9.429792 | 15-19 |
| ATTCC | 6774600 | 2.175586 | 18.64216 | 110-114 |
| CGTTT | 5491620 | 2.174413 | 18.027187 | 45-49 |
| GTGAT | 5558935 | 2.1502707 | 20.261095 | 115-119 |
| GCCAC | 14585475 | 2.1329286 | 12.047671 | 1 |
| ATTCA | 4801865 | 2.130977 | 87.1464 | 6 |
| GTTGC | 7592275 | 2.1251876 | 12.288325 | 115-119 |
| TACAA | 5341570 | 2.1100526 | 26.93865 | 7 |
| GAAGC | 9485780 | 2.1038244 | 17.313866 | 60-64 |
| TAAGA | 4805085 | 2.083195 | 26.788424 | 130-134 |
| AACTT | 4688240 | 2.0805523 | 8.52045 | 70-74 |
| GATAC | 6604220 | 2.0719256 | 14.632367 | 50-54 |
| GACAT | 6548985 | 2.0545971 | 13.612527 | 105-109 |
| GAAAT | 4736895 | 2.053632 | 9.663609 | 4 |
| CCTCG | 12437505 | 2.043306 | 9.0315695 | 15-19 |
| GCTTC | 8006890 | 2.0421364 | 11.198092 | 100-104 |
| AACCC | 11072520 | 2.038797 | 9.485589 | 85-89 |
| ATGCA | 6489040 | 2.0357904 | 15.854121 | 80-84 |
| CTGAT | 5746280 | 2.0252743 | 25.697294 | 105-109 |
| TTCGT | 5111935 | 2.024076 | 19.183168 | 125-129 |
| ATGGC | 8030890 | 2.0009882 | 19.29525 | 120-124 |
| TGAAC | 6372445 | 1.9992113 | 32.026062 | 7 |
| GCACC | 13665935 | 1.9984583 | 10.759728 | 45-49 |
| CACCA | 10787255 | 1.9862707 | 13.814326 | 55-59 |
| TGCAA | 6319505 | 1.9826026 | 17.524675 | 85-89 |
| CGAGA | 8878740 | 1.9691907 | 28.042316 | 2 |
| AGATC | 6259205 | 1.9636849 | 17.478065 | 4 |
| CTACA | 6867555 | 1.9631356 | 37.40643 | 6 |
| TGGCG | 9917265 | 1.9624609 | 12.07927 | 65-69 |
| ACCTG | 8633820 | 1.9601052 | 13.831766 | 105-109 |
| CCTCC | 13093300 | 1.9599493 | 11.614708 | 95-99 |
| GTAGA | 5670700 | 1.9525121 | 24.931635 | 35-39 |
| CGTTA | 5507065 | 1.9409631 | 27.036947 | 125-129 |
| AAGTC | 6134375 | 1.9245223 | 13.587921 | 10-14 |
| AGAAC | 6851500 | 1.9133475 | 20.543993 | 40-44 |
| AAGGC | 8564005 | 1.8993865 | 9.834825 | 70-74 |
| TAGAG | 5503840 | 1.8950596 | 18.249647 | 35-39 |
| GGGAG | 9718245 | 1.8786979 | 9.911979 | 115-119 |
| TGGTG | 6104195 | 1.8752456 | 9.687947 | 130-134 |
| GCGGA | 10619460 | 1.8705404 | 10.160972 | 35-39 |
| AGGGC | 10528650 | 1.8545449 | 12.819181 | 30-34 |
| TCCTC | 7935010 | 1.8440125 | 7.32312 | 2 |
| ACGCG | 11487635 | 1.8437024 | 9.366448 | 55-59 |
| AACAG | 6579980 | 1.8375227 | 14.826173 | 9 |
| GCAAC | 9017810 | 1.8223554 | 14.700019 | 85-89 |
| AAGCG | 8184870 | 1.8152993 | 12.48659 | 60-64 |
| TTTTT | 2075395 | 1.8045926 | 5.807094 | 110-114 |
| CAGAA | 6461875 | 1.804541 | 15.615267 | 60-64 |
| CATAC | 6281930 | 1.795731 | 13.022674 | 105-109 |
| TACCT | 5560305 | 1.7856289 | 16.343222 | 90-94 |
| GCGTT | 6349115 | 1.7772089 | 12.841081 | 45-49 |
| AGTCG | 7085340 | 1.7653936 | 19.756739 | 7 |
| CGCGC | 15157920 | 1.7604495 | 5.3112264 | 55-59 |
| TTACC | 5478820 | 1.7594609 | 18.005356 | 75-79 |
| GCCAG | 10934185 | 1.7548767 | 8.194381 | 40-44 |
| GGGCC | 13720205 | 1.7488358 | 9.302424 | 30-34 |
| ACCCT | 8446790 | 1.7472845 | 10.3698435 | 85-89 |
| CCGTA | 7690445 | 1.7459341 | 12.495448 | 35-39 |
| CAGCC | 11939065 | 1.7459269 | 10.275907 | 9 |
| GTCGC | 9629755 | 1.7362804 | 16.548414 | 8 |
| GCGGG | 12386630 | 1.7327904 | 8.050683 | 9 |
| AGATG | 4979980 | 1.7146863 | 7.6018643 | 4 |
| TCGCC | 10408110 | 1.7099051 | 9.841013 | 9 |
| GGAGA | 6956025 | 1.6931758 | 10.672275 | 90-94 |
| CGGAG | 9610670 | 1.6928494 | 8.780274 | 85-89 |
| AGGGT | 6187075 | 1.6918846 | 15.656671 | 115-119 |
| ATATT | 2448220 | 1.6867082 | 9.475122 | 4 |
| TTATT | 2175325 | 1.6836743 | 5.541791 | 3 |
| CCATT | 5173050 | 1.6612663 | 62.12712 | 4 |
| ATACC | 5800520 | 1.6581168 | 12.1530075 | 110-114 |
| CGGGC | 13006350 | 1.6578447 | 11.21094 | 80-84 |
| CTTGC | 6484780 | 1.6539259 | 14.097115 | 8 |
| CGGGA | 9375260 | 1.6513836 | 10.775728 | 110-114 |
| CAAGA | 5911955 | 1.6509705 | 17.8393 | 9 |
| GAACC | 8158200 | 1.648642 | 20.53249 | 8 |
| CCCTC | 11007135 | 1.6476691 | 11.436298 | 15-19 |
| TACGC | 7248210 | 1.6455351 | 11.607256 | 55-59 |
| TGAAG | 4776700 | 1.6446937 | 8.533746 | 3 |
| TAACA | 4146690 | 1.6380454 | 18.758272 | 8 |
| TCGAA | 5213440 | 1.6355996 | 5.836946 | 10-14 |
| CGCCT | 9915250 | 1.6289353 | 8.093463 | 75-79 |
| AGCCA | 8039645 | 1.6246836 | 11.358731 | 100-104 |
| CGTGG | 8208975 | 1.6244191 | 14.539027 | 65-69 |
| GGCCG | 12728785 | 1.6224653 | 7.030197 | 30-34 |
| CTACG | 7090270 | 1.6096786 | 11.435723 | 75-79 |
| TGAAT | 3264970 | 1.5902027 | 9.751386 | 8 |
| GCCGT | 8788720 | 1.5846387 | 13.083058 | 1 |
| GTCTA | 4494440 | 1.5840636 | 22.324978 | 4 |
| TAATA | 2578960 | 1.5815744 | 5.207936 | 100-104 |
| CCACC | 11859490 | 1.5802191 | 9.795993 | 2 |
| GGCGG | 11193125 | 1.5658286 | 6.6553354 | 8 |
| CTATA | 3526685 | 1.5650762 | 7.5306063 | 60-64 |
| ACTTC | 4873475 | 1.5650612 | 5.9147625 | 70-74 |
| GCCAA | 7739325 | 1.5639938 | 14.927486 | 100-104 |
| TCACC | 7533270 | 1.5583158 | 9.028853 | 65-69 |
| CCTGA | 6854705 | 1.5561992 | 17.329542 | 105-109 |
| TCGTC | 6088660 | 1.5528966 | 21.176357 | 2 |
| CCGGA | 9673105 | 1.5524803 | 8.906638 | 50-54 |
| TAACT | 3491265 | 1.5493575 | 9.091388 | 70-74 |
| AAGGG | 6280105 | 1.528649 | 13.598839 | 30-34 |
| CGCTC | 9290175 | 1.5262442 | 8.843164 | 10-14 |
| CCCTA | 7348770 | 1.5201504 | 12.265971 | 90-94 |
| CCCGC | 14286135 | 1.5117997 | 5.0842195 | 30-34 |
| GAGAG | 6177645 | 1.5037091 | 5.316868 | 100-104 |
| GGCTA | 6009700 | 1.4973856 | 5.145196 | 55-59 |
| CACGA | 7409390 | 1.4973192 | 5.3822627 | 60-64 |
| CAGGG | 8497830 | 1.4968307 | 9.237385 | 115-119 |
| TTGAA | 3069120 | 1.4948139 | 10.993819 | 7 |
| GCGGT | 7553110 | 1.4946342 | 11.442031 | 60-64 |
| TCTAC | 4650465 | 1.4934441 | 20.42779 | 5 |
| AATTT | 2167415 | 1.4932468 | 13.978507 | 4 |
| GTGGT | 4855710 | 1.4917034 | 9.2652445 | 130-134 |
| CTACC | 7204480 | 1.490303 | 14.79421 | 90-94 |
| TACGG | 5975135 | 1.4887733 | 10.48533 | 80-84 |
| TATAC | 3325720 | 1.4758917 | 9.107811 | 60-64 |
| AGGCC | 9193495 | 1.4755057 | 7.131978 | 70-74 |
| ACGTT | 4184160 | 1.4747057 | 10.883237 | 135-137 |
| TCCAC | 7111535 | 1.4710766 | 6.8740005 | 75-79 |
| TGCCT | 5763335 | 1.4699233 | 8.884272 | 120-124 |
| GGTGT | 4765550 | 1.4640057 | 9.596858 | 130-134 |
| AAATA | 2678300 | 1.4620419 | 6.431863 | 105-109 |
| ACAAG | 5226525 | 1.4595575 | 17.862516 | 8 |
| CCGTT | 5699470 | 1.4536347 | 19.58886 | 125-129 |
| GTCAC | 6402005 | 1.4534242 | 10.540743 | 60-64 |
| GGGCA | 8211475 | 1.4463915 | 9.8556185 | 80-84 |
| ACCTC | 6982995 | 1.4444871 | 10.479845 | 90-94 |
| AGAGG | 5919560 | 1.4408882 | 5.5300446 | 100-104 |
| CCTTC | 6192860 | 1.4391552 | 7.6744194 | 125-129 |
| ATGCG | 5755010 | 1.4339267 | 6.1658564 | 55-59 |
| CCCTT | 6164615 | 1.4325914 | 7.728611 | 125-129 |
| CATGC | 6301380 | 1.4305798 | 11.933244 | 80-84 |
| GGCAT | 5723805 | 1.4261516 | 18.177616 | 80-84 |
| AACGC | 7044115 | 1.4235029 | 10.199626 | 40-44 |
| GCATG | 5706240 | 1.4217751 | 18.11581 | 80-84 |
| AAAAG | 3657330 | 1.4113948 | 31.758135 | 135-137 |
| CTGAA | 4497120 | 1.4108703 | 6.0673385 | 20-24 |
| CTCCT | 6050605 | 1.4060967 | 7.8852386 | 1 |
| ACGTC | 6181435 | 1.4033492 | 5.7922134 | 55-59 |
| CATTC | 4356280 | 1.3989699 | 62.833725 | 5 |
| CTCGG | 7733825 | 1.3944373 | 9.198117 | 15-19 |
| AAAGA | 3611725 | 1.3937955 | 22.969444 | 135-137 |
| TCCGA | 6116630 | 1.3886367 | 5.1771026 | 45-49 |
| CCAGA | 6861280 | 1.386555 | 10.284951 | 55-59 |
| CCAAC | 7490335 | 1.3792048 | 13.4835825 | 100-104 |
| TCTGA | 3902175 | 1.3753201 | 6.4831805 | 15-19 |
| TAGTC | 3889540 | 1.370867 | 20.877716 | 6 |
| GGGTC | 6918665 | 1.3690882 | 5.920544 | 9 |
| ACAGC | 6743030 | 1.3626586 | 15.5949 | 8 |
| CACGT | 5997730 | 1.3616432 | 5.7653713 | 110-114 |
| GCGTG | 6863755 | 1.3582224 | 10.925649 | 65-69 |
| CTCTC | 5749855 | 1.3362056 | 8.284704 | 1 |
| GACGC | 8318700 | 1.3351059 | 14.195717 | 50-54 |
| CGTAA | 4240740 | 1.330437 | 14.556187 | 6 |
| GAATA | 3050580 | 1.3225474 | 10.628109 | 15-19 |
| CAAGG | 5918550 | 1.3126585 | 11.282321 | 25-29 |
| GTAGT | 3390860 | 1.3116301 | 7.388153 | 80-84 |
| CTCCC | 8740560 | 1.3083832 | 8.33604 | 15-19 |
| TGTGC | 4653985 | 1.3027177 | 13.177431 | 135-137 |
| CGTAG | 5225240 | 1.3019284 | 18.0664 | 35-39 |
| CGCAC | 8868045 | 1.2968317 | 10.981258 | 45-49 |
| CCGGG | 10138730 | 1.2923257 | 6.9004097 | 75-79 |
| TCTCC | 5538755 | 1.2871481 | 7.6225634 | 2 |
| GGACG | 7299795 | 1.2858056 | 13.67117 | 50-54 |
| CAAGT | 4094595 | 1.2845873 | 61.26254 | 9 |
| GCCTC | 7781100 | 1.2783246 | 5.483213 | 120-124 |
| GGGTG | 5867350 | 1.2742528 | 16.058 | 115-119 |
| GCTAT | 3589245 | 1.2650281 | 5.7642035 | 60-64 |
| GTAAC | 3993435 | 1.2528504 | 14.818471 | 7 |
| GTCGT | 4457575 | 1.2477396 | 19.141254 | 1 |
| ACGGA | 5594850 | 1.2408661 | 9.693391 | 85-89 |
| ACCAG | 6126155 | 1.2379979 | 10.48045 | 55-59 |
| ACCGC | 8462900 | 1.2375847 | 11.786888 | 10-14 |
| GCCTA | 5419505 | 1.2303708 | 9.724969 | 75-79 |
| TTGGT | 2822085 | 1.2263546 | 6.5139356 | 7 |
| ACAGT | 3898605 | 1.2230998 | 6.1059976 | 15-19 |
| CACGG | 7607105 | 1.2208986 | 6.896036 | 85-89 |
| GGCGC | 9552910 | 1.2176546 | 7.128811 | 70-74 |
| CGACA | 6001870 | 1.2128819 | 8.995998 | 105-109 |
| ACGAA | 4316605 | 1.2054536 | 6.9601336 | 125-129 |
| CGGCT | 6659560 | 1.2007432 | 5.012886 | 70-74 |
| AGGGA | 4926450 | 1.1991541 | 5.264286 | 35-39 |
| GCGAC | 7432765 | 1.192918 | 6.958462 | 105-109 |
| TTAGT | 2150455 | 1.1766522 | 32.668987 | 5 |
| CTGCG | 6493310 | 1.1707678 | 8.32201 | 1 |
| GAGCT | 4697565 | 1.1704522 | 5.0850563 | 115-119 |
| GTGCA | 4680375 | 1.166169 | 11.876513 | 135-137 |
| ACCGA | 5756505 | 1.1632975 | 8.671405 | 65-69 |
| TAAAT | 1890110 | 1.1591299 | 7.225638 | 105-109 |
| AATAG | 2661625 | 1.1539199 | 8.034735 | 15-19 |
| CAGTC | 5064235 | 1.1497152 | 8.421052 | 5 |
| TCGGA | 4610775 | 1.1488274 | 13.674932 | 20-24 |
| GGTTC | 4088755 | 1.1445016 | 6.877791 | 65-69 |
| AAATT | 1864285 | 1.1432924 | 14.441301 | 3 |
| CGCGA | 7112520 | 1.1415205 | 7.289767 | 100-104 |
| CGTCT | 4448805 | 1.134656 | 17.21354 | 3 |
| GCCCG | 9723075 | 1.1292435 | 8.373881 | 125-129 |
| AGTAG | 3259025 | 1.1221341 | 6.521048 | 80-84 |
| TCGTG | 3997125 | 1.118853 | 8.646219 | 130-134 |
| CAACA | 4389060 | 1.1167996 | 5.276495 | 10-14 |
| TACGT | 3167950 | 1.1165428 | 6.086028 | 60-64 |
| CGGGT | 5635615 | 1.115194 | 5.516979 | 8 |
| ATTTG | 2036420 | 1.1142563 | 12.905016 | 5 |
| GTCCC | 6773765 | 1.1128336 | 32.444386 | 1 |
| CACCT | 5358725 | 1.1084942 | 14.382466 | 3 |
| AGGTT | 2847620 | 1.1014977 | 8.360834 | 65-69 |
| AGTCT | 3111775 | 1.0967441 | 7.486319 | 15-19 |
| TTATC | 2192515 | 1.0930898 | 5.8352265 | 50-54 |
| ATCAT | 2452690 | 1.0884575 | 5.909726 | 50-54 |
| CGCCA | 7432990 | 1.0869743 | 7.463113 | 50-54 |
| GTGGC | 5474490 | 1.08331 | 14.304608 | 65-69 |
| TCGGT | 3824290 | 1.070474 | 8.341274 | 7 |
| CCAGG | 6657860 | 1.06855 | 8.517943 | 110-114 |
| CGCCG | 9200160 | 1.0685118 | 5.318639 | 3 |
| CACTA | 3735700 | 1.0678743 | 5.3874316 | 70-74 |
| TACAG | 3397440 | 1.0658703 | 21.790852 | 7 |
| ATAAA | 1950640 | 1.0648236 | 5.1691284 | 105-109 |
| GCAGT | 4250385 | 1.0590322 | 9.323453 | 4 |
| GTCAG | 4249125 | 1.0587182 | 6.026877 | 1 |
| TATTG | 1934050 | 1.058243 | 7.122703 | 5 |
| ACGGG | 5885885 | 1.0367557 | 7.7014613 | 80-84 |
| TGCCC | 6291455 | 1.033597 | 7.117578 | 30-34 |
| CGGAC | 6383225 | 1.0244725 | 9.666819 | 50-54 |
| ACGCC | 6972070 | 1.0195711 | 7.802212 | 50-54 |
| TTGGA | 2630325 | 1.017445 | 5.767229 | 95-99 |
| GTTCG | 3609495 | 1.0103499 | 5.194712 | 65-69 |
| GCGCG | 7843545 | 0.99977165 | 5.939135 | 55-59 |
| ACGCA | 4914240 | 0.9930893 | 10.055529 | 40-44 |
| GAGTA | 2881435 | 0.9921239 | 5.9485774 | 75-79 |
| TGCGC | 5496855 | 0.99110323 | 8.275836 | 2 |
| TATCA | 2225045 | 0.98743296 | 6.329584 | 50-54 |
| GGATG | 3555855 | 0.9723652 | 5.2647953 | 55-59 |
| ACGAG | 4341750 | 0.9629445 | 5.470628 | 30-34 |
| CAGCG | 5981570 | 0.96000916 | 7.136755 | 45-49 |
| CCGCT | 5829955 | 0.95777917 | 11.782626 | 10-14 |
| GATGC | 3833905 | 0.95526135 | 5.06996 | 55-59 |
| AGCTG | 3814885 | 0.9505223 | 5.755835 | 50-54 |
| CCGCG | 8035915 | 0.9332957 | 5.763764 | 30-34 |
| ACCAA | 3661775 | 0.93174136 | 5.1542506 | 45-49 |
| GTTTT | 1513825 | 0.930546 | 5.051574 | 1 |
| GCTGG | 4681990 | 0.9264876 | 9.657298 | 5 |
| TCCCA | 4466400 | 0.92390966 | 39.139236 | 2 |
| CCCAT | 4421635 | 0.9146497 | 39.86523 | 3 |
| TGCGG | 4556455 | 0.90164626 | 7.3500533 | 50-54 |
| AAGCT | 2844515 | 0.8924027 | 5.7649713 | 45-49 |
| ACTAC | 3089540 | 0.8831653 | 5.027488 | 70-74 |
| AACCG | 4342935 | 0.8776377 | 20.457026 | 9 |
| GAGGT | 3194765 | 0.87362343 | 5.6021566 | 65-69 |
| GCCGC | 7506105 | 0.8717633 | 5.3921256 | 70-74 |
| TATAT | 1253175 | 0.8633785 | 8.008173 | 3 |
| GGCGA | 4814745 | 0.8480822 | 8.889034 | 1 |
| CTGGC | 4605000 | 0.8302985 | 8.59556 | 6 |
| GCGCA | 5135180 | 0.8241682 | 6.3191366 | 2 |
| CAGAT | 2612470 | 0.8196038 | 5.9236846 | 3 |
| GTCGG | 4090950 | 0.8095306 | 5.6193943 | 1 |
| GTCCA | 3535530 | 0.8026587 | 5.295424 | 60-64 |
| GATGA | 2328345 | 0.8016862 | 8.028449 | 1 |
| GCGAG | 4469745 | 0.787313 | 21.478624 | 1 |
| CCCGT | 4746170 | 0.7797286 | 9.631872 | 125-129 |
| ATAGC | 2467790 | 0.7742136 | 6.929137 | 20-24 |
| TTATA | 1113500 | 0.76714903 | 7.99755 | 2 |
| TTCGG | 2739035 | 0.76669544 | 6.824721 | 70-74 |
| GGCCC | 6547420 | 0.7604211 | 6.2639565 | 120-124 |
| GTTAG | 1957815 | 0.7573091 | 22.823822 | 4 |
| CGCTG | 3991145 | 0.7196182 | 8.455218 | 4 |
| ACCTA | 2510845 | 0.7177415 | 19.147251 | 4 |
| CTTAT | 1432545 | 0.7142028 | 5.9538145 | 1 |
| GAATG | 2063930 | 0.7106439 | 6.8121796 | 9 |
| CGAAT | 2228945 | 0.69928145 | 6.688817 | 15-19 |
| TCTAA | 1489320 | 0.66093206 | 5.3156257 | 100-104 |
| TCAGA | 2063110 | 0.6472544 | 5.557713 | 2 |
| CTCGT | 2381790 | 0.60746926 | 6.823279 | 1 |
| GACGT | 2435470 | 0.6068253 | 5.282639 | 55-59 |
| CGCAG | 3112615 | 0.49955764 | 5.8644266 | 3 |
| AGCGC | 3037700 | 0.48753422 | 6.0582113 | 1 |

Produced by FastQC (version 0.10.1)
