# Supplementary material for: Freshwater sponge hosts and their green algae symbionts: a tractable model to understand intracellular symbiosis
Source: PeerJ. 2021 Feb 11;9:e10654. doi: 10.7717/peerj.10654 (PMC7882143; doi:10.7717/peerj.10654)
Supplement: Supplemental Information 25 [file peerj-09-10654-s025.zip › EmApo1_Clean_Data2.fq_fastqc/fastqc_report.html]

EmApo1\_Clean\_Data2.fq.gz FastQC Report


FastQC Report

Tue 10 Sep 2019  
EmApo1\_Clean\_Data2.fq.gz

## Summary

- Basic Statistics
- Per base sequence quality
- Per sequence quality scores
- Per base sequence content
- Per base GC content
- Per sequence GC content
- Per base N content
- Sequence Length Distribution
- Sequence Duplication Levels
- Overrepresented sequences
- Kmer Content

## Basic Statistics

| Measure | Value |
| --- | --- |
| Filename | EmApo1\_Clean\_Data2.fq.gz |
| File type | Conventional base calls |
| Encoding | Sanger / Illumina 1.9 |
| Total Sequences | 29316966 |
| Filtered Sequences | 0 |
| Sequence length | 100-141 |
| %GC | 58 |

## Per base sequence quality

## Per sequence quality scores

## Per base sequence content

## Per base GC content

## Per sequence GC content

## Per base N content

## Sequence Length Distribution

## Sequence Duplication Levels

## Overrepresented sequences

| Sequence | Count | Percentage | Possible Source |
| --- | --- | --- | --- |
| GGCAACTCCCGGTATGTCGCGAAGCGCGAATCTCCGTGGCCCGTAGGCGG | 665981 | 2.271657305875376 | No Hit |
| CTCGGAGACGCCGGAGGGGACCCTGGGAAGAGTTCTCTTTTCTTCTTAAC | 626245 | 2.136118041682758 | No Hit |
| CGGAGACGCCGGAGGGGACCCTGGGAAGAGTTCTCTTTTCTTCTTAACGG | 515732 | 1.7591588433809966 | No Hit |
| GCAGGTGCACACCACGAAGGGAGGCAACTCCCGGTATGTCGCGAAGCGCG | 514682 | 1.7555772995063679 | No Hit |
| GTGCACACCACGAAGGGAGGCAACTCCCGGTATGTCGCGAAGCGCGAATC | 451126 | 1.5387881542721713 | No Hit |
| GTTTCGACGTGCCGGCACGCCGGCGAGGACTTCGGCCCTCGCAGGCGTAG | 414292 | 1.413147595150194 | No Hit |
| AGCATATGTAGCCAGGCGTCGCCCCGCGTGAGGTTCAGGTTTCGACGTGC | 319546 | 1.0899695418686912 | No Hit |
| GGTGCACACCACGAAGGGAGGCAACTCCCGGTATGTCGCGAAGCGCGAAT | 304768 | 1.0395618700789162 | No Hit |
| GCCAGGCGTCGCCCCGCGTGAGGTTCAGGTTTCGACGTGCCGGCACGCCG | 302161 | 1.0306694082873378 | No Hit |
| CAGGTTTCGACGTGCCGGCACGCCGGCGAGGACTTCGGCCCTCGCAGGCG | 268606 | 0.9162134990367011 | No Hit |
| GGAGACGCCGGAGGGGACCCTGGGAAGAGTTCTCTTTTCTTCTTAACGGG | 255024 | 0.8698853762698364 | No Hit |
| GGCGTCGCCCCGCGTGAGGTTCAGGTTTCGACGTGCCGGCACGCCGGCGA | 226153 | 0.7714065636942105 | No Hit |
| GTGCCGGCACGCCGGCGAGGACTTCGGCCCTCGCAGGCGTAGCCGACCGC | 224949 | 0.7672997267179694 | No Hit |
| CACGAAGGGAGGCAACTCCCGGTATGTCGCGAAGCGCGAATCTCCGTGGC | 224259 | 0.7649461407432133 | No Hit |
| GCCGGAGGGGACCCTGGGAAGAGTTCTCTTTTCTTCTTAACGGGCCATCA | 198611 | 0.6774609623656145 | No Hit |
| CGACGTGCCGGCACGCCGGCGAGGACTTCGGCCCTCGCAGGCGTAGCCGA | 178849 | 0.6100528956509347 | No Hit |
| GGGAAGAGTTCTCTTTTCTTCTTAACGGGCCATCACCCTGGAATCAGGTT | 175046 | 0.5970808848364458 | No Hit |
| GGGAAGCATATGTAGCCAGGCGTCGCCCCGCGTGAGGTTCAGGTTTCGAC | 173649 | 0.5923157259861065 | No Hit |
| GGGACGTATAGCCGCGTCGTTCGGAGCGCGCCCGCGACCGAGGAGAGGGT | 170442 | 0.5813766676947403 | No Hit |
| GGAAGAGTTCTCTTTTCTTCTTAACGGGCCATCACCCTGGAATCAGGTTG | 164718 | 0.561852137086764 | No Hit |
| CGTGCCGGCACGCCGGCGAGGACTTCGGCCCTCGCAGGCGTAGCCGACCG | 153630 | 0.5240310337706842 | No Hit |
| CACATTTCCCCGCGGGCTGCAGGTGCACACCACGAAGGGAGGCAACTCCC | 150724 | 0.5141186847233783 | No Hit |
| GGCTGCAGGTGCACACCACGAAGGGAGGCAACTCCCGGTATGTCGCGAAG | 150301 | 0.5126758341910278 | No Hit |
| GGAAGCTCCCTGTAGCACGGTGCAACTCGCCATCTTGGCGACCGGCACCC | 147089 | 0.5017197209288301 | No Hit |
| GAGACGCCGGAGGGGACCCTGGGAAGAGTTCTCTTTTCTTCTTAACGGGC | 141907 | 0.48404394915899546 | No Hit |
| GACGTATAGCCGCGTCGTTCGGAGCGCGCCCGCGACCGAGGAGAGGGTCT | 140942 | 0.48075233978850335 | No Hit |
| CTCTTTTCTTCTTAACGGGCCATCACCCTGGAATCAGGTTGGCTGGAGGT | 137344 | 0.4684795827781088 | No Hit |
| GCTGCAGGTGCACACCACGAAGGGAGGCAACTCCCGGTATGTCGCGAAGC | 134515 | 0.458829880281609 | No Hit |
| GACGTGCCGGCACGCCGGCGAGGACTTCGGCCCTCGCAGGCGTAGCCGAC | 131561 | 0.44875380351432004 | No Hit |
| GTCGGAAGCGAGGGTCGACGAAGCGGGCTGGCGGGGGGGCCCTCTCGGGG | 127079 | 0.4334657276609046 | No Hit |
| GTTCTCTTTTCTTCTTAACGGGCCATCACCCTGGAATCAGGTTGGCTGGA | 119957 | 0.4091726272084226 | No Hit |
| CCGGTATGTCGCGAAGCGCGAATCTCCGTGGCCCGTAGGCGGCCTTCGGT | 110172 | 0.37579604929104876 | No Hit |
| GAAGAGTTCTCTTTTCTTCTTAACGGGCCATCACCCTGGAATCAGGTTGG | 106974 | 0.3648876899471794 | No Hit |
| GGCGAAGTTAGGGACGTATAGCCGCGTCGTTCGGAGCGCGCCCGCGACCG | 99473 | 0.3393018227056647 | No Hit |
| GCAACTCCCGGTATGTCGCGAAGCGCGAATCTCCGTGGCCCGTAGGCGGC | 98753 | 0.33684590690591926 | No Hit |
| GTCCCGACTTTGCGGAAGGGATGTATTTATTAGATCCAAAGCCAATGCGG | 97829 | 0.33369414829624594 | No Hit |
| GGACGTATAGCCGCGTCGTTCGGAGCGCGCCCGCGACCGAGGAGAGGGTC | 97776 | 0.3335133656054313 | No Hit |
| TCGGAGACGCCGGAGGGGACCCTGGGAAGAGTTCTCTTTTCTTCTTAACG | 96172 | 0.3280421309626651 | No Hit |
| GGGGAAGCTCCCTGTAGCACGGTGCAACTCGCCATCTTGGCGACCGGCAC | 95829 | 0.32687215996361974 | No Hit |
| GAAGCGGGCTGGCGGGGGGGCCCTCTCGGGGGTCCTGCCGCCGGAGCGTG | 93374 | 0.318498169285321 | No Hit |
| TGCAGGTGCACACCACGAAGGGAGGCAACTCCCGGTATGTCGCGAAGCGC | 92728 | 0.31629466705388276 | No Hit |
| CCCGCGTGAGGTTCAGGTTTCGACGTGCCGGCACGCCGGCGAGGACTTCG | 88400 | 0.30153188430207956 | No Hit |
| CATTTCCCCGCGGGCTGCAGGTGCACACCACGAAGGGAGGCAACTCCCGG | 87178 | 0.2973636494308449 | No Hit |
| AGACGCCGGAGGGGACCCTGGGAAGAGTTCTCTTTTCTTCTTAACGGGCC | 86278 | 0.29429375468116314 | No Hit |
| TTCGACGTGCCGGCACGCCGGCGAGGACTTCGGCCCTCGCAGGCGTAGCC | 86173 | 0.29393560029370025 | No Hit |
| GACGCCGGAGGGGACCCTGGGAAGAGTTCTCTTTTCTTCTTAACGGGCCA | 85628 | 0.2920766084730596 | No Hit |
| CGTATAGCCGCGTCGTTCGGAGCGCGCCCGCGACCGAGGAGAGGGTCTCT | 83940 | 0.28631885032032306 | No Hit |
| CCCCGCGTGAGGTTCAGGTTTCGACGTGCCGGCACGCCGGCGAGGACTTC | 83691 | 0.28546951277291105 | No Hit |
| GGCGAATTGTAGCCGAGAGAGGCACCTGCGCTCGGCAGGCGGTCGACCAA | 82966 | 0.28299654200233404 | No Hit |
| ATTTCCCCGCGGGCTGCAGGTGCACACCACGAAGGGAGGCAACTCCCGGT | 82946 | 0.2829283221190078 | No Hit |
| CCGGAGGGGACCCTGGGAAGAGTTCTCTTTTCTTCTTAACGGGCCATCAC | 81124 | 0.2767134907479853 | No Hit |
| GTCGACGAAGCGGGCTGGCGGGGGGGCCCTCTCGGGGGTCCTGCCGCCGG | 80299 | 0.273899420560777 | No Hit |
| CCCGACTTTGCGGAAGGGATGTATTTATTAGATCCAAAGCCAATGCGGGG | 79436 | 0.27095573259524874 | No Hit |
| CAGGTGCACACCACGAAGGGAGGCAACTCCCGGTATGTCGCGAAGCGCGA | 78999 | 0.2694651281445699 | No Hit |
| CGGAGGGGACCCTGGGAAGAGTTCTCTTTTCTTCTTAACGGGCCATCACC | 77130 | 0.2630899800477307 | No Hit |
| CGCAACGACACATTTCCCCGCGGGCTGCAGGTGCACACCACGAAGGGAGG | 74010 | 0.2524476782488338 | No Hit |
| GCATATGTAGCCAGGCGTCGCCCCGCGTGAGGTTCAGGTTTCGACGTGCC | 73652 | 0.2512265423372937 | No Hit |
| CTTTTCTTCTTAACGGGCCATCACCCTGGAATCAGGTTGGCTGGAGGTAG | 73482 | 0.25064667332902046 | No Hit |
| GTTCAGGTTTCGACGTGCCGGCACGCCGGCGAGGACTTCGGCCCTCGCAG | 73108 | 0.2493709615108194 | No Hit |
| AGGCAACTCCCGGTATGTCGCGAAGCGCGAATCTCCGTGGCCCGTAGGCG | 71027 | 0.24227268265072177 | No Hit |
| CTGGAATCAGGTTGGCTGGAGGTAGGGTTGCATGCCCGGTAAAGCGCCAC | 70731 | 0.24126302837749308 | No Hit |
| AGCGAGGGTCGACGAAGCGGGCTGGCGGGGGGGCCCTCTCGGGGGTCCTG | 69751 | 0.2379202540945062 | No Hit |
| GCCCCGCGTGAGGTTCAGGTTTCGACGTGCCGGCACGCCGGCGAGGACTT | 63863 | 0.2178363204432546 | No Hit |
| GCGAGGGTCGACGAAGCGGGCTGGCGGGGGGGCCCTCTCGGGGGTCCTGC | 62525 | 0.21327241024872765 | No Hit |
| CGAAGGGAGGCAACTCCCGGTATGTCGCGAAGCGCGAATCTCCGTGGCCC | 62508 | 0.21321442334790033 | No Hit |
| GCCGCGTCGTTCGGAGCGCGCCCGCGACCGAGGAGAGGGTCTCTTCGACC | 62367 | 0.2127334731704502 | No Hit |
| GTCGCGAAGCGCGAATCTCCGTGGCCCGTAGGCGGCCTTCGGTGACCGCG | 61439 | 0.2095680705841116 | No Hit |
| GGGGACCCTGGGAAGAGTTCTCTTTTCTTCTTAACGGGCCATCACCCTGG | 61387 | 0.2093906988874633 | No Hit |
| GGGAGGCAACTCCCGGTATGTCGCGAAGCGCGAATCTCCGTGGCCCGTAG | 61213 | 0.20879718590252483 | No Hit |
| ACGAAGGGAGGCAACTCCCGGTATGTCGCGAAGCGCGAATCTCCGTGGCC | 60986 | 0.20802289022677176 | No Hit |
| AGCGGGCTGGCGGGGGGGCCCTCTCGGGGGTCCTGCCGCCGGAGCGTGGA | 60144 | 0.2051508331387361 | No Hit |
| GGCGTGTGCCTGTAACCGTAGTGAATCAACGGGGCTTGATCTGGCGAATA | 59947 | 0.20447886728797243 | No Hit |
| CTTCGACCCGCCAGCGCAGGCCTTCGTGGCCGGAGCTCCCGCGTTCCGGT | 59444 | 0.20276313722231692 | No Hit |
| AAGCGAGGGTCGACGAAGCGGGCTGGCGGGGGGGCCCTCTCGGGGGTCCT | 59378 | 0.20253801160734028 | No Hit |
| GCGTGAGGTTCAGGTTTCGACGTGCCGGCACGCCGGCGAGGACTTCGGCC | 56550 | 0.19289172010500677 | No Hit |
| GGCGGTGCTGTTACGGCGACCGGGTGGTGCCCTGACCCGCCTCTCGGGGC | 56531 | 0.19282691121584683 | No Hit |
| AAGCGGGCTGGCGGGGGGGCCCTCTCGGGGGTCCTGCCGCCGGAGCGTGG | 56117 | 0.1914147596309932 | No Hit |
| GGAGGGGACCCTGGGAAGAGTTCTCTTTTCTTCTTAACGGGCCATCACCC | 54709 | 0.18661207984482434 | No Hit |
| AAGCATATGTAGCCAGGCGTCGCCCCGCGTGAGGTTCAGGTTTCGACGTG | 54272 | 0.1851214753941455 | No Hit |
| GCCGGCACGCCGGCGAGGACTTCGGCCCTCGCAGGCGTAGCCGACCGCCG | 53266 | 0.18169001526283451 | No Hit |
| TTTCGACGTGCCGGCACGCCGGCGAGGACTTCGGCCCTCGCAGGCGTAGC | 52044 | 0.17752178039159985 | No Hit |
| CAGGCGTCGCCCCGCGTGAGGTTCAGGTTTCGACGTGCCGGCACGCCGGC | 51471 | 0.17556728073430244 | No Hit |
| GTCTCTTCGACCCGCCAGCGCAGGCCTTCGTGGCCGGAGCTCCCGCGTTC | 47640 | 0.16249976208315692 | No Hit |
| GTCGATTCAGACATTTGGCATTTGCGCTTGGCTGAAAAGCCAATGGCGCG | 47336 | 0.16146281985659772 | No Hit |
| GAAGCATATGTAGCCAGGCGTCGCCCCGCGTGAGGTTCAGGTTTCGACGT | 46039 | 0.1570387604228896 | No Hit |
| CCCTGACCCGCCTCTCGGGGCGAAGTTAGGGACGTATAGCCGCGTCGTTC | 45819 | 0.1562883417063007 | No Hit |
| GTCGCCCCGCGTGAGGTTCAGGTTTCGACGTGCCGGCACGCCGGCGAGGA | 44955 | 0.15334124274660618 | No Hit |
| GCAACAAGTCCCGACTTTGCGGAAGGGATGTATTTATTAGATCCAAAGCC | 44271 | 0.15100812273684802 | No Hit |
| GCGTCGCCCCGCGTGAGGTTCAGGTTTCGACGTGCCGGCACGCCGGCGAG | 43045 | 0.14682624388894813 | No Hit |
| CGTCGCCCCGCGTGAGGTTCAGGTTTCGACGTGCCGGCACGCCGGCGAGG | 42314 | 0.14433280715337324 | No Hit |
| GAAGCTCCCTGTAGCACGGTGCAACTCGCCATCTTGGCGACCGGCACCCA | 42231 | 0.14404969463756925 | No Hit |
| CACCACGAAGGGAGGCAACTCCCGGTATGTCGCGAAGCGCGAATCTCCGT | 41930 | 0.143022985393509 | No Hit |
| CCTGACCCGCCTCTCGGGGCGAAGTTAGGGACGTATAGCCGCGTCGTTCG | 40401 | 0.13780757531321625 | No Hit |
| GTGAGGTTCAGGTTTCGACGTGCCGGCACGCCGGCGAGGACTTCGGCCCT | 40375 | 0.1377188894648921 | No Hit |
| CTCTTCGACCCGCCAGCGCAGGCCTTCGTGGCCGGAGCTCCCGCGTTCCG | 40141 | 0.13692071682997484 | No Hit |
| GGCGACCGGGTGGTGCCCTGACCCGCCTCTCGGGGCGAAGTTAGGGACGT | 39580 | 0.1350071491026732 | No Hit |
| GCCCTGACCCGCCTCTCGGGGCGAAGTTAGGGACGTATAGCCGCGTCGTT | 39420 | 0.13446139003606308 | No Hit |
| GTATAGCCGCGTCGTTCGGAGCGCGCCCGCGACCGAGGAGAGGGTCTCTT | 39076 | 0.13328800804285137 | No Hit |
| CTGGGAAGAGTTCTCTTTTCTTCTTAACGGGCCATCACCCTGGAATCAGG | 38965 | 0.1329093876903906 | No Hit |
| CCCGGTATGTCGCGAAGCGCGAATCTCCGTGGCCCGTAGGCGGCCTTCGG | 38771 | 0.13224765482212586 | No Hit |
| GGGACCCTGGGAAGAGTTCTCTTTTCTTCTTAACGGGCCATCACCCTGGA | 38688 | 0.13196454230632187 | No Hit |
| CCCCGCGGGCTGCAGGTGCACACCACGAAGGGAGGCAACTCCCGGTATGT | 38265 | 0.1305216917739714 | No Hit |
| GTGGGTTGCGGGCGGTGCTGTTACGGCGACCGGGTGGTGCCCTGACCCGC | 38072 | 0.129863369899873 | No Hit |
| CGGTATGTCGCGAAGCGCGAATCTCCGTGGCCCGTAGGCGGCCTTCGGTG | 36697 | 0.12517325292119247 | No Hit |
| CAACTCCCGGTATGTCGCGAAGCGCGAATCTCCGTGGCCCGTAGGCGGCC | 35944 | 0.12260477431395868 | No Hit |
| CCGCGTGAGGTTCAGGTTTCGACGTGCCGGCACGCCGGCGAGGACTTCGG | 35902 | 0.12246151255897353 | No Hit |
| GCACACCACGAAGGGAGGCAACTCCCGGTATGTCGCGAAGCGCGAATCTC | 35216 | 0.12012157056088273 | No Hit |
| CGGAAGCGAGGGTCGACGAAGCGGGCTGGCGGGGGGGCCCTCTCGGGGGT | 34644 | 0.11817048189775162 | No Hit |
| GAGGCAACTCCCGGTATGTCGCGAAGCGCGAATCTCCGTGGCCCGTAGGC | 34593 | 0.11799652119526965 | No Hit |
| GACACATTTCCCCGCGGGCTGCAGGTGCACACCACGAAGGGAGGCAACTC | 34371 | 0.11723928049034815 | No Hit |
| GCGAACTCGGAGACGCCGGAGGGGACCCTGGGAAGAGTTCTCTTTTCTTC | 34292 | 0.11696981195120941 | No Hit |
| GCTCCCTGTAGCACGGTGCAACTCGCCATCTTGGCGACCGGCACCCACCA | 34142 | 0.11645816282626245 | No Hit |
| GTCGGGCTGCGGTCGGAAGCGAGGGTCGACGAAGCGGGCTGGCGGGGGGG | 33856 | 0.11548261849469689 | No Hit |
| AAGGGAGGCAACTCCCGGTATGTCGCGAAGCGCGAATCTCCGTGGCCCGT | 33658 | 0.1148072416497669 | No Hit |
| CTCCGGCGCACAGCCGGCGAATTGTAGCCGAGAGAGGCACCTGCGCTCGG | 33054 | 0.11274700117331377 | No Hit |
| GCGGGCTGGCGGGGGGGCCCTCTCGGGGGTCCTGCCGCCGGAGCGTGGAG | 32709 | 0.11157020818593574 | No Hit |
| TCGACGTGCCGGCACGCCGGCGAGGACTTCGGCCCTCGCAGGCGTAGCCG | 31819 | 0.10853442337791708 | No Hit |
| TCTTTTCTTCTTAACGGGCCATCACCCTGGAATCAGGTTGGCTGGAGGTA | 31135 | 0.1062013033681589 | No Hit |
| CATGCAACAAGTCCCGACTTTGCGGAAGGGATGTATTTATTAGATCCAAA | 30977 | 0.10566236628988142 | No Hit |
| AGCCAGGCGTCGCCCCGCGTGAGGTTCAGGTTTCGACGTGCCGGCACGCC | 30700 | 0.1047175209058127 | No Hit |
| CGGCGACCGGGTGGTGCCCTGACCCGCCTCTCGGGGCGAAGTTAGGGACG | 30637 | 0.10450262827333497 | No Hit |
| GGTTTCGACGTGCCGGCACGCCGGCGAGGACTTCGGCCCTCGCAGGCGTA | 30102 | 0.10267774639435745 | No Hit |
| CTTTGCGGAAGGGATGTATTTATTAGATCCAAAGCCAATGCGGGGGGCAA | 29778 | 0.10157258428447201 | No Hit |
| GGGCGGTGCTGTTACGGCGACCGGGTGGTGCCCTGACCCGCCTCTCGGGG | 29776 | 0.10156576229613937 | No Hit |
| TTCCCCGCGGGCTGCAGGTGCACACCACGAAGGGAGGCAACTCCCGGTAT | 29539 | 0.10075735667872317 | No Hit |
| GACGAAGCGGGCTGGCGGGGGGGCCCTCTCGGGGGTCCTGCCGCCGGAGC | 29348 | 0.10010585679295736 | No Hit |
| GTAATTCTAGAGCTAATACATGCAACAAGTCCCGACTTTGCGGAAGGGAT | 29318 | 0.10000352696796797 | No Hit |

## Kmer Content

| Sequence | Count | Obs/Exp Overall | Obs/Exp Max | Max Obs/Exp Position |
| --- | --- | --- | --- | --- |
| TCTCT | 14492210 | 4.970539 | 16.222313 | 30-34 |
| TTCTC | 13255840 | 4.5464897 | 19.700197 | 130-134 |
| TTTCT | 9865705 | 4.4832053 | 21.29947 | 35-39 |
| TTCTT | 9210730 | 4.1855693 | 27.822832 | 40-44 |
| ATCTC | 11314355 | 4.080704 | 11.108058 | 120-124 |
| GAATC | 11301075 | 3.908496 | 14.7125635 | 60-64 |
| ATCAA | 7387585 | 3.9036622 | 13.496103 | 85-89 |
| CTCTT | 10564535 | 3.6234245 | 11.052607 | 30-34 |
| TCAAA | 6686375 | 3.5331368 | 13.3038435 | 85-89 |
| CATTT | 7206740 | 3.443782 | 11.65888 | 3 |
| CTTCT | 9687875 | 3.3227482 | 15.280588 | 110-114 |
| CTTCG | 13952035 | 3.2935407 | 14.329501 | 70-74 |
| AAGAT | 6532195 | 3.1475818 | 11.984081 | 110-114 |
| TATGT | 7208205 | 3.1410306 | 27.365574 | 5 |
| AGGTT | 10337050 | 3.1002605 | 11.671005 | 2 |
| ATTTC | 6480605 | 3.0967948 | 11.1016245 | 4 |
| AATCT | 6053405 | 3.041817 | 12.297866 | 50-54 |
| GCCAA | 11621185 | 3.0335333 | 10.202863 | 80-84 |
| GCGAA | 12610675 | 3.001821 | 10.352222 | 40-44 |
| TATCA | 5934290 | 2.9819617 | 13.22675 | 90-94 |
| TTGAA | 6383630 | 2.925157 | 15.371243 | 135-137 |
| GTTCT | 9268750 | 2.8989332 | 15.721526 | 130-134 |
| AATCA | 5475620 | 2.893364 | 16.365429 | 60-64 |
| CCAAT | 7570905 | 2.8713722 | 11.265639 | 100-104 |
| CATCA | 7516695 | 2.8508127 | 12.821289 | 50-54 |
| TGGCC | 17410290 | 2.8287075 | 10.890247 | 75-79 |
| AATCC | 7322485 | 2.777156 | 10.368426 | 105-109 |
| CGAAG | 11606580 | 2.7628083 | 13.919739 | 3 |
| CAACT | 7238010 | 2.7451172 | 36.32366 | 3 |
| GGCAA | 11482130 | 2.7331843 | 23.420782 | 1 |
| TCTTC | 7939175 | 2.722979 | 11.109306 | 35-39 |
| GGTTG | 13783120 | 2.7056355 | 9.885731 | 85-89 |
| TTCGG | 12407555 | 2.6709135 | 7.5406857 | 70-74 |
| TCACC | 9802925 | 2.6685135 | 9.976345 | 55-59 |
| AACTC | 7006625 | 2.6573615 | 38.089497 | 4 |
| GACTT | 8030590 | 2.6411974 | 6.8060274 | 20-24 |
| GACCG | 15289170 | 2.612176 | 6.5749607 | 80-84 |
| CTGGA | 11482330 | 2.5992022 | 9.944864 | 60-64 |
| AAGCG | 10903985 | 2.5955641 | 10.173627 | 100-104 |
| CCGGT | 15767000 | 2.561717 | 15.019526 | 9 |
| CCATC | 9164300 | 2.4946697 | 15.816875 | 50-54 |
| GTAAA | 5173505 | 2.492888 | 15.040603 | 95-99 |
| CAAGA | 6779155 | 2.465482 | 9.714812 | 115-119 |
| CAAAC | 6175645 | 2.4629776 | 11.633084 | 90-94 |
| CTTAA | 4889855 | 2.4571369 | 15.609432 | 40-44 |
| TTTTC | 5405805 | 2.456523 | 19.294922 | 35-39 |
| GGCCA | 14360845 | 2.4535704 | 6.5696516 | 75-79 |
| TCTTT | 5375895 | 2.4429314 | 14.237716 | 30-34 |
| CAGGT | 10781980 | 2.4406672 | 16.369726 | 2 |
| GCTGG | 16416200 | 2.4322202 | 8.740802 | 75-79 |
| ACACA | 6092895 | 2.4299753 | 6.072088 | 8 |
| CGGTA | 10686145 | 2.4189734 | 9.924197 | 95-99 |
| TAAAG | 4971350 | 2.3954785 | 14.927948 | 95-99 |
| ACGCT | 9613340 | 2.3863614 | 8.779325 | 105-109 |
| CGACC | 12712480 | 2.3817744 | 5.157406 | 50-54 |
| CGTAT | 7236600 | 2.380061 | 9.6367035 | 85-89 |
| TCTTA | 4948550 | 2.3646934 | 21.975544 | 40-44 |
| AGATC | 6778785 | 2.3444543 | 10.243999 | 115-119 |
| AAAAT | 3177200 | 2.3390648 | 10.823682 | 125-129 |
| CTTTT | 5129705 | 2.331057 | 16.369799 | 35-39 |
| TTAAC | 4638275 | 2.3307183 | 15.3790045 | 40-44 |
| GCTTC | 9872150 | 2.3304362 | 8.688004 | 110-114 |
| AAAGC | 6389985 | 2.3239465 | 13.16127 | 100-104 |
| CCTTC | 8973310 | 2.322897 | 10.599978 | 70-74 |
| ACCCT | 8373560 | 2.2794175 | 12.903774 | 55-59 |
| AGCGC | 13207270 | 2.2564805 | 8.3028345 | 100-104 |
| CACAC | 7859995 | 2.2499478 | 20.80043 | 8 |
| TGGAA | 7081545 | 2.2333977 | 14.079364 | 60-64 |
| TCTCA | 6164520 | 2.2233331 | 8.262368 | 70-74 |
| CGCGA | 12940780 | 2.2109501 | 9.107159 | 40-44 |
| TGACC | 8875275 | 2.2031484 | 6.954445 | 80-84 |
| CGGTG | 14850920 | 2.2003086 | 7.928727 | 125-129 |
| ACTTC | 6083245 | 2.19402 | 8.195209 | 25-29 |
| ATGTC | 6663515 | 2.1915777 | 8.108483 | 30-34 |
| CAATC | 5761875 | 2.1852725 | 14.61199 | 100-104 |
| CCGCG | 17793075 | 2.1819336 | 5.740732 | 85-89 |
| CGAAT | 6263270 | 2.1661625 | 13.878036 | 45-49 |
| CGCTT | 9162520 | 2.16292 | 7.3533616 | 105-109 |
| ATCAC | 5687110 | 2.1569166 | 11.924064 | 50-54 |
| TTCGA | 6551425 | 2.1547122 | 18.970009 | 3 |
| GAAGC | 9047600 | 2.1536736 | 8.686165 | 3 |
| GCATT | 6531685 | 2.1482196 | 7.5110745 | 65-69 |
| GCAAC | 8226540 | 2.1474128 | 27.494728 | 2 |
| GGAGG | 15008215 | 2.132278 | 7.552735 | 8 |
| GGTGC | 14343945 | 2.125195 | 11.880225 | 4 |
| CGTAG | 9387805 | 2.1250744 | 10.37824 | 60-64 |
| GAAGA | 6344260 | 2.104047 | 12.375252 | 25-29 |
| GAGGA | 9653285 | 2.0954134 | 5.481608 | 25-29 |
| GGAAT | 6636225 | 2.0929513 | 18.38943 | 60-64 |
| ACTCC | 7669785 | 2.0878386 | 25.881788 | 5 |
| TGCAT | 6329960 | 2.081874 | 10.821871 | 85-89 |
| GTTGC | 9665980 | 2.080748 | 11.797798 | 85-89 |
| GGGTT | 10581970 | 2.0772476 | 8.10653 | 85-89 |
| TGAAA | 4291075 | 2.067683 | 7.718497 | 120-124 |
| GCGTA | 9130295 | 2.066783 | 8.206182 | 80-84 |
| TTGCA | 6252320 | 2.0563388 | 15.417014 | 85-89 |
| TGGAG | 9949185 | 2.0537412 | 9.660387 | 75-79 |
| AGGCG | 13125975 | 2.0450227 | 6.6036897 | 4 |
| ACGAA | 5576900 | 2.028239 | 16.412178 | 10-14 |
| CTCTA | 5585630 | 2.0145473 | 15.641313 | 135-137 |
| TTGTA | 4598120 | 2.003666 | 9.230325 | 125-129 |
| GTATC | 6081060 | 2.0000126 | 12.825364 | 85-89 |
| ATCAG | 5726650 | 1.9805715 | 12.374932 | 65-69 |
| ACGTG | 8710830 | 1.9718305 | 13.040044 | 7 |
| CTGGC | 12130690 | 1.9709134 | 7.606614 | 95-99 |
| TGGTG | 9987100 | 1.9604744 | 10.6391735 | 115-119 |
| TAGCC | 7882405 | 1.9566839 | 17.89865 | 9 |
| TCTAC | 5412665 | 1.9521649 | 25.234682 | 135-137 |
| CGTTC | 8182420 | 1.9315559 | 10.115067 | 130-134 |
| CCCTG | 10820180 | 1.9278286 | 6.399397 | 15-19 |
| AAACG | 5291440 | 1.9244213 | 13.904309 | 90-94 |
| GTAGG | 9316170 | 1.9230723 | 10.509899 | 80-84 |
| GCCCT | 10710025 | 1.9082023 | 5.2482686 | 35-39 |
| TCCGC | 10700495 | 1.9065042 | 7.895741 | 100-104 |
| GGCTG | 12789800 | 1.8949335 | 6.703226 | 70-74 |
| CTTGA | 5761515 | 1.8949169 | 13.049152 | 135-137 |
| GCCGG | 16823925 | 1.8813344 | 8.831661 | 8 |
| AATAT | 2686535 | 1.8808485 | 5.933278 | 70-74 |
| AGGGA | 8591625 | 1.8649617 | 7.8242135 | 15-19 |
| GCCAT | 7499360 | 1.8615991 | 10.928287 | 50-54 |
| CAGGC | 10894625 | 1.8613617 | 7.534405 | 3 |
| TGAAT | 4053575 | 1.857461 | 8.820016 | 135-137 |
| TCGAC | 7471100 | 1.8545839 | 14.618744 | 4 |
| TTCTG | 5928115 | 1.8541024 | 14.94377 | 110-114 |
| GATCT | 5629975 | 1.8516543 | 12.552953 | 115-119 |
| TGTTA | 4235530 | 1.8456645 | 8.833166 | 105-109 |
| GAAAA | 3621335 | 1.8349448 | 5.4726453 | 120-124 |
| ACCGC | 9742070 | 1.8252468 | 6.393483 | 75-79 |
| CACCA | 6374365 | 1.8246816 | 18.593513 | 6 |
| GTATG | 6078685 | 1.8231031 | 11.677998 | 30-34 |
| GCCTT | 7670115 | 1.8106203 | 7.652108 | 70-74 |
| GTGGC | 12163160 | 1.8020906 | 7.476039 | 115-119 |
| GGCGA | 11498360 | 1.7914411 | 5.4106264 | 1 |
| AACGT | 5171855 | 1.7886947 | 6.8131094 | 80-84 |
| CAACG | 6835730 | 1.7843632 | 7.513671 | 125-129 |
| CGCGT | 10976715 | 1.7834234 | 5.05345 | 85-89 |
| GCAGG | 11399180 | 1.7759888 | 11.469221 | 1 |
| CCAAC | 6191960 | 1.7724676 | 6.6671133 | 85-89 |
| AAGAG | 5341665 | 1.7715406 | 14.346381 | 25-29 |
| TCAGG | 7792100 | 1.7638619 | 9.788205 | 65-69 |
| TAACG | 5098030 | 1.7631623 | 12.095276 | 45-49 |
| CGCCG | 14341030 | 1.7586155 | 11.19148 | 9 |
| TGTAG | 5806000 | 1.7413201 | 16.553078 | 7 |
| TCTCC | 6726410 | 1.7412483 | 7.6760645 | 50-54 |
| CGGAG | 11036545 | 1.7194904 | 18.509617 | 3 |
| CTCCC | 8770465 | 1.7135957 | 22.365343 | 6 |
| GGTAT | 5695970 | 1.7083201 | 8.645937 | 30-34 |
| CACGC | 9095790 | 1.7041616 | 8.84064 | 105-109 |
| CTGTT | 5422860 | 1.6960765 | 6.7858624 | 135-137 |
| CGCCA | 9009335 | 1.6879638 | 7.009736 | 105-109 |
| ATATT | 2524610 | 1.6808121 | 5.8363147 | 70-74 |
| GAGGT | 8141825 | 1.6806602 | 6.535274 | 75-79 |
| CCCTC | 8589070 | 1.6781545 | 5.339493 | 35-39 |
| AGGTA | 5319775 | 1.6777655 | 9.831404 | 75-79 |
| GACGT | 7347010 | 1.6631088 | 12.9231415 | 6 |
| GGTAA | 5269410 | 1.6618812 | 14.865281 | 95-99 |
| AACGC | 6311730 | 1.6475809 | 7.09899 | 95-99 |
| AGGGT | 7976235 | 1.6464787 | 6.8401256 | 80-84 |
| TCTTG | 5247690 | 1.6412897 | 7.1981854 | 120-124 |
| ATCCG | 6504515 | 1.6146442 | 6.141295 | 105-109 |
| CTCAC | 5894510 | 1.6045803 | 5.44379 | 110-114 |
| GTAGC | 7046045 | 1.5949808 | 12.308454 | 8 |
| AGTTC | 4847010 | 1.5941433 | 10.360254 | 25-29 |
| GACCC | 8457410 | 1.5845566 | 9.052824 | 15-19 |
| CGGCC | 12878250 | 1.5792373 | 5.1063046 | 70-74 |
| AAGTT | 3425780 | 1.5697875 | 7.8700376 | 5 |
| GGTGA | 7595825 | 1.5679532 | 7.0958366 | 75-79 |
| CCACG | 8341290 | 1.5628004 | 11.992673 | 8 |
| CTTCC | 6002560 | 1.5538671 | 6.0082927 | 60-64 |
| AAATC | 2925615 | 1.5459195 | 9.045277 | 125-129 |
| AGAGT | 4901070 | 1.5457133 | 14.588521 | 25-29 |
| CCCGC | 11461345 | 1.5412673 | 8.029258 | 9 |
| CGCAT | 6201160 | 1.5393412 | 5.635799 | 65-69 |
| TCCCG | 8570455 | 1.5269955 | 17.10175 | 7 |
| GTTCA | 4613350 | 1.5172945 | 5.6699214 | 1 |
| GGCGT | 10203770 | 1.5117878 | 6.551564 | 1 |
| GGAAG | 6958655 | 1.5104971 | 12.928254 | 2 |
| TTCAG | 4563375 | 1.5008581 | 6.391335 | 20-24 |
| CCGTA | 5956630 | 1.4786403 | 7.0509167 | 60-64 |
| TACGG | 6519960 | 1.4758934 | 14.298712 | 135-137 |
| GCAAG | 6140945 | 1.4617789 | 6.567207 | 115-119 |
| CCTGG | 8917680 | 1.4488851 | 6.22686 | 20-24 |
| ACGCC | 7691715 | 1.4410981 | 16.693136 | 8 |
| CATGC | 5792540 | 1.4379076 | 8.926612 | 90-94 |
| GTGCC | 8802030 | 1.430095 | 9.611948 | 9 |
| AGGAC | 5979600 | 1.4233726 | 5.515407 | 25-29 |
| ACCAC | 4966105 | 1.4215629 | 18.474253 | 7 |
| GCATG | 6263075 | 1.4177434 | 7.235873 | 85-89 |
| TCTGG | 6553735 | 1.4107903 | 7.820902 | 110-114 |
| AATGG | 4467595 | 1.4090029 | 6.284333 | 135-137 |
| AGGCA | 5913765 | 1.4077014 | 6.185102 | 15-19 |
| GAGGG | 9842845 | 1.3984128 | 7.366574 | 10-14 |
| TGCCC | 7819515 | 1.3932008 | 8.308515 | 90-94 |
| GCGTC | 8570230 | 1.3924336 | 6.9777546 | 6 |
| CTTGT | 4450480 | 1.3919508 | 9.3413315 | 120-124 |
| AAGGG | 6408860 | 1.3911545 | 7.5460978 | 5 |
| ACACC | 4859210 | 1.3909638 | 20.572453 | 9 |
| ATGCC | 5564375 | 1.3812691 | 10.662571 | 90-94 |
| CCGGA | 8071205 | 1.3789766 | 12.508323 | 9 |
| GGAGA | 6345380 | 1.3773751 | 19.631485 | 4 |
| TTGGC | 6385015 | 1.3744708 | 9.222658 | 70-74 |
| CGGCA | 8040420 | 1.373717 | 5.381669 | 5 |
| GTGAC | 6057075 | 1.3711122 | 8.794578 | 75-79 |
| TAATA | 1955800 | 1.3692595 | 7.2347875 | 70-74 |
| GAAGG | 6241370 | 1.354798 | 7.660234 | 4 |
| GGCCT | 8287035 | 1.3464221 | 5.3500724 | 65-69 |
| GCGTT | 6247125 | 1.344788 | 7.7108135 | 125-129 |
| AACGG | 5638215 | 1.34211 | 10.052215 | 45-49 |
| GTGCA | 5921625 | 1.340451 | 16.334398 | 5 |
| GAGTT | 4468390 | 1.3401476 | 9.397674 | 25-29 |
| GTCGC | 8245895 | 1.3397379 | 9.484509 | 8 |
| TCGCA | 5390995 | 1.3382303 | 5.368664 | 35-39 |
| TTAAT | 2006755 | 1.3360392 | 6.83417 | 70-74 |
| TGGCT | 6166580 | 1.3274494 | 10.283057 | 70-74 |
| GCACA | 5082050 | 1.3265917 | 18.949768 | 7 |
| GACGA | 5565305 | 1.3247546 | 5.2203593 | 130-134 |
| GGTGG | 9798125 | 1.3237966 | 8.490432 | 115-119 |
| CTCCG | 7395655 | 1.3176817 | 6.889106 | 50-54 |
| AGACG | 5524445 | 1.3150285 | 20.918356 | 6 |
| TCCGG | 8052000 | 1.3082352 | 7.498159 | 120-124 |
| ATGTA | 2854655 | 1.3080823 | 21.758398 | 6 |
| TAGAC | 3769240 | 1.303598 | 7.769788 | 125-129 |
| ATTTT | 2058815 | 1.3034841 | 6.133108 | 135-137 |
| GGTAG | 6266855 | 1.2936231 | 7.7071695 | 80-84 |
| GTTTC | 4134225 | 1.2930375 | 19.008713 | 1 |
| ATATG | 2813075 | 1.2890292 | 21.195177 | 4 |
| TCGGA | 5689145 | 1.2878256 | 24.30259 | 2 |
| TCCGT | 5418220 | 1.279034 | 6.097755 | 55-59 |
| CGTCC | 7174580 | 1.278293 | 8.347504 | 120-124 |
| GTTAC | 3878535 | 1.2756196 | 6.3803873 | 110-114 |
| GTAGA | 4042600 | 1.2749666 | 5.9909244 | 105-109 |
| GGGAA | 5818925 | 1.263099 | 12.603733 | 1 |
| CGACT | 5078035 | 1.2605429 | 5.3937383 | 130-134 |
| CTGGT | 5852600 | 1.2598605 | 6.9866004 | 110-114 |
| TTACG | 3809895 | 1.2530444 | 6.759259 | 110-114 |
| TGTCG | 5793930 | 1.2472309 | 5.2716885 | 15-19 |
| TGGGA | 6007525 | 1.2400917 | 9.772908 | 20-24 |
| GCGAC | 7204355 | 1.2308741 | 5.11005 | 115-119 |
| TATAG | 2674465 | 1.2255142 | 11.409268 | 7 |
| TAGGC | 5393905 | 1.2209935 | 5.3568163 | 65-69 |
| TAGGG | 5906200 | 1.2191759 | 9.567554 | 80-84 |
| CCCGG | 9886295 | 1.2123389 | 11.39814 | 8 |
| CACCC | 5850945 | 1.202121 | 8.803668 | 55-59 |
| TGCAC | 4836695 | 1.200634 | 17.684156 | 6 |
| ACGGC | 6944020 | 1.1863954 | 8.345361 | 135-137 |
| TCGGT | 5457020 | 1.1747059 | 5.267998 | 75-79 |
| TTTCG | 3727120 | 1.1657099 | 18.027624 | 2 |
| GACTA | 3366775 | 1.1644049 | 7.8633466 | 115-119 |
| ACTTG | 3499835 | 1.1510681 | 8.296484 | 130-134 |
| GTTGG | 5846090 | 1.1475914 | 7.0345187 | 70-74 |
| ACATT | 2249515 | 1.1303741 | 11.124445 | 2 |
| GTATA | 2465990 | 1.1299853 | 11.2843485 | 6 |
| CTACG | 4475790 | 1.1110449 | 12.197602 | 135-137 |
| GAGGC | 7103320 | 1.1066952 | 5.313821 | 9 |
| GAAGT | 3509000 | 1.1066784 | 6.7348886 | 4 |
| TGCCG | 6794125 | 1.103864 | 5.0859895 | 2 |
| GGGAC | 7055155 | 1.0991911 | 5.794287 | 15-19 |
| CACGA | 4187500 | 1.093083 | 16.4519 | 9 |
| GGCAC | 6345100 | 1.084069 | 5.4331665 | 6 |
| ACGAC | 4139685 | 1.0806017 | 5.2997165 | 130-134 |
| CACAT | 2789295 | 1.0578793 | 8.897096 | 1 |
| GGTTT | 3662360 | 1.0445427 | 10.7523365 | 3 |
| GCCAC | 5520495 | 1.0343045 | 6.8842745 | 105-109 |
| TAAAT | 1471265 | 1.0300356 | 5.2320175 | 3 |
| CTCGG | 6247445 | 1.0150431 | 15.228875 | 1 |
| TGCGT | 4710120 | 1.0139245 | 7.443445 | 125-129 |
| ATCTT | 2120175 | 1.0131378 | 5.084968 | 30-34 |
| CATAT | 1997770 | 1.003873 | 23.317207 | 3 |
| GTCCG | 6177565 | 1.0036894 | 8.077747 | 120-124 |
| AGGTG | 4811490 | 0.99320227 | 14.823744 | 3 |
| AGTTA | 2156735 | 0.9882759 | 7.1238866 | 6 |
| AGCCA | 3729455 | 0.97351736 | 5.315391 | 10-14 |
| CGTGC | 5921670 | 0.96211344 | 9.479504 | 8 |
| AGCTC | 3858020 | 0.95769316 | 5.753054 | 4 |
| AATTG | 2087855 | 0.9567133 | 7.464275 | 5 |
| ATAGC | 2752945 | 0.95211065 | 9.1378145 | 8 |
| GAGCG | 6095480 | 0.9496739 | 5.0547853 | 135-137 |
| TCCCT | 3628705 | 0.9393533 | 5.851057 | 7 |
| AAGCT | 2704815 | 0.93546486 | 7.9350376 | 3 |
| GTGCG | 6301090 | 0.9335679 | 8.355254 | 125-129 |
| AAGCA | 2560745 | 0.9313066 | 9.211205 | 4 |
| GCACG | 5369575 | 0.91739935 | 5.353402 | 7 |
| GGGAG | 6305075 | 0.89578754 | 5.537968 | 15-19 |
| GGACC | 5236130 | 0.8946 | 7.374715 | 15-19 |
| TCGCC | 5014390 | 0.8934124 | 7.6503177 | 9 |
| ACGTA | 2542770 | 0.8794213 | 8.36268 | 4 |
| ACTTT | 1833475 | 0.8761366 | 6.6576447 | 7 |
| GCCAG | 5119700 | 0.8747078 | 7.95999 | 1 |
| TCGCG | 5377720 | 0.873736 | 6.159582 | 35-39 |
| CGACG | 5047740 | 0.8624134 | 9.967062 | 5 |
| CTGCA | 3449065 | 0.8561765 | 5.443786 | 3 |
| GAGAC | 3533225 | 0.84104216 | 21.104902 | 5 |
| CGTCG | 4995280 | 0.8115998 | 7.08876 | 7 |
| ATTGT | 1795985 | 0.78261423 | 6.1630626 | 6 |
| AGCAT | 2200235 | 0.760955 | 15.993277 | 1 |
| TTCCC | 2889750 | 0.7480621 | 6.093291 | 6 |
| ACGGG | 4796950 | 0.7473634 | 7.135731 | 45-49 |
| TTTCC | 2101795 | 0.7208739 | 7.9631147 | 5 |
| GAATT | 1560080 | 0.714872 | 6.8722024 | 4 |
| CTGGG | 4540635 | 0.6727393 | 6.3504643 | 20-24 |
| CCAGG | 3877035 | 0.6623968 | 7.3667502 | 2 |
| CCTGT | 2688140 | 0.6345668 | 7.9456716 | 9 |
| GTCGA | 2725725 | 0.6170098 | 5.4767566 | 1 |
| GCATA | 1632885 | 0.564736 | 15.82212 | 2 |
| GACGC | 3274805 | 0.559505 | 15.095245 | 7 |
| CCCCG | 3915375 | 0.526521 | 7.136438 | 8 |

Produced by FastQC (version 0.10.1)
