# Supplementary material for: Freshwater sponge hosts and their green algae symbionts: a tractable model to understand intracellular symbiosis
Source: PeerJ. 2021 Feb 11;9:e10654. doi: 10.7717/peerj.10654 (PMC7882143; doi:10.7717/peerj.10654)
Supplement: Supplemental Information 26 [file peerj-09-10654-s026.zip › EmApo2_Clean_Data1.fq_fastqc/fastqc_report.html]

EmApo2\_Clean\_Data1.fq.gz FastQC Report


FastQC Report

Tue 10 Sep 2019  
EmApo2\_Clean\_Data1.fq.gz

## Summary

- Basic Statistics
- Per base sequence quality
- Per sequence quality scores
- Per base sequence content
- Per base GC content
- Per sequence GC content
- Per base N content
- Sequence Length Distribution
- Sequence Duplication Levels
- Overrepresented sequences
- Kmer Content

## Basic Statistics

| Measure | Value |
| --- | --- |
| Filename | EmApo2\_Clean\_Data1.fq.gz |
| File type | Conventional base calls |
| Encoding | Sanger / Illumina 1.9 |
| Total Sequences | 33150512 |
| Filtered Sequences | 0 |
| Sequence length | 100-141 |
| %GC | 58 |

## Per base sequence quality

## Per sequence quality scores

## Per base sequence content

## Per base GC content

## Per sequence GC content

## Per base N content

## Sequence Length Distribution

## Sequence Duplication Levels

## Overrepresented sequences

| Sequence | Count | Percentage | Possible Source |
| --- | --- | --- | --- |
| GTCCCATTCAAGTCGTCTACAAGAGATCTTGCCCCGCGGATTGGCCAGCG | 1518612 | 4.5809609215085425 | No Hit |
| GCGAGAAAATGAACCGCTCCCTCGGATTTTCAAGGGCCGTAGAGAACGCA | 1064870 | 3.2122279137046212 | No Hit |
| AGAAAATGAACCGCTCCCTCGGATTTTCAAGGGCCGTAGAGAACGCACCG | 644241 | 1.9433817492773564 | No Hit |
| GAGAAAATGAACCGCTCCCTCGGATTTTCAAGGGCCGTAGAGAACGCACC | 637129 | 1.921928083644681 | No Hit |
| GCCGTTAGTCGCCTGCCGAATAGCCGCCGACCACGAGGGACGGCGACCAA | 511704 | 1.5435779694744987 | No Hit |
| GCCACCTACAGCCAACAGTCTGAAGCGCAGTCGCGAACCCCGCGCACGGC | 509951 | 1.5382899666828675 | No Hit |
| GTCGTCTACAAGAGATCTTGCCCCGCGGATTGGCCAGCGTTTGATACGCG | 485362 | 1.4641161500009412 | No Hit |
| GGCGAGAAAATGAACCGCTCCCTCGGATTTTCAAGGGCCGTAGAGAACGC | 413504 | 1.2473532837139891 | No Hit |
| AAGAGATCTTGCCCCGCGGATTGGCCAGCGTTTGATACGCGCGGTCACCG | 390263 | 1.1772457692357814 | No Hit |
| CTGCGCTGGCGGGTCGAAGAGACCCTCTCCTCGGTCGCGGGCGCGCTCCG | 354104 | 1.0681705308201574 | No Hit |
| GTCGCCGTAACAGCACCGCCCGCAACCCACGTTGGCCAGCCCCGGTGAGA | 322542 | 0.9729623482135057 | No Hit |
| CGGGCGAGAAAATGAACCGCTCCCTCGGATTTTCAAGGGCCGTAGAGAAC | 314461 | 0.9485856508038247 | No Hit |
| ATTCAAGTCGTCTACAAGAGATCTTGCCCCGCGGATTGGCCAGCGTTTGA | 304497 | 0.918528799796516 | No Hit |
| GGCCGTTAGTCGCCTGCCGAATAGCCGCCGACCACGAGGGACGGCGACCA | 285583 | 0.861473874068672 | No Hit |
| AGCGCAGTCGCGAACCCCGCGCACGGCGGAGGGATGCGCCGGCCTCGCAC | 256032 | 0.7723319627763214 | No Hit |
| GAAAATGAACCGCTCCCTCGGATTTTCAAGGGCCGTAGAGAACGCACCGG | 241483 | 0.7284442544959788 | No Hit |
| CTCTCCTCGGTCGCGGGCGCGCTCCGAACGACGCGGCTATACGTCCCTAA | 225930 | 0.6815279353754777 | No Hit |
| CTCCTCGGTCGCGGGCGCGCTCCGAACGACGCGGCTATACGTCCCTAACT | 182221 | 0.5496777847654358 | No Hit |
| GCGCTGGCGGGTCGAAGAGACCCTCTCCTCGGTCGCGGGCGCGCTCCGAA | 178176 | 0.5374758616096186 | No Hit |
| GATGAAGCCACCTACAGCCAACAGTCTGAAGCGCAGTCGCGAACCCCGCG | 174771 | 0.5272045270371691 | No Hit |
| GTCTACAAGAGATCTTGCCCCGCGGATTGGCCAGCGTTTGATACGCGCGG | 170838 | 0.5153404568834412 | No Hit |
| GCAGAAATTTGAATGCACCATCGCCGGCACGAGGCCATGCGATTCGAGCA | 170609 | 0.5146496681559549 | No Hit |
| GAGATCTTGCCCCGCGGATTGGCCAGCGTTTGATACGCGCGGTCACCGAA | 164020 | 0.49477365538125023 | No Hit |
| AGAGATCTTGCCCCGCGGATTGGCCAGCGTTTGATACGCGCGGTCACCGA | 161363 | 0.48675869621561196 | No Hit |
| GTCGGCCGTTAGTCGCCTGCCGAATAGCCGCCGACCACGAGGGACGGCGA | 160700 | 0.48475872710502926 | No Hit |
| GGGCGAGAAAATGAACCGCTCCCTCGGATTTTCAAGGGCCGTAGAGAACG | 152544 | 0.4601557888457348 | No Hit |
| GTCAGATGAAGCCACCTACAGCCAACAGTCTGAAGCGCAGTCGCGAACCC | 149686 | 0.4515345042031327 | No Hit |
| AGAAATTTGAATGCACCATCGCCGGCACGAGGCCATGCGATTCGAGCAGT | 141585 | 0.4270974759002214 | No Hit |
| CTCGTCCCATTCAAGTCGTCTACAAGAGATCTTGCCCCGCGGATTGGCCA | 130665 | 0.3941568081965069 | No Hit |
| GCCCGCAACCCACGTTGGCCAGCCCCGGTGAGAAATGCGGAAGCGGCGGT | 123761 | 0.3733305838534259 | No Hit |
| CCTGACTCTCCAAAGACACCTAATATCTAGGCAGGCGGTCGGCCGCGTAC | 118792 | 0.35834137343037115 | No Hit |
| CGAGAAAATGAACCGCTCCCTCGGATTTTCAAGGGCCGTAGAGAACGCAC | 116096 | 0.35020876902293396 | No Hit |
| CAAGTCGTCTACAAGAGATCTTGCCCCGCGGATTGGCCAGCGTTTGATAC | 108050 | 0.32593765067640584 | No Hit |
| CTTATATTGGTCGGGCTAGGAGCTGAGTCTACTCACAGGCACTATCCCAT | 104060 | 0.3139016374769717 | No Hit |
| CCGCCCGCAACCCACGTTGGCCAGCCCCGGTGAGAAATGCGGAAGCGGCG | 101945 | 0.30752164551787314 | No Hit |
| CAAGAGATCTTGCCCCGCGGATTGGCCAGCGTTTGATACGCGCGGTCACC | 101488 | 0.306143084607562 | No Hit |
| GAAATTTGAATGCACCATCGCCGGCACGAGGCCATGCGATTCGAGCAGTT | 97297 | 0.2935007459311639 | No Hit |
| GCGGGAGCTCCGGCCACGAAGGCCTGCGCTGGCGGGTCGAAGAGACCCTC | 97032 | 0.29270136159586313 | No Hit |
| CCCGCAACCCACGTTGGCCAGCCCCGGTGAGAAATGCGGAAGCGGCGGTC | 94888 | 0.2862338898415807 | No Hit |
| GCCTGCGCTGGCGGGTCGAAGAGACCCTCTCCTCGGTCGCGGGCGCGCTC | 94371 | 0.2846743362515789 | No Hit |
| CCGCAACCCACGTTGGCCAGCCCCGGTGAGAAATGCGGAAGCGGCGGTCG | 93822 | 0.2830182532324086 | No Hit |
| CATTCAAGTCGTCTACAAGAGATCTTGCCCCGCGGATTGGCCAGCGTTTG | 92752 | 0.2797905504445904 | No Hit |
| TTCAAGTCGTCTACAAGAGATCTTGCCCCGCGGATTGGCCAGCGTTTGAT | 88439 | 0.2667801933194878 | No Hit |
| CGCGGATTGGCCAGCGTTTGATACGCGCGGTCACCGAAGGCCGCCTACGG | 87750 | 0.2647017940477058 | No Hit |
| GTTAGTCGCCTGCCGAATAGCCGCCGACCACGAGGGACGGCGACCAAGCT | 86532 | 0.2610276426499838 | No Hit |
| GCGCAGTCGCGAACCCCGCGCACGGCGGAGGGATGCGCCGGCCTCGCACT | 84553 | 0.2550579007648509 | No Hit |
| CCCATTCAAGTCGTCTACAAGAGATCTTGCCCCGCGGATTGGCCAGCGTT | 83112 | 0.25071105990761167 | No Hit |
| AAAATGAACCGCTCCCTCGGATTTTCAAGGGCCGTAGAGAACGCACCGGA | 82288 | 0.24822542710652554 | No Hit |
| CACCCGGTCGCCGTAACAGCACCGCCCGCAACCCACGTTGGCCAGCCCCG | 81210 | 0.24497359196141524 | No Hit |
| GCCGTAACAGCACCGCCCGCAACCCACGTTGGCCAGCCCCGGTGAGAAAT | 79927 | 0.24110336516069497 | No Hit |
| CCTGCGCTGGCGGGTCGAAGAGACCCTCTCCTCGGTCGCGGGCGCGCTCC | 79854 | 0.24088315740040453 | No Hit |
| GAAGCCACCTACAGCCAACAGTCTGAAGCGCAGTCGCGAACCCCGCGCAC | 78444 | 0.23662982942767216 | No Hit |
| GGCAGAAATTTGAATGCACCATCGCCGGCACGAGGCCATGCGATTCGAGC | 78307 | 0.23621656280904502 | No Hit |
| CGTTAGTCGCCTGCCGAATAGCCGCCGACCACGAGGGACGGCGACCAAGC | 77580 | 0.23402353484012553 | No Hit |
| AGATCTTGCCCCGCGGATTGGCCAGCGTTTGATACGCGCGGTCACCGAAG | 71772 | 0.21650344344606198 | No Hit |
| GCCAACAGTCTGAAGCGCAGTCGCGAACCCCGCGCACGGCGGAGGGATGC | 71556 | 0.21585186979917537 | No Hit |
| CCCGGTCGCCGTAACAGCACCGCCCGCAACCCACGTTGGCCAGCCCCGGT | 68979 | 0.20807823420645813 | No Hit |
| GGCGGGTCGAAGAGACCCTCTCCTCGGTCGCGGGCGCGCTCCGAACGACG | 68136 | 0.20553528705680324 | No Hit |
| CGAGATGGCGCCCTCCACCGGAACGCGGGAGCTCCGGCCACGAAGGCCTG | 67953 | 0.20498325938374645 | No Hit |
| TGACTCTCCAAAGACACCTAATATCTAGGCAGGCGGTCGGCCGCGTACGG | 66595 | 0.20088679173341276 | No Hit |
| GGCCTGCGCTGGCGGGTCGAAGAGACCCTCTCCTCGGTCGCGGGCGCGCT | 66382 | 0.20024426772051063 | No Hit |
| CACCTACAGCCAACAGTCTGAAGCGCAGTCGCGAACCCCGCGCACGGCGG | 63096 | 0.19033190196278113 | No Hit |
| CTGCTTACAACACCTCGTCCCATTCAAGTCGTCTACAAGAGATCTTGCCC | 60943 | 0.18383728130654514 | No Hit |
| CTCCACCGGAACGCGGGAGCTCCGGCCACGAAGGCCTGCGCTGGCGGGTC | 60171 | 0.18150850882785763 | No Hit |
| CTACAAGAGATCTTGCCCCGCGGATTGGCCAGCGTTTGATACGCGCGGTC | 59617 | 0.1798373430853798 | No Hit |
| CCGGTCGCCGTAACAGCACCGCCCGCAACCCACGTTGGCCAGCCCCGGTG | 59464 | 0.1793758117521684 | No Hit |
| GCCACGAAGGCCTGCGCTGGCGGGTCGAAGAGACCCTCTCCTCGGTCGCG | 58120 | 0.17532157572709586 | No Hit |
| CTCCAAAGACACCTAATATCTAGGCAGGCGGTCGGCCGCGTACGGGGTTC | 58010 | 0.17498975581432952 | No Hit |
| AAGCGCAGTCGCGAACCCCGCGCACGGCGGAGGGATGCGCCGGCCTCGCA | 57995 | 0.1749445076444068 | No Hit |
| AGATGAAGCCACCTACAGCCAACAGTCTGAAGCGCAGTCGCGAACCCCGC | 57666 | 0.17395206445076927 | No Hit |
| GCCCACTGGTGTTAGTTTTAGTACAGCCGAGCCCAATTTATTGGGCTGAA | 54932 | 0.1657048313461946 | No Hit |
| GCTCCCTCGGATTTTCAAGGGCCGTAGAGAACGCACCGGACGCCACCAGA | 54455 | 0.1642659395426532 | No Hit |
| GATCTTGCCCCGCGGATTGGCCAGCGTTTGATACGCGCGGTCACCGAAGG | 53701 | 0.16199146486787294 | No Hit |
| CCGAGATGGCGCCCTCCACCGGAACGCGGGAGCTCCGGCCACGAAGGCCT | 53592 | 0.1616626614997681 | No Hit |
| GTCTACTTATATTGGTCGGGCTAGGAGCTGAGTCTACTCACAGGCACTAT | 52272 | 0.1576808225465718 | No Hit |
| CCGGGCGAGAAAATGAACCGCTCCCTCGGATTTTCAAGGGCCGTAGAGAA | 51485 | 0.15530680189796164 | No Hit |
| CACCGGAACGCGGGAGCTCCGGCCACGAAGGCCTGCGCTGGCGGGTCGAA | 50980 | 0.15378344684389791 | No Hit |
| GCTTACAACACCTCGTCCCATTCAAGTCGTCTACAAGAGATCTTGCCCCG | 50711 | 0.1529719963299511 | No Hit |
| CTCGGTCGCGGGCGCGCTCCGAACGACGCGGCTATACGTCCCTAACTTCG | 48802 | 0.14721341257112408 | No Hit |
| CGCCGTAACAGCACCGCCCGCAACCCACGTTGGCCAGCCCCGGTGAGAAA | 48718 | 0.14696002281955706 | No Hit |
| AAATGAACCGCTCCCTCGGATTTTCAAGGGCCGTAGAGAACGCACCGGAC | 48293 | 0.1456779913384143 | No Hit |
| ACCGCCCGCAACCCACGTTGGCCAGCCCCGGTGAGAAATGCGGAAGCGGC | 48066 | 0.14499323570025102 | No Hit |
| GTCGGGCTAGGAGCTGAGTCTACTCACAGGCACTATCCCATTACCGCCTG | 45644 | 0.1376871645300682 | No Hit |
| CTCGGATTTTCAAGGGCCGTAGAGAACGCACCGGACGCCACCAGAAGCGT | 44565 | 0.1344323128402964 | No Hit |
| CTACTGCTTACAACACCTCGTCCCATTCAAGTCGTCTACAAGAGATCTTG | 44426 | 0.1340130131323462 | No Hit |
| GCGCATATGTAGCCCAAAACATTAGGATCATAAGGACCTGACGTCATCCT | 43028 | 0.12979588369555198 | No Hit |
| CAGATGAAGCCACCTACAGCCAACAGTCTGAAGCGCAGTCGCGAACCCCG | 42700 | 0.12880645704657592 | No Hit |
| CTCGTCCCGGTTCGGGAATATTAACCCGATTCCCTTTCGATGGTGGGTGC | 42411 | 0.12793467563939886 | No Hit |
| CCATTCAAGTCGTCTACAAGAGATCTTGCCCCGCGGATTGGCCAGCGTTT | 41993 | 0.12667375997088673 | No Hit |
| GTCCGAGATGGCGCCCTCCACCGGAACGCGGGAGCTCCGGCCACGAAGGC | 41901 | 0.12639623786202758 | No Hit |
| CCGTTAGTCGCCTGCCGAATAGCCGCCGACCACGAGGGACGGCGACCAAG | 41036 | 0.12378692672981943 | No Hit |
| ACAAGAGATCTTGCCCCGCGGATTGGCCAGCGTTTGATACGCGCGGTCAC | 40759 | 0.12295134385858053 | No Hit |
| CAACAGTCTGAAGCGCAGTCGCGAACCCCGCGCACGGCGGAGGGATGCGC | 40420 | 0.12192873521832785 | No Hit |
| CCTCGGTCGCGGGCGCGCTCCGAACGACGCGGCTATACGTCCCTAACTTC | 40398 | 0.12186237123577458 | No Hit |
| CGCCCGCAACCCACGTTGGCCAGCCCCGGTGAGAAATGCGGAAGCGGCGG | 39805 | 0.12007356025149776 | No Hit |
| CAGAAATTTGAATGCACCATCGCCGGCACGAGGCCATGCGATTCGAGCAG | 39459 | 0.11902983579861451 | No Hit |
| CTACAGCCAACAGTCTGAAGCGCAGTCGCGAACCCCGCGCACGGCGGAGG | 39326 | 0.11862863535863338 | No Hit |
| ACCCGGTCGCCGTAACAGCACCGCCCGCAACCCACGTTGGCCAGCCCCGG | 39272 | 0.11846574194691172 | No Hit |
| GGCATTCGTTAAGCCATTCATACTTGCCAACAATTAATTGGCTATTGATT | 38823 | 0.11711131339389269 | No Hit |
| CATTATTCAACCTGGATACAGCCGGACTCCTCCGGCGAACCCATATTGAT | 38638 | 0.1165532526315129 | No Hit |
| GGTCGCCGTAACAGCACCGCCCGCAACCCACGTTGGCCAGCCCCGGTGAG | 38150 | 0.11508117883669489 | No Hit |
| GCACCGCCCGCAACCCACGTTGGCCAGCCCCGGTGAGAAATGCGGAAGCG | 37756 | 0.11389266024005903 | No Hit |
| CTCTACTGCTTACAACACCTCGTCCCATTCAAGTCGTCTACAAGAGATCT | 37240 | 0.11233612319471867 | No Hit |
| GCCAGCGTTTGATACGCGCGGTCACCGAAGGCCGCCTACGGGCCACGGAG | 36680 | 0.11064685818427179 | No Hit |
| CCGCGGATTGGCCAGCGTTTGATACGCGCGGTCACCGAAGGCCGCCTACG | 36634 | 0.11050809712984222 | No Hit |
| TACAAGAGATCTTGCCCCGCGGATTGGCCAGCGTTTGATACGCGCGGTCA | 36465 | 0.10999830108204664 | No Hit |
| CTGACTCTCCAAAGACACCTAATATCTAGGCAGGCGGTCGGCCGCGTACG | 36234 | 0.1093014792652373 | No Hit |
| CTCCAGCCAACCTGATTCCAGGGTGATGGCCCGTTAAGAAGAAAAGAGAA | 35855 | 0.10815820883852412 | No Hit |
| GGGAGCTCCGGCCACGAAGGCCTGCGCTGGCGGGTCGAAGAGACCCTCTC | 35280 | 0.10642369565815454 | No Hit |
| GTCCCGGTTCGGGAATATTAACCCGATTCCCTTTCGATGGTGGGTGCCGG | 35051 | 0.10573290693066822 | No Hit |
| GTTCTAAGTCGGCCGTTAGTCGCCTGCCGAATAGCCGCCGACCACGAGGG | 34901 | 0.10528042523144138 | No Hit |
| GTCGCCTGCCGAATAGCCGCCGACCACGAGGGACGGCGACCAAGCTGCGG | 34696 | 0.10466203357583136 | No Hit |
| GTAAATTAGACCATTAGTAATCTTTAGCTTAGAATAGAGGCGTGGCCTGC | 34536 | 0.10417938642998939 | No Hit |
| GGTCGGGCTAGGAGCTGAGTCTACTCACAGGCACTATCCCATTACCGCCT | 34468 | 0.10397426139300654 | No Hit |
| GGGAAATGTGTCGTTGCGTTCTAGCGTGGATTCTGACTTAGAGGCGTTCA | 34149 | 0.10301198364598411 | No Hit |

## Kmer Content

| Sequence | Count | Obs/Exp Overall | Obs/Exp Max | Max Obs/Exp Position |
| --- | --- | --- | --- | --- |
| GAGAT | 13986700 | 4.2863693 | 19.540195 | 3 |
| TTCAA | 10774605 | 4.2166796 | 90.58743 | 7 |
| ATTTT | 6092000 | 4.123585 | 39.544895 | 25-29 |
| AGAGA | 15016955 | 4.120452 | 18.750116 | 40-44 |
| AGAAA | 11808255 | 4.0794554 | 55.106262 | 4 |
| GATTC | 12463115 | 3.8738458 | 19.543917 | 110-114 |
| TTGAT | 7992240 | 3.8469706 | 24.794075 | 50-54 |
| GGATT | 10946680 | 3.7468648 | 29.008484 | 20-24 |
| TTTGA | 7680100 | 3.6967256 | 25.107298 | 50-54 |
| AAATG | 8944230 | 3.4512048 | 58.63131 | 7 |
| AAGAA | 9963555 | 3.4421577 | 49.16339 | 130-134 |
| GAGAA | 12503605 | 3.4308226 | 41.11764 | 3 |
| ATCTT | 7749950 | 3.387501 | 26.9721 | 6 |
| TTCGC | 15045055 | 3.3727667 | 15.701281 | 95-99 |
| TTTCA | 7352265 | 3.2136729 | 35.77856 | 25-29 |
| TTTTC | 6458735 | 3.153111 | 32.9659 | 25-29 |
| CTTCG | 14015640 | 3.1419942 | 12.356713 | 100-104 |
| ATTGG | 8750670 | 2.9952078 | 18.238869 | 40-44 |
| TGATA | 6890210 | 2.9694192 | 22.231478 | 50-54 |
| AGAAG | 10761685 | 2.952863 | 23.62735 | 130-134 |
| TGATT | 6041015 | 2.9077713 | 27.469429 | 110-114 |
| TTGCC | 12922735 | 2.8969896 | 12.973302 | 25-29 |
| GATTT | 5976035 | 2.876494 | 27.140467 | 25-29 |
| AGATT | 6662620 | 2.8713365 | 22.00551 | 90-94 |
| CGAAG | 14280025 | 2.8259773 | 11.88405 | 65-69 |
| AAAAT | 5815365 | 2.8252585 | 71.65764 | 6 |
| TTGGC | 11317620 | 2.7939422 | 14.109049 | 40-44 |
| ACCGG | 19529300 | 2.7874243 | 14.424446 | 75-79 |
| ATGAA | 7166165 | 2.7651236 | 57.33556 | 9 |
| CGCTT | 12270710 | 2.75082 | 18.255035 | 70-74 |
| TCCCT | 13485260 | 2.7452505 | 16.786983 | 15-19 |
| AGTTG | 8013430 | 2.7428627 | 17.063345 | 115-119 |
| AAGAG | 9863780 | 2.70649 | 15.250978 | 20-24 |
| GGCCA | 18942575 | 2.7036812 | 8.28021 | 40-44 |
| TGATG | 7897370 | 2.7031372 | 21.291794 | 120-124 |
| TTTAC | 6145175 | 2.6860542 | 25.936432 | 75-79 |
| GATGG | 11011515 | 2.6802046 | 18.08267 | 120-124 |
| GAGTT | 7798775 | 2.6693897 | 16.57865 | 115-119 |
| GAAAA | 7713265 | 2.6647391 | 51.043934 | 5 |
| GCGCT | 16710155 | 2.6638405 | 11.462138 | 70-74 |
| ATACG | 9484560 | 2.6395001 | 14.805096 | 55-59 |
| TCCAG | 13147125 | 2.6388333 | 16.197706 | 95-99 |
| TCGCG | 16517420 | 2.6331158 | 10.964518 | 95-99 |
| TACCG | 13031515 | 2.6156285 | 18.57403 | 75-79 |
| GTTTG | 6839070 | 2.614532 | 19.197588 | 50-54 |
| TCTTG | 7518040 | 2.6099472 | 20.540752 | 7 |
| CTTTA | 5903690 | 2.5805013 | 24.459713 | 75-79 |
| TCAAG | 9214060 | 2.5642214 | 63.76028 | 8 |
| ACATA | 7255560 | 2.5423121 | 18.48749 | 105-109 |
| GCTTT | 7151885 | 2.4828336 | 26.614378 | 70-74 |
| CAACC | 15195660 | 2.479817 | 12.777304 | 85-89 |
| GAAGA | 9037490 | 2.4797666 | 25.656206 | 135-137 |
| CGGTC | 15473100 | 2.466636 | 11.05934 | 60-64 |
| TTAAG | 5637465 | 2.4295335 | 27.922665 | 130-134 |
| AGCGT | 10873130 | 2.4032867 | 13.414046 | 65-69 |
| GATCT | 7689330 | 2.390035 | 18.912872 | 5 |
| GAAGG | 10895085 | 2.374325 | 11.463319 | 70-74 |
| TTCCA | 8321665 | 2.3488545 | 23.877787 | 110-114 |
| AATGA | 6055545 | 2.3365817 | 56.935158 | 8 |
| CGCGG | 20594845 | 2.3346486 | 9.414117 | 35-39 |
| GTTAA | 5390625 | 2.323155 | 26.793333 | 130-134 |
| CGGAT | 10424400 | 2.304104 | 19.539976 | 20-24 |
| CCACG | 17687805 | 2.2925591 | 6.951861 | 85-89 |
| CTCCA | 12562425 | 2.289733 | 18.373863 | 95-99 |
| GGTCG | 13041450 | 2.2894094 | 9.266432 | 9 |
| TGGCC | 14340060 | 2.286013 | 13.082854 | 120-124 |
| GGTCA | 10331935 | 2.2836666 | 12.183819 | 60-64 |
| AACCT | 9033180 | 2.2828393 | 17.761166 | 105-109 |
| GGTGA | 9320860 | 2.268699 | 19.154528 | 115-119 |
| GATTG | 6624250 | 2.2673695 | 18.436945 | 35-39 |
| GGAGT | 9232050 | 2.2470822 | 11.977901 | 115-119 |
| GAACG | 11282450 | 2.2327657 | 15.526344 | 40-44 |
| GCTCC | 15364875 | 2.2242672 | 10.341443 | 15-19 |
| CCGAA | 12351395 | 2.219657 | 11.201308 | 65-69 |
| CACCG | 17062875 | 2.2115605 | 12.409917 | 45-49 |
| ATTCC | 7824310 | 2.2084725 | 19.804804 | 110-114 |
| ATTCG | 7075705 | 2.1993053 | 16.785614 | 90-94 |
| CCAGC | 16962655 | 2.1985707 | 7.563547 | 100-104 |
| CCTAC | 12057920 | 2.1977773 | 15.3923235 | 90-94 |
| ATTCA | 5568380 | 2.179205 | 88.31901 | 6 |
| GTGTG | 7891455 | 2.1453059 | 14.840923 | 135-137 |
| TACAA | 6088690 | 2.1334467 | 26.322826 | 7 |
| GTGAT | 6173660 | 2.11314 | 20.192513 | 120-124 |
| GCCAC | 16216860 | 2.1019063 | 11.304713 | 1 |
| GAAGC | 10601760 | 2.098059 | 16.596817 | 60-64 |
| AACTT | 5357790 | 2.0967898 | 8.123527 | 70-74 |
| CGTTT | 6020610 | 2.0901024 | 18.42792 | 45-49 |
| TAAGA | 5402610 | 2.0846415 | 28.969456 | 130-134 |
| CGAGA | 10506705 | 2.0792477 | 30.710207 | 2 |
| GTTGC | 8403235 | 2.074478 | 12.649424 | 115-119 |
| TTCGT | 5882295 | 2.0420854 | 21.092726 | 125-129 |
| GATAC | 7332295 | 2.0405366 | 15.112247 | 50-54 |
| GCTTC | 9032780 | 2.0249484 | 11.503453 | 100-104 |
| CCTCG | 13979950 | 2.0237808 | 8.728361 | 20-24 |
| CTGAT | 6485235 | 2.0157723 | 23.906057 | 105-109 |
| ATGCA | 7226260 | 2.0110278 | 16.276026 | 85-89 |
| GAAAT | 5206005 | 2.0087798 | 11.022122 | 4 |
| AACCC | 12304865 | 2.0080612 | 9.366651 | 90-94 |
| CGTTA | 6443165 | 2.0026958 | 25.17866 | 125-129 |
| ATGGC | 9044045 | 1.9990044 | 19.107283 | 120-124 |
| GACAT | 7150540 | 1.9899552 | 14.026511 | 105-109 |
| CTACA | 7851395 | 1.9841818 | 35.53881 | 6 |
| TGAAC | 7060965 | 1.965027 | 25.504187 | 7 |
| CACCA | 12020425 | 1.9616426 | 12.901568 | 55-59 |
| TGCAA | 7045880 | 1.9608289 | 18.821747 | 85-89 |
| TGGCG | 11162575 | 1.959575 | 10.463313 | 7 |
| GTAGA | 6384165 | 1.9564935 | 23.401949 | 35-39 |
| GCACC | 14957940 | 1.9387347 | 10.647429 | 45-49 |
| CCTCC | 14741775 | 1.9379287 | 11.808939 | 95-99 |
| AGATC | 6954980 | 1.9355319 | 15.941558 | 4 |
| AGAAC | 7732240 | 1.9266306 | 20.462778 | 40-44 |
| AAGTC | 6902070 | 1.9208075 | 14.141567 | 10-14 |
| ACCTG | 9539065 | 1.9146392 | 14.814491 | 105-109 |
| TAGAG | 6238630 | 1.911893 | 17.858559 | 40-44 |
| TTTTT | 2508575 | 1.8965025 | 7.065478 | 110-114 |
| GGGAG | 10852190 | 1.8783369 | 10.147874 | 115-119 |
| AGGGC | 11859325 | 1.8640015 | 12.779546 | 30-34 |
| AAGGC | 9405930 | 1.8614075 | 10.258534 | 70-74 |
| GCGGA | 11792545 | 1.8535055 | 10.7373495 | 35-39 |
| TGGTG | 6728300 | 1.8291003 | 10.152848 | 130-134 |
| CAGAA | 7300855 | 1.8191433 | 16.884153 | 60-64 |
| TTATT | 2687025 | 1.8188077 | 5.752396 | 3 |
| AGTCG | 8218540 | 1.8165431 | 21.667286 | 7 |
| ACGCG | 12682045 | 1.8101131 | 9.594868 | 55-59 |
| TCCTC | 8886300 | 1.809021 | 6.9381533 | 4 |
| AAGCG | 9125390 | 1.8058895 | 11.722973 | 65-69 |
| TTACC | 6321755 | 1.7843645 | 19.330994 | 75-79 |
| TACCT | 6320880 | 1.7841176 | 15.735209 | 95-99 |
| GCAAC | 9911225 | 1.7811364 | 14.586954 | 85-89 |
| ATATT | 2936945 | 1.7799164 | 10.740532 | 4 |
| AACAG | 7108195 | 1.7711385 | 13.269598 | 9 |
| GGGCC | 15560695 | 1.7639732 | 8.740071 | 30-34 |
| ACCCT | 9671480 | 1.762805 | 10.575388 | 90-94 |
| CAGCC | 13431425 | 1.7408795 | 9.627578 | 9 |
| AGATG | 5679000 | 1.7403884 | 9.675105 | 4 |
| GTCGC | 10861535 | 1.7314857 | 17.045778 | 8 |
| CATAC | 6851125 | 1.7313964 | 13.419988 | 105-109 |
| GCGTT | 7008445 | 1.7301509 | 13.174969 | 45-49 |
| GCCAG | 12111305 | 1.7286512 | 8.568611 | 40-44 |
| CCATT | 6100265 | 1.7218473 | 62.744286 | 4 |
| CGCGC | 16677990 | 1.7168678 | 5.4839854 | 55-59 |
| AGGGT | 7041095 | 1.7138035 | 16.834112 | 115-119 |
| TCGCC | 11767505 | 1.7035007 | 10.671753 | 9 |
| GCGGG | 13613380 | 1.6994126 | 7.809016 | 9 |
| CAAGA | 6769710 | 1.6867987 | 17.506802 | 9 |
| CCGTA | 8388240 | 1.6836507 | 13.428594 | 35-39 |
| GGAGA | 7667435 | 1.6709354 | 11.138087 | 90-94 |
| CGGAG | 10605140 | 1.6668738 | 9.336586 | 85-89 |
| TGAAG | 5422975 | 1.6619269 | 8.403474 | 3 |
| CGGGC | 14641290 | 1.6597488 | 12.286447 | 80-84 |
| CGGGA | 10534285 | 1.6557372 | 10.985804 | 110-114 |
| CCCCG | 17696005 | 1.65424 | 5.0543656 | 30-34 |
| CCCTC | 12578640 | 1.6535667 | 10.580684 | 15-19 |
| GGCCG | 14411940 | 1.6337495 | 7.1447525 | 35-39 |
| AGCCA | 9086265 | 1.6328837 | 12.257985 | 100-104 |
| CGCCT | 11244330 | 1.627764 | 8.467562 | 75-79 |
| TCGAA | 5841565 | 1.6256747 | 5.461238 | 10-14 |
| GTCTA | 5222035 | 1.6231383 | 21.759127 | 4 |
| GAACC | 8996890 | 1.6168222 | 16.454807 | 9 |
| ATACC | 6392440 | 1.6154789 | 12.569033 | 110-114 |
| TGAAT | 3743520 | 1.6133151 | 9.247321 | 8 |
| GAGGG | 9316555 | 1.6125436 | 5.6469803 | 35-39 |
| AATTT | 2654055 | 1.6084728 | 14.825439 | 6 |
| CGTGG | 9133420 | 1.6033598 | 14.379268 | 65-69 |
| CTTGC | 7105685 | 1.5929365 | 12.722577 | 8 |
| GCCGT | 9991770 | 1.5928327 | 13.618543 | 1 |
| TCGTC | 7098200 | 1.5912585 | 21.483145 | 2 |
| TACGC | 7921650 | 1.5899988 | 12.181358 | 55-59 |
| CACGA | 8811015 | 1.583419 | 6.041221 | 60-64 |
| GGCGG | 12632930 | 1.5770191 | 6.508614 | 8 |
| GCCAA | 8758005 | 1.5738924 | 14.781936 | 100-104 |
| AAGGG | 7183595 | 1.565494 | 14.773565 | 30-34 |
| GAGAG | 7160125 | 1.5603793 | 6.148085 | 100-104 |
| CCGGA | 10918185 | 1.5583566 | 9.904503 | 50-54 |
| CCACC | 13220150 | 1.5560126 | 9.628981 | 55-59 |
| CCTGA | 7744460 | 1.5544341 | 17.22548 | 105-109 |
| CTACG | 7740535 | 1.5536462 | 11.965419 | 75-79 |
| AAATA | 3181045 | 1.545436 | 6.4591885 | 105-109 |
| CTATA | 3943750 | 1.5434004 | 7.44819 | 60-64 |
| ACTTC | 5466905 | 1.5430765 | 5.4893928 | 70-74 |
| CGCTC | 10658870 | 1.5430111 | 9.758457 | 15-19 |
| TCACC | 8418405 | 1.534409 | 9.28664 | 65-69 |
| TAACA | 4357715 | 1.5269216 | 16.575274 | 8 |
| TCCAC | 8375185 | 1.5265315 | 7.4263544 | 75-79 |
| TAACT | 3898970 | 1.5258756 | 8.677084 | 70-74 |
| TCTAC | 5384280 | 1.519755 | 19.837914 | 5 |
| GTCGA | 6862695 | 1.5168607 | 5.099436 | 125-129 |
| CCCTA | 8239200 | 1.5017457 | 13.090506 | 90-94 |
| TTGAA | 3481230 | 1.5002781 | 11.223676 | 9 |
| CAGGG | 9533600 | 1.4984534 | 10.521876 | 115-119 |
| CCGTT | 6647950 | 1.4903222 | 19.1815 | 125-129 |
| CCCGC | 15942300 | 1.490302 | 5.2085457 | 30-34 |
| CTACC | 8120940 | 1.4801906 | 13.742921 | 90-94 |
| TACGG | 6681645 | 1.4768434 | 10.86226 | 80-84 |
| AGAGG | 6775025 | 1.4764558 | 6.31097 | 100-104 |
| ACAAG | 5896960 | 1.4693369 | 17.54083 | 8 |
| GCGGT | 8339150 | 1.4639268 | 11.4834585 | 60-64 |
| TGCCT | 6521695 | 1.4620187 | 9.445262 | 120-124 |
| CATTC | 5161100 | 1.4567606 | 63.695347 | 5 |
| AGGCC | 10182760 | 1.4533892 | 7.396424 | 70-74 |
| GGGCA | 9191705 | 1.4447156 | 10.622568 | 80-84 |
| GAATA | 3736205 | 1.4416454 | 11.8053055 | 15-19 |
| TATAC | 3675725 | 1.4385079 | 8.702917 | 60-64 |
| GGCAT | 6490980 | 1.4347007 | 18.008797 | 80-84 |
| CCCTT | 7014565 | 1.4279841 | 8.220291 | 125-129 |
| GTGGT | 5250820 | 1.4274447 | 9.702149 | 130-134 |
| AACGC | 7934995 | 1.4259902 | 10.058837 | 45-49 |
| CATGC | 7061715 | 1.4173964 | 11.450953 | 85-89 |
| ACGTC | 7055460 | 1.4161412 | 6.302373 | 55-59 |
| ACCTC | 7732655 | 1.4094186 | 10.819624 | 95-99 |
| GTCAC | 7009390 | 1.406894 | 10.85425 | 60-64 |
| GGTGT | 5174395 | 1.4066685 | 10.032759 | 130-134 |
| TAGTC | 4523535 | 1.4060272 | 22.549038 | 6 |
| ACGTT | 4521990 | 1.405547 | 10.00117 | 135-137 |
| CCTTC | 6895970 | 1.403841 | 8.082896 | 125-129 |
| GCATG | 6325845 | 1.3982009 | 16.922487 | 80-84 |
| CTCGG | 8764690 | 1.3972185 | 9.686653 | 20-24 |
| ATGCG | 6312980 | 1.3953574 | 6.1175237 | 55-59 |
| TCTGA | 4468590 | 1.3889488 | 6.1556735 | 15-19 |
| TCCGA | 6911370 | 1.38722 | 5.007031 | 45-49 |
| CCAAC | 8497750 | 1.3867686 | 12.625237 | 100-104 |
| CCAGA | 7709315 | 1.3854336 | 10.790014 | 60-64 |
| CTCTC | 6794220 | 1.3831276 | 8.166926 | 1 |
| CTGAA | 4955290 | 1.3790294 | 5.5246205 | 20-24 |
| CTCCT | 6769185 | 1.3780311 | 7.1136928 | 3 |
| GGGTC | 7780875 | 1.3659222 | 5.0956793 | 9 |
| GCCCC | 14540095 | 1.3592224 | 5.0705996 | 30-34 |
| AAAGA | 3921755 | 1.3548678 | 18.228338 | 135-137 |
| GGACG | 8612410 | 1.3536643 | 13.645826 | 50-54 |
| GCGTG | 7611150 | 1.3361273 | 11.760913 | 65-69 |
| AAAAG | 3864040 | 1.3349288 | 27.468842 | 135-137 |
| GACGC | 9329245 | 1.3315667 | 13.2941885 | 50-54 |
| CAAGG | 6685575 | 1.3230568 | 11.572865 | 30-34 |
| ACAGC | 7316565 | 1.3148528 | 14.571822 | 8 |
| CGTAG | 5914895 | 1.3073686 | 17.88003 | 35-39 |
| CACGT | 6498655 | 1.3043815 | 5.5599456 | 135-137 |
| TTAGT | 2706440 | 1.302713 | 35.23067 | 5 |
| TCTCC | 6348620 | 1.2924149 | 7.459728 | 2 |
| CCGGG | 11400560 | 1.2923769 | 6.7118926 | 80-84 |
| TTGGT | 3376105 | 1.290663 | 7.2954698 | 7 |
| CAAGT | 4629055 | 1.2882401 | 62.45517 | 9 |
| CTCCC | 9795845 | 1.2877452 | 8.876791 | 15-19 |
| CGCAC | 9892050 | 1.2821324 | 11.33255 | 45-49 |
| GGGTG | 6612370 | 1.2782757 | 15.930346 | 115-119 |
| GTAGT | 3720040 | 1.2733073 | 6.7328544 | 80-84 |
| GCCTC | 8790665 | 1.2725639 | 5.787897 | 120-124 |
| CGTAA | 4569900 | 1.2717775 | 12.846551 | 6 |
| TTTGG | 3301430 | 1.2621152 | 5.3024344 | 95-99 |
| AATAG | 3244645 | 1.2519729 | 8.432539 | 15-19 |
| GTCGT | 5058245 | 1.2487116 | 18.679956 | 1 |
| AGGGA | 5718060 | 1.2461154 | 6.0718904 | 35-39 |
| ACCAG | 6900490 | 1.2400802 | 10.237834 | 60-64 |
| GGTTC | 5021685 | 1.2396863 | 7.5678077 | 65-69 |
| GCTAT | 3971490 | 1.2344378 | 5.721057 | 60-64 |
| TAAAT | 2262635 | 1.2277416 | 7.4221215 | 105-109 |
| ACCGC | 9451175 | 1.2249897 | 11.090005 | 10-14 |
| TGTGC | 4959345 | 1.2242966 | 13.628743 | 135-137 |
| ACGAA | 4912295 | 1.2239894 | 6.635893 | 125-129 |
| ACGGA | 6151220 | 1.2173095 | 10.06362 | 85-89 |
| CGGCT | 7630000 | 1.2163324 | 5.723278 | 70-74 |
| GGCGC | 10681170 | 1.2108263 | 7.9539323 | 70-74 |
| ACAGT | 4349610 | 1.2104721 | 5.5434012 | 15-19 |
| CACGG | 8403050 | 1.1993705 | 7.1327505 | 85-89 |
| GCCTA | 5960350 | 1.1963353 | 10.021532 | 75-79 |
| AAATT | 2197310 | 1.1922953 | 14.1896305 | 3 |
| CGACA | 6582285 | 1.1828959 | 9.338578 | 105-109 |
| GCGAC | 8241180 | 1.1762669 | 7.2061534 | 105-109 |
| GTAAC | 4199310 | 1.1686444 | 13.085218 | 7 |
| CTGCG | 7330400 | 1.1685718 | 8.07294 | 1 |
| AGGTT | 3399160 | 1.1634754 | 9.495447 | 65-69 |
| TCGGA | 5220050 | 1.1537871 | 14.726491 | 20-24 |
| CGTCT | 5128440 | 1.1496822 | 16.646011 | 3 |
| ACCGA | 6354365 | 1.1419368 | 8.973103 | 65-69 |
| TTATC | 2610465 | 1.1410333 | 6.289568 | 50-54 |
| CAGTC | 5662335 | 1.1365192 | 7.620908 | 5 |
| CGCCA | 8676885 | 1.124632 | 7.7734914 | 55-59 |
| GTCCC | 7706585 | 1.1156292 | 32.9123 | 1 |
| CGCGA | 7796685 | 1.1128238 | 7.5519333 | 100-104 |
| GCCCG | 10794775 | 1.111237 | 9.044632 | 125-129 |
| ATCAT | 2835120 | 1.1095341 | 5.927741 | 50-54 |
| ATAAA | 2278015 | 1.1067201 | 5.6104527 | 105-109 |
| TATTG | 2298135 | 1.1061803 | 8.011645 | 5 |
| AGTCT | 3557175 | 1.1056584 | 6.965476 | 15-19 |
| CAACA | 4875115 | 1.1030831 | 5.0215397 | 10-14 |
| GTGCA | 4980125 | 1.1007565 | 12.438052 | 135-137 |
| GTTCG | 4458040 | 1.1005411 | 5.4145865 | 65-69 |
| AGTAG | 3590855 | 1.1004547 | 5.9225016 | 80-84 |
| ATTTG | 2272850 | 1.0940096 | 12.51469 | 5 |
| CCAGG | 7659660 | 1.0932661 | 8.459868 | 115-119 |
| ACCAC | 6694340 | 1.0924656 | 5.081663 | 30-34 |
| TACGT | 3504840 | 1.0893914 | 5.6301217 | 60-64 |
| TCGTG | 4391190 | 1.0840381 | 9.164389 | 130-134 |
| GTGGC | 6172870 | 1.083639 | 13.387256 | 65-69 |
| CGAGG | 6864835 | 1.0789875 | 5.5841975 | 65-69 |
| CACCT | 5910000 | 1.0772061 | 13.288575 | 3 |
| TACAG | 3839525 | 1.0685184 | 20.327335 | 7 |
| TCGGT | 4305210 | 1.0628124 | 8.136806 | 7 |
| ACGAG | 5348725 | 1.0584979 | 6.1647506 | 30-34 |
| CGGAC | 7404635 | 1.0568664 | 10.355732 | 50-54 |
| GTCAG | 4753925 | 1.0507596 | 7.0549693 | 1 |
| GCAGT | 4736365 | 1.0468782 | 8.472267 | 4 |
| ACGGG | 6585110 | 1.0350214 | 8.06283 | 80-84 |
| TTGGA | 2981750 | 1.020603 | 6.14036 | 95-99 |
| TATCA | 2583565 | 1.0110872 | 6.596527 | 50-54 |
| TGCCC | 6891160 | 0.9975857 | 7.3164396 | 30-34 |
| ACGCC | 7684175 | 0.9959645 | 8.773197 | 55-59 |
| ACGCA | 5525510 | 0.99298406 | 10.370322 | 45-49 |
| AGCTG | 4481555 | 0.9905576 | 6.6984997 | 50-54 |
| ACCAA | 4334080 | 0.98066396 | 5.9546022 | 45-49 |
| TGCGC | 6124120 | 0.97627324 | 7.9804087 | 2 |
| GAGTA | 3182155 | 0.9752044 | 5.63781 | 75-79 |
| CCGCT | 6722705 | 0.97319955 | 10.937453 | 10-14 |
| GCGCG | 8563865 | 0.97080684 | 6.1584096 | 55-59 |
| TTCGA | 3098275 | 0.96302086 | 5.04156 | 40-44 |
| TCGGC | 6029510 | 0.9611911 | 5.2937775 | 70-74 |
| TCCCA | 5224985 | 0.9523496 | 39.583847 | 2 |
| AAGCT | 3420525 | 0.951913 | 6.052777 | 45-49 |
| CCCAT | 5162440 | 0.9409495 | 40.387135 | 3 |
| CAGCG | 6579075 | 0.93903387 | 7.4257784 | 45-49 |
| GCTGG | 5269005 | 0.9249669 | 9.368207 | 5 |
| GAGGT | 3771945 | 0.91809195 | 6.4887924 | 65-69 |
| CCGCG | 8918100 | 0.91804814 | 5.937006 | 30-34 |
| TGCGG | 5181390 | 0.9095861 | 7.5220666 | 50-54 |
| GGCGA | 5780850 | 0.90861106 | 10.561603 | 1 |
| CTAAT | 2316420 | 0.9065391 | 5.040099 | 100-104 |
| TATAT | 1495320 | 0.90622896 | 9.1695385 | 3 |
| GACCA | 5021315 | 0.9023756 | 5.418876 | 45-49 |
| GCCGC | 8624630 | 0.88783777 | 5.5753837 | 70-74 |
| AACCG | 4903255 | 0.8811592 | 16.285172 | 9 |
| CCAAG | 4858050 | 0.8730354 | 5.0467787 | 45-49 |
| TTATA | 1417265 | 0.85892415 | 9.155336 | 2 |
| CAGAT | 3024055 | 0.8415776 | 7.3619137 | 3 |
| GTCCA | 4183650 | 0.8397239 | 6.1287336 | 60-64 |
| GCGAG | 5342005 | 0.83963513 | 23.336731 | 1 |
| TTCGG | 3378060 | 0.83393013 | 7.971764 | 70-74 |
| ATAGC | 2988770 | 0.8317579 | 8.140882 | 20-24 |
| GTCGG | 4727940 | 0.82998353 | 6.346975 | 1 |
| CTGGC | 5198035 | 0.828642 | 8.3089075 | 6 |
| GCGCA | 5799380 | 0.82774776 | 5.8596005 | 2 |
| GTTAG | 2411795 | 0.82551694 | 24.805315 | 4 |
| GATGA | 2625100 | 0.80448914 | 7.912302 | 1 |
| ACTCT | 2849550 | 0.8043077 | 5.885879 | 5 |
| GGCCC | 7670315 | 0.7895986 | 6.2754517 | 125-129 |
| CTTAT | 1783995 | 0.7797837 | 6.8132553 | 1 |
| CCCGT | 5369345 | 0.77728295 | 10.29202 | 125-129 |
| CGAAT | 2712360 | 0.75483453 | 7.6375046 | 15-19 |
| ACCTA | 2909410 | 0.7352577 | 17.793344 | 4 |
| CAGGA | 3607245 | 0.71386385 | 5.2616096 | 90-94 |
| GGGCG | 5717905 | 0.71378887 | 5.6376944 | 2 |
| GAATG | 2309205 | 0.7076798 | 6.4615607 | 9 |
| CGCTG | 4412390 | 0.7033988 | 8.165576 | 4 |
| TCTAA | 1727430 | 0.6760358 | 5.6170697 | 100-104 |
| CAAGC | 3756380 | 0.6750554 | 5.265023 | 45-49 |
| TCAGA | 2408060 | 0.67014956 | 6.9874268 | 2 |
| AGGAC | 3383535 | 0.6695922 | 5.4283843 | 90-94 |
| TAGAT | 1543990 | 0.6654012 | 5.158169 | 135-137 |
| TGACT | 2111745 | 0.65638286 | 5.7010384 | 3 |
| GACGT | 2959795 | 0.6542032 | 6.025868 | 55-59 |
| CTCGT | 2754730 | 0.6175492 | 7.8875437 | 1 |
| TAGCC | 2701010 | 0.54213494 | 5.172876 | 20-24 |
| CGCAG | 3563825 | 0.5086661 | 5.324302 | 3 |
| GCAGA | 2541825 | 0.5030201 | 5.074934 | 1 |
| AGCGC | 3486060 | 0.49756667 | 5.5820136 | 1 |

Produced by FastQC (version 0.10.1)
