# Supplementary material for: Freshwater sponge hosts and their green algae symbionts: a tractable model to understand intracellular symbiosis
Source: PeerJ. 2021 Feb 11;9:e10654. doi: 10.7717/peerj.10654 (PMC7882143; doi:10.7717/peerj.10654)
Supplement: Supplemental Information 27 [file peerj-09-10654-s027.zip › EmApo2_Clean_Data2.fq_fastqc/fastqc_report.html]

EmApo2\_Clean\_Data2.fq.gz FastQC Report


FastQC Report

Tue 10 Sep 2019  
EmApo2\_Clean\_Data2.fq.gz

## Summary

- Basic Statistics
- Per base sequence quality
- Per sequence quality scores
- Per base sequence content
- Per base GC content
- Per sequence GC content
- Per base N content
- Sequence Length Distribution
- Sequence Duplication Levels
- Overrepresented sequences
- Kmer Content

## Basic Statistics

| Measure | Value |
| --- | --- |
| Filename | EmApo2\_Clean\_Data2.fq.gz |
| File type | Conventional base calls |
| Encoding | Sanger / Illumina 1.9 |
| Total Sequences | 33150512 |
| Filtered Sequences | 0 |
| Sequence length | 100-141 |
| %GC | 58 |

## Per base sequence quality

## Per sequence quality scores

## Per base sequence content

## Per base GC content

## Per sequence GC content

## Per base N content

## Sequence Length Distribution

## Sequence Duplication Levels

## Overrepresented sequences

| Sequence | Count | Percentage | Possible Source |
| --- | --- | --- | --- |
| CTCGGAGACGCCGGAGGGGACCCTGGGAAGAGTTCTCTTTTCTTCTTAAC | 802011 | 2.419302000524155 | No Hit |
| GGCAACTCCCGGTATGTCGCGAAGCGCGAATCTCCGTGGCCCGTAGGCGG | 655712 | 1.9779845330895645 | No Hit |
| CGGAGACGCCGGAGGGGACCCTGGGAAGAGTTCTCTTTTCTTCTTAACGG | 574458 | 1.7328782131630427 | No Hit |
| GCAGGTGCACACCACGAAGGGAGGCAACTCCCGGTATGTCGCGAAGCGCG | 504198 | 1.520935785245187 | No Hit |
| GTTTCGACGTGCCGGCACGCCGGCGAGGACTTCGGCCCTCGCAGGCGTAG | 482407 | 1.4552022605261723 | No Hit |
| GTGCACACCACGAAGGGAGGCAACTCCCGGTATGTCGCGAAGCGCGAATC | 448729 | 1.3536110694157606 | No Hit |
| GGTGCACACCACGAAGGGAGGCAACTCCCGGTATGTCGCGAAGCGCGAAT | 360225 | 1.0866348006932744 | No Hit |
| CAGGTTTCGACGTGCCGGCACGCCGGCGAGGACTTCGGCCCTCGCAGGCG | 345271 | 1.0415253918250191 | No Hit |
| CACGAAGGGAGGCAACTCCCGGTATGTCGCGAAGCGCGAATCTCCGTGGC | 287087 | 0.8660107572395865 | No Hit |
| AGCATATGTAGCCAGGCGTCGCCCCGCGTGAGGTTCAGGTTTCGACGTGC | 276679 | 0.8346145604025664 | No Hit |
| GCCAGGCGTCGCCCCGCGTGAGGTTCAGGTTTCGACGTGCCGGCACGCCG | 261004 | 0.7873302228333607 | No Hit |
| GGAGACGCCGGAGGGGACCCTGGGAAGAGTTCTCTTTTCTTCTTAACGGG | 240953 | 0.7268454858253773 | No Hit |
| GGGACGTATAGCCGCGTCGTTCGGAGCGCGCCCGCGACCGAGGAGAGGGT | 223705 | 0.6748161235036129 | No Hit |
| GCCGGAGGGGACCCTGGGAAGAGTTCTCTTTTCTTCTTAACGGGCCATCA | 211738 | 0.6387171335392949 | No Hit |
| CGACGTGCCGGCACGCCGGCGAGGACTTCGGCCCTCGCAGGCGTAGCCGA | 210133 | 0.6338755793575677 | No Hit |
| GGGAAGAGTTCTCTTTTCTTCTTAACGGGCCATCACCCTGGAATCAGGTT | 208351 | 0.6285000967707528 | No Hit |
| GGCGTCGCCCCGCGTGAGGTTCAGGTTTCGACGTGCCGGCACGCCGGCGA | 200262 | 0.6040992670037796 | No Hit |
| GGAAGCTCCCTGTAGCACGGTGCAACTCGCCATCTTGGCGACCGGCACCC | 189470 | 0.5715447170167387 | No Hit |
| GTGCCGGCACGCCGGCGAGGACTTCGGCCCTCGCAGGCGTAGCCGACCGC | 184287 | 0.5559099660361204 | No Hit |
| CACATTTCCCCGCGGGCTGCAGGTGCACACCACGAAGGGAGGCAACTCCC | 176630 | 0.5328122835629205 | No Hit |
| GGAAGAGTTCTCTTTTCTTCTTAACGGGCCATCACCCTGGAATCAGGTTG | 176237 | 0.5316267815109462 | No Hit |
| GGGAAGCATATGTAGCCAGGCGTCGCCCCGCGTGAGGTTCAGGTTTCGAC | 171642 | 0.5177657587912972 | No Hit |
| GTCGGAAGCGAGGGTCGACGAAGCGGGCTGGCGGGGGGGCCCTCTCGGGG | 168484 | 0.5082395107502412 | No Hit |
| GACGTATAGCCGCGTCGTTCGGAGCGCGCCCGCGACCGAGGAGAGGGTCT | 167462 | 0.5051566021061756 | No Hit |
| GGCTGCAGGTGCACACCACGAAGGGAGGCAACTCCCGGTATGTCGCGAAG | 162124 | 0.4890542867030228 | No Hit |
| CTCTTTTCTTCTTAACGGGCCATCACCCTGGAATCAGGTTGGCTGGAGGT | 154068 | 0.46475300290987964 | No Hit |
| GACGTGCCGGCACGCCGGCGAGGACTTCGGCCCTCGCAGGCGTAGCCGAC | 149946 | 0.4523188058151259 | No Hit |
| CGTGCCGGCACGCCGGCGAGGACTTCGGCCCTCGCAGGCGTAGCCGACCG | 148666 | 0.4484576286483901 | No Hit |
| TGCAGGTGCACACCACGAAGGGAGGCAACTCCCGGTATGTCGCGAAGCGC | 130924 | 0.39493809326383855 | No Hit |
| GAGACGCCGGAGGGGACCCTGGGAAGAGTTCTCTTTTCTTCTTAACGGGC | 130866 | 0.3947631336734709 | No Hit |
| GCTGCAGGTGCACACCACGAAGGGAGGCAACTCCCGGTATGTCGCGAAGC | 130161 | 0.3926364696871047 | No Hit |
| GAAGCGGGCTGGCGGGGGGGCCCTCTCGGGGGTCCTGCCGCCGGAGCGTG | 127891 | 0.3857889133054717 | No Hit |
| TCGGAGACGCCGGAGGGGACCCTGGGAAGAGTTCTCTTTTCTTCTTAACG | 125352 | 0.37812990640989197 | No Hit |
| GGACGTATAGCCGCGTCGTTCGGAGCGCGCCCGCGACCGAGGAGAGGGTC | 120489 | 0.3634604497209576 | No Hit |
| GTTCTCTTTTCTTCTTAACGGGCCATCACCCTGGAATCAGGTTGGCTGGA | 119007 | 0.3589899305325963 | No Hit |
| CCGGTATGTCGCGAAGCGCGAATCTCCGTGGCCCGTAGGCGGCCTTCGGT | 118467 | 0.35736099641537966 | No Hit |
| GTCCCGACTTTGCGGAAGGGATGTATTTATTAGATCCAAAGCCAATGCGG | 115306 | 0.3478256987403392 | No Hit |
| GAAGAGTTCTCTTTTCTTCTTAACGGGCCATCACCCTGGAATCAGGTTGG | 114044 | 0.34401881937751067 | No Hit |
| GTTCAGGTTTCGACGTGCCGGCACGCCGGCGAGGACTTCGGCCCTCGCAG | 113354 | 0.34193740356106717 | No Hit |
| TTCGACGTGCCGGCACGCCGGCGAGGACTTCGGCCCTCGCAGGCGTAGCC | 111670 | 0.33685754235108045 | No Hit |
| GGGGAAGCTCCCTGTAGCACGGTGCAACTCGCCATCTTGGCGACCGGCAC | 110491 | 0.33330103619515744 | No Hit |
| CCCCGCGTGAGGTTCAGGTTTCGACGTGCCGGCACGCCGGCGAGGACTTC | 108862 | 0.32838708494155383 | No Hit |
| CCCGCGTGAGGTTCAGGTTTCGACGTGCCGGCACGCCGGCGAGGACTTCG | 101371 | 0.30579014888216505 | No Hit |
| GGCGAAGTTAGGGACGTATAGCCGCGTCGTTCGGAGCGCGCCCGCGACCG | 99589 | 0.30041466629535013 | No Hit |
| GTCGACGAAGCGGGCTGGCGGGGGGGCCCTCTCGGGGGTCCTGCCGCCGG | 98745 | 0.29786870260103376 | No Hit |
| GGCGAATTGTAGCCGAGAGAGGCACCTGCGCTCGGCAGGCGGTCGACCAA | 98445 | 0.29696373920258007 | No Hit |
| GCAACTCCCGGTATGTCGCGAAGCGCGAATCTCCGTGGCCCGTAGGCGGC | 96778 | 0.291935159251839 | No Hit |
| CATTTCCCCGCGGGCTGCAGGTGCACACCACGAAGGGAGGCAACTCCCGG | 95437 | 0.28788997286075096 | No Hit |
| CGCAACGACACATTTCCCCGCGGGCTGCAGGTGCACACCACGAAGGGAGG | 95269 | 0.2873831933576169 | No Hit |
| CCGGAGGGGACCCTGGGAAGAGTTCTCTTTTCTTCTTAACGGGCCATCAC | 94648 | 0.28550991912281776 | No Hit |
| AGGCAACTCCCGGTATGTCGCGAAGCGCGAATCTCCGTGGCCCGTAGGCG | 93049 | 0.28068646420905957 | No Hit |
| AGCGAGGGTCGACGAAGCGGGCTGGCGGGGGGGCCCTCTCGGGGGTCCTG | 88661 | 0.2674498662343435 | No Hit |
| CTGGAATCAGGTTGGCTGGAGGTAGGGTTGCATGCCCGGTAAAGCGCCAC | 86981 | 0.26238207120300283 | No Hit |
| CCCGACTTTGCGGAAGGGATGTATTTATTAGATCCAAAGCCAATGCGGGG | 86548 | 0.261075907364568 | No Hit |
| CGGAGGGGACCCTGGGAAGAGTTCTCTTTTCTTCTTAACGGGCCATCACC | 86420 | 0.26068978964789447 | No Hit |
| CGTATAGCCGCGTCGTTCGGAGCGCGCCCGCGACCGAGGAGAGGGTCTCT | 85485 | 0.25786932038938043 | No Hit |
| AAGCGAGGGTCGACGAAGCGGGCTGGCGGGGGGGCCCTCTCGGGGGTCCT | 82308 | 0.2482857579997558 | No Hit |
| ATTTCCCCGCGGGCTGCAGGTGCACACCACGAAGGGAGGCAACTCCCGGT | 81955 | 0.24722091773424193 | No Hit |
| GACGCCGGAGGGGACCCTGGGAAGAGTTCTCTTTTCTTCTTAACGGGCCA | 77687 | 0.23434630511890733 | No Hit |
| ACGAAGGGAGGCAACTCCCGGTATGTCGCGAAGCGCGAATCTCCGTGGCC | 76885 | 0.23192703630037448 | No Hit |
| GCGAGGGTCGACGAAGCGGGCTGGCGGGGGGGCCCTCTCGGGGGTCCTGC | 76426 | 0.23054244230074034 | No Hit |
| TTTCGACGTGCCGGCACGCCGGCGAGGACTTCGGCCCTCGCAGGCGTAGC | 75931 | 0.22904925269329174 | No Hit |
| CAGGTGCACACCACGAAGGGAGGCAACTCCCGGTATGTCGCGAAGCGCGA | 74192 | 0.22380348152692184 | No Hit |
| CTTTTCTTCTTAACGGGCCATCACCCTGGAATCAGGTTGGCTGGAGGTAG | 73968 | 0.22312777552274307 | No Hit |
| GGCGGTGCTGTTACGGCGACCGGGTGGTGCCCTGACCCGCCTCTCGGGGC | 73155 | 0.22067532471293352 | No Hit |
| AGACGCCGGAGGGGACCCTGGGAAGAGTTCTCTTTTCTTCTTAACGGGCC | 72368 | 0.21830130406432338 | No Hit |
| CGAAGGGAGGCAACTCCCGGTATGTCGCGAAGCGCGAATCTCCGTGGCCC | 71417 | 0.21543257009122513 | No Hit |
| CTGGGAAGAGTTCTCTTTTCTTCTTAACGGGCCATCACCCTGGAATCAGG | 65294 | 0.1969622671287852 | No Hit |
| GGGAGGCAACTCCCGGTATGTCGCGAAGCGCGAATCTCCGTGGCCCGTAG | 65163 | 0.19656709977812709 | No Hit |
| GTCGCGAAGCGCGAATCTCCGTGGCCCGTAGGCGGCCTTCGGTGACCGCG | 64621 | 0.19493213257158742 | No Hit |
| GCCCCGCGTGAGGTTCAGGTTTCGACGTGCCGGCACGCCGGCGAGGACTT | 63194 | 0.19062752333960936 | No Hit |
| AGCGGGCTGGCGGGGGGGCCCTCTCGGGGGTCCTGCCGCCGGAGCGTGGA | 62681 | 0.18908003592825354 | No Hit |
| AAGCGGGCTGGCGGGGGGGCCCTCTCGGGGGTCCTGCCGCCGGAGCGTGG | 62608 | 0.18885982816796315 | No Hit |
| GGCGTGTGCCTGTAACCGTAGTGAATCAACGGGGCTTGATCTGGCGAATA | 62089 | 0.18729424148863824 | No Hit |
| GTCTCTTCGACCCGCCAGCGCAGGCCTTCGTGGCCGGAGCTCCCGCGTTC | 61699 | 0.18611778907064844 | No Hit |
| CTTCGACCCGCCAGCGCAGGCCTTCGTGGCCGGAGCTCCCGCGTTCCGGT | 60727 | 0.18318570765965847 | No Hit |
| GCATATGTAGCCAGGCGTCGCCCCGCGTGAGGTTCAGGTTTCGACGTGCC | 59733 | 0.18018726226611523 | No Hit |
| GGGGACCCTGGGAAGAGTTCTCTTTTCTTCTTAACGGGCCATCACCCTGG | 57710 | 0.1740847924158758 | No Hit |
| CACCACGAAGGGAGGCAACTCCCGGTATGTCGCGAAGCGCGAATCTCCGT | 56753 | 0.1711979591748085 | No Hit |
| AAGCATATGTAGCCAGGCGTCGCCCCGCGTGAGGTTCAGGTTTCGACGTG | 55139 | 0.16632925609112764 | No Hit |
| CCCTGACCCGCCTCTCGGGGCGAAGTTAGGGACGTATAGCCGCGTCGTTC | 54921 | 0.16567164935491796 | No Hit |
| GGAGGGGACCCTGGGAAGAGTTCTCTTTTCTTCTTAACGGGCCATCACCC | 54809 | 0.16533379635282858 | No Hit |
| GCCCTGACCCGCCTCTCGGGGCGAAGTTAGGGACGTATAGCCGCGTCGTT | 53748 | 0.162133242466964 | No Hit |
| GCGTGAGGTTCAGGTTTCGACGTGCCGGCACGCCGGCGAGGACTTCGGCC | 53448 | 0.1612282790685103 | No Hit |
| GAAGCTCCCTGTAGCACGGTGCAACTCGCCATCTTGGCGACCGGCACCCA | 51772 | 0.15617255021581566 | No Hit |
| GCCGCGTCGTTCGGAGCGCGCCCGCGACCGAGGAGAGGGTCTCTTCGACC | 51582 | 0.15559940673012831 | No Hit |
| CCCGGTATGTCGCGAAGCGCGAATCTCCGTGGCCCGTAGGCGGCCTTCGG | 49695 | 0.14990718695385458 | No Hit |
| CCTGGGAAGAGTTCTCTTTTCTTCTTAACGGGCCATCACCCTGGAATCAG | 47938 | 0.14460711798357745 | No Hit |
| GTCGATTCAGACATTTGGCATTTGCGCTTGGCTGAAAAGCCAATGGCGCG | 47600 | 0.1435875258879863 | No Hit |
| CAACTCCCGGTATGTCGCGAAGCGCGAATCTCCGTGGCCCGTAGGCGGCC | 47018 | 0.1418318968949861 | No Hit |
| CTCTTCGACCCGCCAGCGCAGGCCTTCGTGGCCGGAGCTCCCGCGTTCCG | 46872 | 0.1413914813744053 | No Hit |
| GAAGCATATGTAGCCAGGCGTCGCCCCGCGTGAGGTTCAGGTTTCGACGT | 45926 | 0.13853783012461465 | No Hit |
| GCCGAGAGAGGCACCTGCGCTCGGCAGGCGGTCGACCAAAGTTGACCTGG | 45879 | 0.1383960525255236 | No Hit |
| GCAACAAGTCCCGACTTTGCGGAAGGGATGTATTTATTAGATCCAAAGCC | 45508 | 0.13727691445610252 | No Hit |
| GACACATTTCCCCGCGGGCTGCAGGTGCACACCACGAAGGGAGGCAACTC | 44759 | 0.13501752250462978 | No Hit |
| GAGGCAACTCCCGGTATGTCGCGAAGCGCGAATCTCCGTGGCCCGTAGGC | 44712 | 0.13487574490553872 | No Hit |
| CGTCGCCCCGCGTGAGGTTCAGGTTTCGACGTGCCGGCACGCCGGCGAGG | 44527 | 0.13431768414315892 | No Hit |
| GGGACCCTGGGAAGAGTTCTCTTTTCTTCTTAACGGGCCATCACCCTGGA | 43179 | 0.13025138193944033 | No Hit |
| CCTGACCCGCCTCTCGGGGCGAAGTTAGGGACGTATAGCCGCGTCGTTCG | 42980 | 0.12965108955179938 | No Hit |
| GTCGGGCTGCGGTCGGAAGCGAGGGTCGACGAAGCGGGCTGGCGGGGGGG | 42614 | 0.12854703420568586 | No Hit |
| CTCCGGCGCACAGCCGGCGAATTGTAGCCGAGAGAGGCACCTGCGCTCGG | 42097 | 0.12698748061568402 | No Hit |
| CAGGCGTCGCCCCGCGTGAGGTTCAGGTTTCGACGTGCCGGCACGCCGGC | 41750 | 0.12594073961813923 | No Hit |
| TCGACGTGCCGGCACGCCGGCGAGGACTTCGGCCCTCGCAGGCGTAGCCG | 41367 | 0.12478540301278 | No Hit |
| GTGAGGTTCAGGTTTCGACGTGCCGGCACGCCGGCGAGGACTTCGGCCCT | 41245 | 0.1244173845640755 | No Hit |
| CGGAAGCGAGGGTCGACGAAGCGGGCTGGCGGGGGGGCCCTCTCGGGGGT | 40822 | 0.12314138617225581 | No Hit |
| GTCGCCCCGCGTGAGGTTCAGGTTTCGACGTGCCGGCACGCCGGCGAGGA | 40087 | 0.12092422584604424 | No Hit |
| GTGGGTTGCGGGCGGTGCTGTTACGGCGACCGGGTGGTGCCCTGACCCGC | 39806 | 0.12007657679615928 | No Hit |
| CATGCAACAAGTCCCGACTTTGCGGAAGGGATGTATTTATTAGATCCAAA | 39261 | 0.11843255995563506 | No Hit |
| CCGCGTGAGGTTCAGGTTTCGACGTGCCGGCACGCCGGCGAGGACTTCGG | 38894 | 0.11732548806486005 | No Hit |
| GTCTGTCGGGCTGCGGTCGGAAGCGAGGGTCGACGAAGCGGGCTGGCGGG | 38822 | 0.11710829684923117 | No Hit |
| GGACCCTGGGAAGAGTTCTCTTTTCTTCTTAACGGGCCATCACCCTGGAA | 38356 | 0.11570258703696643 | No Hit |
| AGCCAGGCGTCGCCCCGCGTGAGGTTCAGGTTTCGACGTGCCGGCACGCC | 38333 | 0.11563320650975165 | No Hit |
| TGGGAAGAGTTCTCTTTTCTTCTTAACGGGCCATCACCCTGGAATCAGGT | 38328 | 0.1156181237864441 | No Hit |
| GCCGGCACGCCGGCGAGGACTTCGGCCCTCGCAGGCGTAGCCGACCGCCG | 38256 | 0.1154009325708152 | No Hit |
| CGGCGACCGGGTGGTGCCCTGACCCGCCTCTCGGGGCGAAGTTAGGGACG | 38235 | 0.11533758513292344 | No Hit |
| CGCCCCGCGTGAGGTTCAGGTTTCGACGTGCCGGCACGCCGGCGAGGACT | 38149 | 0.11507816229203338 | No Hit |
| GGCGACCGGGTGGTGCCCTGACCCGCCTCTCGGGGCGAAGTTAGGGACGT | 37995 | 0.11461361441416047 | No Hit |
| GGCCAACGTGGGTTGCGGGCGGTGCTGTTACGGCGACCGGGTGGTGCCCT | 37635 | 0.11352765833601605 | No Hit |
| GCACACCACGAAGGGAGGCAACTCCCGGTATGTCGCGAAGCGCGAATCTC | 37584 | 0.11337381455827891 | No Hit |
| GTATAGCCGCGTCGTTCGGAGCGCGCCCGCGACCGAGGAGAGGGTCTCTT | 37383 | 0.11276748908131493 | No Hit |
| CCCCGCGGGCTGCAGGTGCACACCACGAAGGGAGGCAACTCCCGGTATGT | 37364 | 0.1127101747327462 | No Hit |
| TTCCCCGCGGGCTGCAGGTGCACACCACGAAGGGAGGCAACTCCCGGTAT | 37321 | 0.11258046331230118 | No Hit |
| CTTTGCGGAAGGGATGTATTTATTAGATCCAAAGCCAATGCGGGGGGCAA | 36924 | 0.1113828950816808 | No Hit |
| GGGCGGTGCTGTTACGGCGACCGGGTGGTGCCCTGACCCGCCTCTCGGGG | 36828 | 0.11109330679417562 | No Hit |
| GCGAACTCGGAGACGCCGGAGGGGACCCTGGGAAGAGTTCTCTTTTCTTC | 36289 | 0.10946738922162048 | No Hit |
| AAATCTCCGGCGCACAGCCGGCGAATTGTAGCCGAGAGAGGCACCTGCGC | 36108 | 0.10892139463788673 | No Hit |
| GCGTCGCCCCGCGTGAGGTTCAGGTTTCGACGTGCCGGCACGCCGGCGAG | 36010 | 0.10862577326105852 | No Hit |
| GAAGCGAGGGTCGACGAAGCGGGCTGGCGGGGGGGCCCTCTCGGGGGTCC | 35437 | 0.10689729317001197 | No Hit |
| CTCGGGGCGAAGTTAGGGACGTATAGCCGCGTCGTTCGGAGCGCGCCCGC | 34860 | 0.10515674690031937 | No Hit |
| AAGGGAGGCAACTCCCGGTATGTCGCGAAGCGCGAATCTCCGTGGCCCGT | 34761 | 0.10485810897882963 | No Hit |
| CTTCGGCCCTCGCAGGCGTAGCCGACCGCCGCTTCCGCATTTCTCACCGG | 34578 | 0.1043060813057729 | No Hit |
| GGTTTCGACGTGCCGGCACGCCGGCGAGGACTTCGGCCCTCGCAGGCGTA | 34501 | 0.10407380736683644 | No Hit |
| CGGTATGTCGCGAAGCGCGAATCTCCGTGGCCCGTAGGCGGCCTTCGGTG | 33934 | 0.10236342654375898 | No Hit |

## Kmer Content

| Sequence | Count | Obs/Exp Overall | Obs/Exp Max | Max Obs/Exp Position |
| --- | --- | --- | --- | --- |
| TCTCT | 16387920 | 5.0408244 | 17.685978 | 135-137 |
| TTCTC | 14678050 | 4.514879 | 20.32581 | 130-134 |
| TTTCT | 10877380 | 4.4331264 | 21.79402 | 35-39 |
| TTCTT | 10356825 | 4.220972 | 29.860687 | 40-44 |
| ATCTC | 12524780 | 4.026974 | 10.620631 | 120-124 |
| GAATC | 12632630 | 3.855479 | 14.189054 | 60-64 |
| ATCAA | 8147055 | 3.7920973 | 12.772171 | 85-89 |
| CTCTT | 11994595 | 3.6894643 | 10.38448 | 30-34 |
| TCAAA | 7382835 | 3.4363863 | 12.508006 | 85-89 |
| CTTCT | 10867065 | 3.342643 | 15.957159 | 110-114 |
| CATTT | 7737025 | 3.2960274 | 11.936644 | 3 |
| CTTCG | 15408025 | 3.2483401 | 13.747466 | 70-74 |
| AAGAT | 7286940 | 3.0801287 | 11.382953 | 110-114 |
| ATTTC | 7070670 | 3.0121558 | 11.470244 | 4 |
| AATCT | 6748390 | 3.0050242 | 11.503965 | 45-49 |
| TATGT | 7701115 | 2.9793072 | 22.76761 | 5 |
| TTGAA | 7340325 | 2.9683006 | 15.122819 | 135-137 |
| AGGTT | 11149510 | 2.9563384 | 13.30641 | 2 |
| GCGAA | 14067110 | 2.942566 | 9.417096 | 40-44 |
| GCCAA | 12770020 | 2.941495 | 9.94894 | 80-84 |
| TATCA | 6566765 | 2.9241476 | 12.264749 | 90-94 |
| GTTCT | 10446075 | 2.9179358 | 16.438696 | 130-134 |
| CCAAT | 8535810 | 2.868696 | 10.863142 | 100-104 |
| AATCA | 6160290 | 2.867345 | 14.628359 | 60-64 |
| CATCA | 8344055 | 2.8042512 | 11.93548 | 50-54 |
| TCTTC | 9015295 | 2.7730498 | 10.128651 | 35-39 |
| TGGCC | 19177400 | 2.7710288 | 10.503049 | 75-79 |
| CGAAG | 13082170 | 2.7365355 | 14.352364 | 3 |
| AATCC | 8092255 | 2.719627 | 9.545032 | 105-109 |
| CAACT | 8021040 | 2.6956933 | 31.794418 | 3 |
| GGTTG | 15340905 | 2.6671975 | 9.939316 | 85-89 |
| GGCAA | 12741840 | 2.6653452 | 20.334553 | 1 |
| TTCGG | 13811425 | 2.6442215 | 7.051013 | 70-74 |
| AACTC | 7856790 | 2.6404924 | 33.489162 | 4 |
| CTGGA | 13059990 | 2.6135643 | 10.493979 | 60-64 |
| GACCG | 17185010 | 2.595566 | 6.213245 | 55-59 |
| TCACC | 10690565 | 2.5941873 | 10.711748 | 55-59 |
| AAGCG | 12396760 | 2.5931616 | 10.411795 | 100-104 |
| GACTT | 8750055 | 2.5548437 | 6.751606 | 130-134 |
| CCGGT | 17659250 | 2.551664 | 13.178021 | 9 |
| CCATC | 10388315 | 2.520843 | 16.232197 | 50-54 |
| GTAAA | 5932660 | 2.5076857 | 13.843979 | 95-99 |
| TCTTT | 6152200 | 2.5073574 | 12.991358 | 30-34 |
| CTTAA | 5600780 | 2.4939992 | 14.496485 | 40-44 |
| TTTTC | 6083920 | 2.4795299 | 20.08266 | 35-39 |
| CGGTA | 12021195 | 2.4056807 | 10.245839 | 95-99 |
| CGACC | 14452590 | 2.4037158 | 5.456241 | 50-54 |
| GCTGG | 18249780 | 2.3947144 | 8.900884 | 75-79 |
| CGTAT | 8197040 | 2.3933742 | 9.460874 | 5 |
| GGCCA | 15839540 | 2.3923507 | 6.618021 | 50-54 |
| CAAGA | 7469840 | 2.3830156 | 8.89615 | 115-119 |
| CAAAC | 6779090 | 2.3814538 | 11.095868 | 90-94 |
| TAAAG | 5628830 | 2.3792593 | 13.325722 | 95-99 |
| CAGGT | 11873650 | 2.3761542 | 14.342339 | 2 |
| CTTTT | 5812420 | 2.3688784 | 17.70192 | 35-39 |
| TCTTA | 5533505 | 2.3573122 | 22.382689 | 40-44 |
| ACGCT | 10665355 | 2.3502867 | 8.185327 | 105-109 |
| AAAAT | 3624605 | 2.3365686 | 11.825473 | 125-129 |
| TTAAC | 5237965 | 2.3324397 | 13.829031 | 40-44 |
| CCTTC | 10024310 | 2.327149 | 10.780613 | 70-74 |
| AGATC | 7556085 | 2.3061173 | 9.920755 | 115-119 |
| ACCCT | 9484930 | 2.3016262 | 13.172151 | 55-59 |
| GCTTC | 10887505 | 2.2953184 | 9.358488 | 110-114 |
| AAAGC | 7188275 | 2.293191 | 13.932101 | 100-104 |
| CGTGG | 17418175 | 2.285592 | 5.086901 | 90-94 |
| ACACA | 6488170 | 2.2792552 | 6.401508 | 8 |
| TGGAA | 8136300 | 2.255051 | 14.542331 | 60-64 |
| AGCGC | 14895230 | 2.249725 | 8.405282 | 100-104 |
| TGACC | 9990585 | 2.2015898 | 6.556108 | 80-84 |
| CGCGA | 14413710 | 2.1769981 | 8.494586 | 40-44 |
| CGGTG | 16554435 | 2.1722531 | 8.023661 | 125-129 |
| GAAGC | 10317875 | 2.1582992 | 8.368995 | 1 |
| CCGCG | 19668915 | 2.1449819 | 5.4669313 | 85-89 |
| TTCGA | 7322050 | 2.1378944 | 19.517336 | 3 |
| CGAAT | 6994160 | 2.1346176 | 12.711664 | 45-49 |
| CAATC | 6346805 | 2.13302 | 13.36842 | 100-104 |
| GGAAT | 7688385 | 2.130907 | 18.731712 | 60-64 |
| ATGTC | 7296025 | 2.1302958 | 7.717928 | 30-34 |
| TCTCA | 6609980 | 2.1252444 | 8.959578 | 70-74 |
| ATCAC | 6323170 | 2.1250768 | 10.72227 | 50-54 |
| CGCTT | 10057805 | 2.1203997 | 6.6898165 | 105-109 |
| GCAAC | 9186055 | 2.1159506 | 24.602678 | 2 |
| GGAGG | 16976775 | 2.1145947 | 7.7410045 | 8 |
| CACAC | 8332475 | 2.1135168 | 18.123672 | 8 |
| GGTGC | 16081875 | 2.1102448 | 11.443567 | 125-129 |
| GAAGA | 7255845 | 2.102076 | 13.061383 | 25-29 |
| ACTTC | 6520465 | 2.0964637 | 8.753535 | 25-29 |
| TGGAG | 11483705 | 2.0869749 | 9.90926 | 75-79 |
| TGAAA | 4881615 | 2.0634177 | 7.5910025 | 120-124 |
| ACTCC | 8493295 | 2.0609949 | 22.832024 | 5 |
| TGCAT | 7050560 | 2.0586247 | 10.105124 | 85-89 |
| GGGTT | 11825155 | 2.0559428 | 8.253612 | 85-89 |
| ACGAA | 6443510 | 2.0555978 | 16.250393 | 2 |
| GCATT | 7022620 | 2.0504665 | 8.110664 | 65-69 |
| GAGGA | 10766270 | 2.0451796 | 5.6579423 | 25-29 |
| TTGCA | 6970135 | 2.0351422 | 15.61297 | 85-89 |
| CGTAG | 10128485 | 2.0269117 | 9.655397 | 60-64 |
| GTTGC | 10582660 | 2.0260687 | 11.510892 | 85-89 |
| TTGTA | 5197455 | 2.0107238 | 9.177879 | 125-129 |
| CTCTA | 6212095 | 1.9973161 | 17.738178 | 135-137 |
| AGGCG | 14481885 | 1.9863336 | 5.535778 | 45-49 |
| GTATC | 6787065 | 1.9816893 | 11.784176 | 85-89 |
| GCGTA | 9860030 | 1.9731884 | 7.27033 | 80-84 |
| ATCAG | 6448425 | 1.9680597 | 13.155778 | 65-69 |
| CGTTC | 9323025 | 1.9654925 | 10.992984 | 130-134 |
| TGGTG | 11278855 | 1.9609622 | 10.689095 | 115-119 |
| AATAT | 3174645 | 1.9578619 | 6.2776933 | 70-74 |
| CCCTG | 12284040 | 1.9545538 | 5.8254924 | 15-19 |
| TCTAC | 6035000 | 1.9403766 | 27.282534 | 135-137 |
| CTGGC | 13398920 | 1.9360701 | 7.116787 | 95-99 |
| TAGCC | 8662055 | 1.9088264 | 16.36374 | 9 |
| GCCCT | 11987290 | 1.9073372 | 5.741383 | 35-39 |
| CTTGA | 6495610 | 1.8965902 | 12.908397 | 135-137 |
| TCCGC | 11846870 | 1.8849943 | 7.2818522 | 100-104 |
| TCGAC | 8549700 | 1.8840672 | 15.004602 | 4 |
| GTAGG | 10362580 | 1.8832289 | 10.6623125 | 80-84 |
| GGCTG | 14346860 | 1.8825779 | 5.9413 | 70-74 |
| TGAAT | 4644245 | 1.8780527 | 8.318415 | 135-137 |
| ACGTG | 9384405 | 1.8780066 | 13.3534 | 7 |
| GCCAT | 8478170 | 1.8683044 | 11.291797 | 50-54 |
| TTCTG | 6676535 | 1.8649782 | 15.287973 | 110-114 |
| GCCGG | 18773645 | 1.8592452 | 8.72666 | 8 |
| AAACG | 5821420 | 1.8571392 | 12.578803 | 90-94 |
| AGGGA | 9766715 | 1.8553023 | 8.20129 | 6 |
| CAGGC | 12177235 | 1.8392085 | 5.907514 | 3 |
| ATATT | 3104470 | 1.8316535 | 6.106475 | 70-74 |
| GATCT | 6197610 | 1.8095801 | 11.787143 | 115-119 |
| GAAAA | 4086550 | 1.8055581 | 5.725655 | 125-129 |
| GCCTT | 8527290 | 1.7977345 | 7.6795263 | 70-74 |
| TAACG | 5870455 | 1.7916628 | 12.891818 | 45-49 |
| GGCGA | 13035415 | 1.787936 | 5.0975084 | 1 |
| CGGAG | 13031475 | 1.7873955 | 19.888556 | 3 |
| GTGGC | 13618385 | 1.7869884 | 7.612955 | 115-119 |
| CACCA | 7034490 | 1.7842854 | 16.466305 | 6 |
| AAGAG | 6154025 | 1.78287 | 14.639092 | 25-29 |
| ACCGC | 10626430 | 1.7673593 | 5.7655177 | 75-79 |
| TGTTA | 4557265 | 1.7630553 | 9.160426 | 105-109 |
| GTATG | 6628360 | 1.7575371 | 10.652399 | 30-34 |
| GCAGG | 12810985 | 1.7571532 | 9.949539 | 1 |
| CGCCG | 16052410 | 1.750586 | 12.626728 | 9 |
| CAACG | 7531765 | 1.7348953 | 6.66206 | 125-129 |
| TCTCC | 7453785 | 1.7304001 | 7.445812 | 50-54 |
| CTCCC | 9850345 | 1.7258897 | 20.838165 | 6 |
| TGTAG | 6478655 | 1.7178421 | 13.17536 | 7 |
| TCAGG | 8560450 | 1.7131165 | 10.0657215 | 65-69 |
| CGCCA | 10298355 | 1.7127948 | 7.7939134 | 105-109 |
| CCCTC | 9768580 | 1.7115636 | 5.9381094 | 35-39 |
| GGCCC | 15684530 | 1.7104671 | 5.0375338 | 35-39 |
| AACGT | 5593355 | 1.707092 | 6.543066 | 85-89 |
| CCAAC | 6726095 | 1.7060615 | 7.192911 | 85-89 |
| GACGT | 8480280 | 1.697073 | 13.260515 | 6 |
| AGGGT | 9276995 | 1.6859415 | 6.4313703 | 80-84 |
| GGTAA | 6064155 | 1.6807368 | 14.977779 | 95-99 |
| AGGTA | 6052660 | 1.6775508 | 8.786301 | 75-79 |
| GGTAT | 6303225 | 1.6713263 | 8.312482 | 30-34 |
| TCTTG | 5954515 | 1.6632938 | 7.4015174 | 120-124 |
| CTGTT | 5930930 | 1.6567059 | 5.7827334 | 105-109 |
| AAGTT | 4080240 | 1.6499788 | 7.157705 | 5 |
| CACGC | 9877585 | 1.6428134 | 8.96114 | 105-109 |
| AGTTC | 5538280 | 1.6170688 | 9.375265 | 25-29 |
| GACCC | 9703510 | 1.6138617 | 9.207597 | 15-19 |
| AACGC | 6980595 | 1.6079367 | 6.5557814 | 95-99 |
| GAGGT | 8842525 | 1.6069837 | 6.030134 | 75-79 |
| ATCCG | 7265875 | 1.6011552 | 5.841207 | 100-104 |
| AAATC | 3386935 | 1.5764698 | 9.469373 | 125-129 |
| AGAGT | 5634860 | 1.5617536 | 14.689286 | 25-29 |
| GGTGA | 8552935 | 1.5543556 | 6.768481 | 75-79 |
| CATCT | 4820875 | 1.5500104 | 5.408021 | 110-114 |
| CCACG | 9275885 | 1.5427402 | 10.648698 | 8 |
| CCCGC | 12820095 | 1.5395352 | 7.308011 | 9 |
| TCCCG | 9666830 | 1.538121 | 15.166673 | 7 |
| GTAGC | 7645265 | 1.52997 | 9.780163 | 8 |
| CTCAC | 6287790 | 1.5258038 | 5.315246 | 70-74 |
| GGAAG | 8006720 | 1.5209706 | 12.431268 | 2 |
| CTTCC | 6485150 | 1.505531 | 6.5595927 | 60-64 |
| CGCAT | 6733135 | 1.4837571 | 6.106835 | 65-69 |
| CCGCC | 12330580 | 1.4807506 | 5.3432326 | 55-59 |
| CCTGG | 10186175 | 1.4718461 | 6.604318 | 20-24 |
| CCGTA | 6677135 | 1.4714167 | 6.7944217 | 60-64 |
| TTAAT | 2491035 | 1.4697237 | 7.4704237 | 70-74 |
| TAATA | 2378490 | 1.4668584 | 7.6766677 | 70-74 |
| AATGG | 5233285 | 1.4504535 | 6.452252 | 135-137 |
| CCGGA | 9520265 | 1.4379086 | 12.305646 | 9 |
| TACGG | 7145625 | 1.429982 | 12.677894 | 135-137 |
| GGCGT | 10893625 | 1.4294485 | 5.4547825 | 1 |
| GAGGG | 11445765 | 1.4256626 | 7.508554 | 10-14 |
| GTGCC | 9837045 | 1.4213989 | 9.888301 | 9 |
| TTCAG | 4857135 | 1.4181879 | 5.8699636 | 2 |
| CATGC | 6434000 | 1.4178379 | 9.541937 | 90-94 |
| GTTCA | 4855280 | 1.4176462 | 6.7148066 | 1 |
| TGCCC | 8889770 | 1.4144804 | 8.485803 | 90-94 |
| GGAGA | 7431620 | 1.4117236 | 21.87341 | 4 |
| AGGCA | 6735975 | 1.409035 | 5.8503466 | 15-19 |
| GCAAG | 6715450 | 1.4047415 | 6.0899563 | 115-119 |
| TCTGG | 7336850 | 1.4046528 | 7.355253 | 110-114 |
| GCATG | 7018520 | 1.4045457 | 6.5040474 | 85-89 |
| ACGCC | 8419740 | 1.4003487 | 18.847286 | 8 |
| AAGGG | 7333325 | 1.3930513 | 8.394272 | 5 |
| ATGCC | 6305740 | 1.3895736 | 11.003739 | 90-94 |
| CTTGT | 4962355 | 1.3861506 | 9.163827 | 120-124 |
| TTGGC | 7215925 | 1.3815014 | 9.518227 | 70-74 |
| GCGTC | 9536400 | 1.3779572 | 6.8549414 | 120-124 |
| AGGAC | 6550085 | 1.3701506 | 5.9311805 | 25-29 |
| AACGG | 6496960 | 1.3590379 | 10.277059 | 45-49 |
| GACGA | 6492735 | 1.3581539 | 5.1822724 | 130-134 |
| TCGGA | 6782665 | 1.3573464 | 27.548246 | 2 |
| ACCAC | 5335750 | 1.353403 | 16.363672 | 7 |
| GCGTT | 7053860 | 1.3504739 | 7.0692444 | 125-129 |
| GAGTT | 5082425 | 1.347626 | 8.695061 | 25-29 |
| GGCCT | 9317325 | 1.346302 | 5.2881975 | 70-74 |
| CGTCC | 8452590 | 1.3449193 | 8.77749 | 120-124 |
| ACACC | 5259100 | 1.3339609 | 17.93147 | 9 |
| TGGCT | 6953060 | 1.3311756 | 10.426787 | 70-74 |
| TCCGG | 9208695 | 1.3306056 | 7.1465926 | 120-124 |
| GTGAC | 6643425 | 1.3294818 | 8.02778 | 75-79 |
| GAAGG | 6988185 | 1.327488 | 8.43137 | 4 |
| GGTGG | 11092095 | 1.3217659 | 8.412694 | 115-119 |
| CTCCG | 8276990 | 1.316979 | 6.3747396 | 50-54 |
| TAGAC | 4313460 | 1.3164681 | 7.8914466 | 125-129 |
| AGACG | 6287180 | 1.315156 | 23.46216 | 6 |
| ATTTT | 2321075 | 1.310129 | 6.0224524 | 135-137 |
| CGACT | 5926110 | 1.3059158 | 5.7071576 | 130-134 |
| GTGCA | 6523130 | 1.3054085 | 14.193777 | 5 |
| GTCGC | 8994175 | 1.2996087 | 7.816979 | 8 |
| GGTAG | 7127220 | 1.2952553 | 8.20064 | 80-84 |
| GTAGA | 4626925 | 1.2823951 | 5.9488635 | 120-124 |
| GGGAA | 6748630 | 1.2819816 | 12.062224 | 1 |
| TCGCA | 5804255 | 1.2790632 | 5.7517676 | 35-39 |
| TATAG | 3160890 | 1.2782094 | 13.127868 | 7 |
| GCGAC | 8427015 | 1.2727878 | 5.4584804 | 115-119 |
| GCACA | 5518215 | 1.2710866 | 16.47409 | 7 |
| TGGGA | 6940605 | 1.2613411 | 9.832615 | 20-24 |
| TCCGT | 5969580 | 1.2585146 | 5.704532 | 55-59 |
| CTGGT | 6566855 | 1.2572359 | 6.3462133 | 110-114 |
| TTACG | 4260250 | 1.2439091 | 7.1167927 | 110-114 |
| GTTAC | 4252705 | 1.241706 | 6.5972676 | 110-114 |
| TAGGG | 6780625 | 1.2322674 | 9.6553335 | 80-84 |
| GTTTC | 4406710 | 1.2309405 | 19.600481 | 1 |
| GCCGA | 8094130 | 1.2225101 | 5.2918186 | 50-54 |
| ATATG | 3012460 | 1.2181867 | 16.449251 | 4 |
| GACTA | 3972700 | 1.212468 | 8.421933 | 115-119 |
| CCCGG | 11095915 | 1.2100583 | 9.996545 | 8 |
| CACCC | 6595575 | 1.2079399 | 9.120497 | 55-59 |
| ATGTA | 2956950 | 1.1957396 | 16.919024 | 6 |
| TAGGC | 5951135 | 1.1909409 | 5.0710588 | 60-64 |
| ACGGC | 7803450 | 1.1786067 | 7.2450576 | 135-137 |
| GTATA | 2879695 | 1.164499 | 13.022663 | 6 |
| GTTGG | 6637480 | 1.1540043 | 7.519154 | 70-74 |
| TTGCG | 6001025 | 1.1489067 | 5.157404 | 95-99 |
| TGCAC | 5209050 | 1.1478999 | 15.418446 | 6 |
| ACTTG | 3906985 | 1.1407627 | 8.135857 | 130-134 |
| GAAGT | 4080885 | 1.1310551 | 6.140138 | 4 |
| TTTCG | 4032925 | 1.12653 | 18.659054 | 2 |
| GGGAC | 8169375 | 1.1205105 | 6.1574974 | 15-19 |
| TAAAT | 1810235 | 1.1164051 | 5.9311457 | 3 |
| GAGGC | 8135585 | 1.1158758 | 5.907895 | 9 |
| CTACG | 4983845 | 1.0982723 | 11.846805 | 135-137 |
| ACATT | 2454000 | 1.0927539 | 11.430579 | 2 |
| CTCGG | 7555995 | 1.0917996 | 17.226366 | 1 |
| ACGAC | 4667405 | 1.0751078 | 5.1426716 | 130-134 |
| CACGA | 4619300 | 1.0640271 | 14.47541 | 9 |
| GCCAC | 6247005 | 1.0389851 | 7.3811164 | 105-109 |
| GTCCG | 7177540 | 1.037115 | 8.250256 | 120-124 |
| ATCTT | 2431950 | 1.0360279 | 5.41491 | 30-34 |
| AGTTA | 2547935 | 1.030341 | 6.4750915 | 6 |
| CCTGA | 4661140 | 1.027159 | 5.148896 | 130-134 |
| AATTG | 2526275 | 1.0215821 | 7.7862287 | 5 |
| TGCGT | 5284055 | 1.0116416 | 6.976496 | 125-129 |
| TCCCT | 4356035 | 1.0112559 | 6.5827584 | 7 |
| CACAT | 2986440 | 1.003676 | 9.082949 | 1 |
| GGTTT | 3909980 | 0.99184084 | 12.277922 | 3 |
| AGCTC | 4479490 | 0.98712933 | 6.4232593 | 4 |
| GAGCG | 7190275 | 0.98621726 | 5.131023 | 135-137 |
| ATAGC | 3214755 | 0.98114324 | 10.516676 | 8 |
| AGGTG | 5373755 | 0.9765917 | 12.837435 | 3 |
| CGTGC | 6591265 | 0.95240146 | 9.754747 | 8 |
| AAGCT | 3090760 | 0.9433 | 8.769034 | 3 |
| GTGCG | 7135025 | 0.9362496 | 8.427676 | 125-129 |
| ACGTA | 3030460 | 0.92489654 | 9.69062 | 4 |
| GGACC | 6056125 | 0.9146966 | 7.5844827 | 15-19 |
| CATAT | 2052140 | 0.91380763 | 18.170637 | 3 |
| TCGCC | 5615295 | 0.89346796 | 5.999309 | 9 |
| AAGCA | 2778645 | 0.88643855 | 7.9868345 | 4 |
| TTAAA | 1436725 | 0.88605475 | 5.209757 | 2 |
| GGGAG | 7096670 | 0.8839477 | 5.139261 | 7 |
| GCACG | 5785340 | 0.87379825 | 5.3384705 | 15-19 |
| GCCAG | 5707580 | 0.86205363 | 6.230832 | 1 |
| ACTTT | 2019240 | 0.8602105 | 7.0518394 | 7 |
| CGACG | 5692330 | 0.85975033 | 10.201757 | 5 |
| TCGCG | 5936480 | 0.8577886 | 5.652288 | 35-39 |
| GAGAC | 4096610 | 0.85693127 | 23.652283 | 5 |
| TACTC | 2607030 | 0.83821374 | 5.3852983 | 120-124 |
| CTGCA | 3769755 | 0.83072764 | 5.1790786 | 3 |
| TTTGC | 2859985 | 0.7988888 | 5.1322255 | 9 |
| ATTGT | 2040710 | 0.7894833 | 6.3462424 | 6 |
| CGTCG | 5430620 | 0.7846946 | 5.5377917 | 7 |
| TTCCC | 3318045 | 0.77028596 | 6.3258014 | 6 |
| GAATT | 1841980 | 0.744865 | 7.0219393 | 4 |
| ACGGG | 5415430 | 0.7427798 | 7.1854653 | 45-49 |
| TTTCC | 2376120 | 0.7308801 | 8.292255 | 5 |
| AGCAT | 2273270 | 0.693802 | 12.41797 | 1 |
| CTGGG | 5169985 | 0.67839926 | 6.5287023 | 20-24 |
| GTCGA | 3292370 | 0.65886885 | 5.4514704 | 1 |
| CCTGT | 3123805 | 0.6585647 | 8.478523 | 9 |
| CCAGG | 4306195 | 0.6503932 | 5.7090244 | 2 |
| GTAGT | 2380295 | 0.6311451 | 5.0044208 | 125-129 |
| GACGC | 3744685 | 0.56558454 | 17.034342 | 7 |
| CCCCG | 4243790 | 0.5096268 | 6.382312 | 8 |
| GCATA | 1633420 | 0.49851978 | 12.226558 | 2 |
| CGGAA | 2372505 | 0.49628192 | 5.2296987 | 3 |
| AGCGA | 2360405 | 0.49375084 | 5.067836 | 7 |

Produced by FastQC (version 0.10.1)
