# Supplementary material for: Freshwater sponge hosts and their green algae symbionts: a tractable model to understand intracellular symbiosis
Source: PeerJ. 2021 Feb 11;9:e10654. doi: 10.7717/peerj.10654 (PMC7882143; doi:10.7717/peerj.10654)
Supplement: Supplemental Information 28 [file peerj-09-10654-s028.zip › EmApo3_Clean_Data1.fq_fastqc/fastqc_report.html]

EmApo3\_Clean\_Data1.fq.gz FastQC Report


FastQC Report

Tue 10 Sep 2019  
EmApo3\_Clean\_Data1.fq.gz

## Summary

- Basic Statistics
- Per base sequence quality
- Per sequence quality scores
- Per base sequence content
- Per base GC content
- Per sequence GC content
- Per base N content
- Sequence Length Distribution
- Sequence Duplication Levels
- Overrepresented sequences
- Kmer Content

## Basic Statistics

| Measure | Value |
| --- | --- |
| Filename | EmApo3\_Clean\_Data1.fq.gz |
| File type | Conventional base calls |
| Encoding | Sanger / Illumina 1.9 |
| Total Sequences | 32861912 |
| Filtered Sequences | 0 |
| Sequence length | 100-141 |
| %GC | 57 |

## Per base sequence quality

## Per sequence quality scores

## Per base sequence content

## Per base GC content

## Per sequence GC content

## Per base N content

## Sequence Length Distribution

## Sequence Duplication Levels

## Overrepresented sequences

| Sequence | Count | Percentage | Possible Source |
| --- | --- | --- | --- |
| GTCCCATTCAAGTCGTCTACAAGAGATCTTGCCCCGCGGATTGGCCAGCG | 1557127 | 4.738394406265831 | No Hit |
| GCGAGAAAATGAACCGCTCCCTCGGATTTTCAAGGGCCGTAGAGAACGCA | 986767 | 3.002768067786196 | No Hit |
| GAGAAAATGAACCGCTCCCTCGGATTTTCAAGGGCCGTAGAGAACGCACC | 560658 | 1.706102797670446 | No Hit |
| GCCACCTACAGCCAACAGTCTGAAGCGCAGTCGCGAACCCCGCGCACGGC | 552770 | 1.6820993252005545 | No Hit |
| GTCGTCTACAAGAGATCTTGCCCCGCGGATTGGCCAGCGTTTGATACGCG | 495156 | 1.506777816214711 | No Hit |
| GGCGAGAAAATGAACCGCTCCCTCGGATTTTCAAGGGCCGTAGAGAACGC | 488222 | 1.4856774006332922 | No Hit |
| AGAAAATGAACCGCTCCCTCGGATTTTCAAGGGCCGTAGAGAACGCACCG | 475017 | 1.4454941027168475 | No Hit |
| CGGGCGAGAAAATGAACCGCTCCCTCGGATTTTCAAGGGCCGTAGAGAAC | 428397 | 1.3036277377895724 | No Hit |
| AAGAGATCTTGCCCCGCGGATTGGCCAGCGTTTGATACGCGCGGTCACCG | 391119 | 1.1901894205060253 | No Hit |
| GCCGTTAGTCGCCTGCCGAATAGCCGCCGACCACGAGGGACGGCGACCAA | 363398 | 1.1058334037289126 | No Hit |
| CTGCGCTGGCGGGTCGAAGAGACCCTCTCCTCGGTCGCGGGCGCGCTCCG | 344903 | 1.0495524423533238 | No Hit |
| ATTCAAGTCGTCTACAAGAGATCTTGCCCCGCGGATTGGCCAGCGTTTGA | 309683 | 0.9423766943323322 | No Hit |
| AGCGCAGTCGCGAACCCCGCGCACGGCGGAGGGATGCGCCGGCCTCGCAC | 279456 | 0.8503948279089787 | No Hit |
| GGCCGTTAGTCGCCTGCCGAATAGCCGCCGACCACGAGGGACGGCGACCA | 262139 | 0.7976985636137057 | No Hit |
| CTCTCCTCGGTCGCGGGCGCGCTCCGAACGACGCGGCTATACGTCCCTAA | 238374 | 0.7253807995103876 | No Hit |
| GTCAGATGAAGCCACCTACAGCCAACAGTCTGAAGCGCAGTCGCGAACCC | 220885 | 0.6721611329249497 | No Hit |
| GATGAAGCCACCTACAGCCAACAGTCTGAAGCGCAGTCGCGAACCCCGCG | 196719 | 0.5986231111567702 | No Hit |
| GGGCGAGAAAATGAACCGCTCCCTCGGATTTTCAAGGGCCGTAGAGAACG | 193375 | 0.5884471968642603 | No Hit |
| GTCTACAAGAGATCTTGCCCCGCGGATTGGCCAGCGTTTGATACGCGCGG | 193059 | 0.5874855973079107 | No Hit |
| GCAGAAATTTGAATGCACCATCGCCGGCACGAGGCCATGCGATTCGAGCA | 189508 | 0.5766797744452605 | No Hit |
| GTCGCCGTAACAGCACCGCCCGCAACCCACGTTGGCCAGCCCCGGTGAGA | 188632 | 0.574014074409304 | No Hit |
| GCGCTGGCGGGTCGAAGAGACCCTCTCCTCGGTCGCGGGCGCGCTCCGAA | 168595 | 0.5130407506416547 | No Hit |
| CTCCTCGGTCGCGGGCGCGCTCCGAACGACGCGGCTATACGTCCCTAACT | 168456 | 0.5126177685583236 | No Hit |
| CTCGTCCCATTCAAGTCGTCTACAAGAGATCTTGCCCCGCGGATTGGCCA | 165250 | 0.5028617933125742 | No Hit |
| GAAAATGAACCGCTCCCTCGGATTTTCAAGGGCCGTAGAGAACGCACCGG | 156705 | 0.47685904581571514 | No Hit |
| AGAAATTTGAATGCACCATCGCCGGCACGAGGCCATGCGATTCGAGCAGT | 139157 | 0.42345984007260445 | No Hit |
| GTCGGCCGTTAGTCGCCTGCCGAATAGCCGCCGACCACGAGGGACGGCGA | 138836 | 0.42248302533340115 | No Hit |
| AGAGATCTTGCCCCGCGGATTGGCCAGCGTTTGATACGCGCGGTCACCGA | 131594 | 0.400445354488199 | No Hit |
| GAGATCTTGCCCCGCGGATTGGCCAGCGTTTGATACGCGCGGTCACCGAA | 123973 | 0.3772543727826914 | No Hit |
| CAAGAGATCTTGCCCCGCGGATTGGCCAGCGTTTGATACGCGCGGTCACC | 120526 | 0.36676502572339675 | No Hit |
| CGAGAAAATGAACCGCTCCCTCGGATTTTCAAGGGCCGTAGAGAACGCAC | 118127 | 0.3594647809902236 | No Hit |
| GGCAGAAATTTGAATGCACCATCGCCGGCACGAGGCCATGCGATTCGAGC | 116403 | 0.3542185859422909 | No Hit |
| CCTGACTCTCCAAAGACACCTAATATCTAGGCAGGCGGTCGGCCGCGTAC | 110076 | 0.33496529355930355 | No Hit |
| GCCCGCAACCCACGTTGGCCAGCCCCGGTGAGAAATGCGGAAGCGGCGGT | 107258 | 0.3263900165029959 | No Hit |
| CCGCCCGCAACCCACGTTGGCCAGCCCCGGTGAGAAATGCGGAAGCGGCG | 98754 | 0.30051203350553674 | No Hit |
| CATTCAAGTCGTCTACAAGAGATCTTGCCCCGCGGATTGGCCAGCGTTTG | 96335 | 0.29315092804094905 | No Hit |
| CCCGCAACCCACGTTGGCCAGCCCCGGTGAGAAATGCGGAAGCGGCGGTC | 88591 | 0.26958565283724206 | No Hit |
| GCCTGCGCTGGCGGGTCGAAGAGACCCTCTCCTCGGTCGCGGGCGCGCTC | 88085 | 0.2680458763324544 | No Hit |
| CAAGTCGTCTACAAGAGATCTTGCCCCGCGGATTGGCCAGCGTTTGATAC | 86817 | 0.26418730596077306 | No Hit |
| CCTGCGCTGGCGGGTCGAAGAGACCCTCTCCTCGGTCGCGGGCGCGCTCC | 85117 | 0.2590141437905378 | No Hit |
| GCGGGAGCTCCGGCCACGAAGGCCTGCGCTGGCGGGTCGAAGAGACCCTC | 85023 | 0.2587280983528895 | No Hit |
| CCCATTCAAGTCGTCTACAAGAGATCTTGCCCCGCGGATTGGCCAGCGTT | 84549 | 0.25728569901836507 | No Hit |
| CTTATATTGGTCGGGCTAGGAGCTGAGTCTACTCACAGGCACTATCCCAT | 82103 | 0.2498424315663678 | No Hit |
| AGATGAAGCCACCTACAGCCAACAGTCTGAAGCGCAGTCGCGAACCCCGC | 80019 | 0.2435007433529735 | No Hit |
| CCGGGCGAGAAAATGAACCGCTCCCTCGGATTTTCAAGGGCCGTAGAGAA | 79557 | 0.2420948604572978 | No Hit |
| CGCGGATTGGCCAGCGTTTGATACGCGCGGTCACCGAAGGCCGCCTACGG | 79366 | 0.24151364047228902 | No Hit |
| TTCAAGTCGTCTACAAGAGATCTTGCCCCGCGGATTGGCCAGCGTTTGAT | 74950 | 0.22807559097596025 | No Hit |
| CTACAAGAGATCTTGCCCCGCGGATTGGCCAGCGTTTGATACGCGCGGTC | 72629 | 0.22101270309530374 | No Hit |
| GCCAACAGTCTGAAGCGCAGTCGCGAACCCCGCGCACGGCGGAGGGATGC | 72429 | 0.22040409578115844 | No Hit |
| GCGCAGTCGCGAACCCCGCGCACGGCGGAGGGATGCGCCGGCCTCGCACT | 72406 | 0.22033410594003172 | No Hit |
| CCGCAACCCACGTTGGCCAGCCCCGGTGAGAAATGCGGAAGCGGCGGTCG | 72168 | 0.2196098632361988 | No Hit |
| GAAATTTGAATGCACCATCGCCGGCACGAGGCCATGCGATTCGAGCAGTT | 71724 | 0.21825875499879616 | No Hit |
| GCCGTAACAGCACCGCCCGCAACCCACGTTGGCCAGCCCCGGTGAGAAAT | 70089 | 0.21328339020565815 | No Hit |
| GAAGCCACCTACAGCCAACAGTCTGAAGCGCAGTCGCGAACCCCGCGCAC | 69705 | 0.2121148641624991 | No Hit |
| GCCCACTGGTGTTAGTTTTAGTACAGCCGAGCCCAATTTATTGGGCTGAA | 69374 | 0.21110761905758862 | No Hit |
| CTCCACCGGAACGCGGGAGCTCCGGCCACGAAGGCCTGCGCTGGCGGGTC | 68277 | 0.2077694079395015 | No Hit |
| CTGCTTACAACACCTCGTCCCATTCAAGTCGTCTACAAGAGATCTTGCCC | 66389 | 0.20202415489396966 | No Hit |
| CACCTACAGCCAACAGTCTGAAGCGCAGTCGCGAACCCCGCGCACGGCGG | 66340 | 0.20187504610200405 | No Hit |
| AAGCGCAGTCGCGAACCCCGCGCACGGCGGAGGGATGCGCCGGCCTCGCA | 65884 | 0.20048742142575268 | No Hit |
| GGCGGGTCGAAGAGACCCTCTCCTCGGTCGCGGGCGCGCTCCGAACGACG | 65598 | 0.19961711296652487 | No Hit |
| GCCACGAAGGCCTGCGCTGGCGGGTCGAAGAGACCCTCTCCTCGGTCGCG | 64613 | 0.19661972194435917 | No Hit |
| GGCCTGCGCTGGCGGGTCGAAGAGACCCTCTCCTCGGTCGCGGGCGCGCT | 64157 | 0.19523209726810783 | No Hit |
| CAGATGAAGCCACCTACAGCCAACAGTCTGAAGCGCAGTCGCGAACCCCG | 60421 | 0.18386331263987318 | No Hit |
| GCTCCCTCGGATTTTCAAGGGCCGTAGAGAACGCACCGGACGCCACCAGA | 59988 | 0.18254567780474854 | No Hit |
| CGAGATGGCGCCCTCCACCGGAACGCGGGAGCTCCGGCCACGAAGGCCTG | 59801 | 0.18197662996602268 | No Hit |
| GTCTACTTATATTGGTCGGGCTAGGAGCTGAGTCTACTCACAGGCACTAT | 57437 | 0.17478289151282494 | No Hit |
| CCGAGATGGCGCCCTCCACCGGAACGCGGGAGCTCCGGCCACGAAGGCCT | 54169 | 0.16483824799969035 | No Hit |
| TGACTCTCCAAAGACACCTAATATCTAGGCAGGCGGTCGGCCGCGTACGG | 53539 | 0.16292113496013258 | No Hit |
| CGTTAGTCGCCTGCCGAATAGCCGCCGACCACGAGGGACGGCGACCAAGC | 53491 | 0.1627750692047377 | No Hit |
| CTCGGATTTTCAAGGGCCGTAGAGAACGCACCGGACGCCACCAGAAGCGT | 53488 | 0.1627659400950255 | No Hit |
| GTTAGTCGCCTGCCGAATAGCCGCCGACCACGAGGGACGGCGACCAAGCT | 52880 | 0.16091577386002373 | No Hit |
| CTACTGCTTACAACACCTCGTCCCATTCAAGTCGTCTACAAGAGATCTTG | 51975 | 0.15816182576351615 | No Hit |
| GTCCGAGATGGCGCCCTCCACCGGAACGCGGGAGCTCCGGCCACGAAGGC | 51966 | 0.15813443843437958 | No Hit |
| GACCGGGCGAGAAAATGAACCGCTCCCTCGGATTTTCAAGGGCCGTAGAG | 50392 | 0.1533446988720559 | No Hit |
| AAAATGAACCGCTCCCTCGGATTTTCAAGGGCCGTAGAGAACGCACCGGA | 50240 | 0.15288215731330546 | No Hit |
| CACCGGAACGCGGGAGCTCCGGCCACGAAGGCCTGCGCTGGCGGGTCGAA | 50002 | 0.15215791460947253 | No Hit |
| AGATCTTGCCCCGCGGATTGGCCAGCGTTTGATACGCGCGGTCACCGAAG | 49935 | 0.15195403115923384 | No Hit |
| GCTTACAACACCTCGTCCCATTCAAGTCGTCTACAAGAGATCTTGCCCCG | 49499 | 0.150627267214397 | No Hit |
| ACAAGAGATCTTGCCCCGCGGATTGGCCAGCGTTTGATACGCGCGGTCAC | 46417 | 0.14124862850341757 | No Hit |
| CAGAAATTTGAATGCACCATCGCCGGCACGAGGCCATGCGATTCGAGCAG | 45243 | 0.13767610356938453 | No Hit |
| ACCGCCCGCAACCCACGTTGGCCAGCCCCGGTGAGAAATGCGGAAGCGGC | 44859 | 0.1365075775262255 | No Hit |
| CACCCGGTCGCCGTAACAGCACCGCCCGCAACCCACGTTGGCCAGCCCCG | 44571 | 0.13563118299385624 | No Hit |
| CTCTACTGCTTACAACACCTCGTCCCATTCAAGTCGTCTACAAGAGATCT | 43951 | 0.13374450032000573 | No Hit |
| TACAAGAGATCTTGCCCCGCGGATTGGCCAGCGTTTGATACGCGCGGTCA | 42952 | 0.13070450678584983 | No Hit |
| CTCCAAAGACACCTAATATCTAGGCAGGCGGTCGGCCGCGTACGGGGTTC | 42888 | 0.13050975244532334 | No Hit |
| CTCGTCCCGGTTCGGGAATATTAACCCGATTCCCTTTCGATGGTGGGTGC | 42873 | 0.1304641068967624 | No Hit |
| CTCCAGCCAACCTGATTCCAGGGTGATGGCCCGTTAAGAAGAAAAGAGAA | 42507 | 0.12935035551187649 | No Hit |
| CCATTCAAGTCGTCTACAAGAGATCTTGCCCCGCGGATTGGCCAGCGTTT | 40756 | 0.12402199847653417 | No Hit |
| CTCGGTCGCGGGCGCGCTCCGAACGACGCGGCTATACGTCCCTAACTTCG | 40133 | 0.12212618669297148 | No Hit |
| CCGCGGATTGGCCAGCGTTTGATACGCGCGGTCACCGAAGGCCGCCTACG | 39805 | 0.12112807069777315 | No Hit |
| CTACAGCCAACAGTCTGAAGCGCAGTCGCGAACCCCGCGCACGGCGGAGG | 38911 | 0.11840759600354356 | No Hit |
| GGCATTCGTTAAGCCATTCATACTTGCCAACAATTAATTGGCTATTGATT | 38282 | 0.1164935260005565 | No Hit |
| CAACAGTCTGAAGCGCAGTCGCGAACCCCGCGCACGGCGGAGGGATGCGC | 37920 | 0.11539194676195347 | No Hit |
| CCTCGGTCGCGGGCGCGCTCCGAACGACGCGGCTATACGTCCCTAACTTC | 37663 | 0.11460988636327674 | No Hit |
| GCGCATATGTAGCCCAAAACATTAGGATCATAAGGACCTGACGTCATCCT | 37608 | 0.11444251935188678 | No Hit |
| CCCGGTCGCCGTAACAGCACCGCCCGCAACCCACGTTGGCCAGCCCCGGT | 37277 | 0.11343527424697625 | No Hit |
| CGCCGTAACAGCACCGCCCGCAACCCACGTTGGCCAGCCCCGGTGAGAAA | 36927 | 0.11237021144722194 | No Hit |
| GGCGATTTGTACGAACACTAGAGCCGTATTCACCAGAACGCTCTACTTTC | 36590 | 0.11134470812288706 | No Hit |
| CATTATTCAACCTGGATACAGCCGGACTCCTCCGGCGAACCCATATTGAT | 35434 | 0.10782695784712709 | No Hit |
| GTAAATTAGACCATTAGTAATCTTTAGCTTAGAATAGAGGCGTGGCCTGC | 34872 | 0.10611677129437874 | No Hit |
| GAACCGCTCCCTCGGATTTTCAAGGGCCGTAGAGAACGCACCGGACGCCA | 34762 | 0.1057820372715988 | No Hit |
| GGGAGCTCCGGCCACGAAGGCCTGCGCTGGCGGGTCGAAGAGACCCTCTC | 33527 | 0.10202388710675142 | No Hit |
| GCCAGCGTTTGATACGCGCGGTCACCGAAGGCCGCCTACGGGCCACGGAG | 33521 | 0.10200562888732706 | No Hit |
| GTCCCGGTTCGGGAATATTAACCCGATTCCCTTTCGATGGTGGGTGCCGG | 33137 | 0.10083710284416805 | No Hit |
| CGCCCGCAACCCACGTTGGCCAGCCCCGGTGAGAAATGCGGAAGCGGCGG | 32989 | 0.10038673343170051 | No Hit |

## Kmer Content

| Sequence | Count | Obs/Exp Overall | Obs/Exp Max | Max Obs/Exp Position |
| --- | --- | --- | --- | --- |
| GAGAT | 14388005 | 4.3107038 | 19.608423 | 90-94 |
| AGAGA | 15201745 | 4.076017 | 19.603012 | 40-44 |
| TTCAA | 10815180 | 4.0648584 | 89.96221 | 7 |
| ATTTT | 6174820 | 3.9051807 | 39.883575 | 25-29 |
| GATTC | 12362920 | 3.7775114 | 20.684713 | 110-114 |
| TTGAT | 8084410 | 3.7199156 | 24.52313 | 50-54 |
| AGAAA | 11217455 | 3.699677 | 50.99545 | 4 |
| GGATT | 10959800 | 3.6690648 | 25.09983 | 20-24 |
| TTTGA | 7905070 | 3.6373951 | 25.268322 | 50-54 |
| TTCGC | 14925280 | 3.383859 | 16.333607 | 95-99 |
| ATCTT | 7915085 | 3.3240871 | 25.90712 | 6 |
| GAGAA | 12146885 | 3.2569225 | 37.334152 | 3 |
| AAGAA | 9841195 | 3.2457666 | 40.5296 | 130-134 |
| CTTCG | 13773345 | 3.1226923 | 12.856502 | 100-104 |
| AAATG | 8430060 | 3.1067424 | 52.11836 | 7 |
| TTTCA | 7394295 | 3.1053712 | 31.627323 | 25-29 |
| TTTTC | 6478640 | 3.0402253 | 31.363861 | 25-29 |
| ATTGG | 8939090 | 2.9925818 | 18.909231 | 40-44 |
| TTGCC | 12927935 | 2.931021 | 13.510564 | 25-29 |
| TGATA | 7095010 | 2.9216802 | 21.986588 | 50-54 |
| ACCGG | 19733910 | 2.9131696 | 13.364989 | 75-79 |
| AGAAG | 10692090 | 2.8668509 | 20.34696 | 60-64 |
| CGAAG | 14017930 | 2.7888904 | 12.134259 | 65-69 |
| AGATT | 6751840 | 2.780365 | 21.75415 | 90-94 |
| TGATT | 6040980 | 2.779663 | 28.75807 | 110-114 |
| GGCCA | 18760005 | 2.7693994 | 8.7956085 | 80-84 |
| TCCCT | 13344405 | 2.761347 | 15.659631 | 15-19 |
| TCGCG | 16683220 | 2.7519317 | 11.660052 | 95-99 |
| GATTT | 5972615 | 2.748206 | 28.399525 | 25-29 |
| GCGCT | 16548120 | 2.7296464 | 11.5282955 | 70-74 |
| TTGGC | 10930755 | 2.7152383 | 14.555746 | 40-44 |
| ATGAA | 7363425 | 2.7136538 | 51.14482 | 9 |
| GATGG | 11111660 | 2.7064478 | 17.766684 | 120-124 |
| AGTTG | 8082590 | 2.7058473 | 17.07892 | 115-119 |
| AAGAG | 10088670 | 2.7050571 | 15.623571 | 20-24 |
| AAAAT | 5952165 | 2.6982145 | 62.245735 | 6 |
| CGCTT | 11782940 | 2.6714277 | 16.852581 | 70-74 |
| TGATG | 7966385 | 2.6669447 | 22.448298 | 120-124 |
| TACCG | 13062160 | 2.6503284 | 17.365122 | 75-79 |
| TCTTG | 7729090 | 2.6388717 | 20.374256 | 7 |
| GAGTT | 7845425 | 2.6264505 | 16.658495 | 115-119 |
| TCCAG | 12942325 | 2.6260138 | 14.216306 | 95-99 |
| GTTTG | 6969495 | 2.6071088 | 19.83294 | 50-54 |
| TTTAC | 6155935 | 2.585299 | 26.853973 | 75-79 |
| ATACG | 9413405 | 2.5741055 | 15.220686 | 55-59 |
| TCAAG | 9295210 | 2.5417852 | 64.68213 | 8 |
| ACATA | 7418050 | 2.4951482 | 18.341728 | 105-109 |
| CTTTA | 5895490 | 2.4759207 | 25.70159 | 75-79 |
| CGCGG | 20595585 | 2.471723 | 9.853983 | 35-39 |
| GAAAA | 7473730 | 2.464943 | 45.397457 | 5 |
| GCTTT | 7149485 | 2.4409819 | 22.39694 | 70-74 |
| GAAGG | 11134085 | 2.4270017 | 11.998749 | 70-74 |
| AGCGT | 10805910 | 2.402227 | 14.578382 | 65-69 |
| GAAGA | 8945870 | 2.3986402 | 25.33876 | 135-137 |
| GATCT | 7792665 | 2.381062 | 18.581873 | 5 |
| CAACC | 14300460 | 2.3700721 | 12.951682 | 105-109 |
| CGGTC | 14353315 | 2.3676085 | 10.890983 | 60-64 |
| GGAGT | 9573730 | 2.3318567 | 12.543617 | 115-119 |
| TTAAG | 5631035 | 2.3188248 | 28.653751 | 130-134 |
| TTCCA | 8313115 | 2.3183582 | 21.722694 | 110-114 |
| CGGAT | 10391150 | 2.3100228 | 18.437384 | 20-24 |
| CTCCA | 12395625 | 2.295541 | 17.7368 | 95-99 |
| GGTCA | 10290925 | 2.2877421 | 12.52516 | 60-64 |
| TGGCC | 13850025 | 2.28459 | 11.717566 | 120-124 |
| GATTG | 6761445 | 2.2635612 | 18.608074 | 35-39 |
| CCTAC | 12203715 | 2.2600014 | 14.447999 | 90-94 |
| CACCG | 16713115 | 2.2518635 | 11.415022 | 45-49 |
| GAACG | 11317575 | 2.25165 | 13.378921 | 40-44 |
| GTGTG | 8270230 | 2.2508335 | 15.09148 | 135-137 |
| CCACG | 16654390 | 2.2439508 | 7.585205 | 85-89 |
| AATGA | 6085015 | 2.2425196 | 50.642834 | 8 |
| GCCAC | 16526825 | 2.2267632 | 12.645024 | 1 |
| GTTAA | 5401395 | 2.2242603 | 27.82191 | 130-134 |
| GCTCC | 14752315 | 2.2210066 | 11.330209 | 15-19 |
| CCAGC | 16263770 | 2.1913202 | 8.586618 | 100-104 |
| TACAA | 6435090 | 2.164518 | 26.26336 | 7 |
| ATTCG | 7080035 | 2.1633167 | 17.004131 | 90-94 |
| GGTGA | 8857165 | 2.1573243 | 16.489382 | 115-119 |
| ATTCA | 5683055 | 2.1359618 | 87.71255 | 6 |
| GGTCG | 11816860 | 2.135639 | 9.056382 | 9 |
| CCGAA | 11564740 | 2.0999815 | 11.382381 | 65-69 |
| ATTCC | 7523600 | 2.0981784 | 19.282494 | 110-114 |
| GTTGC | 8416620 | 2.0907183 | 13.025336 | 115-119 |
| GTGAT | 6238635 | 2.0885377 | 21.654922 | 120-124 |
| CGTTT | 6102355 | 2.08347 | 18.713343 | 45-49 |
| CGAGA | 10459535 | 2.0809417 | 28.714008 | 2 |
| GAAGC | 10377740 | 2.0646684 | 13.832923 | 60-64 |
| CTACA | 8240825 | 2.0567539 | 37.237595 | 6 |
| ATGGC | 9219560 | 2.0495706 | 17.613676 | 120-124 |
| CCTCC | 14749315 | 2.026719 | 12.388712 | 95-99 |
| AACTT | 5361400 | 2.015069 | 7.7601976 | 70-74 |
| TGGCG | 11124420 | 2.0104957 | 11.617147 | 70-74 |
| GATAC | 7346455 | 2.008896 | 15.343315 | 50-54 |
| CACCA | 12100455 | 2.0054567 | 11.269364 | 55-59 |
| ATGCA | 7317795 | 2.001059 | 17.264097 | 85-89 |
| GCTTC | 8802630 | 1.9957318 | 12.216831 | 100-104 |
| CTGAT | 6526380 | 1.9941466 | 20.158363 | 105-109 |
| TAAGA | 5403910 | 1.9915109 | 27.149368 | 130-134 |
| GGGAG | 11215020 | 1.9874159 | 10.908215 | 115-119 |
| CCTCG | 13125815 | 1.9761323 | 9.827638 | 20-24 |
| AACCT | 7912430 | 1.9747925 | 17.56319 | 105-109 |
| GACAT | 7186055 | 1.9650344 | 14.218351 | 105-109 |
| TTCGT | 5722575 | 1.9538056 | 19.83143 | 125-129 |
| AGAAC | 7979505 | 1.9527673 | 18.516232 | 40-44 |
| AACCC | 11761625 | 1.9493009 | 10.352277 | 90-94 |
| TGCAA | 7068370 | 1.9328535 | 18.292852 | 85-89 |
| AGATC | 7022870 | 1.9204113 | 15.776627 | 4 |
| GCACC | 14253045 | 1.9204025 | 10.17228 | 45-49 |
| AAGGC | 9644490 | 1.9187872 | 10.8582115 | 70-74 |
| TTTTT | 2696890 | 1.9058347 | 6.649924 | 110-114 |
| GTAGA | 6317440 | 1.8927305 | 19.588272 | 35-39 |
| CAGAA | 7714395 | 1.8878888 | 16.439388 | 60-64 |
| CGTTA | 6135205 | 1.8746222 | 21.049793 | 125-129 |
| TAGAG | 6227565 | 1.8658035 | 19.067137 | 40-44 |
| ACGCG | 12628060 | 1.8641862 | 10.127116 | 55-59 |
| TGGTG | 6832100 | 1.8594308 | 10.305927 | 130-134 |
| CGCGC | 16799305 | 1.840132 | 6.059796 | 55-59 |
| AGTCG | 8252355 | 1.8345546 | 19.404255 | 6 |
| AGATG | 6079525 | 1.8214501 | 12.45069 | 4 |
| AAGTC | 6647620 | 1.8177989 | 14.667942 | 10-14 |
| GCGGA | 11180455 | 1.8083401 | 11.210967 | 35-39 |
| GAAAT | 4899730 | 1.8057047 | 11.602557 | 4 |
| TGAAC | 6555715 | 1.7926674 | 22.132362 | 8 |
| CGGAG | 11078130 | 1.79179 | 10.348745 | 85-89 |
| ACCTG | 8790615 | 1.7836268 | 14.5208235 | 105-109 |
| TGAAG | 5930010 | 1.7766547 | 11.150571 | 7 |
| GCGGG | 13461290 | 1.770029 | 8.007595 | 9 |
| TTATT | 2784670 | 1.7611266 | 5.24261 | 3 |
| CATAC | 7046330 | 1.7586306 | 13.692219 | 105-109 |
| AGGGC | 10870800 | 1.758256 | 12.084111 | 30-34 |
| CGGGC | 14611190 | 1.7535223 | 13.783468 | 80-84 |
| GGGCC | 14609425 | 1.7533106 | 7.9362516 | 30-34 |
| CGGGA | 10809485 | 1.7483389 | 11.816074 | 110-114 |
| GCGTT | 7014360 | 1.7423918 | 13.571695 | 45-49 |
| AAGCG | 8682975 | 1.7274922 | 12.588343 | 65-69 |
| AACAG | 7054885 | 1.7264916 | 8.576546 | 9 |
| ACCCT | 9319295 | 1.7258369 | 11.559261 | 90-94 |
| CCATT | 6178825 | 1.7231482 | 64.09044 | 4 |
| AGGGT | 7067205 | 1.7213466 | 16.723404 | 115-119 |
| CAGCC | 12738785 | 1.7163768 | 10.889078 | 9 |
| AGCCA | 9434520 | 1.7131656 | 12.294906 | 100-104 |
| GGAGA | 7852980 | 1.7117883 | 11.781616 | 90-94 |
| CAAGA | 6987665 | 1.7100414 | 17.646124 | 9 |
| CCCTC | 12436210 | 1.7088728 | 9.504508 | 15-19 |
| TTACC | 6113775 | 1.7050072 | 18.886057 | 75-79 |
| TACCT | 6105755 | 1.7027705 | 17.025349 | 95-99 |
| GCAAC | 9318495 | 1.6920973 | 13.536744 | 85-89 |
| GCCAG | 11445915 | 1.6896747 | 8.726434 | 40-44 |
| AATTT | 2979180 | 1.6861975 | 15.39056 | 6 |
| GTCGC | 10201255 | 1.6827182 | 14.178072 | 8 |
| CCCCG | 16774600 | 1.677034 | 5.573592 | 30-34 |
| ATATT | 2952435 | 1.67106 | 8.488461 | 4 |
| CCACC | 13466635 | 1.6560585 | 10.307825 | 2 |
| TCCTC | 7942270 | 1.6434876 | 7.488468 | 4 |
| CTTGC | 7229885 | 1.6391593 | 12.943515 | 8 |
| ATACC | 6565625 | 1.6386555 | 13.081549 | 110-114 |
| AAATA | 3610510 | 1.6367037 | 6.1940126 | 105-109 |
| CCGGA | 11052100 | 1.631539 | 10.954012 | 50-54 |
| GGCCG | 13521650 | 1.6227642 | 8.052543 | 35-39 |
| GTCTA | 5302815 | 1.6202843 | 21.956799 | 4 |
| CGTGG | 8931705 | 1.6142102 | 13.473024 | 65-69 |
| TGAAT | 3905635 | 1.6083157 | 8.9215355 | 8 |
| GAACC | 8855880 | 1.6080934 | 14.684897 | 9 |
| TCGTC | 7087580 | 1.6068962 | 23.281921 | 2 |
| GCCAA | 8844900 | 1.6060996 | 13.738925 | 100-104 |
| CCGTA | 7828055 | 1.588322 | 13.406662 | 35-39 |
| TCACC | 8487660 | 1.5718266 | 9.959549 | 65-69 |
| AAGGG | 7175900 | 1.5641987 | 14.602086 | 30-34 |
| GGCGG | 11866105 | 1.5602776 | 6.6987715 | 8 |
| TCTAC | 5539715 | 1.5449135 | 20.117847 | 5 |
| AGGCC | 10406080 | 1.5361717 | 7.9499817 | 70-74 |
| TCCAC | 8255620 | 1.5288552 | 7.158581 | 75-79 |
| GAGAG | 7009975 | 1.5280305 | 5.130686 | 100-104 |
| ACTTC | 5470165 | 1.5255176 | 5.3238454 | 70-74 |
| TCGCC | 10111860 | 1.5223721 | 8.010231 | 9 |
| GCCGT | 9219815 | 1.5208274 | 10.722862 | 1 |
| CAGGG | 9386800 | 1.5182322 | 11.519831 | 115-119 |
| CGCTC | 10084285 | 1.5182204 | 10.748429 | 15-19 |
| CGCCT | 10063650 | 1.5151136 | 8.708808 | 75-79 |
| ACAAG | 6182070 | 1.5128938 | 17.699844 | 8 |
| TACGC | 7453405 | 1.512305 | 12.659887 | 55-59 |
| CCCGC | 15092100 | 1.5088266 | 5.688695 | 30-34 |
| TTGAA | 3657515 | 1.5061415 | 11.920999 | 9 |
| CTACC | 8102575 | 1.5005128 | 11.9392395 | 90-94 |
| CCCTA | 8099745 | 1.4999887 | 13.105462 | 90-94 |
| CTATA | 3947270 | 1.4835716 | 7.0704145 | 60-64 |
| TCGAA | 5419560 | 1.4819845 | 5.236663 | 10-14 |
| CATTC | 5299630 | 1.4779588 | 65.03141 | 5 |
| CTACG | 7263445 | 1.4737619 | 11.797985 | 75-79 |
| TAACT | 3916265 | 1.4719183 | 8.165872 | 70-74 |
| TGCCT | 6477645 | 1.4686117 | 10.078434 | 120-124 |
| AACGC | 8075995 | 1.4664781 | 11.146913 | 45-49 |
| CCTGA | 7225750 | 1.4661136 | 15.7753105 | 105-109 |
| GTGGT | 5381725 | 1.4646953 | 9.857148 | 130-134 |
| TACGG | 6583390 | 1.4635321 | 11.526711 | 80-84 |
| GGGCA | 9028420 | 1.4602674 | 10.818686 | 80-84 |
| CCCTT | 7038225 | 1.4564142 | 8.778501 | 125-129 |
| CCTTC | 7018420 | 1.4523159 | 8.480994 | 125-129 |
| CCAAC | 8738730 | 1.4483045 | 11.0279 | 100-104 |
| GGTGT | 5310030 | 1.4451828 | 10.136891 | 130-134 |
| TCCGA | 7117955 | 1.4442419 | 5.024791 | 45-49 |
| CCGTT | 6336190 | 1.4365408 | 17.714478 | 125-129 |
| GTCAC | 7067780 | 1.4340613 | 11.333962 | 60-64 |
| GGCAT | 6443325 | 1.4323949 | 16.661005 | 80-84 |
| CATGC | 7047850 | 1.4300175 | 12.65591 | 85-89 |
| CCAGA | 7872140 | 1.4294611 | 11.680652 | 60-64 |
| AGAGG | 6547460 | 1.4272118 | 5.172372 | 100-104 |
| TCTGA | 4670650 | 1.4271251 | 6.5799212 | 15-19 |
| GCGGT | 7790270 | 1.4079208 | 11.42346 | 60-64 |
| GCATG | 6329115 | 1.4070051 | 14.597245 | 80-84 |
| TATAC | 3743250 | 1.4068911 | 8.236974 | 60-64 |
| CTCGG | 8454795 | 1.3946358 | 10.713362 | 20-24 |
| CTCCT | 6670585 | 1.3803388 | 7.734351 | 3 |
| GCCCC | 13787700 | 1.3784198 | 5.656761 | 30-34 |
| CCGGG | 11458070 | 1.3751092 | 7.818671 | 80-84 |
| TAGTC | 4498770 | 1.3746068 | 16.126936 | 6 |
| CGCAC | 10195595 | 1.3737167 | 11.794231 | 45-49 |
| CTCTC | 6598660 | 1.3654554 | 8.732749 | 1 |
| TAACA | 4038780 | 1.358491 | 9.829678 | 8 |
| ACCTC | 7301265 | 1.3521186 | 11.775654 | 95-99 |
| GCCTC | 8975950 | 1.3513572 | 6.242116 | 120-124 |
| GTAGT | 4032060 | 1.349832 | 7.0073175 | 80-84 |
| CTCCC | 9785510 | 1.3446374 | 9.170866 | 15-19 |
| GACGC | 9081230 | 1.340594 | 12.041844 | 50-54 |
| CAAGG | 6702795 | 1.3335323 | 12.52955 | 30-34 |
| ATGCG | 5988645 | 1.3313165 | 5.675916 | 55-59 |
| CAAGT | 4847125 | 1.3254516 | 63.47788 | 9 |
| TAAAT | 2604340 | 1.3191806 | 7.542948 | 105-109 |
| TCTCC | 6355650 | 1.3151696 | 7.948145 | 2 |
| CTGAA | 4795090 | 1.3112226 | 5.760329 | 20-24 |
| ACCAG | 7202150 | 1.3078012 | 11.331621 | 60-64 |
| ACAGC | 7172095 | 1.3023437 | 15.6472 | 8 |
| GGGTG | 6567105 | 1.3003724 | 15.034743 | 115-119 |
| CCGCC | 12893830 | 1.2890555 | 5.4008226 | 75-79 |
| CGTAG | 5797685 | 1.2888644 | 16.487404 | 35-39 |
| GTCGT | 5180195 | 1.2867789 | 19.168896 | 1 |
| CACGG | 8708510 | 1.2855723 | 7.6386204 | 85-89 |
| TTTGG | 3409080 | 1.2752491 | 5.8795066 | 95-99 |
| GGCGC | 10625715 | 1.2752163 | 9.025186 | 70-74 |
| TTGGT | 3406295 | 1.2742074 | 6.177703 | 7 |
| AAAGA | 3857170 | 1.2721498 | 12.577016 | 135-137 |
| GGACG | 7854760 | 1.2704382 | 12.682656 | 50-54 |
| ACGAA | 5190765 | 1.2702988 | 6.653743 | 125-129 |
| ACAGT | 4606930 | 1.25977 | 5.7924185 | 15-19 |
| AAAAG | 3818290 | 1.2593266 | 20.729523 | 135-137 |
| GAATA | 3411085 | 1.2570921 | 8.926524 | 15-19 |
| TGAGA | 4189350 | 1.2551461 | 5.032841 | 130-134 |
| TGTGC | 5033375 | 1.2503082 | 13.717691 | 135-137 |
| GCGTG | 6905045 | 1.2479358 | 11.911779 | 65-69 |
| CAGTC | 6133200 | 1.2444339 | 8.339782 | 5 |
| ACGGA | 6239315 | 1.2413222 | 10.450904 | 85-89 |
| GCCTA | 6057380 | 1.2290499 | 10.478163 | 75-79 |
| ACGTT | 4016450 | 1.2272332 | 9.562309 | 135-137 |
| AAATT | 2418850 | 1.225224 | 14.3119545 | 5 |
| GCTAT | 3976785 | 1.2151134 | 5.599013 | 60-64 |
| CAACA | 5367825 | 1.1989605 | 5.4438314 | 10-14 |
| ATAAA | 2627765 | 1.1912092 | 6.0936794 | 105-109 |
| TTAGT | 2585335 | 1.1896019 | 24.57367 | 5 |
| CGACA | 6548320 | 1.1890756 | 9.878525 | 105-109 |
| CGCGA | 8041000 | 1.1870327 | 8.06584 | 100-104 |
| CGTCT | 5220495 | 1.1835904 | 17.116774 | 3 |
| ACCGA | 6469035 | 1.1746787 | 9.382858 | 65-69 |
| GTCCC | 7758700 | 1.1680965 | 35.20453 | 1 |
| AGTAG | 3894880 | 1.1669216 | 6.145636 | 80-84 |
| ACCGC | 8650385 | 1.1655208 | 10.521499 | 10-14 |
| GCGAC | 7889875 | 1.1647232 | 7.8560452 | 105-109 |
| ATCAT | 3046475 | 1.1450101 | 5.728343 | 50-54 |
| AGTCT | 3746435 | 1.1447296 | 6.9654336 | 15-19 |
| AATAG | 3096715 | 1.1412369 | 5.9795833 | 15-19 |
| ATTTG | 2475740 | 1.1391733 | 12.949425 | 7 |
| TCGGA | 5082815 | 1.1299443 | 14.619484 | 20-24 |
| CCAGG | 7649810 | 1.1292841 | 9.645396 | 115-119 |
| CACCT | 6084990 | 1.1268771 | 14.729144 | 3 |
| GCAGT | 5065365 | 1.1260649 | 9.223513 | 4 |
| GTGCA | 5063395 | 1.1256269 | 12.55983 | 135-137 |
| TTATC | 2670550 | 1.1215471 | 6.668807 | 50-54 |
| TACAG | 4083755 | 1.1167072 | 21.756035 | 7 |
| CGTAA | 4079060 | 1.1154233 | 7.582568 | 6 |
| CACGT | 5471995 | 1.1102746 | 5.5206323 | 135-137 |
| TCGTG | 4442840 | 1.1036173 | 9.658154 | 130-134 |
| GGTTC | 4442290 | 1.1034806 | 5.9304194 | 65-69 |
| GTGGC | 6101820 | 1.1027704 | 11.78157 | 65-69 |
| CGCCA | 8184340 | 1.1027277 | 8.763073 | 55-59 |
| CACTA | 4380995 | 1.0934135 | 5.205493 | 70-74 |
| GCCCG | 9929120 | 1.0875981 | 10.026684 | 125-129 |
| TATTG | 2363500 | 1.0875278 | 6.3633537 | 5 |
| ACGGG | 6648215 | 1.0752902 | 8.735946 | 80-84 |
| GTCAG | 4816405 | 1.0707196 | 9.087256 | 1 |
| CTGCG | 6442330 | 1.0626757 | 8.204488 | 1 |
| TACGT | 3476460 | 1.0622383 | 5.385601 | 60-64 |
| TTGGA | 3170400 | 1.06137 | 6.409671 | 95-99 |
| TGCCC | 7035235 | 1.0591766 | 8.014355 | 30-34 |
| TGCGC | 6373675 | 1.051351 | 8.092976 | 2 |
| AGGTT | 3122270 | 1.0452572 | 7.42561 | 65-69 |
| GTAAC | 3799330 | 1.0389309 | 7.865825 | 7 |
| GAGTA | 3467490 | 1.0388739 | 5.989879 | 75-79 |
| GGATG | 4230920 | 1.0305179 | 5.0868535 | 55-59 |
| TCGGT | 4145440 | 1.029742 | 8.649342 | 7 |
| GCGCG | 8549110 | 1.0259982 | 6.7472363 | 55-59 |
| TATCA | 2703850 | 1.0162352 | 6.753908 | 50-54 |
| CGGAC | 6815460 | 1.0061153 | 10.285205 | 50-54 |
| CAGCG | 6771345 | 0.9996031 | 8.073424 | 45-49 |
| ACGCA | 5492830 | 0.9974147 | 11.306924 | 45-49 |
| CCGCG | 9100885 | 0.9968764 | 6.4575453 | 30-34 |
| TCCCA | 5382080 | 0.99670535 | 41.626755 | 2 |
| GGGAT | 4038090 | 0.9835506 | 5.05922 | 50-54 |
| ACGCC | 7263485 | 0.9786551 | 9.684653 | 55-59 |
| CCCAT | 5272530 | 0.97641784 | 42.6002 | 3 |
| GTCTG | 3910340 | 0.9713424 | 5.1563864 | 15-19 |
| GCTGG | 5333860 | 0.9639785 | 9.509298 | 5 |
| CCGCT | 6261955 | 0.9427568 | 9.700108 | 10-14 |
| AGCTG | 4184370 | 0.93021375 | 5.5892367 | 50-54 |
| ACGAG | 4650055 | 0.9251362 | 5.1196575 | 30-34 |
| ACTAC | 3675995 | 0.9174588 | 5.0210533 | 70-74 |
| GCGCA | 6164160 | 0.9099688 | 6.5115614 | 2 |
| GGCGA | 5585485 | 0.903403 | 12.735058 | 1 |
| GCCGC | 8241110 | 0.9026999 | 6.130786 | 70-74 |
| TATAT | 1558610 | 0.88216364 | 6.8705616 | 3 |
| GATGA | 2942650 | 0.8816298 | 10.344634 | 5 |
| TTCGA | 2881295 | 0.8803846 | 5.2154703 | 40-44 |
| CAGAT | 3194145 | 0.8734424 | 10.064871 | 3 |
| AACCG | 4809675 | 0.873364 | 12.184916 | 9 |
| TTATA | 1541765 | 0.8726295 | 7.003171 | 7 |
| GAGGT | 3564470 | 0.86819166 | 5.289087 | 65-69 |
| CTGGC | 5091215 | 0.8398065 | 8.426625 | 6 |
| GTCCA | 4122975 | 0.8365568 | 5.1295667 | 60-64 |
| GCGAG | 5079160 | 0.8215094 | 22.256329 | 1 |
| GGCCC | 7371445 | 0.80744016 | 7.2698336 | 125-129 |
| ACTCT | 2892760 | 0.8067318 | 5.3222737 | 5 |
| ATCTA | 2129475 | 0.8003579 | 5.0153522 | 100-104 |
| CCCGT | 5272470 | 0.79378676 | 10.518532 | 125-129 |
| TGCGG | 4287250 | 0.77482677 | 5.6822114 | 50-54 |
| ACCTA | 3063090 | 0.7644893 | 19.189894 | 4 |
| GGGCG | 5810970 | 0.7640861 | 8.114846 | 2 |
| ATAGC | 2794095 | 0.7640482 | 6.5844703 | 20-24 |
| CTTAT | 1793455 | 0.75319475 | 5.277282 | 1 |
| GTTAG | 2213470 | 0.74101394 | 17.602348 | 4 |
| GAATG | 2472600 | 0.74080086 | 6.358394 | 9 |
| CGCTG | 4468255 | 0.73704785 | 8.311126 | 4 |
| GTCGG | 4035810 | 0.7293845 | 5.635428 | 1 |
| TTCGG | 2903385 | 0.72121114 | 6.477592 | 70-74 |
| TCAGA | 2565595 | 0.70156467 | 9.655682 | 2 |
| TCTAA | 1861215 | 0.69953305 | 5.859836 | 100-104 |
| TGACT | 2141710 | 0.65440315 | 5.303099 | 3 |
| CGAAT | 2363335 | 0.64625645 | 5.972725 | 15-19 |
| CTCGT | 2804775 | 0.6358985 | 9.446037 | 1 |
| CGCAG | 3698965 | 0.54605055 | 5.953612 | 3 |
| AGCGC | 3664235 | 0.5409236 | 6.2323885 | 1 |
| GCAGA | 2680705 | 0.5333307 | 5.5898204 | 1 |
| AAGCC | 2558755 | 0.46463108 | 6.216067 | 9 |

Produced by FastQC (version 0.10.1)
