# Supplementary material for: Freshwater sponge hosts and their green algae symbionts: a tractable model to understand intracellular symbiosis
Source: PeerJ. 2021 Feb 11;9:e10654. doi: 10.7717/peerj.10654 (PMC7882143; doi:10.7717/peerj.10654)
Supplement: Supplemental Information 29 [file peerj-09-10654-s029.zip › EmApo3_Clean_Data2.fq_fastqc/fastqc_report.html]

EmApo3\_Clean\_Data2.fq.gz FastQC Report


FastQC Report

Tue 10 Sep 2019  
EmApo3\_Clean\_Data2.fq.gz

## Summary

- Basic Statistics
- Per base sequence quality
- Per sequence quality scores
- Per base sequence content
- Per base GC content
- Per sequence GC content
- Per base N content
- Sequence Length Distribution
- Sequence Duplication Levels
- Overrepresented sequences
- Kmer Content

## Basic Statistics

| Measure | Value |
| --- | --- |
| Filename | EmApo3\_Clean\_Data2.fq.gz |
| File type | Conventional base calls |
| Encoding | Sanger / Illumina 1.9 |
| Total Sequences | 32861912 |
| Filtered Sequences | 0 |
| Sequence length | 100-141 |
| %GC | 58 |

## Per base sequence quality

## Per sequence quality scores

## Per base sequence content

## Per base GC content

## Per sequence GC content

## Per base N content

## Sequence Length Distribution

## Sequence Duplication Levels

## Overrepresented sequences

| Sequence | Count | Percentage | Possible Source |
| --- | --- | --- | --- |
| CTCGGAGACGCCGGAGGGGACCCTGGGAAGAGTTCTCTTTTCTTCTTAAC | 907240 | 2.76076449842602 | No Hit |
| GGCAACTCCCGGTATGTCGCGAAGCGCGAATCTCCGTGGCCCGTAGGCGG | 624449 | 1.9002211435536678 | No Hit |
| CGGAGACGCCGGAGGGGACCCTGGGAAGAGTTCTCTTTTCTTCTTAACGG | 553184 | 1.6833591423408352 | No Hit |
| GTTTCGACGTGCCGGCACGCCGGCGAGGACTTCGGCCCTCGCAGGCGTAG | 460617 | 1.401674376098384 | No Hit |
| GCAGGTGCACACCACGAAGGGAGGCAACTCCCGGTATGTCGCGAAGCGCG | 450922 | 1.3721721365451895 | No Hit |
| GTGCACACCACGAAGGGAGGCAACTCCCGGTATGTCGCGAAGCGCGAATC | 438180 | 1.3333977645609907 | No Hit |
| GGTGCACACCACGAAGGGAGGCAACTCCCGGTATGTCGCGAAGCGCGAAT | 408058 | 1.2417354169775636 | No Hit |
| CAGGTTTCGACGTGCCGGCACGCCGGCGAGGACTTCGGCCCTCGCAGGCG | 337151 | 1.0259628228570512 | No Hit |
| CACGAAGGGAGGCAACTCCCGGTATGTCGCGAAGCGCGAATCTCCGTGGC | 328091 | 0.9983929115262679 | No Hit |
| GGGACGTATAGCCGCGTCGTTCGGAGCGCGCCCGCGACCGAGGAGAGGGT | 276452 | 0.8412535460505158 | No Hit |
| AGCATATGTAGCCAGGCGTCGCCCCGCGTGAGGTTCAGGTTTCGACGTGC | 266427 | 0.8107471044289815 | No Hit |
| GCCAGGCGTCGCCCCGCGTGAGGTTCAGGTTTCGACGTGCCGGCACGCCG | 227260 | 0.691560491063332 | No Hit |
| GGGAAGAGTTCTCTTTTCTTCTTAACGGGCCATCACCCTGGAATCAGGTT | 215567 | 0.6559782644418255 | No Hit |
| GGAGACGCCGGAGGGGACCCTGGGAAGAGTTCTCTTTTCTTCTTAACGGG | 206307 | 0.627799745796897 | No Hit |
| CGACGTGCCGGCACGCCGGCGAGGACTTCGGCCCTCGCAGGCGTAGCCGA | 198688 | 0.6046148501645309 | No Hit |
| GGAAGCTCCCTGTAGCACGGTGCAACTCGCCATCTTGGCGACCGGCACCC | 191145 | 0.58166122531154 | No Hit |
| GCCGGAGGGGACCCTGGGAAGAGTTCTCTTTTCTTCTTAACGGGCCATCA | 184434 | 0.5612394068853936 | No Hit |
| GGCGTCGCCCCGCGTGAGGTTCAGGTTTCGACGTGCCGGCACGCCGGCGA | 175639 | 0.534475900245853 | No Hit |
| GACGTATAGCCGCGTCGTTCGGAGCGCGCCCGCGACCGAGGAGAGGGTCT | 170546 | 0.5189777149911423 | No Hit |
| GGAAGAGTTCTCTTTTCTTCTTAACGGGCCATCACCCTGGAATCAGGTTG | 170291 | 0.5182017406656071 | No Hit |
| GGCTGCAGGTGCACACCACGAAGGGAGGCAACTCCCGGTATGTCGCGAAG | 159116 | 0.48419580698773707 | No Hit |
| CTCTTTTCTTCTTAACGGGCCATCACCCTGGAATCAGGTTGGCTGGAGGT | 153940 | 0.46844504969765605 | No Hit |
| CACATTTCCCCGCGGGCTGCAGGTGCACACCACGAAGGGAGGCAACTCCC | 147238 | 0.4480506186006462 | No Hit |
| GGGAAGCATATGTAGCCAGGCGTCGCCCCGCGTGAGGTTCAGGTTTCGAC | 146226 | 0.4449710655910709 | No Hit |
| GGACGTATAGCCGCGTCGTTCGGAGCGCGCCCGCGACCGAGGAGAGGGTC | 141848 | 0.43164865148442977 | No Hit |
| GTGCCGGCACGCCGGCGAGGACTTCGGCCCTCGCAGGCGTAGCCGACCGC | 137534 | 0.41852099171831514 | No Hit |
| GACGTGCCGGCACGCCGGCGAGGACTTCGGCCCTCGCAGGCGTAGCCGAC | 135360 | 0.4119054302135554 | No Hit |
| GTCGGAAGCGAGGGTCGACGAAGCGGGCTGGCGGGGGGGCCCTCTCGGGG | 133394 | 0.4059228203155069 | No Hit |
| TGCAGGTGCACACCACGAAGGGAGGCAACTCCCGGTATGTCGCGAAGCGC | 131480 | 0.4000984483191362 | No Hit |
| GTTCAGGTTTCGACGTGCCGGCACGCCGGCGAGGACTTCGGCCCTCGCAG | 128306 | 0.39043985024364986 | No Hit |
| CGTGCCGGCACGCCGGCGAGGACTTCGGCCCTCGCAGGCGTAGCCGACCG | 127250 | 0.3872264036249626 | No Hit |
| GTCCCGACTTTGCGGAAGGGATGTATTTATTAGATCCAAAGCCAATGCGG | 127162 | 0.38695861640673856 | No Hit |
| GCTGCAGGTGCACACCACGAAGGGAGGCAACTCCCGGTATGTCGCGAAGC | 118772 | 0.3614275395783423 | No Hit |
| GTTCTCTTTTCTTCTTAACGGGCCATCACCCTGGAATCAGGTTGGCTGGA | 114470 | 0.3483363962510763 | No Hit |
| CCGGTATGTCGCGAAGCGCGAATCTCCGTGGCCCGTAGGCGGCCTTCGGT | 113379 | 0.3450164433524136 | No Hit |
| TCGGAGACGCCGGAGGGGACCCTGGGAAGAGTTCTCTTTTCTTCTTAACG | 112061 | 0.3410057211521959 | No Hit |
| AGGCAACTCCCGGTATGTCGCGAAGCGCGAATCTCCGTGGCCCGTAGGCG | 108051 | 0.32880314450358217 | No Hit |
| CCCCGCGTGAGGTTCAGGTTTCGACGTGCCGGCACGCCGGCGAGGACTTC | 106119 | 0.32292399784893827 | No Hit |
| GAGACGCCGGAGGGGACCCTGGGAAGAGTTCTCTTTTCTTCTTAACGGGC | 103619 | 0.3153164064221218 | No Hit |
| CATTTCCCCGCGGGCTGCAGGTGCACACCACGAAGGGAGGCAACTCCCGG | 100896 | 0.3070302178400332 | No Hit |
| GAAGCGGGCTGGCGGGGGGGCCCTCTCGGGGGTCCTGCCGCCGGAGCGTG | 100572 | 0.3060442739911178 | No Hit |
| GGCGAAGTTAGGGACGTATAGCCGCGTCGTTCGGAGCGCGCCCGCGACCG | 99621 | 0.30315034621235676 | No Hit |
| GAAGAGTTCTCTTTTCTTCTTAACGGGCCATCACCCTGGAATCAGGTTGG | 96505 | 0.29366824425797255 | No Hit |
| GGGGAAGCTCCCTGTAGCACGGTGCAACTCGCCATCTTGGCGACCGGCAC | 94625 | 0.2879473355050065 | No Hit |
| CCGGAGGGGACCCTGGGAAGAGTTCTCTTTTCTTCTTAACGGGCCATCAC | 88542 | 0.26943654404527645 | No Hit |
| TTCGACGTGCCGGCACGCCGGCGAGGACTTCGGCCCTCGCAGGCGTAGCC | 87276 | 0.2655840597467366 | No Hit |
| CGTATAGCCGCGTCGTTCGGAGCGCGCCCGCGACCGAGGAGAGGGTCTCT | 86273 | 0.2625318940662978 | No Hit |
| CGCAACGACACATTTCCCCGCGGGCTGCAGGTGCACACCACGAAGGGAGG | 85433 | 0.25997574334688744 | No Hit |
| CCCGCGTGAGGTTCAGGTTTCGACGTGCCGGCACGCCGGCGAGGACTTCG | 84876 | 0.25828077197699273 | No Hit |
| GCAACTCCCGGTATGTCGCGAAGCGCGAATCTCCGTGGCCCGTAGGCGGC | 84685 | 0.2576995519919839 | No Hit |
| CCCGACTTTGCGGAAGGGATGTATTTATTAGATCCAAAGCCAATGCGGGG | 84160 | 0.25610195779235245 | No Hit |
| ATTTCCCCGCGGGCTGCAGGTGCACACCACGAAGGGAGGCAACTCCCGGT | 82865 | 0.25216122543326147 | No Hit |
| CTGGGAAGAGTTCTCTTTTCTTCTTAACGGGCCATCACCCTGGAATCAGG | 80436 | 0.2447696896029665 | No Hit |
| GTCGACGAAGCGGGCTGGCGGGGGGGCCCTCTCGGGGGTCCTGCCGCCGG | 79284 | 0.24126411147348945 | No Hit |
| CGGAGGGGACCCTGGGAAGAGTTCTCTTTTCTTCTTAACGGGCCATCACC | 79206 | 0.24102675462097273 | No Hit |
| ACGAAGGGAGGCAACTCCCGGTATGTCGCGAAGCGCGAATCTCCGTGGCC | 78194 | 0.2379472016113974 | No Hit |
| AGCGAGGGTCGACGAAGCGGGCTGGCGGGGGGGCCCTCTCGGGGGTCCTG | 75910 | 0.23099690608385778 | No Hit |
| GGCGAATTGTAGCCGAGAGAGGCACCTGCGCTCGGCAGGCGGTCGACCAA | 74523 | 0.22677621436026 | No Hit |
| CGAAGGGAGGCAACTCCCGGTATGTCGCGAAGCGCGAATCTCCGTGGCCC | 73898 | 0.22487431650355585 | No Hit |
| CAGGTGCACACCACGAAGGGAGGCAACTCCCGGTATGTCGCGAAGCGCGA | 72879 | 0.2217734622379854 | No Hit |
| CACCACGAAGGGAGGCAACTCCCGGTATGTCGCGAAGCGCGAATCTCCGT | 72774 | 0.22145394339805913 | No Hit |
| GCCCTGACCCGCCTCTCGGGGCGAAGTTAGGGACGTATAGCCGCGTCGTT | 68910 | 0.20969565008877147 | No Hit |
| AAGCGAGGGTCGACGAAGCGGGCTGGCGGGGGGGCCCTCTCGGGGGTCCT | 68214 | 0.20757769663554576 | No Hit |
| GGGAGGCAACTCCCGGTATGTCGCGAAGCGCGAATCTCCGTGGCCCGTAG | 68089 | 0.2071973170642049 | No Hit |
| TTTCGACGTGCCGGCACGCCGGCGAGGACTTCGGCCCTCGCAGGCGTAGC | 67498 | 0.20539888245090548 | No Hit |
| CTTTTCTTCTTAACGGGCCATCACCCTGGAATCAGGTTGGCTGGAGGTAG | 67363 | 0.20498807251385737 | No Hit |
| GTCTCTTCGACCCGCCAGCGCAGGCCTTCGTGGCCGGAGCTCCCGCGTTC | 65637 | 0.19973579139278322 | No Hit |
| GGCGGTGCTGTTACGGCGACCGGGTGGTGCCCTGACCCGCCTCTCGGGGC | 64265 | 0.19556074521774633 | No Hit |
| CTGGAATCAGGTTGGCTGGAGGTAGGGTTGCATGCCCGGTAAAGCGCCAC | 64219 | 0.19542076553549287 | No Hit |
| GTCGCGAAGCGCGAATCTCCGTGGCCCGTAGGCGGCCTTCGGTGACCGCG | 62456 | 0.19005589206130186 | No Hit |
| AGACGCCGGAGGGGACCCTGGGAAGAGTTCTCTTTTCTTCTTAACGGGCC | 61925 | 0.188440039642246 | No Hit |
| GACGCCGGAGGGGACCCTGGGAAGAGTTCTCTTTTCTTCTTAACGGGCCA | 60960 | 0.18550350935149482 | No Hit |
| CCTGGGAAGAGTTCTCTTTTCTTCTTAACGGGCCATCACCCTGGAATCAG | 60098 | 0.18288041182752848 | No Hit |
| CCCTGACCCGCCTCTCGGGGCGAAGTTAGGGACGTATAGCCGCGTCGTTC | 57466 | 0.174871139573376 | No Hit |
| CAACTCCCGGTATGTCGCGAAGCGCGAATCTCCGTGGCCCGTAGGCGGCC | 55832 | 0.1698988178168087 | No Hit |
| AAGCATATGTAGCCAGGCGTCGCCCCGCGTGAGGTTCAGGTTTCGACGTG | 54629 | 0.16623804482222457 | No Hit |
| CCCGGTATGTCGCGAAGCGCGAATCTCCGTGGCCCGTAGGCGGCCTTCGG | 53177 | 0.16181955572152953 | No Hit |
| GCATATGTAGCCAGGCGTCGCCCCGCGTGAGGTTCAGGTTTCGACGTGCC | 53158 | 0.16176173802668573 | No Hit |
| GGCGTGTGCCTGTAACCGTAGTGAATCAACGGGGCTTGATCTGGCGAATA | 52795 | 0.16065711575151195 | No Hit |
| GAGGCAACTCCCGGTATGTCGCGAAGCGCGAATCTCCGTGGCCCGTAGGC | 51418 | 0.1564668543936214 | No Hit |
| GCGAGGGTCGACGAAGCGGGCTGGCGGGGGGGCCCTCTCGGGGGTCCTGC | 51385 | 0.15636643418678742 | No Hit |
| CTTCGACCCGCCAGCGCAGGCCTTCGTGGCCGGAGCTCCCGCGTTCCGGT | 51116 | 0.15554785734926196 | No Hit |
| GGGGACCCTGGGAAGAGTTCTCTTTTCTTCTTAACGGGCCATCACCCTGG | 50990 | 0.15516443474135042 | No Hit |
| CTCTTCGACCCGCCAGCGCAGGCCTTCGTGGCCGGAGCTCCCGCGTTCCG | 50845 | 0.15472319443859506 | No Hit |
| AGCCAGGCGTCGCCCCGCGTGAGGTTCAGGTTTCGACGTGCCGGCACGCC | 48364 | 0.14717342070662232 | No Hit |
| GCCCCGCGTGAGGTTCAGGTTTCGACGTGCCGGCACGCCGGCGAGGACTT | 48114 | 0.14641266156394064 | No Hit |
| GTCGATTCAGACATTTGGCATTTGCGCTTGGCTGAAAAGCCAATGGCGCG | 47512 | 0.14458075354836322 | No Hit |
| GGAGGGGACCCTGGGAAGAGTTCTCTTTTCTTCTTAACGGGCCATCACCC | 46454 | 0.14136122085653446 | No Hit |
| GGACCCTGGGAAGAGTTCTCTTTTCTTCTTAACGGGCCATCACCCTGGAA | 45997 | 0.1399705531437124 | No Hit |
| GAAGCTCCCTGTAGCACGGTGCAACTCGCCATCTTGGCGACCGGCACCCA | 45457 | 0.13832731339552 | No Hit |
| GCCGCGTCGTTCGGAGCGCGCCCGCGACCGAGGAGAGGGTCTCTTCGACC | 45427 | 0.1382360222983982 | No Hit |
| CATGCAACAAGTCCCGACTTTGCGGAAGGGATGTATTTATTAGATCCAAA | 45143 | 0.13737179991231185 | No Hit |
| TGGGAAGAGTTCTCTTTTCTTCTTAACGGGCCATCACCCTGGAATCAGGT | 44851 | 0.13648323323365968 | No Hit |
| AGCGGGCTGGCGGGGGGGCCCTCTCGGGGGTCCTGCCGCCGGAGCGTGGA | 44001 | 0.13389665214854207 | No Hit |
| AAGCGGGCTGGCGGGGGGGCCCTCTCGGGGGTCCTGCCGCCGGAGCGTGG | 43836 | 0.13339455111437215 | No Hit |
| GCAACAAGTCCCGACTTTGCGGAAGGGATGTATTTATTAGATCCAAAGCC | 42374 | 0.12894563164796985 | No Hit |
| CGTCGCCCCGCGTGAGGTTCAGGTTTCGACGTGCCGGCACGCCGGCGAGG | 42171 | 0.12832789522411234 | No Hit |
| GAAGCATATGTAGCCAGGCGTCGCCCCGCGTGAGGTTCAGGTTTCGACGT | 42048 | 0.12795360172591297 | No Hit |
| GGGACCCTGGGAAGAGTTCTCTTTTCTTCTTAACGGGCCATCACCCTGGA | 41414 | 0.1260243165400723 | No Hit |
| CCTGACCCGCCTCTCGGGGCGAAGTTAGGGACGTATAGCCGCGTCGTTCG | 40739 | 0.12397026685483181 | No Hit |
| GGCCAACGTGGGTTGCGGGCGGTGCTGTTACGGCGACCGGGTGGTGCCCT | 40238 | 0.1224457055328978 | No Hit |
| GACACATTTCCCCGCGGGCTGCAGGTGCACACCACGAAGGGAGGCAACTC | 40142 | 0.12215357402210804 | No Hit |
| CTCGGGGCGAAGTTAGGGACGTATAGCCGCGTCGTTCGGAGCGCGCCCGC | 39809 | 0.12114024284405607 | No Hit |
| GCACACCACGAAGGGAGGCAACTCCCGGTATGTCGCGAAGCGCGAATCTC | 38684 | 0.11771682670198862 | No Hit |
| TTCCCCGCGGGCTGCAGGTGCACACCACGAAGGGAGGCAACTCCCGGTAT | 38252 | 0.1164022349034347 | No Hit |
| GCCGAGAGAGGCACCTGCGCTCGGCAGGCGGTCGACCAAAGTTGACCTGG | 38204 | 0.11625616914803985 | No Hit |
| GTAATTCTAGAGCTAATACATGCAACAAGTCCCGACTTTGCGGAAGGGAT | 37998 | 0.11562930361447014 | No Hit |
| GCGTGAGGTTCAGGTTTCGACGTGCCGGCACGCCGGCGAGGACTTCGGCC | 37457 | 0.11398302082970704 | No Hit |
| CGCCCCGCGTGAGGTTCAGGTTTCGACGTGCCGGCACGCCGGCGAGGACT | 37432 | 0.11390694491543887 | No Hit |
| TCGACGTGCCGGCACGCCGGCGAGGACTTCGGCCCTCGCAGGCGTAGCCG | 37019 | 0.11265017081172879 | No Hit |
| CGGCGACCGGGTGGTGCCCTGACCCGCCTCTCGGGGCGAAGTTAGGGACG | 36763 | 0.11187115344962277 | No Hit |
| GTAGCCAGGCGTCGCCCCGCGTGAGGTTCAGGTTTCGACGTGCCGGCACG | 36509 | 0.11109822216065822 | No Hit |
| CTTTGCGGAAGGGATGTATTTATTAGATCCAAAGCCAATGCGGGGGGCAA | 36276 | 0.11038919463967892 | No Hit |
| CTCCGGCGCACAGCCGGCGAATTGTAGCCGAGAGAGGCACCTGCGCTCGG | 36006 | 0.10956757476558272 | No Hit |
| CAGGCGTCGCCCCGCGTGAGGTTCAGGTTTCGACGTGCCGGCACGCCGGC | 35421 | 0.10778739837170766 | No Hit |
| CGAAGCGGGCTGGCGGGGGGGCCCTCTCGGGGGTCCTGCCGCCGGAGCGT | 35086 | 0.10676798112051424 | No Hit |
| CCCCGCGGGCTGCAGGTGCACACCACGAAGGGAGGCAACTCCCGGTATGT | 35026 | 0.10658539892627063 | No Hit |
| GTATAGCCGCGTCGTTCGGAGCGCGCCCGCGACCGAGGAGAGGGTCTCTT | 34958 | 0.10637847243946122 | No Hit |
| AAATCTCCGGCGCACAGCCGGCGAATTGTAGCCGAGAGAGGCACCTGCGC | 34414 | 0.10472306054498594 | No Hit |
| AAGGGAGGCAACTCCCGGTATGTCGCGAAGCGCGAATCTCCGTGGCCCGT | 34121 | 0.10383145082976304 | No Hit |
| GGTTTCGACGTGCCGGCACGCCGGCGAGGACTTCGGCCCTCGCAGGCGTA | 33919 | 0.10321675744247626 | No Hit |
| GTCTGTCGGGCTGCGGTCGGAAGCGAGGGTCGACGAAGCGGGCTGGCGGG | 33781 | 0.10279681839571599 | No Hit |
| ATTAGATCCAAAGCCAATGCGGGGGGCAACTCCCGGACCCTTGGTGATTC | 33176 | 0.10095578127042638 | No Hit |
| GAAGCGAGGGTCGACGAAGCGGGCTGGCGGGGGGGCCCTCTCGGGGGTCC | 32878 | 0.10004895637234984 | No Hit |

## Kmer Content

| Sequence | Count | Obs/Exp Overall | Obs/Exp Max | Max Obs/Exp Position |
| --- | --- | --- | --- | --- |
| TCTCT | 16494610 | 4.961427 | 19.01361 | 135-137 |
| TTCTC | 14127575 | 4.249445 | 19.42803 | 130-134 |
| TTTCT | 10584500 | 4.105123 | 21.019855 | 35-39 |
| TTCTT | 10410855 | 4.0377765 | 30.324228 | 40-44 |
| ATCTC | 12736285 | 3.9984095 | 9.938759 | 120-124 |
| GAATC | 12488070 | 3.7264314 | 12.93911 | 60-64 |
| CTCTT | 12261810 | 3.6882398 | 9.332083 | 30-34 |
| ATCAA | 8121635 | 3.5812867 | 12.623524 | 85-89 |
| TCAAA | 7530675 | 3.320699 | 12.37823 | 85-89 |
| CTTCT | 10764065 | 3.2377317 | 15.976443 | 110-114 |
| CTTCG | 15144275 | 3.2173266 | 14.117046 | 70-74 |
| CATTT | 7478710 | 3.0273488 | 9.802983 | 3 |
| AAGAT | 7399835 | 2.9716043 | 11.36104 | 110-114 |
| GCGAA | 13926695 | 2.9351344 | 9.307262 | 35-39 |
| GCCAA | 12506715 | 2.894342 | 9.638605 | 80-84 |
| TATGT | 7812160 | 2.8799186 | 21.036976 | 5 |
| AATCT | 6805950 | 2.875441 | 11.498604 | 45-49 |
| CCAAT | 8713695 | 2.8551357 | 10.7660265 | 100-104 |
| TTGAA | 7413390 | 2.8523705 | 13.54127 | 135-137 |
| TGGCC | 18903335 | 2.8363981 | 10.680333 | 75-79 |
| GTTCT | 10341015 | 2.8327034 | 16.197252 | 130-134 |
| TATCA | 6695055 | 2.8285887 | 12.028856 | 85-89 |
| ATTTC | 6856155 | 2.7753413 | 9.417003 | 4 |
| TCTTC | 9153510 | 2.7532916 | 10.131316 | 40-44 |
| AGGTT | 10550770 | 2.7471056 | 12.831845 | 2 |
| CGAAG | 12901650 | 2.7191 | 15.232656 | 3 |
| CATCA | 8281145 | 2.7134063 | 10.693588 | 50-54 |
| AATCA | 6143225 | 2.7088945 | 13.503859 | 65-69 |
| AATCC | 8151510 | 2.6709301 | 9.6020775 | 100-104 |
| GGCAA | 12663170 | 2.6688387 | 19.522598 | 1 |
| CAACT | 8114360 | 2.6587574 | 29.44635 | 3 |
| CCGGT | 17521090 | 2.6289957 | 12.976574 | 9 |
| CTGGA | 12880050 | 2.6008632 | 11.079334 | 60-64 |
| AACTC | 7925350 | 2.596826 | 30.888456 | 4 |
| GGTTG | 14607495 | 2.5737665 | 10.07396 | 85-89 |
| GACCG | 16401660 | 2.5686002 | 6.2521257 | 55-59 |
| TTCGG | 13211635 | 2.5560927 | 7.1826544 | 70-74 |
| CCATC | 10392385 | 2.5302792 | 16.268227 | 50-54 |
| TCACC | 10289005 | 2.505109 | 11.299362 | 55-59 |
| AAGCG | 11874290 | 2.5025775 | 10.790335 | 100-104 |
| GACTT | 8561435 | 2.4477353 | 6.6947656 | 130-134 |
| TCTTT | 6248055 | 2.4232638 | 12.030597 | 35-39 |
| GGCCA | 15367695 | 2.406675 | 7.231088 | 50-54 |
| GCTGG | 17525355 | 2.3947985 | 9.359891 | 75-79 |
| CGGTA | 11846845 | 2.3922286 | 10.49833 | 95-99 |
| GTAAA | 5938380 | 2.3847177 | 12.060648 | 95-99 |
| TTTTC | 6109435 | 2.3695009 | 19.720581 | 35-39 |
| CAAGA | 7605790 | 2.3687656 | 8.974025 | 110-114 |
| CCTTC | 10139920 | 2.3654177 | 11.411649 | 70-74 |
| CGACC | 13746770 | 2.3639379 | 5.44213 | 50-54 |
| CTTAA | 5593870 | 2.3633502 | 12.547151 | 40-44 |
| ACGCT | 10570965 | 2.3439116 | 8.487945 | 110-114 |
| CAAAC | 6848125 | 2.3419414 | 10.908486 | 90-94 |
| CGTAT | 8162270 | 2.333613 | 11.318934 | 5 |
| AGCGC | 14811230 | 2.319529 | 8.797681 | 100-104 |
| CTTTT | 5974965 | 2.3173478 | 17.84355 | 35-39 |
| ACACA | 6753880 | 2.3097112 | 5.8652143 | 8 |
| CAGGT | 11408895 | 2.3037934 | 13.052062 | 2 |
| ACCCT | 9398300 | 2.2882454 | 13.350552 | 55-59 |
| CCGCG | 19568240 | 2.2771375 | 5.677851 | 85-89 |
| AAAAT | 3830500 | 2.2731194 | 11.5447855 | 125-129 |
| AGATC | 7584430 | 2.2631886 | 9.741779 | 115-119 |
| CGCGA | 14387070 | 2.2531033 | 8.743692 | 40-44 |
| TAAAG | 5604095 | 2.2504764 | 12.140139 | 100-104 |
| AAAGC | 7203160 | 2.2433693 | 14.342635 | 100-104 |
| TCTTA | 5521885 | 2.2352347 | 21.532557 | 40-44 |
| GCTTC | 10483235 | 2.2271116 | 9.908976 | 110-114 |
| CGGTG | 16196125 | 2.2131622 | 8.511799 | 125-129 |
| TTAAC | 5226355 | 2.208079 | 12.702397 | 45-49 |
| TGGAA | 8102935 | 2.2019806 | 14.690625 | 60-64 |
| TGACC | 9898230 | 2.1947455 | 6.6651697 | 75-79 |
| GGAGG | 16838130 | 2.1870024 | 8.280921 | 8 |
| CACAC | 8558395 | 2.174831 | 16.422365 | 8 |
| GCAAC | 9219695 | 2.1336498 | 23.332722 | 2 |
| GGTGC | 15586015 | 2.1297922 | 12.028303 | 125-129 |
| CAATC | 6459865 | 2.1166444 | 13.089311 | 100-104 |
| GAAGC | 10012375 | 2.1101677 | 7.966699 | 2 |
| ACTCC | 8612165 | 2.0968416 | 21.705515 | 5 |
| GAAGA | 7340310 | 2.0819268 | 13.421131 | 25-29 |
| ATGTC | 7267335 | 2.0777488 | 7.9572873 | 30-34 |
| ATCAC | 6329535 | 2.07394 | 10.181206 | 55-59 |
| CGAAT | 6944180 | 2.0721385 | 12.310106 | 45-49 |
| TGGAG | 11249330 | 2.0687115 | 10.21883 | 75-79 |
| TTCGA | 7202010 | 2.0590723 | 18.530437 | 3 |
| GGAAT | 7502425 | 2.0387917 | 18.151564 | 60-64 |
| CGCTT | 9590160 | 2.0373826 | 6.4952865 | 110-114 |
| TGCAT | 7118105 | 2.0350835 | 9.077095 | 85-89 |
| TTGTA | 5455760 | 2.0112424 | 9.003554 | 120-124 |
| TTGCA | 7011350 | 2.0045621 | 15.506863 | 85-89 |
| TGAAA | 4984880 | 2.001814 | 7.011358 | 120-124 |
| TGGTG | 11345605 | 1.999038 | 11.050362 | 115-119 |
| CCCTG | 12108470 | 1.995008 | 5.874511 | 20-24 |
| GAGGA | 10386430 | 1.993515 | 5.60137 | 25-29 |
| CGTTC | 9359260 | 1.9883287 | 11.392017 | 130-134 |
| GGGTT | 11201070 | 1.9735719 | 8.415165 | 85-89 |
| TCTCA | 6267240 | 1.9675274 | 8.5471115 | 70-74 |
| GTTGC | 10148390 | 1.963438 | 11.47267 | 85-89 |
| CGTAG | 9697725 | 1.9582577 | 9.452969 | 60-64 |
| ACTTC | 6208850 | 1.9491967 | 8.049325 | 25-29 |
| ACGAA | 6250930 | 1.9468045 | 17.090097 | 2 |
| CTCTA | 6169370 | 1.9368023 | 18.562176 | 135-137 |
| ATCAG | 6447570 | 1.9239503 | 13.5743265 | 65-69 |
| AGGCG | 13487900 | 1.9236517 | 5.7254643 | 45-49 |
| GCATT | 6727775 | 1.9234875 | 7.73325 | 65-69 |
| GTATC | 6726195 | 1.9230356 | 11.467807 | 85-89 |
| GCGTA | 9475155 | 1.9133141 | 7.477233 | 80-84 |
| CTGGC | 12651985 | 1.8983989 | 7.1825933 | 95-99 |
| GCCAT | 8517865 | 1.8886756 | 11.667778 | 50-54 |
| GTAGG | 10208270 | 1.8772643 | 10.896903 | 80-84 |
| TCTAC | 5956480 | 1.869968 | 27.298697 | 135-137 |
| AGGGA | 9725630 | 1.8666847 | 9.329048 | 6 |
| CTTGA | 6529005 | 1.8666584 | 11.646298 | 135-137 |
| GCCGG | 17578740 | 1.8629391 | 8.939138 | 8 |
| GTGGC | 13610415 | 1.8598311 | 7.9730744 | 115-119 |
| TTCTG | 6770250 | 1.8545675 | 15.160501 | 110-114 |
| GGCTG | 13541885 | 1.8504666 | 5.906298 | 75-79 |
| GCCTT | 8670395 | 1.841983 | 7.984305 | 70-74 |
| GCCCT | 11140580 | 1.8355374 | 5.5279336 | 35-39 |
| TCCGC | 11116090 | 1.8315022 | 7.5146184 | 100-104 |
| AATAT | 3217900 | 1.8296139 | 5.673271 | 70-74 |
| TAGCC | 8210225 | 1.8204622 | 17.415857 | 9 |
| TGAAT | 4724490 | 1.8177912 | 7.562658 | 135-137 |
| AAACG | 5834010 | 1.816958 | 12.236189 | 90-94 |
| CGGAG | 12702065 | 1.8115754 | 22.260595 | 3 |
| CTCCC | 10001615 | 1.8094733 | 20.690884 | 6 |
| TCGAC | 8120940 | 1.800665 | 14.595468 | 4 |
| AAGAG | 6339555 | 1.7980834 | 14.703286 | 25-29 |
| GATCT | 6277875 | 1.7948598 | 11.58642 | 115-119 |
| CACCA | 7061810 | 1.7945238 | 16.182276 | 6 |
| ACGTG | 8793255 | 1.7756183 | 13.076316 | 7 |
| CAGGC | 11337145 | 1.7754662 | 5.444709 | 3 |
| GAAAA | 4233530 | 1.7744 | 6.017378 | 125-129 |
| GTATG | 6787640 | 1.767299 | 10.464265 | 30-34 |
| ATATT | 3243760 | 1.7670777 | 5.5525775 | 70-74 |
| CGCCA | 10231455 | 1.7594333 | 8.945856 | 105-109 |
| TCTCC | 7510295 | 1.7519846 | 7.562071 | 50-54 |
| ACCGC | 10136145 | 1.7430434 | 5.9495244 | 75-79 |
| GCAGG | 12036195 | 1.7166089 | 9.33145 | 1 |
| TGTAG | 6583380 | 1.7141159 | 12.097524 | 7 |
| GGCCC | 14719845 | 1.7129345 | 5.0987973 | 35-39 |
| CGCCG | 14490365 | 1.6862302 | 15.234657 | 9 |
| TCTTG | 6155525 | 1.6861765 | 7.576212 | 120-124 |
| TCAGG | 8340715 | 1.6842371 | 10.459671 | 65-69 |
| TAACG | 5625545 | 1.6786586 | 13.311186 | 45-49 |
| GGTAT | 6413150 | 1.6697929 | 8.246309 | 30-34 |
| GACCC | 9704560 | 1.6688266 | 9.631642 | 15-19 |
| AGGGT | 8977395 | 1.6509109 | 6.008457 | 80-84 |
| CGAGG | 11565040 | 1.6494125 | 5.1534257 | 130-134 |
| CAACG | 7084365 | 1.639485 | 6.1506057 | 125-129 |
| CCAAC | 6397075 | 1.6256034 | 7.0194187 | 85-89 |
| TGTTA | 4404700 | 1.6237736 | 8.309862 | 105-109 |
| AACGC | 7012945 | 1.6229568 | 6.5703926 | 90-94 |
| TCCCG | 9850060 | 1.6229092 | 14.8717375 | 7 |
| CCCGC | 12692240 | 1.621819 | 6.937741 | 9 |
| CACGC | 9423755 | 1.6205386 | 9.36347 | 105-109 |
| ATCCG | 7255035 | 1.6086669 | 6.160918 | 100-104 |
| GACGT | 7939885 | 1.6032977 | 12.979706 | 6 |
| AGTTC | 5598030 | 1.6004905 | 9.006609 | 7 |
| CCCTC | 8823555 | 1.5963409 | 5.806573 | 35-39 |
| CTGTT | 5808275 | 1.5910547 | 5.3892064 | 105-109 |
| AAGTT | 4132005 | 1.589827 | 6.8046246 | 5 |
| GGTAA | 5850215 | 1.5898018 | 14.713228 | 95-99 |
| AGGTA | 5842300 | 1.5876508 | 8.167986 | 80-84 |
| CATCT | 5036495 | 1.5811495 | 6.098789 | 110-114 |
| CCACG | 9134955 | 1.5708754 | 10.782111 | 8 |
| GGTGA | 8541160 | 1.570689 | 6.7157273 | 75-79 |
| GAGGT | 8528430 | 1.5683477 | 5.5870275 | 75-79 |
| AACGT | 5253300 | 1.567581 | 5.9806485 | 85-89 |
| AGAGT | 5672885 | 1.5416121 | 14.540478 | 25-29 |
| GGAAG | 7963985 | 1.5285642 | 11.973016 | 1 |
| AAATC | 3425805 | 1.5106305 | 9.023427 | 125-129 |
| CCTGG | 10030185 | 1.5050042 | 7.133405 | 20-24 |
| CCGCC | 11555910 | 1.4766184 | 5.502588 | 55-59 |
| GAGGG | 11306015 | 1.4684696 | 7.7612348 | 10-14 |
| TTAAT | 2688845 | 1.4647812 | 6.728614 | 70-74 |
| TAATA | 2568355 | 1.4602994 | 6.841804 | 70-74 |
| GGAGA | 7598440 | 1.4584033 | 25.144964 | 4 |
| CTCAC | 5982260 | 1.4565269 | 5.0514297 | 70-74 |
| CTTCC | 6226360 | 1.4524711 | 6.4328313 | 60-64 |
| AATGG | 5321910 | 1.4462345 | 6.8005114 | 135-137 |
| TGCCC | 8748290 | 1.4413803 | 8.888151 | 90-94 |
| CCGTA | 6485545 | 1.4380469 | 6.8563886 | 60-64 |
| GCAAG | 6804800 | 1.4341524 | 6.069372 | 110-114 |
| CCGGA | 9121475 | 1.4284787 | 12.311545 | 9 |
| GTAGC | 7064770 | 1.4265862 | 9.132894 | 8 |
| CATGC | 6431375 | 1.4260359 | 10.145186 | 90-94 |
| CTTGT | 5198450 | 1.424006 | 9.225079 | 120-124 |
| GGCCT | 9456490 | 1.4189228 | 5.5948997 | 70-74 |
| AAGGG | 7387980 | 1.4180088 | 9.564609 | 5 |
| TCGGA | 7003050 | 1.414123 | 30.008387 | 2 |
| GCATG | 6999065 | 1.4133185 | 6.698955 | 90-94 |
| AGGCA | 6690295 | 1.4100198 | 6.120837 | 15-19 |
| GGCGT | 10281970 | 1.405007 | 5.0134764 | 1 |
| GTGCC | 9330190 | 1.3999717 | 9.9693165 | 9 |
| TCTGG | 7212125 | 1.3953505 | 6.753781 | 110-114 |
| CGCAT | 6291270 | 1.3949702 | 6.0182776 | 65-69 |
| ACGCC | 8085225 | 1.3903606 | 22.125715 | 8 |
| TTCAG | 4857725 | 1.3888354 | 6.4481473 | 2 |
| GCGTC | 9248055 | 1.3876476 | 7.389406 | 120-124 |
| TTGGC | 7164705 | 1.3861759 | 9.924282 | 70-74 |
| ATGCC | 6246835 | 1.3851177 | 11.379754 | 90-94 |
| GGTGG | 11093595 | 1.3805362 | 8.796652 | 115-119 |
| ACCAC | 5407095 | 1.3740331 | 16.079624 | 7 |
| TCCGG | 9153690 | 1.3734883 | 6.9809685 | 120-124 |
| GAAGG | 7139530 | 1.3703226 | 9.576329 | 4 |
| GCGTT | 7066470 | 1.3671701 | 6.8721538 | 130-134 |
| CTCCG | 8290975 | 1.3660322 | 6.5712066 | 50-54 |
| GACGA | 6429355 | 1.3550249 | 5.0407734 | 130-134 |
| AGACG | 6411865 | 1.3513389 | 26.93161 | 6 |
| ACACC | 5316385 | 1.3509822 | 16.241617 | 9 |
| TGGCT | 6959805 | 1.3465332 | 10.692061 | 70-74 |
| GTGAC | 6666835 | 1.3462312 | 8.080664 | 75-79 |
| TACGG | 6656610 | 1.3441665 | 11.288293 | 135-137 |
| GTTCA | 4691080 | 1.3411913 | 7.3633304 | 1 |
| GAGTT | 5137895 | 1.3377545 | 8.107588 | 6 |
| GTGCA | 6601445 | 1.3330271 | 13.563169 | 1 |
| ATTTT | 2522845 | 1.3167932 | 5.3808794 | 135-137 |
| AACGG | 6245240 | 1.3162216 | 10.64183 | 45-49 |
| TAGAC | 4390775 | 1.3102041 | 7.879479 | 125-129 |
| AGGAC | 6149045 | 1.295948 | 5.807249 | 25-29 |
| TGCTG | 6685750 | 1.2935112 | 5.0175247 | 105-109 |
| TGGGA | 7028885 | 1.2925869 | 10.071838 | 20-24 |
| GGGAA | 6698720 | 1.2857162 | 11.723286 | 1 |
| CGACT | 5798455 | 1.2856978 | 5.272097 | 130-134 |
| GTCGC | 8564360 | 1.2850609 | 7.336105 | 8 |
| GCACA | 5537760 | 1.2815653 | 14.874721 | 7 |
| CCCGG | 11005215 | 1.2806665 | 10.093943 | 8 |
| GTAGA | 4711335 | 1.2803099 | 6.180443 | 120-124 |
| GGTAG | 6927485 | 1.2739397 | 8.680868 | 80-84 |
| TCCGT | 5973020 | 1.2689387 | 5.8761744 | 50-54 |
| CTGGT | 6555640 | 1.2683384 | 6.27146 | 115-119 |
| TATAG | 3296390 | 1.2683164 | 15.35791 | 7 |
| CGTCC | 7695110 | 1.2678568 | 9.195837 | 120-124 |
| TCTCG | 5921505 | 1.2579945 | 5.0070314 | 110-114 |
| GCGAC | 7918205 | 1.2400395 | 5.262511 | 115-119 |
| CACCC | 6508240 | 1.2289256 | 9.6609125 | 55-59 |
| TGTCG | 6346460 | 1.2278677 | 5.224568 | 30-34 |
| TAGGG | 6647235 | 1.2224027 | 9.872204 | 80-84 |
| TCGCA | 5463920 | 1.2115209 | 5.404921 | 35-39 |
| ATGTA | 3118965 | 1.2000506 | 15.612479 | 6 |
| TAGGC | 5932950 | 1.1980381 | 5.390578 | 60-64 |
| GTTTC | 4329470 | 1.1859672 | 18.803967 | 1 |
| ATATG | 3076165 | 1.1835828 | 15.145685 | 4 |
| ACTTG | 4115240 | 1.1765572 | 8.057367 | 130-134 |
| TCGGT | 6070425 | 1.1744624 | 5.284079 | 70-74 |
| GTATA | 3041300 | 1.1701683 | 15.217462 | 6 |
| GTTGG | 6638940 | 1.1697476 | 8.04929 | 70-74 |
| TGCAC | 5258315 | 1.1659319 | 14.00434 | 6 |
| GGGAC | 8166800 | 1.1647534 | 6.848263 | 1 |
| GAGGC | 8164295 | 1.164396 | 6.978249 | 9 |
| GTTAC | 4058035 | 1.1602021 | 6.4162946 | 110-114 |
| GACTA | 3849940 | 1.1488193 | 8.279406 | 115-119 |
| TTACG | 4013260 | 1.1474009 | 6.842434 | 110-114 |
| GAAGT | 4209995 | 1.1440703 | 5.8995833 | 4 |
| GCCGA | 7279970 | 1.1400878 | 5.0530148 | 50-54 |
| TAAAT | 1993615 | 1.1335174 | 5.8987517 | 3 |
| ACGGC | 7194770 | 1.126745 | 6.19938 | 135-137 |
| TTGCG | 5748990 | 1.1122735 | 5.075374 | 95-99 |
| ACATT | 2622720 | 1.1080711 | 9.180828 | 2 |
| ACGAC | 4787840 | 1.1080163 | 5.052336 | 125-129 |
| CTCGG | 7275045 | 1.0916023 | 20.295067 | 1 |
| GCGCC | 9290275 | 1.0811006 | 5.2579713 | 105-109 |
| CACGA | 4649020 | 1.0758903 | 14.238655 | 9 |
| TTTCG | 3884555 | 1.0640922 | 17.770933 | 2 |
| CTACG | 4769110 | 1.0574601 | 10.811791 | 135-137 |
| GCCAC | 6118240 | 1.0521119 | 8.0131035 | 105-109 |
| CCTGA | 4743210 | 1.0517172 | 5.4891667 | 130-134 |
| AATTG | 2685320 | 1.0332016 | 6.591367 | 5 |
| CACAT | 3109165 | 1.0187514 | 7.618251 | 1 |
| AGCTC | 4553040 | 1.0095506 | 6.528732 | 4 |
| TGCGT | 5209415 | 1.0078804 | 6.3152957 | 125-129 |
| AGTTA | 2618830 | 1.007619 | 6.150136 | 6 |
| ATAGC | 3335650 | 0.9953556 | 12.4748535 | 8 |
| GTCCG | 6607125 | 0.9913826 | 8.542196 | 120-124 |
| AGGTG | 5330970 | 0.9803463 | 11.729249 | 3 |
| AGGCC | 6242530 | 0.97761846 | 5.5293508 | 135-137 |
| GTGCG | 7079115 | 0.9673444 | 8.701161 | 125-129 |
| GGTTT | 3851815 | 0.9608967 | 11.934716 | 3 |
| GAGCG | 6728925 | 0.95968294 | 5.037913 | 135-137 |
| TCCCT | 4113235 | 0.9595262 | 6.7364445 | 7 |
| AAGCT | 3208545 | 0.95742756 | 8.732116 | 3 |
| TTAAA | 1667240 | 0.9479491 | 5.3604207 | 2 |
| CGTGC | 6200180 | 0.9303216 | 9.804194 | 8 |
| GGACC | 5925075 | 0.92790306 | 8.054475 | 15-19 |
| GGGAG | 7106150 | 0.9229747 | 6.114167 | 7 |
| ACGTA | 3066650 | 0.9150862 | 11.636081 | 4 |
| CATAT | 2155655 | 0.91074103 | 16.640247 | 3 |
| CTTTG | 3299735 | 0.903893 | 5.2981935 | 8 |
| AAGCA | 2866245 | 0.89267015 | 6.7180047 | 4 |
| ACTTT | 2204570 | 0.8924002 | 7.3735604 | 7 |
| TCGCG | 5922755 | 0.8886946 | 5.848887 | 35-39 |
| GCCAG | 5661835 | 0.88667804 | 5.752251 | 1 |
| GAGAC | 4185755 | 0.88217294 | 27.12946 | 5 |
| CTGCA | 3878085 | 0.859892 | 5.164215 | 3 |
| TACTC | 2734030 | 0.85831714 | 6.009008 | 120-124 |
| TTTGC | 3113370 | 0.8528422 | 5.519687 | 9 |
| GCACG | 5402375 | 0.846045 | 5.381238 | 15-19 |
| CGACG | 5304620 | 0.830736 | 10.229166 | 5 |
| TCGCC | 5025735 | 0.828047 | 5.4908743 | 9 |
| CGTCG | 5395515 | 0.80958354 | 5.070291 | 7 |
| ATTGT | 2193965 | 0.80879563 | 5.0893636 | 6 |
| TTCCC | 3361270 | 0.78410953 | 5.454274 | 6 |
| GGACG | 5414330 | 0.7721947 | 5.7327933 | 2 |
| TTTCC | 2512635 | 0.75577754 | 6.97245 | 5 |
| ACGGG | 5286130 | 0.75391066 | 7.5524974 | 45-49 |
| GAATT | 1919255 | 0.738451 | 5.6410017 | 4 |
| CTGGG | 5223665 | 0.7138015 | 6.9865007 | 20-24 |
| AGCAT | 2320450 | 0.69242066 | 11.708085 | 1 |
| GTAGT | 2530245 | 0.6588003 | 5.5578275 | 125-129 |
| CCTGT | 3066695 | 0.6515042 | 8.305537 | 9 |
| CCAGG | 3970735 | 0.6218413 | 5.212364 | 2 |
| GACGC | 3786555 | 0.59299767 | 20.098618 | 7 |
| CCCCG | 4036585 | 0.5157963 | 5.8587484 | 8 |
| GCATA | 1650330 | 0.49245736 | 11.522151 | 2 |

Produced by FastQC (version 0.10.1)
