# Supplementary material for: Freshwater sponge hosts and their green algae symbionts: a tractable model to understand intracellular symbiosis
Source: PeerJ. 2021 Feb 11;9:e10654. doi: 10.7717/peerj.10654 (PMC7882143; doi:10.7717/peerj.10654)
Supplement: Supplemental Information 30 [file peerj-09-10654-s030.zip › EmInf1_Clean_Data1.fq_fastqc/fastqc_report.html]

EmInf1\_Clean\_Data1.fq.gz FastQC Report


FastQC Report

Tue 10 Sep 2019  
EmInf1\_Clean\_Data1.fq.gz

## Summary

- Basic Statistics
- Per base sequence quality
- Per sequence quality scores
- Per base sequence content
- Per base GC content
- Per sequence GC content
- Per base N content
- Sequence Length Distribution
- Sequence Duplication Levels
- Overrepresented sequences
- Kmer Content

## Basic Statistics

| Measure | Value |
| --- | --- |
| Filename | EmInf1\_Clean\_Data1.fq.gz |
| File type | Conventional base calls |
| Encoding | Sanger / Illumina 1.9 |
| Total Sequences | 33511120 |
| Filtered Sequences | 0 |
| Sequence length | 100-141 |
| %GC | 57 |

## Per base sequence quality

## Per sequence quality scores

## Per base sequence content

## Per base GC content

## Per sequence GC content

## Per base N content

## Sequence Length Distribution

## Sequence Duplication Levels

## Overrepresented sequences

| Sequence | Count | Percentage | Possible Source |
| --- | --- | --- | --- |
| GTCCCATTCAAGTCGTCTACAAGAGATCTTGCCCCGCGGATTGGCCAGCG | 1550722 | 4.6274848468209955 | No Hit |
| GCGAGAAAATGAACCGCTCCCTCGGATTTTCAAGGGCCGTAGAGAACGCA | 1104217 | 3.295076380616345 | No Hit |
| AGAAAATGAACCGCTCCCTCGGATTTTCAAGGGCCGTAGAGAACGCACCG | 638733 | 1.9060329824846198 | No Hit |
| GAGAAAATGAACCGCTCCCTCGGATTTTCAAGGGCCGTAGAGAACGCACC | 632684 | 1.8879822578296397 | No Hit |
| GCCACCTACAGCCAACAGTCTGAAGCGCAGTCGCGAACCCCGCGCACGGC | 553375 | 1.6513175328070204 | No Hit |
| GTCGTCTACAAGAGATCTTGCCCCGCGGATTGGCCAGCGTTTGATACGCG | 503512 | 1.5025221478721091 | No Hit |
| GGCGAGAAAATGAACCGCTCCCTCGGATTTTCAAGGGCCGTAGAGAACGC | 468198 | 1.3971422023495483 | No Hit |
| AAGAGATCTTGCCCCGCGGATTGGCCAGCGTTTGATACGCGCGGTCACCG | 422287 | 1.2601399177347699 | No Hit |
| GCCGTTAGTCGCCTGCCGAATAGCCGCCGACCACGAGGGACGGCGACCAA | 415141 | 1.2388156528340444 | No Hit |
| CGGGCGAGAAAATGAACCGCTCCCTCGGATTTTCAAGGGCCGTAGAGAAC | 345903 | 1.0322036386727749 | No Hit |
| CTGCGCTGGCGGGTCGAAGAGACCCTCTCCTCGGTCGCGGGCGCGCTCCG | 328325 | 0.9797494085545335 | No Hit |
| ATTCAAGTCGTCTACAAGAGATCTTGCCCCGCGGATTGGCCAGCGTTTGA | 306800 | 0.9155169985366052 | No Hit |
| AGCGCAGTCGCGAACCCCGCGCACGGCGGAGGGATGCGCCGGCCTCGCAC | 252122 | 0.7523532487126662 | No Hit |
| GGCCGTTAGTCGCCTGCCGAATAGCCGCCGACCACGAGGGACGGCGACCA | 249776 | 0.745352587439632 | No Hit |
| GTCGCCGTAACAGCACCGCCCGCAACCCACGTTGGCCAGCCCCGGTGAGA | 249203 | 0.7436427072565763 | No Hit |
| CTCTCCTCGGTCGCGGGCGCGCTCCGAACGACGCGGCTATACGTCCCTAA | 227325 | 0.6783569155551947 | No Hit |
| GAAAATGAACCGCTCCCTCGGATTTTCAAGGGCCGTAGAGAACGCACCGG | 223070 | 0.6656596377560642 | No Hit |
| GATGAAGCCACCTACAGCCAACAGTCTGAAGCGCAGTCGCGAACCCCGCG | 216502 | 0.6460601734588399 | No Hit |
| GTCAGATGAAGCCACCTACAGCCAACAGTCTGAAGCGCAGTCGCGAACCC | 188899 | 0.5636905003473474 | No Hit |
| GTCTACAAGAGATCTTGCCCCGCGGATTGGCCAGCGTTTGATACGCGCGG | 184540 | 0.5506828778029502 | No Hit |
| GGGCGAGAAAATGAACCGCTCCCTCGGATTTTCAAGGGCCGTAGAGAACG | 179252 | 0.5349030411397768 | No Hit |
| GCAGAAATTTGAATGCACCATCGCCGGCACGAGGCCATGCGATTCGAGCA | 177680 | 0.5302120609517079 | No Hit |
| GCGCTGGCGGGTCGAAGAGACCCTCTCCTCGGTCGCGGGCGCGCTCCGAA | 174117 | 0.5195797693422363 | No Hit |
| CTCCTCGGTCGCGGGCGCGCTCCGAACGACGCGGCTATACGTCCCTAACT | 171259 | 0.5110512570155817 | No Hit |
| GAGATCTTGCCCCGCGGATTGGCCAGCGTTTGATACGCGCGGTCACCGAA | 168511 | 0.5028509939387283 | No Hit |
| AGAGATCTTGCCCCGCGGATTGGCCAGCGTTTGATACGCGCGGTCACCGA | 167020 | 0.49840172456187676 | No Hit |
| AGAAATTTGAATGCACCATCGCCGGCACGAGGCCATGCGATTCGAGCAGT | 146855 | 0.4382276689051276 | No Hit |
| GTCGGCCGTTAGTCGCCTGCCGAATAGCCGCCGACCACGAGGGACGGCGA | 138081 | 0.41204531510734344 | No Hit |
| CTCGTCCCATTCAAGTCGTCTACAAGAGATCTTGCCCCGCGGATTGGCCA | 135256 | 0.4036152775556293 | No Hit |
| CGAGAAAATGAACCGCTCCCTCGGATTTTCAAGGGCCGTAGAGAACGCAC | 121779 | 0.36339877628679673 | No Hit |
| GCCCGCAACCCACGTTGGCCAGCCCCGGTGAGAAATGCGGAAGCGGCGGT | 118012 | 0.3521577315231481 | No Hit |
| CAAGAGATCTTGCCCCGCGGATTGGCCAGCGTTTGATACGCGCGGTCACC | 109407 | 0.3264796879364223 | No Hit |
| CCTGACTCTCCAAAGACACCTAATATCTAGGCAGGCGGTCGGCCGCGTAC | 105962 | 0.31619951824946463 | No Hit |
| CAAGTCGTCTACAAGAGATCTTGCCCCGCGGATTGGCCAGCGTTTGATAC | 103031 | 0.3074531677843056 | No Hit |
| CTTATATTGGTCGGGCTAGGAGCTGAGTCTACTCACAGGCACTATCCCAT | 97144 | 0.28988586475176004 | No Hit |
| GAAGCCACCTACAGCCAACAGTCTGAAGCGCAGTCGCGAACCCCGCGCAC | 92489 | 0.27599495331698853 | No Hit |
| CCGCCCGCAACCCACGTTGGCCAGCCCCGGTGAGAAATGCGGAAGCGGCG | 90038 | 0.2686809632145986 | No Hit |
| CATTCAAGTCGTCTACAAGAGATCTTGCCCCGCGGATTGGCCAGCGTTTG | 89252 | 0.26633547312056416 | No Hit |
| GGCAGAAATTTGAATGCACCATCGCCGGCACGAGGCCATGCGATTCGAGC | 89132 | 0.2659773830298719 | No Hit |
| GCCTGCGCTGGCGGGTCGAAGAGACCCTCTCCTCGGTCGCGGGCGCGCTC | 89123 | 0.26595052627307 | No Hit |
| GAAATTTGAATGCACCATCGCCGGCACGAGGCCATGCGATTCGAGCAGTT | 87800 | 0.2620025830231875 | No Hit |
| GCGGGAGCTCCGGCCACGAAGGCCTGCGCTGGCGGGTCGAAGAGACCCTC | 85786 | 0.25599263766773533 | No Hit |
| GCCGTAACAGCACCGCCCGCAACCCACGTTGGCCAGCCCCGGTGAGAAAT | 82671 | 0.24669721573018152 | No Hit |
| CCCGCAACCCACGTTGGCCAGCCCCGGTGAGAAATGCGGAAGCGGCGGTC | 82418 | 0.2459422424556386 | No Hit |
| CCGCAACCCACGTTGGCCAGCCCCGGTGAGAAATGCGGAAGCGGCGGTCG | 79902 | 0.23843428688745705 | No Hit |
| TTCAAGTCGTCTACAAGAGATCTTGCCCCGCGGATTGGCCAGCGTTTGAT | 78827 | 0.235226396491672 | No Hit |
| GCGCAGTCGCGAACCCCGCGCACGGCGGAGGGATGCGCCGGCCTCGCACT | 77062 | 0.22995948807440636 | No Hit |
| CGCGGATTGGCCAGCGTTTGATACGCGCGGTCACCGAAGGCCGCCTACGG | 76382 | 0.22793031089381674 | No Hit |
| AGATGAAGCCACCTACAGCCAACAGTCTGAAGCGCAGTCGCGAACCCCGC | 76245 | 0.2275214913736097 | No Hit |
| CCCATTCAAGTCGTCTACAAGAGATCTTGCCCCGCGGATTGGCCAGCGTT | 76165 | 0.2272827646464815 | No Hit |
| AAAATGAACCGCTCCCTCGGATTTTCAAGGGCCGTAGAGAACGCACCGGA | 75985 | 0.2267456295104431 | No Hit |
| AGATCTTGCCCCGCGGATTGGCCAGCGTTTGATACGCGCGGTCACCGAAG | 73890 | 0.22049397334377366 | No Hit |
| CCTGCGCTGGCGGGTCGAAGAGACCCTCTCCTCGGTCGCGGGCGCGCTCC | 73314 | 0.2187751409084507 | No Hit |
| GCCAACAGTCTGAAGCGCAGTCGCGAACCCCGCGCACGGCGGAGGGATGC | 73025 | 0.2179127406067001 | No Hit |
| GTTAGTCGCCTGCCGAATAGCCGCCGACCACGAGGGACGGCGACCAAGCT | 68989 | 0.20586897722308295 | No Hit |
| GCCACGAAGGCCTGCGCTGGCGGGTCGAAGAGACCCTCTCCTCGGTCGCG | 66717 | 0.19908913817264237 | No Hit |
| GGCGGGTCGAAGAGACCCTCTCCTCGGTCGCGGGCGCGCTCCGAACGACG | 66585 | 0.1986952390728809 | No Hit |
| CTACAAGAGATCTTGCCCCGCGGATTGGCCAGCGTTTGATACGCGCGGTC | 64929 | 0.19375359582132737 | No Hit |
| CACCTACAGCCAACAGTCTGAAGCGCAGTCGCGAACCCCGCGCACGGCGG | 62551 | 0.18665744385744193 | No Hit |
| GGCCTGCGCTGGCGGGTCGAAGAGACCCTCTCCTCGGTCGCGGGCGCGCT | 62379 | 0.18614418139411634 | No Hit |
| CGAGATGGCGCCCTCCACCGGAACGCGGGAGCTCCGGCCACGAAGGCCTG | 62148 | 0.1854548579695337 | No Hit |
| CGTTAGTCGCCTGCCGAATAGCCGCCGACCACGAGGGACGGCGACCAAGC | 61754 | 0.18427912883842737 | No Hit |
| CTGCTTACAACACCTCGTCCCATTCAAGTCGTCTACAAGAGATCTTGCCC | 61467 | 0.183422696704855 | No Hit |
| CTCCACCGGAACGCGGGAGCTCCGGCCACGAAGGCCTGCGCTGGCGGGTC | 59634 | 0.17795287056953035 | No Hit |
| TGACTCTCCAAAGACACCTAATATCTAGGCAGGCGGTCGGCCGCGTACGG | 59222 | 0.17672342792482018 | No Hit |
| GTCTACTTATATTGGTCGGGCTAGGAGCTGAGTCTACTCACAGGCACTAT | 58951 | 0.17591474113667344 | No Hit |
| CCGGGCGAGAAAATGAACCGCTCCCTCGGATTTTCAAGGGCCGTAGAGAA | 56909 | 0.16982124142672642 | No Hit |
| GCTCCCTCGGATTTTCAAGGGCCGTAGAGAACGCACCGGACGCCACCAGA | 55042 | 0.16424995643237228 | No Hit |
| GCTTACAACACCTCGTCCCATTCAAGTCGTCTACAAGAGATCTTGCCCCG | 54918 | 0.1638799300053236 | No Hit |
| AAGCGCAGTCGCGAACCCCGCGCACGGCGGAGGGATGCGCCGGCCTCGCA | 54019 | 0.1611972384092206 | No Hit |
| CAGATGAAGCCACCTACAGCCAACAGTCTGAAGCGCAGTCGCGAACCCCG | 52951 | 0.15801023660205926 | No Hit |
| GATCTTGCCCCGCGGATTGGCCAGCGTTTGATACGCGCGGTCACCGAAGG | 52905 | 0.15787296873396053 | No Hit |
| CTCCAAAGACACCTAATATCTAGGCAGGCGGTCGGCCGCGTACGGGGTTC | 49481 | 0.14765546481287406 | No Hit |
| CTACTGCTTACAACACCTCGTCCCATTCAAGTCGTCTACAAGAGATCTTG | 48708 | 0.14534876781199793 | No Hit |
| CACCGGAACGCGGGAGCTCCGGCCACGAAGGCCTGCGCTGGCGGGTCGAA | 48317 | 0.1441819909331589 | No Hit |
| GCCCACTGGTGTTAGTTTTAGTACAGCCGAGCCCAATTTATTGGGCTGAA | 47386 | 0.1414038086462046 | No Hit |
| CACCCGGTCGCCGTAACAGCACCGCCCGCAACCCACGTTGGCCAGCCCCG | 46855 | 0.13981925999489125 | No Hit |
| AAATGAACCGCTCCCTCGGATTTTCAAGGGCCGTAGAGAACGCACCGGAC | 46185 | 0.13781992365519266 | No Hit |
| GGGAAATGTGTCGTTGCGTTCTAGCGTGGATTCTGACTTAGAGGCGTTCA | 45617 | 0.13612496389258252 | No Hit |
| CCGAGATGGCGCCCTCCACCGGAACGCGGGAGCTCCGGCCACGAAGGCCT | 45511 | 0.13580865097913766 | No Hit |
| ACCGCCCGCAACCCACGTTGGCCAGCCCCGGTGAGAAATGCGGAAGCGGC | 45370 | 0.13538789512257424 | No Hit |
| CTCCAGCCAACCTGATTCCAGGGTGATGGCCCGTTAAGAAGAAAAGAGAA | 43904 | 0.13101322784795016 | No Hit |
| ACAAGAGATCTTGCCCCGCGGATTGGCCAGCGTTTGATACGCGCGGTCAC | 43875 | 0.13092668940936622 | No Hit |
| GTCCGAGATGGCGCCCTCCACCGGAACGCGGGAGCTCCGGCCACGAAGGC | 43552 | 0.12996283024858613 | No Hit |
| CTCGGATTTTCAAGGGCCGTAGAGAACGCACCGGACGCCACCAGAAGCGT | 43471 | 0.12972111943736886 | No Hit |
| CTCGGTCGCGGGCGCGCTCCGAACGACGCGGCTATACGTCCCTAACTTCG | 43465 | 0.12970321493283424 | No Hit |
| CTCTACTGCTTACAACACCTCGTCCCATTCAAGTCGTCTACAAGAGATCT | 42699 | 0.12741740652058184 | No Hit |
| CTCGTCCCGGTTCGGGAATATTAACCCGATTCCCTTTCGATGGTGGGTGC | 42457 | 0.12669525817101904 | No Hit |
| CATTATTCAACCTGGATACAGCCGGACTCCTCCGGCGAACCCATATTGAT | 40728 | 0.12153577678096106 | No Hit |
| CGCCGTAACAGCACCGCCCGCAACCCACGTTGGCCAGCCCCGGTGAGAAA | 40218 | 0.12001389389551886 | No Hit |
| GTCGGGCTAGGAGCTGAGTCTACTCACAGGCACTATCCCATTACCGCCTG | 40197 | 0.1199512281296477 | No Hit |
| CCCGGTCGCCGTAACAGCACCGCCCGCAACCCACGTTGGCCAGCCCCGGT | 40013 | 0.11940215665725287 | No Hit |
| CTACAGCCAACAGTCTGAAGCGCAGTCGCGAACCCCGCGCACGGCGGAGG | 39897 | 0.11905600290291699 | No Hit |
| GCCAGCGTTTGATACGCGCGGTCACCGAAGGCCGCCTACGGGCCACGGAG | 39891 | 0.11903809839838239 | No Hit |
| CCATTCAAGTCGTCTACAAGAGATCTTGCCCCGCGGATTGGCCAGCGTTT | 39362 | 0.11745951791524724 | No Hit |
| CAACAGTCTGAAGCGCAGTCGCGAACCCCGCGCACGGCGGAGGGATGCGC | 37979 | 0.11333252962001866 | No Hit |
| CAGAAATTTGAATGCACCATCGCCGGCACGAGGCCATGCGATTCGAGCAG | 37859 | 0.11297443952932638 | No Hit |
| TACAAGAGATCTTGCCCCGCGGATTGGCCAGCGTTTGATACGCGCGGTCA | 37457 | 0.11177483772550723 | No Hit |
| GACCGGGCGAGAAAATGAACCGCTCCCTCGGATTTTCAAGGGCCGTAGAG | 37227 | 0.1110884983850137 | No Hit |
| GCGGGGAAATGTGTCGTTGCGTTCTAGCGTGGATTCTGACTTAGAGGCGT | 36987 | 0.11037231820362912 | No Hit |
| CCGGTCGCCGTAACAGCACCGCCCGCAACCCACGTTGGCCAGCCCCGGTG | 36122 | 0.10779108546655558 | No Hit |
| CCTCGGTCGCGGGCGCGCTCCGAACGACGCGGCTATACGTCCCTAACTTC | 36058 | 0.10760010408485303 | No Hit |
| GTTCTAAGTCGGCCGTTAGTCGCCTGCCGAATAGCCGCCGACCACGAGGG | 35735 | 0.10663624492407296 | No Hit |
| GTCCCGGTTCGGGAATATTAACCCGATTCCCTTTCGATGGTGGGTGCCGG | 35617 | 0.1062841230015589 | No Hit |
| CGCCCGCAACCCACGTTGGCCAGCCCCGGTGAGAAATGCGGAAGCGGCGG | 34977 | 0.10437430918453339 | No Hit |
| GTCCCCTCCGGCGTCTCCGAGTTCGCTCGCGTTGCCGCCTCCGGCCCCTT | 34387 | 0.10261369957196298 | No Hit |
| GCGCATATGTAGCCCAAAACATTAGGATCATAAGGACCTGACGTCATCCT | 33956 | 0.10132755932955985 | No Hit |
| GCACCGCCCGCAACCCACGTTGGCCAGCCCCGGTGAGAAATGCGGAAGCG | 33814 | 0.10090381938890733 | No Hit |

## Kmer Content

| Sequence | Count | Obs/Exp Overall | Obs/Exp Max | Max Obs/Exp Position |
| --- | --- | --- | --- | --- |
| GAGAT | 14466160 | 4.2839537 | 20.017403 | 3 |
| TTCAA | 11079935 | 4.182629 | 89.71402 | 7 |
| ATTTT | 6345180 | 4.097203 | 40.641254 | 25-29 |
| AGAGA | 15403845 | 4.069379 | 19.26431 | 40-44 |
| GATTC | 12983685 | 3.939335 | 20.531868 | 110-114 |
| AGAAA | 11951430 | 3.9283133 | 54.71821 | 4 |
| TTGAT | 8217895 | 3.8047342 | 24.362175 | 50-54 |
| GGATT | 11381515 | 3.7781923 | 28.57436 | 20-24 |
| TTTGA | 7941580 | 3.6768055 | 24.802902 | 50-54 |
| AAGAA | 10505105 | 3.452921 | 46.839615 | 130-134 |
| TTCGC | 15276150 | 3.4048066 | 15.782144 | 95-99 |
| AAATG | 9128550 | 3.3634155 | 58.461185 | 7 |
| GAGAA | 12683305 | 3.3506682 | 41.014122 | 3 |
| ATCTT | 7875330 | 3.3325248 | 28.188234 | 6 |
| TTTCA | 7707905 | 3.2616777 | 35.829468 | 25-29 |
| TTTTC | 6841840 | 3.2454135 | 33.792984 | 25-29 |
| CTTCG | 14083900 | 3.1390734 | 12.522247 | 100-104 |
| AGAAG | 11285715 | 2.9814537 | 22.682123 | 130-134 |
| ATTGG | 8931875 | 2.965013 | 17.991896 | 40-44 |
| TTGCC | 13267235 | 2.957052 | 13.47873 | 9 |
| TGATA | 7067940 | 2.919202 | 21.734861 | 50-54 |
| TGATT | 6284220 | 2.9094784 | 28.442513 | 110-114 |
| ACCGG | 20385785 | 2.9062548 | 14.707131 | 75-79 |
| GATTT | 6164430 | 2.8540177 | 28.206423 | 25-29 |
| ATGAA | 7700920 | 2.8374052 | 56.814613 | 9 |
| AAAAT | 6149385 | 2.819017 | 70.063446 | 6 |
| TCCCT | 13809630 | 2.8132057 | 17.356676 | 15-19 |
| AGATT | 6786190 | 2.8028333 | 21.463097 | 90-94 |
| TGATG | 8339175 | 2.7682612 | 22.114126 | 120-124 |
| GATGG | 11607695 | 2.762805 | 18.466976 | 120-124 |
| CGAAG | 14218865 | 2.7594185 | 11.65553 | 65-69 |
| AGTTG | 8297670 | 2.7544832 | 16.810503 | 115-119 |
| CGCTT | 12314630 | 2.7447317 | 18.707405 | 70-74 |
| TTGGC | 11163205 | 2.722241 | 13.925651 | 40-44 |
| TCGCG | 16978505 | 2.7133026 | 11.189007 | 95-99 |
| TCCAG | 13624560 | 2.708997 | 16.198282 | 95-99 |
| GGCCA | 18977770 | 2.7055244 | 8.306158 | 80-84 |
| GCGCT | 16766415 | 2.679409 | 11.99769 | 70-74 |
| GAGTT | 8068430 | 2.678385 | 16.35996 | 115-119 |
| TACCG | 13405755 | 2.6654916 | 18.84144 | 75-79 |
| TTTAC | 6286890 | 2.6603606 | 26.800274 | 75-79 |
| AAGAG | 10067810 | 2.6597083 | 15.622899 | 1 |
| GAAAA | 8072345 | 2.6532974 | 50.34684 | 5 |
| TCTTG | 7761845 | 2.6398687 | 21.774874 | 7 |
| GTTTG | 7072225 | 2.6316767 | 19.221703 | 50-54 |
| TCAAG | 9535795 | 2.5810077 | 63.599464 | 8 |
| CTTTA | 6085610 | 2.575187 | 25.534882 | 75-79 |
| GCTTT | 7553785 | 2.569106 | 26.356926 | 70-74 |
| ATACG | 9446775 | 2.556913 | 14.701422 | 55-59 |
| ACATA | 7496630 | 2.5245602 | 17.937422 | 105-109 |
| TGTGT | 6664220 | 2.4798522 | 5.1050367 | 8 |
| CAACC | 15184025 | 2.461622 | 12.7700205 | 85-89 |
| GAAGA | 9273590 | 2.4498916 | 25.956882 | 135-137 |
| AGCGT | 11253205 | 2.4480562 | 14.197546 | 65-69 |
| GTGTG | 9085720 | 2.4241312 | 15.179818 | 135-137 |
| GAAGG | 11323745 | 2.404373 | 11.476366 | 70-74 |
| GATCT | 7910550 | 2.4001124 | 20.032564 | 5 |
| TTCCA | 8586985 | 2.3812582 | 24.090668 | 110-114 |
| TTAAG | 5759820 | 2.3789217 | 28.445312 | 130-134 |
| CGCGG | 20718730 | 2.374008 | 9.552989 | 35-39 |
| AATGA | 6412360 | 2.362635 | 56.364372 | 8 |
| CGGTC | 14708300 | 2.3505056 | 10.800896 | 60-64 |
| CGGAT | 10756255 | 2.3399482 | 19.943047 | 20-24 |
| CTCCA | 12704450 | 2.3087816 | 19.108107 | 95-99 |
| GGAGT | 9670070 | 2.3016212 | 11.985279 | 115-119 |
| GTTAA | 5570245 | 2.3006237 | 27.375732 | 130-134 |
| CCTAC | 12646525 | 2.2982547 | 15.846357 | 90-94 |
| TGGCC | 14291025 | 2.2838218 | 13.159865 | 120-124 |
| GATTG | 6764480 | 2.2455275 | 18.167706 | 35-39 |
| GGTCA | 10289825 | 2.2384794 | 12.113073 | 60-64 |
| CACCG | 17161260 | 2.2361264 | 12.740204 | 45-49 |
| CCACG | 17059495 | 2.2228663 | 7.2035613 | 85-89 |
| GAACG | 11451965 | 2.2224534 | 15.311501 | 40-44 |
| ATTCC | 7980940 | 2.2131958 | 20.330536 | 110-114 |
| CCAGC | 16964620 | 2.210504 | 8.212622 | 100-104 |
| GCTCC | 15131920 | 2.210211 | 10.98947 | 15-19 |
| GCCAC | 16933880 | 2.2064986 | 12.277228 | 1 |
| GGTGA | 9246230 | 2.2007408 | 18.811293 | 115-119 |
| ATTCG | 7199800 | 2.1844661 | 16.692146 | 90-94 |
| GTGAT | 6512790 | 2.161977 | 21.019306 | 120-124 |
| GTTGC | 8853940 | 2.1591074 | 12.71788 | 115-119 |
| ATTCA | 5618755 | 2.1210566 | 87.542915 | 6 |
| TACAA | 6269540 | 2.1113262 | 26.529562 | 7 |
| GGTCG | 12023470 | 2.102266 | 8.802718 | 9 |
| CCGAA | 11842640 | 2.1005936 | 11.036952 | 65-69 |
| GAAGC | 10812410 | 2.0983365 | 15.814222 | 60-64 |
| AACCT | 8472320 | 2.0959246 | 17.499695 | 105-109 |
| CGTTT | 6153330 | 2.0927994 | 18.33546 | 45-49 |
| ATGGC | 9547005 | 2.076884 | 19.293592 | 120-124 |
| TAAGA | 5633215 | 2.0755587 | 28.707352 | 130-134 |
| CCTCC | 15403655 | 2.056385 | 12.693719 | 95-99 |
| ATGCA | 7577455 | 2.0509531 | 17.072868 | 85-89 |
| CTGAT | 6749685 | 2.0478983 | 23.483717 | 105-109 |
| CGAGA | 10533990 | 2.0443044 | 30.842815 | 2 |
| GCTTC | 9085720 | 2.02506 | 11.710183 | 100-104 |
| CTACA | 8173375 | 2.02197 | 37.24458 | 6 |
| AGAAC | 8373860 | 2.0219276 | 20.530119 | 40-44 |
| GATAC | 7429020 | 2.0107772 | 14.941996 | 50-54 |
| TGGCG | 11431965 | 1.9988436 | 11.085433 | 70-74 |
| CACCA | 12317835 | 1.9969577 | 12.943961 | 55-59 |
| AACCC | 12305725 | 1.9949944 | 10.032598 | 90-94 |
| GACAT | 7357380 | 1.9913865 | 13.908931 | 105-109 |
| TGCAA | 7340170 | 1.9867284 | 19.276106 | 85-89 |
| CCTCG | 13569500 | 1.9819996 | 9.4226465 | 20-24 |
| TTCGT | 5814390 | 1.9775229 | 19.705545 | 125-129 |
| CGTTA | 6458565 | 1.9595706 | 24.498854 | 125-129 |
| AACTT | 5177115 | 1.9543393 | 7.445267 | 70-74 |
| GTAGA | 6599055 | 1.954219 | 22.802055 | 35-39 |
| TAGAG | 6548060 | 1.9391176 | 18.760757 | 40-44 |
| GCACC | 14833945 | 1.9328754 | 11.064694 | 45-49 |
| CAGAA | 7988785 | 1.9289485 | 17.246363 | 60-64 |
| GGGAG | 11296030 | 1.9277482 | 10.568705 | 115-119 |
| AGATC | 7108330 | 1.9239776 | 16.847126 | 4 |
| ACCTG | 9517530 | 1.8923885 | 15.166826 | 105-109 |
| TGGTG | 7088190 | 1.8911767 | 10.24606 | 130-134 |
| AAGGC | 9711550 | 1.8846954 | 10.322122 | 70-74 |
| TGAAC | 6945210 | 1.8798267 | 24.658539 | 7 |
| GAAAT | 5074145 | 1.8695695 | 11.205439 | 4 |
| AAGTC | 6766060 | 1.831337 | 14.138502 | 10-14 |
| AGATG | 6153555 | 1.8222903 | 10.748945 | 4 |
| AGTCG | 8364300 | 1.8195951 | 18.32194 | 7 |
| ACGCG | 12735510 | 1.8156103 | 9.554062 | 55-59 |
| GCGTT | 7422125 | 1.8099471 | 13.412161 | 45-49 |
| GCGGA | 11523630 | 1.7974411 | 10.631748 | 35-39 |
| TACCT | 6469840 | 1.7941525 | 16.71241 | 95-99 |
| CGCGC | 17099040 | 1.7907364 | 5.7067947 | 55-59 |
| AAGCG | 9220305 | 1.7893608 | 12.119005 | 65-69 |
| AGGGC | 11465530 | 1.7883788 | 13.11374 | 30-34 |
| TTACC | 6432610 | 1.7838283 | 19.828442 | 75-79 |
| TGAAG | 6005560 | 1.7784637 | 9.827965 | 3 |
| CAGCC | 13540485 | 1.7643365 | 10.569944 | 9 |
| GGGCC | 15345115 | 1.7582846 | 8.907525 | 30-34 |
| TTTTT | 2422585 | 1.7535373 | 5.426124 | 110-114 |
| AGGGT | 7345765 | 1.7484019 | 17.294386 | 115-119 |
| CATAC | 7048435 | 1.7436769 | 13.336463 | 105-109 |
| ACCCT | 9591680 | 1.7430972 | 11.329905 | 90-94 |
| AGCCA | 9825735 | 1.7428443 | 12.757488 | 100-104 |
| GCAAC | 9786785 | 1.7359356 | 14.864105 | 85-89 |
| CGGAG | 11102100 | 1.7316915 | 9.652956 | 85-89 |
| ATATT | 2993005 | 1.724084 | 9.662418 | 4 |
| AACAG | 7102355 | 1.7149137 | 10.567149 | 9 |
| CGGGC | 14950280 | 1.7130435 | 13.095802 | 80-84 |
| CCATT | 6137615 | 1.7020231 | 63.33119 | 4 |
| CGGGA | 10904390 | 1.7008529 | 11.233105 | 110-114 |
| CCCTC | 12729790 | 1.6994245 | 10.805045 | 15-19 |
| GCCAG | 11906240 | 1.6973871 | 8.357271 | 40-44 |
| CAAGA | 7027255 | 1.6967803 | 17.726425 | 9 |
| GCGGG | 13495285 | 1.6918435 | 7.2903185 | 9 |
| GGAGA | 7911295 | 1.6798067 | 11.168319 | 90-94 |
| TCCTC | 8235765 | 1.6777352 | 7.0127764 | 4 |
| TTATT | 2590485 | 1.6727252 | 5.9642735 | 3 |
| GAACC | 9390240 | 1.6655983 | 16.193937 | 9 |
| GCCAA | 9387245 | 1.6650668 | 15.088423 | 100-104 |
| GTCGC | 10354285 | 1.6546986 | 14.671948 | 8 |
| CGTGG | 9459565 | 1.6539754 | 14.672988 | 65-69 |
| CCCCG | 17144165 | 1.6410325 | 5.290741 | 30-34 |
| CCACC | 13760915 | 1.6388357 | 10.080239 | 55-59 |
| GGCCG | 14262430 | 1.6342276 | 7.6658216 | 35-39 |
| TGAAT | 3944305 | 1.6290774 | 9.282035 | 8 |
| CCGGA | 11405570 | 1.6260103 | 10.46651 | 50-54 |
| CCGTA | 8168990 | 1.6242557 | 13.984232 | 35-39 |
| ATACC | 6542285 | 1.6184628 | 12.60283 | 110-114 |
| CTTGC | 7255025 | 1.6170276 | 13.709636 | 8 |
| GTCTA | 5281810 | 1.6025356 | 22.185385 | 4 |
| AAGGG | 7503150 | 1.5931453 | 15.163004 | 30-34 |
| TCGTC | 7134125 | 1.590081 | 22.138369 | 2 |
| TCACC | 8600770 | 1.5630192 | 9.520626 | 65-69 |
| CTACC | 8563070 | 1.556168 | 13.827308 | 90-94 |
| CGCCT | 10624375 | 1.5518261 | 8.336811 | 75-79 |
| AATTT | 2685370 | 1.5468746 | 14.578909 | 6 |
| TTGAA | 3735285 | 1.5427482 | 11.212373 | 9 |
| CGCTC | 10559365 | 1.5423305 | 10.410843 | 15-19 |
| GCCGT | 9611700 | 1.5360276 | 11.765907 | 1 |
| TCGCC | 10472130 | 1.5295889 | 8.796557 | 9 |
| TCCAC | 8401590 | 1.5268222 | 6.9845695 | 75-79 |
| CAGGG | 9740650 | 1.5193342 | 11.048063 | 115-119 |
| TCTAC | 5478140 | 1.5191439 | 20.371037 | 5 |
| TACGC | 7639610 | 1.518998 | 12.129751 | 55-59 |
| CCCTA | 8353190 | 1.5180265 | 13.593866 | 90-94 |
| CACGA | 8549900 | 1.5165423 | 5.079216 | 60-64 |
| GGCGG | 12024460 | 1.5074527 | 6.0385942 | 8 |
| CCCGC | 15681880 | 1.5010631 | 5.41006 | 30-34 |
| CCGTT | 6733590 | 1.5008082 | 19.490679 | 125-129 |
| GTGGT | 5617410 | 1.4987627 | 9.774967 | 130-134 |
| AAATA | 3268640 | 1.4984182 | 6.224179 | 105-109 |
| CCTGA | 7532685 | 1.4977381 | 17.228197 | 105-109 |
| AGGCC | 10481285 | 1.4942416 | 7.529593 | 70-74 |
| TGCCT | 6696555 | 1.4925537 | 9.687235 | 120-124 |
| CTACG | 7489755 | 1.4892021 | 11.450671 | 75-79 |
| CCCTT | 7286370 | 1.4843308 | 8.434394 | 125-129 |
| ACAAG | 6141155 | 1.4828253 | 17.738096 | 8 |
| CCAGA | 8350420 | 1.4811596 | 11.457206 | 60-64 |
| GAGAG | 6962740 | 1.4783999 | 5.1539736 | 100-104 |
| ACTTC | 5315670 | 1.4740894 | 5.130388 | 70-74 |
| AACGC | 8309705 | 1.4739379 | 10.731101 | 45-49 |
| TACGG | 6774050 | 1.4736474 | 10.973592 | 80-84 |
| TCGAA | 5440710 | 1.4726107 | 5.0590987 | 10-14 |
| GGTGT | 5517280 | 1.4720472 | 10.074429 | 130-134 |
| CATGC | 7400935 | 1.4715419 | 12.279456 | 85-89 |
| TCTGA | 4824740 | 1.4638574 | 6.400161 | 15-19 |
| GGCAT | 6706915 | 1.4590425 | 18.280136 | 80-84 |
| CCAAC | 8988965 | 1.4572839 | 12.634408 | 100-104 |
| GCATG | 6698605 | 1.4572347 | 16.835766 | 80-84 |
| CCTTC | 7137310 | 1.4539652 | 8.267711 | 125-129 |
| GGGCA | 9317810 | 1.45338 | 11.108985 | 80-84 |
| CATTC | 5140675 | 1.4255615 | 64.10909 | 5 |
| GTCAC | 7168285 | 1.4252837 | 10.924821 | 60-64 |
| CTATA | 3761245 | 1.4198543 | 6.736098 | 60-64 |
| AGAGG | 6662495 | 1.4146488 | 5.263232 | 100-104 |
| GCGGT | 8063810 | 1.409932 | 11.230368 | 60-64 |
| TAACT | 3727665 | 1.4071779 | 7.9323072 | 70-74 |
| ACCTC | 7728275 | 1.4044605 | 11.558834 | 95-99 |
| CTCGG | 8694090 | 1.389386 | 10.398788 | 20-24 |
| TAGTC | 4552540 | 1.3812704 | 18.125828 | 6 |
| TAACA | 4069000 | 1.3702737 | 12.704606 | 8 |
| CGCAC | 10474165 | 1.3647923 | 12.045234 | 45-49 |
| TATAC | 3601445 | 1.3595304 | 7.9444237 | 60-64 |
| CTCCC | 10178865 | 1.3588765 | 9.492881 | 15-19 |
| CTCCT | 6665275 | 1.3578054 | 7.199339 | 3 |
| CCGGG | 11813940 | 1.3536731 | 7.267896 | 80-84 |
| GCCCC | 14113360 | 1.350925 | 5.320497 | 30-34 |
| CTCTC | 6626385 | 1.349883 | 8.180296 | 1 |
| GCCTC | 9218810 | 1.3465252 | 6.019521 | 120-124 |
| CAAGG | 6936275 | 1.346105 | 12.230463 | 30-34 |
| ATGCG | 6157965 | 1.3396224 | 5.648953 | 55-59 |
| GACGC | 9376680 | 1.336766 | 13.226308 | 50-54 |
| GTAGT | 4025440 | 1.3362796 | 7.158735 | 80-84 |
| ACCAG | 7524840 | 1.334722 | 10.91829 | 60-64 |
| AAAAG | 4059810 | 1.3344182 | 25.492292 | 135-137 |
| AAAGA | 4053535 | 1.3323556 | 16.72001 | 135-137 |
| GGGTG | 6930155 | 1.3257446 | 16.230825 | 115-119 |
| CGTAG | 6050455 | 1.3162342 | 18.153006 | 35-39 |
| CTGAA | 4847150 | 1.3119547 | 5.944588 | 20-24 |
| GCGTG | 7474600 | 1.3069105 | 12.287553 | 65-69 |
| GGACG | 8361425 | 1.3042045 | 13.794105 | 50-54 |
| TGTGC | 5333140 | 1.3005308 | 13.79813 | 135-137 |
| CCGCC | 13510290 | 1.2931994 | 5.0435686 | 75-79 |
| GAATA | 3508460 | 1.2926927 | 9.530708 | 15-19 |
| CAAGT | 4768585 | 1.29069 | 62.42659 | 9 |
| ACAGC | 7270925 | 1.2896837 | 15.402331 | 8 |
| TCTCC | 6290555 | 1.2814698 | 7.4894395 | 2 |
| GTCGT | 5244625 | 1.2789456 | 19.279682 | 1 |
| ACGTC | 6316780 | 1.2559773 | 5.2717476 | 55-59 |
| GGCGC | 10938965 | 1.2534161 | 8.501138 | 70-74 |
| ACAGT | 4628945 | 1.2528943 | 5.9801707 | 15-19 |
| CACGG | 8737005 | 1.245572 | 7.27366 | 85-89 |
| TTGGT | 3345680 | 1.2449757 | 6.8341985 | 7 |
| ACGTT | 4091060 | 1.2412542 | 9.21413 | 135-137 |
| TTTGG | 3333695 | 1.240516 | 5.388789 | 95-99 |
| AGGGA | 5841420 | 1.2403098 | 5.0560837 | 35-39 |
| ACGAA | 5107135 | 1.2331538 | 6.662014 | 125-129 |
| CAGTC | 6170575 | 1.226907 | 7.41544 | 5 |
| ACGGA | 6308905 | 1.224353 | 10.058865 | 85-89 |
| GAGCT | 5597030 | 1.2175949 | 5.0320244 | 115-119 |
| GCCTA | 6109905 | 1.214844 | 10.112028 | 75-79 |
| CGACA | 6742050 | 1.1958743 | 9.438963 | 105-109 |
| ACCGC | 9155955 | 1.1930286 | 11.45474 | 10-14 |
| TAAAT | 2320325 | 1.1923598 | 7.306762 | 105-109 |
| GCTAT | 3876100 | 1.176034 | 5.2414923 | 60-64 |
| GGTTC | 4818375 | 1.175001 | 6.2969847 | 65-69 |
| TCGGA | 5388770 | 1.1722891 | 15.266655 | 20-24 |
| CGTCT | 5247840 | 1.1696587 | 17.255678 | 3 |
| CAACA | 5290930 | 1.1676503 | 5.3019805 | 10-14 |
| GCGAC | 8188390 | 1.1673601 | 7.3990197 | 105-109 |
| GTGCA | 5343915 | 1.1625314 | 12.525766 | 135-137 |
| CGCGA | 8148425 | 1.1616625 | 7.6662736 | 100-104 |
| CACCT | 6388300 | 1.1609466 | 14.50369 | 3 |
| CGCCA | 8898220 | 1.1594454 | 8.48584 | 55-59 |
| AAATT | 2254540 | 1.1585544 | 13.523198 | 5 |
| TTAGT | 2496805 | 1.1559749 | 27.880339 | 5 |
| AGTAG | 3896110 | 1.1537791 | 6.338579 | 80-84 |
| ACCGA | 6499460 | 1.1528447 | 9.034487 | 65-69 |
| GTCCC | 7819010 | 1.1420667 | 34.272133 | 1 |
| AGTCT | 3758210 | 1.1402653 | 7.0896835 | 15-19 |
| CCAGG | 7978090 | 1.1373789 | 9.111937 | 115-119 |
| TTATC | 2679960 | 1.134052 | 6.33106 | 50-54 |
| CACGT | 5673925 | 1.1281573 | 5.0761776 | 135-137 |
| AATAG | 3052190 | 1.1245798 | 6.7117844 | 15-19 |
| ATTTG | 2415010 | 1.1181053 | 12.55439 | 5 |
| CGTAA | 4130050 | 1.1178607 | 9.850403 | 6 |
| TACAG | 4125415 | 1.1166062 | 21.602592 | 7 |
| TCGTG | 4577000 | 1.1161398 | 9.310267 | 130-134 |
| GTGGC | 6375020 | 1.1146525 | 13.431142 | 65-69 |
| GCCCG | 10610740 | 1.1112342 | 9.612428 | 125-129 |
| ATCAT | 2919645 | 1.1021538 | 5.7476196 | 50-54 |
| GCAGT | 5039975 | 1.0964113 | 8.193168 | 4 |
| CACTA | 4423855 | 1.0943953 | 5.2870884 | 70-74 |
| ATAAA | 2387275 | 1.0943807 | 5.623073 | 105-109 |
| TATTG | 2307440 | 1.0683023 | 7.2913504 | 5 |
| AGGTT | 3210760 | 1.0658395 | 7.7413917 | 65-69 |
| CTGCG | 6606710 | 1.0558058 | 7.5292706 | 1 |
| ACGGG | 6733535 | 1.0502882 | 8.198119 | 80-84 |
| CGGAC | 7365805 | 1.05009 | 10.666696 | 50-54 |
| GTAAC | 3864230 | 1.0459126 | 10.121542 | 7 |
| TGCCC | 7133450 | 1.0419319 | 7.592726 | 30-34 |
| TACGT | 3395590 | 1.0302441 | 5.200098 | 60-64 |
| GTCAG | 4719405 | 1.0266737 | 7.975828 | 1 |
| ACGCA | 5773430 | 1.0240649 | 11.026354 | 45-49 |
| TTGGA | 3080320 | 1.0225389 | 5.9662037 | 95-99 |
| GAGTA | 3452700 | 1.0224694 | 5.849656 | 75-79 |
| TCGGT | 4183855 | 1.020268 | 8.162588 | 7 |
| ACGCC | 7777425 | 1.013405 | 9.255488 | 55-59 |
| GGATG | 4232795 | 1.0074685 | 5.188694 | 55-59 |
| TGCGC | 6257050 | 0.9999273 | 7.4419303 | 2 |
| GCGCG | 8650335 | 0.9911787 | 6.3371987 | 55-59 |
| CCGCT | 6738595 | 0.9842582 | 11.1206255 | 10-14 |
| CAGCG | 6870975 | 0.9795456 | 7.6087713 | 45-49 |
| TCCCA | 5375635 | 0.97691494 | 40.56475 | 2 |
| CCGCG | 9318605 | 0.9759124 | 6.114764 | 30-34 |
| TATCA | 2578010 | 0.973188 | 6.4700704 | 50-54 |
| ACGAG | 4935560 | 0.9578314 | 5.1717978 | 30-34 |
| AGCTG | 4379920 | 0.952821 | 5.6521373 | 50-54 |
| TTCGA | 3129340 | 0.94946206 | 5.0112166 | 40-44 |
| CCCAT | 5222630 | 0.9491092 | 41.315876 | 3 |
| AACCG | 5157490 | 0.9148122 | 15.963494 | 9 |
| GCTGG | 5223480 | 0.91330916 | 8.717255 | 5 |
| GGCGA | 5791190 | 0.90330243 | 11.728505 | 1 |
| GCCGC | 8583940 | 0.8989729 | 5.782946 | 70-74 |
| GCGCA | 6237805 | 0.8892791 | 5.644816 | 2 |
| GATGA | 2974570 | 0.88087785 | 9.387954 | 1 |
| CAGAT | 3215780 | 0.87039965 | 8.699698 | 3 |
| GAGGT | 3655260 | 0.8700065 | 5.366655 | 65-69 |
| TATAT | 1458610 | 0.84021455 | 8.1265955 | 3 |
| GTCCA | 4216215 | 0.83831805 | 5.1909285 | 60-64 |
| GCGAG | 5291055 | 0.82529205 | 23.94315 | 1 |
| CTGGC | 5105865 | 0.8159586 | 7.738286 | 6 |
| CCCGT | 5568500 | 0.8133508 | 10.810914 | 125-129 |
| GGCCC | 7765195 | 0.81322795 | 6.836774 | 125-129 |
| ACTCT | 2925110 | 0.81116277 | 5.3205624 | 5 |
| TTATA | 1393425 | 0.80266553 | 8.150188 | 2 |
| TGCGG | 4587740 | 0.80215204 | 6.2239804 | 50-54 |
| TTCGG | 3223395 | 0.78605175 | 6.7291 | 70-74 |
| CTTAT | 1814090 | 0.76765037 | 6.1150174 | 1 |
| ACCTA | 3090130 | 0.7644517 | 19.0524 | 4 |
| GTCGG | 4350930 | 0.76074654 | 5.822773 | 1 |
| ATAGC | 2778045 | 0.75192004 | 6.7218766 | 20-24 |
| GTTAG | 2258440 | 0.74970865 | 19.77651 | 4 |
| GAATG | 2440890 | 0.72283584 | 6.5351424 | 9 |
| GGGCG | 5735775 | 0.71906835 | 6.247845 | 2 |
| TCTAA | 1867785 | 0.70508105 | 5.595309 | 100-104 |
| CGCTG | 4395435 | 0.7024261 | 7.6219745 | 4 |
| TGACT | 2241795 | 0.6801752 | 5.0218916 | 3 |
| TCAGA | 2464020 | 0.6669244 | 8.3138895 | 2 |
| CGAAT | 2440780 | 0.66063416 | 6.230447 | 15-19 |
| CTCGT | 2918750 | 0.65054214 | 8.075865 | 1 |
| AGCGC | 3785110 | 0.539616 | 5.3982444 | 1 |
| GCAGA | 2777785 | 0.5390776 | 5.1624684 | 1 |
| CGCAG | 3729580 | 0.5316994 | 5.2088175 | 3 |
| AAGCC | 2519365 | 0.44687355 | 5.6221113 | 5 |

Produced by FastQC (version 0.10.1)
