# Supplementary material for: Freshwater sponge hosts and their green algae symbionts: a tractable model to understand intracellular symbiosis
Source: PeerJ. 2021 Feb 11;9:e10654. doi: 10.7717/peerj.10654 (PMC7882143; doi:10.7717/peerj.10654)
Supplement: Supplemental Information 31 [file peerj-09-10654-s031.zip › EmInf1_Clean_Data2.fq_fastqc/fastqc_report.html]

EmInf1\_Clean\_Data2.fq.gz FastQC Report


FastQC Report

Tue 10 Sep 2019  
EmInf1\_Clean\_Data2.fq.gz

## Summary

- Basic Statistics
- Per base sequence quality
- Per sequence quality scores
- Per base sequence content
- Per base GC content
- Per sequence GC content
- Per base N content
- Sequence Length Distribution
- Sequence Duplication Levels
- Overrepresented sequences
- Kmer Content

## Basic Statistics

| Measure | Value |
| --- | --- |
| Filename | EmInf1\_Clean\_Data2.fq.gz |
| File type | Conventional base calls |
| Encoding | Sanger / Illumina 1.9 |
| Total Sequences | 33511120 |
| Filtered Sequences | 0 |
| Sequence length | 100-141 |
| %GC | 58 |

## Per base sequence quality

## Per sequence quality scores

## Per base sequence content

## Per base GC content

## Per sequence GC content

## Per base N content

## Sequence Length Distribution

## Sequence Duplication Levels

## Overrepresented sequences

| Sequence | Count | Percentage | Possible Source |
| --- | --- | --- | --- |
| CTCGGAGACGCCGGAGGGGACCCTGGGAAGAGTTCTCTTTTCTTCTTAAC | 822183 | 2.4534632086304486 | No Hit |
| GGCAACTCCCGGTATGTCGCGAAGCGCGAATCTCCGTGGCCCGTAGGCGG | 642124 | 1.9161520116307662 | No Hit |
| CGGAGACGCCGGAGGGGACCCTGGGAAGAGTTCTCTTTTCTTCTTAACGG | 584927 | 1.7454713539863782 | No Hit |
| GCAGGTGCACACCACGAAGGGAGGCAACTCCCGGTATGTCGCGAAGCGCG | 506828 | 1.512417370711573 | No Hit |
| GTGCACACCACGAAGGGAGGCAACTCCCGGTATGTCGCGAAGCGCGAATC | 456524 | 1.3623060046933675 | No Hit |
| GTTTCGACGTGCCGGCACGCCGGCGAGGACTTCGGCCCTCGCAGGCGTAG | 441658 | 1.3179446106247716 | No Hit |
| GGTGCACACCACGAAGGGAGGCAACTCCCGGTATGTCGCGAAGCGCGAAT | 373643 | 1.1149821313044745 | No Hit |
| CAGGTTTCGACGTGCCGGCACGCCGGCGAGGACTTCGGCCCTCGCAGGCG | 303145 | 0.904610171190936 | No Hit |
| AGCATATGTAGCCAGGCGTCGCCCCGCGTGAGGTTCAGGTTTCGACGTGC | 291303 | 0.8692726474077859 | No Hit |
| CACGAAGGGAGGCAACTCCCGGTATGTCGCGAAGCGCGAATCTCCGTGGC | 280245 | 0.8362746455504919 | No Hit |
| GCCAGGCGTCGCCCCGCGTGAGGTTCAGGTTTCGACGTGCCGGCACGCCG | 259564 | 0.7745608025037659 | No Hit |
| GGAGACGCCGGAGGGGACCCTGGGAAGAGTTCTCTTTTCTTCTTAACGGG | 259216 | 0.7735223412407584 | No Hit |
| GGGACGTATAGCCGCGTCGTTCGGAGCGCGCCCGCGACCGAGGAGAGGGT | 250394 | 0.7471967514066973 | No Hit |
| GGGAAGAGTTCTCTTTTCTTCTTAACGGGCCATCACCCTGGAATCAGGTT | 228473 | 0.6817826440894843 | No Hit |
| GCCGGAGGGGACCCTGGGAAGAGTTCTCTTTTCTTCTTAACGGGCCATCA | 217864 | 0.6501244959881973 | No Hit |
| GGAAGCTCCCTGTAGCACGGTGCAACTCGCCATCTTGGCGACCGGCACCC | 202973 | 0.605688499817374 | No Hit |
| GGAAGAGTTCTCTTTTCTTCTTAACGGGCCATCACCCTGGAATCAGGTTG | 188784 | 0.5633473306771006 | No Hit |
| GGCGTCGCCCCGCGTGAGGTTCAGGTTTCGACGTGCCGGCACGCCGGCGA | 186469 | 0.5564391760108286 | No Hit |
| CGACGTGCCGGCACGCCGGCGAGGACTTCGGCCCTCGCAGGCGTAGCCGA | 176215 | 0.525840377761173 | No Hit |
| GGGAAGCATATGTAGCCAGGCGTCGCCCCGCGTGAGGTTCAGGTTTCGAC | 175537 | 0.5238171687487616 | No Hit |
| GACGTATAGCCGCGTCGTTCGGAGCGCGCCCGCGACCGAGGAGAGGGTCT | 172170 | 0.5137697576207539 | No Hit |
| CACATTTCCCCGCGGGCTGCAGGTGCACACCACGAAGGGAGGCAACTCCC | 170976 | 0.5102067612183657 | No Hit |
| GTGCCGGCACGCCGGCGAGGACTTCGGCCCTCGCAGGCGTAGCCGACCGC | 170094 | 0.5075747990517775 | No Hit |
| GGCTGCAGGTGCACACCACGAAGGGAGGCAACTCCCGGTATGTCGCGAAG | 162699 | 0.48550749721286546 | No Hit |
| CTCTTTTCTTCTTAACGGGCCATCACCCTGGAATCAGGTTGGCTGGAGGT | 152789 | 0.455935223889861 | No Hit |
| GTCGGAAGCGAGGGTCGACGAAGCGGGCTGGCGGGGGGGCCCTCTCGGGG | 142674 | 0.4257512133285906 | No Hit |
| GCTGCAGGTGCACACCACGAAGGGAGGCAACTCCCGGTATGTCGCGAAGC | 136086 | 0.40609206734958425 | No Hit |
| GAGACGCCGGAGGGGACCCTGGGAAGAGTTCTCTTTTCTTCTTAACGGGC | 136076 | 0.4060622265086932 | No Hit |
| GACGTGCCGGCACGCCGGCGAGGACTTCGGCCCTCGCAGGCGTAGCCGAC | 134486 | 0.4013175328070205 | No Hit |
| CGTGCCGGCACGCCGGCGAGGACTTCGGCCCTCGCAGGCGTAGCCGACCG | 131647 | 0.3928457180780589 | No Hit |
| GGACGTATAGCCGCGTCGTTCGGAGCGCGCCCGCGACCGAGGAGAGGGTC | 130386 | 0.38908278804170077 | No Hit |
| GTTCTCTTTTCTTCTTAACGGGCCATCACCCTGGAATCAGGTTGGCTGGA | 129322 | 0.3859077225708959 | No Hit |
| TCGGAGACGCCGGAGGGGACCCTGGGAAGAGTTCTCTTTTCTTCTTAACG | 122501 | 0.36555328499912865 | No Hit |
| GTCCCGACTTTGCGGAAGGGATGTATTTATTAGATCCAAAGCCAATGCGG | 120884 | 0.3607280210270501 | No Hit |
| TGCAGGTGCACACCACGAAGGGAGGCAACTCCCGGTATGTCGCGAAGCGC | 115685 | 0.34521376784780694 | No Hit |
| GAAGAGTTCTCTTTTCTTCTTAACGGGCCATCACCCTGGAATCAGGTTGG | 115292 | 0.3440410228007897 | No Hit |
| GGGGAAGCTCCCTGTAGCACGGTGCAACTCGCCATCTTGGCGACCGGCAC | 113197 | 0.33778936663412024 | No Hit |
| GGCGAAGTTAGGGACGTATAGCCGCGTCGTTCGGAGCGCGCCCGCGACCG | 108851 | 0.3248205371828814 | No Hit |
| GAAGCGGGCTGGCGGGGGGGCCCTCTCGGGGGTCCTGCCGCCGGAGCGTG | 106783 | 0.3186494512866177 | No Hit |
| GTTCAGGTTTCGACGTGCCGGCACGCCGGCGAGGACTTCGGCCCTCGCAG | 105791 | 0.3156892398702281 | No Hit |
| CCGGTATGTCGCGAAGCGCGAATCTCCGTGGCCCGTAGGCGGCCTTCGGT | 104284 | 0.31119222514795086 | No Hit |
| CATTTCCCCGCGGGCTGCAGGTGCACACCACGAAGGGAGGCAACTCCCGG | 102362 | 0.30545681552869614 | No Hit |
| AGGCAACTCCCGGTATGTCGCGAAGCGCGAATCTCCGTGGCCCGTAGGCG | 95723 | 0.2856454812611456 | No Hit |
| CGCAACGACACATTTCCCCGCGGGCTGCAGGTGCACACCACGAAGGGAGG | 94171 | 0.2810141827548587 | No Hit |
| GCAACTCCCGGTATGTCGCGAAGCGCGAATCTCCGTGGCCCGTAGGCGGC | 92619 | 0.2763828842485718 | No Hit |
| ATTTCCCCGCGGGCTGCAGGTGCACACCACGAAGGGAGGCAACTCCCGGT | 90878 | 0.27118759384944463 | No Hit |
| CGTATAGCCGCGTCGTTCGGAGCGCGCCCGCGACCGAGGAGAGGGTCTCT | 89182 | 0.26612658723432703 | No Hit |
| GGCGAATTGTAGCCGAGAGAGGCACCTGCGCTCGGCAGGCGGTCGACCAA | 88225 | 0.26327081876105607 | No Hit |
| CCCCGCGTGAGGTTCAGGTTTCGACGTGCCGGCACGCCGGCGAGGACTTC | 87827 | 0.2620831532935933 | No Hit |
| CCGGAGGGGACCCTGGGAAGAGTTCTCTTTTCTTCTTAACGGGCCATCAC | 87498 | 0.2611013896282786 | No Hit |
| TTCGACGTGCCGGCACGCCGGCGAGGACTTCGGCCCTCGCAGGCGTAGCC | 87339 | 0.26062692025811135 | No Hit |
| GTCGACGAAGCGGGCTGGCGGGGGGGCCCTCTCGGGGGTCCTGCCGCCGG | 85839 | 0.2561507941244578 | No Hit |
| CGGAGGGGACCCTGGGAAGAGTTCTCTTTTCTTCTTAACGGGCCATCACC | 84343 | 0.25168660432716067 | No Hit |
| CTGGAATCAGGTTGGCTGGAGGTAGGGTTGCATGCCCGGTAAAGCGCCAC | 82083 | 0.2449425742857893 | No Hit |
| CCCGACTTTGCGGAAGGGATGTATTTATTAGATCCAAAGCCAATGCGGGG | 81135 | 0.2421136625693203 | No Hit |
| AGACGCCGGAGGGGACCCTGGGAAGAGTTCTCTTTTCTTCTTAACGGGCC | 79617 | 0.2375838229220629 | No Hit |
| GACGCCGGAGGGGACCCTGGGAAGAGTTCTCTTTTCTTCTTAACGGGCCA | 78929 | 0.23553077306876047 | No Hit |
| CCCGCGTGAGGTTCAGGTTTCGACGTGCCGGCACGCCGGCGAGGACTTCG | 78578 | 0.2344833595534855 | No Hit |
| CAGGTGCACACCACGAAGGGAGGCAACTCCCGGTATGTCGCGAAGCGCGA | 77851 | 0.2323139304207081 | No Hit |
| AGCGAGGGTCGACGAAGCGGGCTGGCGGGGGGGCCCTCTCGGGGGTCCTG | 77339 | 0.2307860793670877 | No Hit |
| CTTTTCTTCTTAACGGGCCATCACCCTGGAATCAGGTTGGCTGGAGGTAG | 75662 | 0.22578177034966304 | No Hit |
| ACGAAGGGAGGCAACTCCCGGTATGTCGCGAAGCGCGAATCTCCGTGGCC | 73614 | 0.2196703661351814 | No Hit |
| AAGCGAGGGTCGACGAAGCGGGCTGGCGGGGGGGCCCTCTCGGGGGTCCT | 71624 | 0.21373203879786767 | No Hit |
| GTCGATTCAGACATTTGGCATTTGCGCTTGGCTGAAAAGCCAATGGCGCG | 70028 | 0.20896944059166034 | No Hit |
| CGAAGGGAGGCAACTCCCGGTATGTCGCGAAGCGCGAATCTCCGTGGCCC | 68571 | 0.20462163007383816 | No Hit |
| GGGAGGCAACTCCCGGTATGTCGCGAAGCGCGAATCTCCGTGGCCCGTAG | 66423 | 0.1982118174504463 | No Hit |
| CTGGGAAGAGTTCTCTTTTCTTCTTAACGGGCCATCACCCTGGAATCAGG | 65394 | 0.19514119492275997 | No Hit |
| GGCGGTGCTGTTACGGCGACCGGGTGGTGCCCTGACCCGCCTCTCGGGGC | 64575 | 0.1926972300537851 | No Hit |
| GGCGTGTGCCTGTAACCGTAGTGAATCAACGGGGCTTGATCTGGCGAATA | 63689 | 0.19005333155084045 | No Hit |
| GTCGCGAAGCGCGAATCTCCGTGGCCCGTAGGCGGCCTTCGGTGACCGCG | 62665 | 0.18699762944359963 | No Hit |
| GTCTCTTCGACCCGCCAGCGCAGGCCTTCGTGGCCGGAGCTCCCGCGTTC | 62488 | 0.1864694465598285 | No Hit |
| GGGGACCCTGGGAAGAGTTCTCTTTTCTTCTTAACGGGCCATCACCCTGG | 62415 | 0.18625160842132402 | No Hit |
| GCGAGGGTCGACGAAGCGGGCTGGCGGGGGGGCCCTCTCGGGGGTCCTGC | 62357 | 0.1860785315441561 | No Hit |
| GCATATGTAGCCAGGCGTCGCCCCGCGTGAGGTTCAGGTTTCGACGTGCC | 61124 | 0.18239915586229288 | No Hit |
| CACCACGAAGGGAGGCAACTCCCGGTATGTCGCGAAGCGCGAATCTCCGT | 60226 | 0.17971944835027895 | No Hit |
| GCCCTGACCCGCCTCTCGGGGCGAAGTTAGGGACGTATAGCCGCGTCGTT | 59082 | 0.17630565615234584 | No Hit |
| CTTCGACCCGCCAGCGCAGGCCTTCGTGGCCGGAGCTCCCGCGTTCCGGT | 57099 | 0.17038821740365587 | No Hit |
| TTTCGACGTGCCGGCACGCCGGCGAGGACTTCGGCCCTCGCAGGCGTAGC | 56989 | 0.1700599681538546 | No Hit |
| AAGCATATGTAGCCAGGCGTCGCCCCGCGTGAGGTTCAGGTTTCGACGTG | 56930 | 0.16988390719259755 | No Hit |
| GGAGGGGACCCTGGGAAGAGTTCTCTTTTCTTCTTAACGGGCCATCACCC | 56003 | 0.16711766124199967 | No Hit |
| GCCGCGTCGTTCGGAGCGCGCCCGCGACCGAGGAGAGGGTCTCTTCGACC | 55702 | 0.16621945193117985 | No Hit |
| GCCCCGCGTGAGGTTCAGGTTTCGACGTGCCGGCACGCCGGCGAGGACTT | 54454 | 0.1624953149879801 | No Hit |
| GAAGCTCCCTGTAGCACGGTGCAACTCGCCATCTTGGCGACCGGCACCCA | 53541 | 0.15977084621462964 | No Hit |
| AAGCGGGCTGGCGGGGGGGCCCTCTCGGGGGTCCTGCCGCCGGAGCGTGG | 53111 | 0.15848769005631563 | No Hit |
| AGCGGGCTGGCGGGGGGGCCCTCTCGGGGGTCCTGCCGCCGGAGCGTGGA | 52842 | 0.15768497143634708 | No Hit |
| CCCTGACCCGCCTCTCGGGGCGAAGTTAGGGACGTATAGCCGCGTCGTTC | 52350 | 0.15621680206450872 | No Hit |
| CTCTTCGACCCGCCAGCGCAGGCCTTCGTGGCCGGAGCTCCCGCGTTCCG | 48325 | 0.1442058636058717 | No Hit |
| GAAGCATATGTAGCCAGGCGTCGCCCCGCGTGAGGTTCAGGTTTCGACGT | 47545 | 0.14187827801637187 | No Hit |
| CCTGGGAAGAGTTCTCTTTTCTTCTTAACGGGCCATCACCCTGGAATCAG | 47131 | 0.1406428672034835 | No Hit |
| GCAACAAGTCCCGACTTTGCGGAAGGGATGTATTTATTAGATCCAAAGCC | 47095 | 0.1405354401762758 | No Hit |
| CAACTCCCGGTATGTCGCGAAGCGCGAATCTCCGTGGCCCGTAGGCGGCC | 45591 | 0.13604737770626588 | No Hit |
| GACACATTTCCCCGCGGGCTGCAGGTGCACACCACGAAGGGAGGCAACTC | 45260 | 0.13505964587277297 | No Hit |
| GGGACCCTGGGAAGAGTTCTCTTTTCTTCTTAACGGGCCATCACCCTGGA | 45205 | 0.13489552124787235 | No Hit |
| GCGTGAGGTTCAGGTTTCGACGTGCCGGCACGCCGGCGAGGACTTCGGCC | 44599 | 0.13308716628987632 | No Hit |
| GAGGCAACTCCCGGTATGTCGCGAAGCGCGAATCTCCGTGGCCCGTAGGC | 44521 | 0.13285440773092633 | No Hit |
| CCCGGTATGTCGCGAAGCGCGAATCTCCGTGGCCCGTAGGCGGCCTTCGG | 43461 | 0.1296912785964778 | No Hit |
| AGCCAGGCGTCGCCCCGCGTGAGGTTCAGGTTTCGACGTGCCGGCACGCC | 42529 | 0.1269101122254344 | No Hit |
| CCTGACCCGCCTCTCGGGGCGAAGTTAGGGACGTATAGCCGCGTCGTTCG | 42529 | 0.1269101122254344 | No Hit |
| GTAATTCTAGAGCTAATACATGCAACAAGTCCCGACTTTGCGGAAGGGAT | 41340 | 0.12336203624349171 | No Hit |
| GCCGAGAGAGGCACCTGCGCTCGGCAGGCGGTCGACCAAAGTTGACCTGG | 40891 | 0.12202218248748475 | No Hit |
| CATGCAACAAGTCCCGACTTTGCGGAAGGGATGTATTTATTAGATCCAAA | 40476 | 0.12078378759050727 | No Hit |
| TGGGAAGAGTTCTCTTTTCTTCTTAACGGGCCATCACCCTGGAATCAGGT | 40462 | 0.12074201041325984 | No Hit |
| GGACCCTGGGAAGAGTTCTCTTTTCTTCTTAACGGGCCATCACCCTGGAA | 40311 | 0.12029141371580539 | No Hit |
| GTATAGCCGCGTCGTTCGGAGCGCGCCCGCGACCGAGGAGAGGGTCTCTT | 39609 | 0.11819658668525551 | No Hit |
| CGTCGCCCCGCGTGAGGTTCAGGTTTCGACGTGCCGGCACGCCGGCGAGG | 39513 | 0.11791011461270168 | No Hit |
| GCACACCACGAAGGGAGGCAACTCCCGGTATGTCGCGAAGCGCGAATCTC | 39473 | 0.11779075124913758 | No Hit |
| CAGGCGTCGCCCCGCGTGAGGTTCAGGTTTCGACGTGCCGGCACGCCGGC | 39188 | 0.11694028728374342 | No Hit |
| GCGAACTCGGAGACGCCGGAGGGGACCCTGGGAAGAGTTCTCTTTTCTTC | 38474 | 0.11480965124412434 | No Hit |
| GGCCAACGTGGGTTGCGGGCGGTGCTGTTACGGCGACCGGGTGGTGCCCT | 38445 | 0.11472311280554036 | No Hit |
| CCCCGCGGGCTGCAGGTGCACACCACGAAGGGAGGCAACTCCCGGTATGT | 37002 | 0.11041707946496566 | No Hit |
| GTGGGTTGCGGGCGGTGCTGTTACGGCGACCGGGTGGTGCCCTGACCCGC | 36907 | 0.11013359147650094 | No Hit |
| GGCGACCGGGTGGTGCCCTGACCCGCCTCTCGGGGCGAAGTTAGGGACGT | 36869 | 0.11002019628111506 | No Hit |
| GCTCCCTGTAGCACGGTGCAACTCGCCATCTTGGCGACCGGCACCCACCA | 36429 | 0.10870719928191001 | No Hit |
| TTCCCCGCGGGCTGCAGGTGCACACCACGAAGGGAGGCAACTCCCGGTAT | 36422 | 0.1086863106932863 | No Hit |
| CTCGGGGCGAAGTTAGGGACGTATAGCCGCGTCGTTCGGAGCGCGCCCGC | 36089 | 0.10769261069161522 | No Hit |
| GTCGGGCTGCGGTCGGAAGCGAGGGTCGACGAAGCGGGCTGGCGGGGGGG | 35841 | 0.10695255783751782 | No Hit |
| GTCGCCCCGCGTGAGGTTCAGGTTTCGACGTGCCGGCACGCCGGCGAGGA | 35648 | 0.10637662960832105 | No Hit |
| CTCCGGCGCACAGCCGGCGAATTGTAGCCGAGAGAGGCACCTGCGCTCGG | 35618 | 0.106287107085648 | No Hit |
| CGGCGACCGGGTGGTGCCCTGACCCGCCTCTCGGGGCGAAGTTAGGGACG | 35483 | 0.10588425573361916 | No Hit |
| AAATCTCCGGCGCACAGCCGGCGAATTGTAGCCGAGAGAGGCACCTGCGC | 35387 | 0.10559778366106534 | No Hit |
| GCCGGCACGCCGGCGAGGACTTCGGCCCTCGCAGGCGTAGCCGACCGCCG | 35322 | 0.1054038181952737 | No Hit |
| GTGAGGTTCAGGTTTCGACGTGCCGGCACGCCGGCGAGGACTTCGGCCCT | 34466 | 0.10284944221500207 | No Hit |
| AAGGGAGGCAACTCCCGGTATGTCGCGAAGCGCGAATCTCCGTGGCCCGT | 34425 | 0.10272709476734888 | No Hit |
| CTTTGCGGAAGGGATGTATTTATTAGATCCAAAGCCAATGCGGGGGGCAA | 34099 | 0.10175428335430149 | No Hit |

## Kmer Content

| Sequence | Count | Obs/Exp Overall | Obs/Exp Max | Max Obs/Exp Position |
| --- | --- | --- | --- | --- |
| TCTCT | 16874275 | 5.0414906 | 17.543163 | 135-137 |
| TTCTC | 14742080 | 4.404459 | 19.890944 | 130-134 |
| TTTCT | 10887770 | 4.254227 | 21.620565 | 35-39 |
| TTCTT | 10865000 | 4.2453303 | 29.634367 | 40-44 |
| ATCTC | 12711885 | 3.960363 | 10.359854 | 120-124 |
| GAATC | 13123590 | 3.8849208 | 14.326048 | 60-64 |
| CTCTT | 12363305 | 3.6937578 | 10.492181 | 30-34 |
| ATCAA | 8270365 | 3.6642087 | 12.628505 | 85-89 |
| CTTCT | 11284350 | 3.3714006 | 15.915322 | 110-114 |
| TCAAA | 7497525 | 3.3217998 | 12.353584 | 85-89 |
| CATTT | 7893260 | 3.2160985 | 11.282633 | 3 |
| CTTCG | 15312080 | 3.1873753 | 14.155981 | 70-74 |
| GTTCT | 10854245 | 2.9549255 | 16.232763 | 130-134 |
| TATGT | 7955820 | 2.9537318 | 22.038485 | 5 |
| GCGAA | 14309020 | 2.9512403 | 9.421226 | 40-44 |
| AAGAT | 7284935 | 2.9409952 | 11.2025 | 110-114 |
| AATCT | 6778315 | 2.879957 | 11.406501 | 45-49 |
| TTGAA | 7435560 | 2.8786645 | 14.06418 | 135-137 |
| AGGTT | 11071530 | 2.863907 | 11.523725 | 2 |
| GCCAA | 12583585 | 2.8483014 | 9.219482 | 80-84 |
| ATTTC | 6943715 | 2.829208 | 10.693907 | 4 |
| CATCA | 8699855 | 2.8263657 | 11.986622 | 50-54 |
| AATCA | 6366885 | 2.8208663 | 14.505196 | 60-64 |
| TATCA | 6612325 | 2.8094316 | 11.999598 | 85-89 |
| TCTTC | 9396680 | 2.8074257 | 10.274388 | 35-39 |
| CCAAT | 8606325 | 2.7959805 | 10.617839 | 100-104 |
| TGGCC | 18944625 | 2.7475796 | 10.370508 | 75-79 |
| CGAAG | 13272865 | 2.7375326 | 14.027968 | 3 |
| CAACT | 8326080 | 2.7049356 | 30.195702 | 3 |
| AATCC | 8321630 | 2.7034898 | 9.489228 | 100-104 |
| CTGGA | 13533665 | 2.6768181 | 10.796234 | 60-64 |
| GGCAA | 12924940 | 2.6657734 | 19.799387 | 1 |
| GGTTG | 15300880 | 2.6444883 | 9.84719 | 85-89 |
| AACTC | 8122370 | 2.6387556 | 31.849234 | 4 |
| CCGGT | 18178605 | 2.636482 | 12.937852 | 9 |
| CCATC | 11041870 | 2.630388 | 16.622921 | 50-54 |
| AAGCG | 12371060 | 2.5515354 | 10.652799 | 100-104 |
| ACACA | 7512575 | 2.5450506 | 6.45735 | 8 |
| TTCGG | 13386370 | 2.5390718 | 7.231758 | 70-74 |
| TCACC | 10613085 | 2.5282433 | 10.788707 | 55-59 |
| GACCG | 16629210 | 2.5149367 | 5.978763 | 80-84 |
| TCTTT | 6398955 | 2.5002923 | 12.987905 | 30-34 |
| TTTTC | 6395175 | 2.4988155 | 19.93538 | 35-39 |
| GTAAA | 6029730 | 2.4342575 | 13.704113 | 95-99 |
| CTTAA | 5726885 | 2.4332275 | 14.339078 | 40-44 |
| GACTT | 8525645 | 2.420279 | 6.4721103 | 130-134 |
| CGGTA | 12177365 | 2.408556 | 10.398399 | 95-99 |
| CTTTT | 6119955 | 2.3912776 | 17.509193 | 35-39 |
| GCTGG | 18061280 | 2.3868542 | 8.725988 | 75-79 |
| CAGGT | 12044880 | 2.3823516 | 14.242848 | 2 |
| ACGCT | 10941400 | 2.3749974 | 8.343518 | 105-109 |
| GGCCA | 15633800 | 2.3643951 | 6.7981496 | 50-54 |
| CCTTC | 10301580 | 2.3533676 | 10.99122 | 70-74 |
| AAAGC | 7608765 | 2.3487396 | 13.875041 | 100-104 |
| TCTTA | 5755875 | 2.3452241 | 22.135248 | 40-44 |
| TAAAG | 5786455 | 2.3360448 | 13.252793 | 95-99 |
| CAAAC | 6886985 | 2.3331184 | 10.859634 | 90-94 |
| ACCCT | 9792165 | 2.3326843 | 13.3316145 | 55-59 |
| CAAGA | 7551525 | 2.3310704 | 8.801647 | 110-114 |
| CACAC | 9361250 | 2.325425 | 17.961693 | 8 |
| CGTAT | 8189915 | 2.3249714 | 10.259775 | 5 |
| CGACC | 13940345 | 2.3137472 | 5.125897 | 50-54 |
| TTAAC | 5416235 | 2.3012393 | 13.761323 | 40-44 |
| AGCGC | 15119370 | 2.2865946 | 8.697417 | 100-104 |
| AAAAT | 3779835 | 2.28385 | 11.585621 | 125-129 |
| AGATC | 7601285 | 2.2501762 | 9.67509 | 115-119 |
| TGGAA | 8322985 | 2.2450278 | 14.542126 | 60-64 |
| GCTTC | 10748765 | 2.237472 | 9.429475 | 110-114 |
| CGCGA | 14754925 | 2.2314777 | 8.721031 | 40-44 |
| GGAGG | 17604870 | 2.210622 | 7.7141914 | 8 |
| CCGCG | 19805795 | 2.1963859 | 5.540554 | 85-89 |
| GCAAC | 9679185 | 2.190889 | 23.743454 | 2 |
| CGGTG | 16550090 | 2.1871457 | 8.074961 | 125-129 |
| GAAGC | 10427890 | 2.150756 | 8.237807 | 2 |
| TGACC | 9856470 | 2.139497 | 6.475678 | 75-79 |
| ATCAC | 6572200 | 2.1351438 | 10.815981 | 50-54 |
| GGAAT | 7909965 | 2.1336203 | 18.80282 | 60-64 |
| GGTGC | 15992550 | 2.113465 | 11.465998 | 125-129 |
| GAAGA | 7498185 | 2.1090653 | 12.957209 | 25-29 |
| ATGTC | 7427115 | 2.108426 | 7.808761 | 30-34 |
| TGAAA | 5186465 | 2.0938237 | 7.6587324 | 120-124 |
| CGAAT | 7061250 | 2.0903118 | 12.694429 | 45-49 |
| ACTCC | 8759640 | 2.0867164 | 21.977255 | 5 |
| CGCTT | 10019480 | 2.0856636 | 6.8592095 | 105-109 |
| TGGAG | 11556255 | 2.0827332 | 10.09212 | 75-79 |
| TGCAT | 7320955 | 2.078289 | 10.204791 | 85-89 |
| CAATC | 6337670 | 2.058951 | 13.23573 | 100-104 |
| TTCGA | 7244095 | 2.05647 | 17.699505 | 3 |
| TTGCA | 7241350 | 2.0556905 | 15.713696 | 85-89 |
| CTCTA | 6577510 | 2.0492105 | 17.176903 | 135-137 |
| GGGTT | 11715520 | 2.0248218 | 7.9769793 | 85-89 |
| GAGGA | 10676565 | 2.0065007 | 5.283001 | 25-29 |
| CCCTG | 12577135 | 2.001856 | 6.00052 | 15-19 |
| CGTTC | 9591415 | 1.9965571 | 10.870793 | 130-134 |
| GTTGC | 10513415 | 1.9941416 | 11.52426 | 85-89 |
| TGGTG | 11493515 | 1.9864523 | 10.7041 | 115-119 |
| ACTTC | 6330910 | 1.9723827 | 7.531994 | 25-29 |
| ATCAG | 6648335 | 1.9680783 | 13.079408 | 65-69 |
| ACGAA | 6373660 | 1.9674767 | 15.146528 | 2 |
| TCTCA | 6310500 | 1.9660242 | 7.8889027 | 70-74 |
| GCATT | 6921515 | 1.9648949 | 7.15898 | 65-69 |
| TTGTA | 5248660 | 1.9486531 | 8.69522 | 125-129 |
| CGTAG | 9851015 | 1.9484283 | 9.422403 | 60-64 |
| TCTAC | 6215985 | 1.9365782 | 26.461512 | 135-137 |
| GCCAT | 8917435 | 1.9356652 | 11.516157 | 50-54 |
| AGGCG | 14033690 | 1.9339288 | 5.317162 | 45-49 |
| GTATC | 6737415 | 1.9126323 | 11.767039 | 85-89 |
| GTAGG | 10581920 | 1.9071329 | 10.909563 | 80-84 |
| AATAT | 3274455 | 1.897328 | 6.2970805 | 70-74 |
| AGGGA | 10085080 | 1.89534 | 7.912658 | 6 |
| TTCTG | 6961200 | 1.8950953 | 15.314914 | 110-114 |
| GGCTG | 14303050 | 1.8901924 | 6.1195993 | 70-74 |
| GCGTA | 9523610 | 1.8836709 | 7.466562 | 80-84 |
| CTTGA | 6629995 | 1.8821378 | 12.230113 | 135-137 |
| CTGGC | 12941445 | 1.8769256 | 7.065914 | 95-99 |
| GAAAA | 4446645 | 1.8719419 | 5.7217317 | 125-129 |
| GCCGG | 18256675 | 1.844808 | 8.961376 | 8 |
| GTGGC | 13947400 | 1.8431922 | 7.8724318 | 115-119 |
| CACCA | 7397430 | 1.8375931 | 16.667177 | 6 |
| GCCCT | 11520850 | 1.8337313 | 5.230205 | 35-39 |
| CGGAG | 13207870 | 1.8201258 | 20.65287 | 3 |
| TAGCC | 8356355 | 1.8138742 | 17.28111 | 9 |
| GCCTT | 8697060 | 1.8103875 | 7.818905 | 70-74 |
| TCGAC | 8299130 | 1.8014528 | 13.782846 | 4 |
| TGAAT | 4651615 | 1.8008649 | 8.002066 | 135-137 |
| TCCGC | 11306930 | 1.7996824 | 7.434523 | 100-104 |
| AAACG | 5827445 | 1.7988664 | 12.501557 | 90-94 |
| AAGAG | 6393015 | 1.7982066 | 14.617323 | 25-29 |
| GATCT | 6265120 | 1.7785562 | 11.723046 | 115-119 |
| CTCCC | 10181625 | 1.7785051 | 20.80795 | 6 |
| CGCCA | 10695530 | 1.7751896 | 8.121529 | 105-109 |
| CAGGC | 11697865 | 1.7691396 | 5.958484 | 3 |
| ATATT | 3176005 | 1.7647911 | 6.2169414 | 70-74 |
| TAACG | 5932055 | 1.7560409 | 12.846012 | 45-49 |
| GTATG | 6787940 | 1.7558577 | 10.71725 | 30-34 |
| ACGTG | 8806540 | 1.7418418 | 12.303653 | 7 |
| TCTCC | 7558690 | 1.7267618 | 7.4404535 | 50-54 |
| GCAGG | 12489095 | 1.7210741 | 10.10306 | 1 |
| TGTAG | 6609060 | 1.7095863 | 13.474465 | 7 |
| TCAGG | 8636970 | 1.7083027 | 10.271593 | 65-69 |
| ACCGC | 10271055 | 1.7047373 | 5.7978363 | 75-79 |
| GGCGA | 12243105 | 1.687175 | 5.1541877 | 1 |
| AGGGT | 9341190 | 1.6835216 | 6.57497 | 80-84 |
| CAACG | 7413345 | 1.6780148 | 6.793072 | 125-129 |
| CGCCG | 15124030 | 1.6771964 | 13.301316 | 9 |
| AACGC | 7409515 | 1.6771479 | 6.5525713 | 95-99 |
| TCTTG | 6140580 | 1.6716923 | 7.285066 | 120-124 |
| ATTCA | 3931700 | 1.6704929 | 5.692016 | 5 |
| AGGTA | 6187145 | 1.6689097 | 8.870696 | 75-79 |
| GGTAA | 6133370 | 1.6544044 | 14.990586 | 95-99 |
| GGTAT | 6371430 | 1.6481175 | 8.206442 | 30-34 |
| CACGC | 9924815 | 1.6472702 | 9.255783 | 105-109 |
| CATCT | 5268560 | 1.6414098 | 5.685368 | 110-114 |
| CGAGG | 11904425 | 1.640503 | 5.097043 | 130-134 |
| AGTTC | 5751695 | 1.632804 | 9.480671 | 25-29 |
| GACCC | 9773505 | 1.6221563 | 9.478712 | 15-19 |
| GAGGT | 8976980 | 1.6178817 | 6.211363 | 75-79 |
| TGTTA | 4330150 | 1.6076409 | 7.858645 | 105-109 |
| CCAAC | 6464245 | 1.6057808 | 6.409968 | 85-89 |
| CCACG | 9651770 | 1.6019515 | 10.936676 | 8 |
| CCCTC | 9105920 | 1.5906032 | 5.320377 | 35-39 |
| TCCCG | 9985925 | 1.5894228 | 14.79498 | 7 |
| ATCCG | 7296660 | 1.5838513 | 5.948668 | 100-104 |
| CCCGC | 13007235 | 1.5830266 | 7.1383452 | 9 |
| GACGT | 8001135 | 1.5825411 | 12.213124 | 6 |
| AGAGT | 5863265 | 1.581547 | 14.731551 | 25-29 |
| GGAAG | 8311360 | 1.5619957 | 12.807492 | 2 |
| AAGTT | 4030280 | 1.5603161 | 7.303324 | 5 |
| CTGTT | 5731280 | 1.5602657 | 5.5270615 | 135-137 |
| AACGT | 5257875 | 1.5564665 | 5.649554 | 85-89 |
| GGTGA | 8569055 | 1.5443631 | 6.743432 | 75-79 |
| AAATC | 3457320 | 1.5317754 | 9.369036 | 125-129 |
| CCTGG | 10472360 | 1.5188289 | 6.7771325 | 20-24 |
| TTGGC | 7773445 | 1.4744353 | 9.810366 | 70-74 |
| CATGC | 6762610 | 1.4679276 | 9.670936 | 90-94 |
| GAGGG | 11689025 | 1.4677767 | 7.6891627 | 10-14 |
| GTAGC | 7359100 | 1.4555533 | 10.065394 | 8 |
| CCGTA | 6674935 | 1.4488964 | 6.76636 | 60-64 |
| TCTGG | 7630410 | 1.4473048 | 7.5063477 | 110-114 |
| CCGGA | 9563510 | 1.4463482 | 12.604611 | 9 |
| GCATG | 7293910 | 1.4426594 | 6.712519 | 85-89 |
| AAGGG | 7653210 | 1.4383065 | 8.059092 | 5 |
| CCGCC | 11774455 | 1.4329932 | 5.0940957 | 55-59 |
| AATGG | 5295775 | 1.428473 | 6.3726835 | 135-137 |
| TGCCC | 8971740 | 1.4279988 | 8.789289 | 90-94 |
| GGAGA | 7598215 | 1.4279709 | 22.329521 | 4 |
| CTTCC | 6237460 | 1.4249305 | 5.869928 | 60-64 |
| TAATA | 2457060 | 1.4237022 | 7.593019 | 70-74 |
| GGCGT | 10758220 | 1.4217321 | 5.350023 | 1 |
| AGGCA | 6892305 | 1.4215404 | 5.987906 | 15-19 |
| ATGCC | 6490565 | 1.4088761 | 11.187744 | 90-94 |
| GCGTC | 9683600 | 1.4044333 | 6.9549665 | 120-124 |
| TTCAG | 4941795 | 1.402888 | 5.545598 | 2 |
| CGCAT | 6458720 | 1.4019637 | 5.534261 | 65-69 |
| ACCAC | 5634405 | 1.3996406 | 16.546644 | 7 |
| ACGCC | 8424320 | 1.3982255 | 19.39273 | 8 |
| GCAAG | 6755140 | 1.3932499 | 5.9550385 | 115-119 |
| TGGCT | 7344645 | 1.393102 | 10.753465 | 70-74 |
| GCGTT | 7337000 | 1.391652 | 7.1561255 | 125-129 |
| CTTGT | 5082545 | 1.3836561 | 9.112461 | 120-124 |
| TTAAT | 2490030 | 1.3836197 | 7.387671 | 70-74 |
| GTTCA | 4868255 | 1.382011 | 6.501117 | 1 |
| TCCGG | 9528490 | 1.3819374 | 7.3998914 | 120-124 |
| GAGTT | 5339935 | 1.3812976 | 8.819491 | 25-29 |
| ACACC | 5558135 | 1.3806943 | 17.711624 | 9 |
| TCGGA | 6962575 | 1.3771249 | 27.27445 | 2 |
| TACGG | 6929090 | 1.3705018 | 12.460867 | 135-137 |
| GGCCT | 9439530 | 1.3690352 | 5.456532 | 70-74 |
| GGTGG | 11356230 | 1.3674915 | 8.532316 | 115-119 |
| GTGCC | 9412025 | 1.3650461 | 9.280451 | 9 |
| GAAGG | 7256410 | 1.3637339 | 8.198335 | 4 |
| AACGG | 6571535 | 1.3553814 | 10.463984 | 45-49 |
| GTGCA | 6840245 | 1.3529292 | 14.16334 | 5 |
| CTCCG | 8409890 | 1.3385711 | 6.5541644 | 50-54 |
| GGTAG | 7371440 | 1.3285222 | 8.320602 | 80-84 |
| GGGAA | 7067190 | 1.3281727 | 12.716696 | 1 |
| AGACG | 6436750 | 1.327582 | 23.85966 | 6 |
| GTGAC | 6701790 | 1.3255442 | 8.165995 | 75-79 |
| GCACA | 5836375 | 1.3210666 | 16.292843 | 7 |
| ATTTT | 2464235 | 1.3131157 | 5.6472483 | 135-137 |
| CGTCC | 8140345 | 1.2956687 | 8.902981 | 120-124 |
| CTGGT | 6815375 | 1.2927123 | 6.5054326 | 110-114 |
| GTCGC | 8875200 | 1.2871892 | 7.591813 | 8 |
| CGACT | 5908840 | 1.2826036 | 5.282145 | 130-134 |
| TGGGA | 7091560 | 1.2780808 | 10.057987 | 20-24 |
| AGGAC | 6175080 | 1.2736124 | 5.3060594 | 25-29 |
| TAGAC | 4278465 | 1.2665359 | 7.603312 | 125-129 |
| CACCC | 6913690 | 1.2593291 | 9.381631 | 55-59 |
| CCCGG | 11334010 | 1.2568978 | 9.93697 | 8 |
| TCCGT | 6022010 | 1.2535467 | 5.7177596 | 50-54 |
| TAGGG | 6925025 | 1.2480668 | 9.876389 | 80-84 |
| GTAGA | 4595700 | 1.2396361 | 5.8982997 | 120-124 |
| TGTCG | 6483830 | 1.2298262 | 5.0728164 | 30-34 |
| TATAG | 3171065 | 1.2276723 | 14.159753 | 7 |
| ATGTA | 3168905 | 1.2268362 | 17.12146 | 6 |
| ATATG | 3143060 | 1.2168303 | 16.573235 | 4 |
| ACATT | 2813335 | 1.1953242 | 10.640665 | 2 |
| TGCAC | 5503055 | 1.194522 | 15.321914 | 6 |
| GTTGG | 6871340 | 1.1875905 | 7.677492 | 70-74 |
| TAGGC | 5974220 | 1.1816385 | 5.1948256 | 60-64 |
| GTTTC | 4312860 | 1.1741195 | 18.011204 | 1 |
| GGGAC | 8431140 | 1.161863 | 6.369722 | 15-19 |
| GAGGC | 8419015 | 1.1601921 | 5.849611 | 9 |
| GACTA | 3915180 | 1.1589941 | 8.149651 | 115-119 |
| GTATA | 2976750 | 1.1524438 | 13.996587 | 6 |
| TCGGT | 6068255 | 1.1510017 | 5.090332 | 70-74 |
| GTTAC | 3992215 | 1.1333189 | 5.9643626 | 110-114 |
| ACTTG | 3979720 | 1.1297717 | 7.936626 | 130-134 |
| ACGGC | 7449430 | 1.1266228 | 6.9995165 | 135-137 |
| TTACG | 3929120 | 1.1154073 | 6.3132987 | 110-114 |
| GAAGT | 4102125 | 1.1065001 | 6.295895 | 4 |
| CACAT | 3381665 | 1.0986186 | 8.627719 | 1 |
| CTACG | 5049610 | 1.0960948 | 11.738592 | 135-137 |
| ACGAC | 4831130 | 1.093529 | 5.0163445 | 130-134 |
| CTCGG | 7428210 | 1.0773293 | 17.84161 | 1 |
| CACGA | 4756965 | 1.0767417 | 14.618823 | 9 |
| GCCAC | 6424705 | 1.0663397 | 7.524802 | 105-109 |
| TTTCG | 3877755 | 1.0556679 | 16.973621 | 2 |
| ATCTT | 2578265 | 1.0505108 | 5.4774957 | 30-34 |
| TGCGT | 5460550 | 1.0357347 | 7.04928 | 125-129 |
| GTCCG | 7117030 | 1.0321982 | 8.457991 | 120-124 |
| TAAAT | 1770535 | 1.0259068 | 5.417308 | 3 |
| AGCTC | 4705570 | 1.0214157 | 6.804236 | 4 |
| AGGTG | 5599600 | 1.0091913 | 12.831956 | 3 |
| AAGCT | 3372660 | 0.99839425 | 9.161164 | 3 |
| GAGCG | 7133970 | 0.98310494 | 5.4830685 | 135-137 |
| TCCCT | 4287025 | 0.97935903 | 7.0144525 | 7 |
| GTGCG | 7385825 | 0.9760596 | 8.638717 | 125-129 |
| ATAGC | 3289035 | 0.973639 | 11.342136 | 8 |
| AGTTA | 2502475 | 0.968829 | 6.6462355 | 6 |
| AATTG | 2481370 | 0.96065813 | 7.022716 | 5 |
| AGGCC | 6301520 | 0.95301735 | 5.078567 | 135-137 |
| GGTTT | 3834700 | 0.9512428 | 10.6388 | 3 |
| CATAT | 2208110 | 0.9381774 | 18.286764 | 3 |
| GGACC | 6111695 | 0.92430896 | 7.836971 | 15-19 |
| CGTGC | 6344460 | 0.92015064 | 9.150024 | 8 |
| ACTTT | 2257570 | 0.919844 | 7.029736 | 7 |
| GGGAG | 7308150 | 0.91767555 | 5.2175374 | 15-19 |
| AAGCA | 2934265 | 0.9057744 | 7.952482 | 4 |
| ACGTA | 3043595 | 0.90098244 | 10.575279 | 4 |
| GCCAG | 5864885 | 0.8869824 | 6.346983 | 1 |
| CTGCA | 4041400 | 0.8772475 | 5.1629043 | 3 |
| TCGCC | 5508760 | 0.87680906 | 6.0514092 | 9 |
| GAGAC | 4247635 | 0.8760762 | 24.050953 | 5 |
| TCGCG | 6029215 | 0.87443 | 5.8410964 | 35-39 |
| TACTC | 2751645 | 0.85726964 | 5.6360393 | 120-124 |
| TTTGC | 3133875 | 0.8531564 | 5.2327857 | 9 |
| GCACG | 5571635 | 0.8426323 | 5.1099687 | 15-19 |
| TTCCC | 3585730 | 0.81915015 | 6.0948715 | 6 |
| CGTCG | 5617510 | 0.81471956 | 5.6076713 | 7 |
| CGACG | 5343630 | 0.80814976 | 9.513823 | 5 |
| ATTGT | 2117845 | 0.7862855 | 5.7337437 | 6 |
| GGACG | 5579650 | 0.7689101 | 5.122687 | 2 |
| ACGGG | 5577395 | 0.76859933 | 7.4575553 | 45-49 |
| TTTCC | 2562865 | 0.7657016 | 7.8737473 | 5 |
| AGCAT | 2455510 | 0.72689414 | 12.710303 | 1 |
| GAATT | 1868485 | 0.7233808 | 6.4760523 | 4 |
| CTGGG | 5408530 | 0.714754 | 6.7982383 | 20-24 |
| GTAGT | 2592090 | 0.670504 | 5.2773967 | 125-129 |
| CCTGT | 3209860 | 0.6681672 | 8.955896 | 9 |
| GTCGA | 3319885 | 0.6566387 | 5.798936 | 1 |
| CCAGG | 4305645 | 0.651169 | 5.743209 | 2 |
| GACGC | 3948400 | 0.59714067 | 17.590347 | 7 |
| CCCCG | 4320540 | 0.525825 | 6.1852536 | 8 |
| GCATA | 1753330 | 0.5190308 | 12.473152 | 2 |

Produced by FastQC (version 0.10.1)
