# Supplementary material for: Freshwater sponge hosts and their green algae symbionts: a tractable model to understand intracellular symbiosis
Source: PeerJ. 2021 Feb 11;9:e10654. doi: 10.7717/peerj.10654 (PMC7882143; doi:10.7717/peerj.10654)
Supplement: Supplemental Information 32 [file peerj-09-10654-s032.zip › EmInf2_Clean_Data1.fq_fastqc/fastqc_report.html]

EmInf2\_Clean\_Data1.fq.gz FastQC Report


FastQC Report

Tue 10 Sep 2019  
EmInf2\_Clean\_Data1.fq.gz

## Summary

- Basic Statistics
- Per base sequence quality
- Per sequence quality scores
- Per base sequence content
- Per base GC content
- Per sequence GC content
- Per base N content
- Sequence Length Distribution
- Sequence Duplication Levels
- Overrepresented sequences
- Kmer Content

## Basic Statistics

| Measure | Value |
| --- | --- |
| Filename | EmInf2\_Clean\_Data1.fq.gz |
| File type | Conventional base calls |
| Encoding | Sanger / Illumina 1.9 |
| Total Sequences | 28504992 |
| Filtered Sequences | 0 |
| Sequence length | 100-141 |
| %GC | 57 |

## Per base sequence quality

## Per sequence quality scores

## Per base sequence content

## Per base GC content

## Per sequence GC content

## Per base N content

## Sequence Length Distribution

## Sequence Duplication Levels

## Overrepresented sequences

| Sequence | Count | Percentage | Possible Source |
| --- | --- | --- | --- |
| GTCCCATTCAAGTCGTCTACAAGAGATCTTGCCCCGCGGATTGGCCAGCG | 1243815 | 4.363498856621324 | No Hit |
| GCGAGAAAATGAACCGCTCCCTCGGATTTTCAAGGGCCGTAGAGAACGCA | 973363 | 3.414710658399764 | No Hit |
| AGAAAATGAACCGCTCCCTCGGATTTTCAAGGGCCGTAGAGAACGCACCG | 870662 | 3.0544193802966157 | No Hit |
| GAGAAAATGAACCGCTCCCTCGGATTTTCAAGGGCCGTAGAGAACGCACC | 703781 | 2.468974557158269 | No Hit |
| GCCACCTACAGCCAACAGTCTGAAGCGCAGTCGCGAACCCCGCGCACGGC | 463464 | 1.6259046836427808 | No Hit |
| GTCGTCTACAAGAGATCTTGCCCCGCGGATTGGCCAGCGTTTGATACGCG | 405354 | 1.4220456543190751 | No Hit |
| GCCGTTAGTCGCCTGCCGAATAGCCGCCGACCACGAGGGACGGCGACCAA | 374308 | 1.3131313981775543 | No Hit |
| AAGAGATCTTGCCCCGCGGATTGGCCAGCGTTTGATACGCGCGGTCACCG | 340776 | 1.1954958626194316 | No Hit |
| GGCGAGAAAATGAACCGCTCCCTCGGATTTTCAAGGGCCGTAGAGAACGC | 339719 | 1.191787740196524 | No Hit |
| GAAAATGAACCGCTCCCTCGGATTTTCAAGGGCCGTAGAGAACGCACCGG | 338886 | 1.1888654450420473 | No Hit |
| CTGCGCTGGCGGGTCGAAGAGACCCTCTCCTCGGTCGCGGGCGCGCTCCG | 327132 | 1.1476305623941239 | No Hit |
| ATTCAAGTCGTCTACAAGAGATCTTGCCCCGCGGATTGGCCAGCGTTTGA | 268255 | 0.9410807763075324 | No Hit |
| CGGGCGAGAAAATGAACCGCTCCCTCGGATTTTCAAGGGCCGTAGAGAAC | 243572 | 0.8544889260098723 | No Hit |
| AGCGCAGTCGCGAACCCCGCGCACGGCGGAGGGATGCGCCGGCCTCGCAC | 235205 | 0.8251361726395153 | No Hit |
| GATGAAGCCACCTACAGCCAACAGTCTGAAGCGCAGTCGCGAACCCCGCG | 184911 | 0.6486969019321247 | No Hit |
| CTCTCCTCGGTCGCGGGCGCGCTCCGAACGACGCGGCTATACGTCCCTAA | 180801 | 0.6342783748194001 | No Hit |
| GGCCGTTAGTCGCCTGCCGAATAGCCGCCGACCACGAGGGACGGCGACCA | 178237 | 0.6252834591218268 | No Hit |
| GAGATCTTGCCCCGCGGATTGGCCAGCGTTTGATACGCGCGGTCACCGAA | 172766 | 0.6060903297218957 | No Hit |
| AGAGATCTTGCCCCGCGGATTGGCCAGCGTTTGATACGCGCGGTCACCGA | 167441 | 0.5874093913094239 | No Hit |
| CTCCTCGGTCGCGGGCGCGCTCCGAACGACGCGGCTATACGTCCCTAACT | 166565 | 0.584336245384668 | No Hit |
| GTCGCCGTAACAGCACCGCCCGCAACCCACGTTGGCCAGCCCCGGTGAGA | 164694 | 0.5777724827988024 | No Hit |
| GCGCTGGCGGGTCGAAGAGACCCTCTCCTCGGTCGCGGGCGCGCTCCGAA | 156531 | 0.5491353935479091 | No Hit |
| GTCTACAAGAGATCTTGCCCCGCGGATTGGCCAGCGTTTGATACGCGCGG | 135835 | 0.4765305669968264 | No Hit |
| AAAATGAACCGCTCCCTCGGATTTTCAAGGGCCGTAGAGAACGCACCGGA | 126958 | 0.44538865332781014 | No Hit |
| CGAGAAAATGAACCGCTCCCTCGGATTTTCAAGGGCCGTAGAGAACGCAC | 119590 | 0.4195405492483562 | No Hit |
| GTCAGATGAAGCCACCTACAGCCAACAGTCTGAAGCGCAGTCGCGAACCC | 117530 | 0.41231374490475214 | No Hit |
| AGAAATTTGAATGCACCATCGCCGGCACGAGGCCATGCGATTCGAGCAGT | 116755 | 0.4095949228822797 | No Hit |
| GGGCGAGAAAATGAACCGCTCCCTCGGATTTTCAAGGGCCGTAGAGAACG | 112611 | 0.3950571184163111 | No Hit |
| CAAGTCGTCTACAAGAGATCTTGCCCCGCGGATTGGCCAGCGTTTGATAC | 109831 | 0.3853044407098939 | No Hit |
| GAAGCCACCTACAGCCAACAGTCTGAAGCGCAGTCGCGAACCCCGCGCAC | 108815 | 0.3817401527423688 | No Hit |
| GCAGAAATTTGAATGCACCATCGCCGGCACGAGGCCATGCGATTCGAGCA | 105716 | 0.37086837280992746 | No Hit |
| GCCCGCAACCCACGTTGGCCAGCCCCGGTGAGAAATGCGGAAGCGGCGGT | 102247 | 0.3586985746215961 | No Hit |
| GTCGGCCGTTAGTCGCCTGCCGAATAGCCGCCGACCACGAGGGACGGCGA | 101550 | 0.3562533888800951 | No Hit |
| CCGCCCGCAACCCACGTTGGCCAGCCCCGGTGAGAAATGCGGAAGCGGCG | 91571 | 0.3212454857029955 | No Hit |
| CCGCAACCCACGTTGGCCAGCCCCGGTGAGAAATGCGGAAGCGGCGGTCG | 89644 | 0.3144852663000221 | No Hit |
| AGATCTTGCCCCGCGGATTGGCCAGCGTTTGATACGCGCGGTCACCGAAG | 88116 | 0.309124801718941 | No Hit |
| CTCGTCCCATTCAAGTCGTCTACAAGAGATCTTGCCCCGCGGATTGGCCA | 86437 | 0.3032346053631588 | No Hit |
| GAAATTTGAATGCACCATCGCCGGCACGAGGCCATGCGATTCGAGCAGTT | 85367 | 0.29948087689342273 | No Hit |
| CGCGGATTGGCCAGCGTTTGATACGCGCGGTCACCGAAGGCCGCCTACGG | 85076 | 0.2984600030759525 | No Hit |
| CCCGCAACCCACGTTGGCCAGCCCCGGTGAGAAATGCGGAAGCGGCGGTC | 80585 | 0.28270486797540584 | No Hit |
| AAATGAACCGCTCCCTCGGATTTTCAAGGGCCGTAGAGAACGCACCGGAC | 79397 | 0.2785371769267643 | No Hit |
| TTCAAGTCGTCTACAAGAGATCTTGCCCCGCGGATTGGCCAGCGTTTGAT | 79105 | 0.2775127949518456 | No Hit |
| CATTCAAGTCGTCTACAAGAGATCTTGCCCCGCGGATTGGCCAGCGTTTG | 78643 | 0.27589202621070724 | No Hit |
| CAAGAGATCTTGCCCCGCGGATTGGCCAGCGTTTGATACGCGCGGTCACC | 78415 | 0.2750921663124831 | No Hit |
| GCCGTAACAGCACCGCCCGCAACCCACGTTGGCCAGCCCCGGTGAGAAAT | 78116 | 0.274043227235426 | No Hit |
| GTTAGTCGCCTGCCGAATAGCCGCCGACCACGAGGGACGGCGACCAAGCT | 77159 | 0.2706859205573536 | No Hit |
| GCCTGCGCTGGCGGGTCGAAGAGACCCTCTCCTCGGTCGCGGGCGCGCTC | 76016 | 0.2666760965938878 | No Hit |
| GATCTTGCCCCGCGGATTGGCCAGCGTTTGATACGCGCGGTCACCGAAGG | 74104 | 0.25996849955263973 | No Hit |
| CTTATATTGGTCGGGCTAGGAGCTGAGTCTACTCACAGGCACTATCCCAT | 73954 | 0.25944227593538705 | No Hit |
| GCGGGAGCTCCGGCCACGAAGGCCTGCGCTGGCGGGTCGAAGAGACCCTC | 73882 | 0.25918968859910574 | No Hit |
| CCTGACTCTCCAAAGACACCTAATATCTAGGCAGGCGGTCGGCCGCGTAC | 73408 | 0.2575268219685871 | No Hit |
| GCGCAGTCGCGAACCCCGCGCACGGCGGAGGGATGCGCCGGCCTCGCACT | 73131 | 0.2565550623553937 | No Hit |
| CCCATTCAAGTCGTCTACAAGAGATCTTGCCCCGCGGATTGGCCAGCGTT | 68066 | 0.2387862448794934 | No Hit |
| CGTTAGTCGCCTGCCGAATAGCCGCCGACCACGAGGGACGGCGACCAAGC | 64574 | 0.2265357590698499 | No Hit |
| CCTGCGCTGGCGGGTCGAAGAGACCCTCTCCTCGGTCGCGGGCGCGCTCC | 60770 | 0.2131907281363208 | No Hit |
| CGAGATGGCGCCCTCCACCGGAACGCGGGAGCTCCGGCCACGAAGGCCTG | 59646 | 0.20924755916437374 | No Hit |
| GGCGGGTCGAAGAGACCCTCTCCTCGGTCGCGGGCGCGCTCCGAACGACG | 57670 | 0.20231544004643115 | No Hit |
| CACCTACAGCCAACAGTCTGAAGCGCAGTCGCGAACCCCGCGCACGGCGG | 56429 | 0.19796181665302695 | No Hit |
| GCCAACAGTCTGAAGCGCAGTCGCGAACCCCGCGCACGGCGGAGGGATGC | 55739 | 0.19554118801366444 | No Hit |
| AGATGAAGCCACCTACAGCCAACAGTCTGAAGCGCAGTCGCGAACCCCGC | 53104 | 0.1862971931372582 | No Hit |
| GGCCTGCGCTGGCGGGTCGAAGAGACCCTCTCCTCGGTCGCGGGCGCGCT | 52996 | 0.18591831213283624 | No Hit |
| GGGAAATGTGTCGTTGCGTTCTAGCGTGGATTCTGACTTAGAGGCGTTCA | 52604 | 0.18454311441308244 | No Hit |
| CTGCTTACAACACCTCGTCCCATTCAAGTCGTCTACAAGAGATCTTGCCC | 51564 | 0.1808946306667969 | No Hit |
| GCCACGAAGGCCTGCGCTGGCGGGTCGAAGAGACCCTCTCCTCGGTCGCG | 50207 | 0.17613406100938392 | No Hit |
| CTCCAAAGACACCTAATATCTAGGCAGGCGGTCGGCCGCGTACGGGGTTC | 48211 | 0.1691317787424743 | No Hit |
| TGACTCTCCAAAGACACCTAATATCTAGGCAGGCGGTCGGCCGCGTACGG | 47239 | 0.16572184970267664 | No Hit |
| CTACAAGAGATCTTGCCCCGCGGATTGGCCAGCGTTTGATACGCGCGGTC | 47234 | 0.1657043089154349 | No Hit |
| CTCCACCGGAACGCGGGAGCTCCGGCCACGAAGGCCTGCGCTGGCGGGTC | 47082 | 0.16517106898328546 | No Hit |
| CTCGGTCGCGGGCGCGCTCCGAACGACGCGGCTATACGTCCCTAACTTCG | 46314 | 0.16247680406295148 | No Hit |
| CACCGGAACGCGGGAGCTCCGGCCACGAAGGCCTGCGCTGGCGGGTCGAA | 45637 | 0.16010178147041754 | No Hit |
| CTCGTCCCGGTTCGGGAATATTAACCCGATTCCCTTTCGATGGTGGGTGC | 43471 | 0.15250311243728817 | No Hit |
| AAGCGCAGTCGCGAACCCCGCGCACGGCGGAGGGATGCGCCGGCCTCGCA | 42779 | 0.15007546748302894 | No Hit |
| GCTTACAACACCTCGTCCCATTCAAGTCGTCTACAAGAGATCTTGCCCCG | 42636 | 0.14957380096791467 | No Hit |
| CCGAGATGGCGCCCTCCACCGGAACGCGGGAGCTCCGGCCACGAAGGCCT | 41885 | 0.1469391747242027 | No Hit |
| GGCAGAAATTTGAATGCACCATCGCCGGCACGAGGCCATGCGATTCGAGC | 41422 | 0.14531489782561594 | No Hit |
| GCCCACTGGTGTTAGTTTTAGTACAGCCGAGCCCAATTTATTGGGCTGAA | 39623 | 0.1390037225760316 | No Hit |
| AAGCCACCTACAGCCAACAGTCTGAAGCGCAGTCGCGAACCCCGCGCACG | 39570 | 0.13881779023126897 | No Hit |
| CTACAGCCAACAGTCTGAAGCGCAGTCGCGAACCCCGCGCACGGCGGAGG | 38714 | 0.13581480745548008 | No Hit |
| GCGGATATGAGTACGACCGGGCGAGAAAATGAACCGCTCCCTCGGATTTT | 38662 | 0.1356323832681658 | No Hit |
| GTCCCGGTTCGGGAATATTAACCCGATTCCCTTTCGATGGTGGGTGCCGG | 38381 | 0.134646591025179 | No Hit |
| ACCGCCCGCAACCCACGTTGGCCAGCCCCGGTGAGAAATGCGGAAGCGGC | 37855 | 0.13280130020734615 | No Hit |
| GCTCCCTCGGATTTTCAAGGGCCGTAGAGAACGCACCGGACGCCACCAGA | 37647 | 0.13207160345808902 | No Hit |
| CAGATGAAGCCACCTACAGCCAACAGTCTGAAGCGCAGTCGCGAACCCCG | 37233 | 0.1306192262744715 | No Hit |
| CGCCGTAACAGCACCGCCCGCAACCCACGTTGGCCAGCCCCGGTGAGAAA | 37161 | 0.1303666389381902 | No Hit |
| ATCTTGCCCCGCGGATTGGCCAGCGTTTGATACGCGCGGTCACCGAAGGC | 37152 | 0.13033506552115504 | No Hit |
| GCGGGGAAATGTGTCGTTGCGTTCTAGCGTGGATTCTGACTTAGAGGCGT | 36761 | 0.1289633759588496 | No Hit |
| GCCAGCGTTTGATACGCGCGGTCACCGAAGGCCGCCTACGGGCCACGGAG | 36382 | 0.12763378428592437 | No Hit |
| GTCGGGCTAGGAGCTGAGTCTACTCACAGGCACTATCCCATTACCGCCTG | 36043 | 0.12644451891093322 | No Hit |
| CTACTGCTTACAACACCTCGTCCCATTCAAGTCGTCTACAAGAGATCTTG | 35510 | 0.12457467099096185 | No Hit |
| CTCCAGCCAACCTGATTCCAGGGTGATGGCCCGTTAAGAAGAAAAGAGAA | 34855 | 0.12227682786229163 | No Hit |
| GGTCGGGCTAGGAGCTGAGTCTACTCACAGGCACTATCCCATTACCGCCT | 34328 | 0.12042802888701039 | No Hit |
| CCATTCAAGTCGTCTACAAGAGATCTTGCCCCGCGGATTGGCCAGCGTTT | 34192 | 0.11995091947403459 | No Hit |
| CCTCGGTCGCGGGCGCGCTCCGAACGACGCGGCTATACGTCCCTAACTTC | 34173 | 0.1198842644825159 | No Hit |
| GCGCATATGTAGCCCAAAACATTAGGATCATAAGGACCTGACGTCATCCT | 34123 | 0.11970885661009834 | No Hit |
| CAACAGTCTGAAGCGCAGTCGCGAACCCCGCGCACGGCGGAGGGATGCGC | 32629 | 0.11446766938226119 | No Hit |
| CCGGGCGAGAAAATGAACCGCTCCCTCGGATTTTCAAGGGCCGTAGAGAA | 32561 | 0.11422911467577329 | No Hit |
| CTCGGATTTTCAAGGGCCGTAGAGAACGCACCGGACGCCACCAGAAGCGT | 32503 | 0.11402564154376889 | No Hit |
| CGCCCGCAACCCACGTTGGCCAGCCCCGGTGAGAAATGCGGAAGCGGCGG | 32294 | 0.11329243663706344 | No Hit |
| CCGTTAGTCGCCTGCCGAATAGCCGCCGACCACGAGGGACGGCGACCAAG | 31748 | 0.11137698267026351 | No Hit |
| TGAAGCCACCTACAGCCAACAGTCTGAAGCGCAGTCGCGAACCCCGCGCA | 31380 | 0.11008598072927014 | No Hit |
| ACAAGAGATCTTGCCCCGCGGATTGGCCAGCGTTTGATACGCGCGGTCAC | 30328 | 0.10639539909360438 | No Hit |
| GTCGCCTGCCGAATAGCCGCCGACCACGAGGGACGGCGACCAAGCTGCGG | 30070 | 0.10549029447192969 | No Hit |
| GCACCGCCCGCAACCCACGTTGGCCAGCCCCGGTGAGAAATGCGGAAGCG | 30035 | 0.1053675089612374 | No Hit |
| CTCTACTGCTTACAACACCTCGTCCCATTCAAGTCGTCTACAAGAGATCT | 29562 | 0.10370815048816712 | No Hit |
| GGCGCTTTACCGGGCATGCAACCCTACCTCCAGCCAACCTGATTCCAGGG | 29491 | 0.10345907130933417 | No Hit |
| CACCCGGTCGCCGTAACAGCACCGCCCGCAACCCACGTTGGCCAGCCCCG | 29097 | 0.10207685727468367 | No Hit |
| ATGAAGCCACCTACAGCCAACAGTCTGAAGCGCAGTCGCGAACCCCGCGC | 28803 | 0.10104545898486834 | No Hit |

## Kmer Content

| Sequence | Count | Obs/Exp Overall | Obs/Exp Max | Max Obs/Exp Position |
| --- | --- | --- | --- | --- |
| ATTTT | 5985725 | 4.719981 | 47.343998 | 20-24 |
| TTCAA | 9767715 | 4.3678203 | 84.5897 | 7 |
| GATTC | 11579325 | 4.189645 | 23.090807 | 105-109 |
| AGAGA | 13687575 | 4.1671968 | 20.52204 | 35-39 |
| GAGAT | 11987960 | 4.1648593 | 19.196075 | 3 |
| AGAAA | 11064300 | 4.1631236 | 53.298157 | 4 |
| GGATT | 10283315 | 4.0768595 | 36.135597 | 20-24 |
| AAGAA | 10077890 | 3.7919703 | 59.547756 | 130-134 |
| TTGAT | 6741790 | 3.7694955 | 23.395737 | 50-54 |
| TTTTC | 6432495 | 3.745643 | 36.9494 | 25-29 |
| TTTCA | 7189135 | 3.6684787 | 43.44578 | 25-29 |
| TTTGA | 6524035 | 3.647744 | 23.461187 | 50-54 |
| AAATG | 8494060 | 3.6471047 | 60.31728 | 7 |
| GAGAA | 11376115 | 3.4634702 | 41.707123 | 3 |
| TTCGC | 12892115 | 3.4446218 | 15.302352 | 95-99 |
| TGATT | 5911720 | 3.3053837 | 34.29908 | 105-109 |
| ATCTT | 6476790 | 3.3049827 | 27.27301 | 6 |
| GATTT | 5862005 | 3.2775874 | 34.07936 | 20-24 |
| AGAAG | 10748805 | 3.272485 | 28.614834 | 130-134 |
| CTTCG | 11875835 | 3.1730838 | 12.142081 | 100-104 |
| ACCGG | 18550050 | 3.0797071 | 18.056149 | 75-79 |
| AAAAT | 5785025 | 3.0698416 | 71.556725 | 6 |
| TTTAC | 5964850 | 3.0437493 | 31.18367 | 70-74 |
| GAAAA | 8039995 | 3.0251796 | 50.815144 | 5 |
| TCCCT | 12255365 | 2.988443 | 20.935427 | 15-19 |
| TCCAG | 12739065 | 2.9827533 | 20.627888 | 110-114 |
| ATGAA | 6943260 | 2.9812362 | 59.976166 | 6 |
| CTTTA | 5782765 | 2.9508352 | 30.9548 | 70-74 |
| TGATG | 7429200 | 2.9453347 | 24.770874 | 115-119 |
| ATTGG | 7424535 | 2.9434857 | 16.824379 | 40-44 |
| GATGG | 10443750 | 2.9358487 | 19.611227 | 120-124 |
| CGCTT | 10978030 | 2.9332008 | 22.748291 | 70-74 |
| TTGCC | 10889485 | 2.9095426 | 13.05265 | 9 |
| TGATA | 5912805 | 2.8971038 | 20.59517 | 50-54 |
| GCTTT | 6953730 | 2.8711069 | 35.061363 | 70-74 |
| GCGCT | 14979285 | 2.8378732 | 12.995385 | 70-74 |
| AGATT | 5698885 | 2.7922893 | 20.251179 | 90-94 |
| TACCG | 11925230 | 2.7922006 | 22.385622 | 75-79 |
| TCGCG | 14386875 | 2.7256393 | 10.862926 | 95-99 |
| CGAAG | 12048825 | 2.708854 | 11.008896 | 65-69 |
| TTGGC | 9241415 | 2.7055433 | 12.976473 | 40-44 |
| TCAAG | 8530810 | 2.7048717 | 59.359097 | 8 |
| AGTTG | 6772420 | 2.684952 | 15.854592 | 115-119 |
| TTAAG | 5479795 | 2.6849413 | 31.119778 | 125-129 |
| GTTTG | 5867875 | 2.6546729 | 18.238691 | 50-54 |
| CAACC | 14082090 | 2.637003 | 16.047106 | 85-89 |
| GGCCA | 15802865 | 2.6236153 | 7.7968907 | 80-84 |
| TCTTG | 6347825 | 2.6209364 | 21.354258 | 7 |
| AGCGT | 10190210 | 2.6143372 | 16.244608 | 60-64 |
| GAGTT | 6594105 | 2.614258 | 15.3558445 | 115-119 |
| AAGAG | 8562255 | 2.6067874 | 14.561324 | 1 |
| GTTAA | 5299500 | 2.596602 | 31.48874 | 125-129 |
| GAAGA | 8518245 | 2.5933886 | 24.402859 | 135-137 |
| AATGA | 6025735 | 2.587277 | 57.906116 | 8 |
| TGTGT | 5706050 | 2.5814621 | 5.756822 | 7 |
| TTCCA | 7815365 | 2.5807414 | 28.947609 | 110-114 |
| ATACG | 7990845 | 2.5336645 | 13.558052 | 55-59 |
| CGGAT | 9746905 | 2.5006056 | 23.155842 | 20-24 |
| GTGTG | 7682275 | 2.4643624 | 14.393502 | 135-137 |
| ACATA | 6245225 | 2.4472723 | 16.648092 | 105-109 |
| ATTCC | 7360185 | 2.430435 | 21.791023 | 110-114 |
| CCTAC | 11263255 | 2.406828 | 18.605461 | 90-94 |
| CTCCA | 11231595 | 2.4000626 | 21.834335 | 95-99 |
| GTGAT | 6022265 | 2.3875499 | 24.724367 | 115-119 |
| CGCGG | 17716385 | 2.3799157 | 9.625621 | 35-39 |
| TGGCC | 12515360 | 2.371075 | 17.017488 | 120-124 |
| GAACG | 10453025 | 2.3500812 | 19.355433 | 40-44 |
| GAAGG | 9529770 | 2.3475888 | 10.473606 | 70-74 |
| CGGTC | 12361205 | 2.3418696 | 10.270765 | 60-64 |
| GCTCC | 13454585 | 2.3263416 | 10.895631 | 10-14 |
| GGTGA | 8270485 | 2.324921 | 24.445656 | 115-119 |
| GATCT | 6375100 | 2.3066459 | 19.166525 | 5 |
| TAAGA | 5345975 | 2.2954075 | 30.071012 | 130-134 |
| CACCG | 15148990 | 2.2953522 | 15.390938 | 45-49 |
| CTGAT | 6287215 | 2.2748473 | 30.775627 | 105-109 |
| CCAGC | 14899200 | 2.2575045 | 9.436419 | 95-99 |
| GCCAC | 14872125 | 2.253402 | 11.678314 | 1 |
| GATTG | 5642910 | 2.2371535 | 17.422155 | 35-39 |
| GGAGT | 7942910 | 2.232836 | 10.931809 | 115-119 |
| AACCT | 7680845 | 2.2226274 | 18.649742 | 100-104 |
| GGTCA | 8637045 | 2.215867 | 11.561221 | 60-64 |
| ATGCA | 6960830 | 2.2070765 | 19.069874 | 80-84 |
| ATGGC | 8591265 | 2.2041218 | 22.998487 | 120-124 |
| CCACG | 14339850 | 2.1727524 | 6.541534 | 85-89 |
| GAAGC | 9663795 | 2.1726444 | 19.567993 | 60-64 |
| CGTTA | 5992865 | 2.1683452 | 32.325485 | 125-129 |
| TGCAA | 6833845 | 2.1668134 | 20.132666 | 85-89 |
| TAGAG | 6217550 | 2.1601026 | 22.122889 | 35-39 |
| TGGCG | 10398805 | 2.158659 | 14.480434 | 65-69 |
| GTTGC | 7334090 | 2.1471493 | 12.135066 | 115-119 |
| GTAGA | 6165750 | 2.1421056 | 29.318838 | 35-39 |
| ATTCG | 5910020 | 2.1383703 | 15.972951 | 90-94 |
| AGAAC | 7668380 | 2.1307006 | 23.727907 | 40-44 |
| CCTCC | 13444075 | 2.121463 | 13.346906 | 95-99 |
| CGTTT | 5127645 | 2.1171396 | 17.847246 | 45-49 |
| GGTCG | 10173785 | 2.1119478 | 8.224746 | 9 |
| CCTCG | 12074940 | 2.087797 | 11.12221 | 15-19 |
| AACCC | 11047990 | 2.0688393 | 11.486277 | 85-89 |
| TGAAC | 6464570 | 2.0497267 | 38.91658 | 7 |
| CCGAA | 9988580 | 2.0494907 | 10.488941 | 65-69 |
| GCTTC | 7616555 | 2.0350542 | 10.971398 | 100-104 |
| CAGAA | 7308480 | 2.0307004 | 17.723381 | 60-64 |
| CACCA | 10837355 | 2.029396 | 16.280394 | 55-59 |
| ACCTG | 8626685 | 2.0198715 | 15.942292 | 105-109 |
| ATTCA | 4512185 | 2.01771 | 82.42307 | 6 |
| GCACC | 13206485 | 2.0010266 | 12.902148 | 45-49 |
| TACCT | 6058265 | 2.000523 | 19.999245 | 90-94 |
| TTACC | 6051435 | 1.9982674 | 21.080048 | 75-79 |
| TTCGT | 4817170 | 1.9889485 | 18.860344 | 125-129 |
| CGAGA | 8794285 | 1.9771583 | 31.647097 | 2 |
| TACAA | 5030980 | 1.9714547 | 24.3188 | 7 |
| AACTT | 4392045 | 1.9639869 | 8.209898 | 70-74 |
| GATAC | 6166680 | 1.9552748 | 14.0310755 | 50-54 |
| CTACA | 6740930 | 1.9506416 | 35.51174 | 6 |
| GACAT | 6109655 | 1.9371938 | 12.966392 | 105-109 |
| AAGCG | 8449260 | 1.8995887 | 14.157387 | 60-64 |
| AGGGT | 6750975 | 1.8977704 | 18.391668 | 115-119 |
| ACCCT | 8821970 | 1.8851534 | 12.752372 | 85-89 |
| TGGTG | 5855145 | 1.8782457 | 9.70012 | 130-134 |
| GGGAG | 9409320 | 1.8755126 | 10.02878 | 115-119 |
| AGGGC | 10295595 | 1.8728997 | 15.367008 | 30-34 |
| GCGTT | 6301135 | 1.8447385 | 13.058203 | 45-49 |
| AAGGC | 8199685 | 1.8434784 | 9.335833 | 70-74 |
| AGATC | 5778130 | 1.8320769 | 15.930181 | 4 |
| TTTTT | 2033845 | 1.8301172 | 5.8543973 | 110-114 |
| GCAAC | 8911540 | 1.8285 | 17.307755 | 85-89 |
| CAGCC | 12066050 | 1.8282298 | 10.252492 | 9 |
| AGCCA | 8895115 | 1.8251299 | 13.164544 | 100-104 |
| GAAAT | 4229525 | 1.8160365 | 8.6682005 | 4 |
| GGGCC | 13453550 | 1.8072715 | 11.441637 | 30-34 |
| CCCTC | 11433150 | 1.8041409 | 13.889163 | 15-19 |
| ACGCG | 10805905 | 1.7940125 | 9.20572 | 55-59 |
| CGTGG | 8638370 | 1.7932153 | 17.560848 | 65-69 |
| AGATG | 5146085 | 1.787854 | 8.823106 | 4 |
| CGCGC | 14508500 | 1.7787311 | 5.3842177 | 40-44 |
| GCCAA | 8665655 | 1.7780485 | 17.602608 | 100-104 |
| CGGGC | 13217495 | 1.7755611 | 12.326481 | 80-84 |
| GCGGA | 9673720 | 1.7597724 | 10.099579 | 35-39 |
| CCGTA | 7505145 | 1.7572718 | 14.699396 | 35-39 |
| GAACC | 8540530 | 1.7523749 | 25.069584 | 8 |
| AGTCG | 6808510 | 1.7467493 | 19.398104 | 6 |
| GCGGG | 11736305 | 1.7274939 | 8.321626 | 9 |
| AAGTC | 5429595 | 1.7215664 | 12.831426 | 10-14 |
| AAGGG | 6968710 | 1.7166903 | 15.69077 | 30-34 |
| CCGGA | 10272040 | 1.7053797 | 9.993705 | 50-54 |
| CATAC | 5891865 | 1.7049452 | 12.5015955 | 105-109 |
| CGGAG | 9313305 | 1.6942084 | 8.794478 | 85-89 |
| CCACC | 12216620 | 1.6893438 | 11.767149 | 55-59 |
| TGAAG | 4837730 | 1.680725 | 9.738024 | 3 |
| CTACC | 7827300 | 1.672604 | 17.844702 | 90-94 |
| TCCTC | 6854035 | 1.6713408 | 6.981909 | 2 |
| GGCCG | 12420515 | 1.6684994 | 8.703077 | 30-34 |
| CCCTA | 7803095 | 1.6674316 | 14.232666 | 90-94 |
| GTCGC | 8771540 | 1.6617962 | 15.686346 | 8 |
| CGCTC | 9590120 | 1.6581631 | 10.96101 | 10-14 |
| ATATT | 2397205 | 1.6564988 | 9.162726 | 4 |
| CCGTT | 6181590 | 1.6516484 | 23.795805 | 125-129 |
| CCATT | 4978500 | 1.6439693 | 59.955513 | 4 |
| CGGGA | 9014850 | 1.6399157 | 10.669318 | 110-114 |
| GCCGT | 8655925 | 1.6398926 | 12.459886 | 1 |
| GCCAG | 9822415 | 1.630732 | 7.6989408 | 40-44 |
| GGAGA | 6596005 | 1.6248773 | 10.136028 | 90-94 |
| AACAG | 5843760 | 1.6237199 | 8.592341 | 9 |
| CCTGA | 6925410 | 1.621531 | 20.451176 | 105-109 |
| GGCAT | 6298545 | 1.6159157 | 21.689623 | 80-84 |
| GCATG | 6279130 | 1.6109347 | 21.750547 | 80-84 |
| CAGGG | 8846930 | 1.6093689 | 11.11484 | 110-114 |
| CTTGC | 5992120 | 1.6010242 | 13.336396 | 8 |
| CATGC | 6817825 | 1.5963408 | 14.596135 | 80-84 |
| ATACC | 5478825 | 1.5854228 | 11.505566 | 110-114 |
| CAAGA | 5699535 | 1.5836464 | 16.242834 | 9 |
| AACGC | 7708105 | 1.581575 | 12.380775 | 40-44 |
| CCAGA | 7611525 | 1.5617585 | 12.413159 | 55-59 |
| GGGCA | 8536820 | 1.5529561 | 11.554252 | 80-84 |
| TCGTC | 5743005 | 1.5344635 | 20.7754 | 2 |
| CCAAC | 8189690 | 1.5335959 | 15.9978285 | 100-104 |
| TGAAT | 3128980 | 1.5331099 | 8.74877 | 8 |
| AAAAG | 4073860 | 1.532856 | 36.81471 | 135-137 |
| GTCTA | 4204810 | 1.5213891 | 20.987312 | 4 |
| CTCGG | 8022155 | 1.5198228 | 11.459737 | 15-19 |
| CGCCT | 8786255 | 1.5191722 | 7.563292 | 75-79 |
| AATTT | 2189455 | 1.5129409 | 12.524277 | 4 |
| ACCTC | 7052125 | 1.5069579 | 12.862229 | 90-94 |
| TCACC | 7051640 | 1.5068544 | 8.710978 | 65-69 |
| GGCGG | 10208385 | 1.5025958 | 6.989901 | 8 |
| TCGCC | 8683660 | 1.5014333 | 9.299013 | 9 |
| ACTTC | 4529185 | 1.4955994 | 5.7362194 | 70-74 |
| TCCAC | 6981595 | 1.4918865 | 6.943412 | 75-79 |
| TACGC | 6368615 | 1.4911621 | 11.404886 | 55-59 |
| GTGGT | 4590435 | 1.472545 | 9.208937 | 130-134 |
| CGTAG | 5733980 | 1.4710745 | 21.58412 | 35-39 |
| TCTGA | 4044895 | 1.4635285 | 6.452899 | 15-19 |
| CCCGC | 13072620 | 1.4626882 | 5.069618 | 30-34 |
| GGGTG | 6428130 | 1.462123 | 19.557707 | 115-119 |
| CCCTT | 5987290 | 1.459987 | 7.7305517 | 125-129 |
| AGGCC | 8793640 | 1.4599334 | 6.992056 | 70-74 |
| CTATA | 3260590 | 1.4580351 | 7.299892 | 60-64 |
| CTACG | 6226735 | 1.4579419 | 10.624083 | 75-79 |
| TCGAA | 4596805 | 1.4575131 | 5.586734 | 10-14 |
| AAAGA | 3872810 | 1.4572079 | 26.326464 | 135-137 |
| GGTGT | 4539325 | 1.4561497 | 9.602614 | 130-134 |
| CAAGG | 6457155 | 1.4517175 | 13.611325 | 25-29 |
| GCGTG | 6986620 | 1.4503329 | 13.049727 | 65-69 |
| TGCCT | 5423515 | 1.4490995 | 8.894393 | 120-124 |
| CGCAC | 9551390 | 1.4472122 | 12.667369 | 45-49 |
| GACGC | 8709490 | 1.4459625 | 16.532852 | 50-54 |
| CTCCC | 9160225 | 1.4454753 | 10.008026 | 15-19 |
| TAACT | 3229660 | 1.4442042 | 8.659835 | 70-74 |
| TCTAC | 4359360 | 1.4395208 | 19.235632 | 5 |
| TTGAA | 2932420 | 1.4368012 | 9.932989 | 7 |
| AAATA | 2705030 | 1.4354328 | 5.5166826 | 105-109 |
| TACGG | 5593420 | 1.4350133 | 10.084064 | 80-84 |
| ACCAG | 6943300 | 1.4246498 | 12.647758 | 55-59 |
| CCTTC | 5796540 | 1.4134731 | 7.707834 | 125-129 |
| AGAGG | 5718065 | 1.4086032 | 5.153404 | 100-104 |
| CCGGG | 10467685 | 1.4061677 | 8.614708 | 75-79 |
| GTCAC | 5958860 | 1.395221 | 10.313707 | 60-64 |
| GGACG | 7667845 | 1.3948785 | 16.168556 | 50-54 |
| TCCGA | 5944785 | 1.3919253 | 5.0918965 | 45-49 |
| TATAC | 3104980 | 1.3884512 | 8.6609745 | 60-64 |
| ACAAG | 4982730 | 1.3844783 | 16.249168 | 8 |
| GCGGT | 6664780 | 1.383523 | 10.312126 | 60-64 |
| TAGTC | 3775390 | 1.366016 | 19.380724 | 6 |
| CATTC | 4114050 | 1.3585161 | 60.68942 | 5 |
| CTCCT | 5560805 | 1.3559896 | 7.5760093 | 1 |
| GGGTC | 6480475 | 1.3452638 | 5.311841 | 9 |
| CTCTC | 5501640 | 1.3415624 | 7.9266825 | 1 |
| GGCGC | 9953885 | 1.3371468 | 8.581818 | 65-69 |
| TCGGA | 5181715 | 1.3293889 | 16.087193 | 20-24 |
| GGCTA | 5176420 | 1.3280302 | 5.0668507 | 55-59 |
| TAACA | 3347590 | 1.3117965 | 9.845901 | 8 |
| GTAGT | 3306865 | 1.3110192 | 7.633192 | 80-84 |
| GCCTC | 7565605 | 1.3081179 | 5.567493 | 120-124 |
| TGTGC | 4432590 | 1.2976979 | 13.102038 | 135-137 |
| ATGCG | 5037505 | 1.2923912 | 5.412095 | 55-59 |
| CTGAA | 4055950 | 1.2860237 | 6.2248845 | 20-24 |
| TCTCC | 5226300 | 1.2744213 | 7.1403246 | 2 |
| GAATA | 2953130 | 1.2679892 | 9.793121 | 15-19 |
| ACCGC | 8305085 | 1.258374 | 13.407301 | 10-14 |
| GTCGT | 4278985 | 1.2527281 | 18.516905 | 1 |
| ACGTT | 3447380 | 1.2473348 | 10.487792 | 135-137 |
| ACGTC | 5311845 | 1.2437273 | 5.4357076 | 55-59 |
| GTGGC | 5990580 | 1.2435678 | 17.47214 | 65-69 |
| CGCCA | 8158860 | 1.2362182 | 9.284485 | 50-54 |
| CAAGT | 3874860 | 1.2286054 | 58.05346 | 9 |
| ACAGC | 5954140 | 1.2216907 | 14.955471 | 8 |
| CACGG | 7330920 | 1.2170904 | 6.741927 | 85-89 |
| CAGTC | 5194675 | 1.2162929 | 8.021057 | 5 |
| ACGAA | 4351815 | 1.2091751 | 6.950455 | 125-129 |
| TTGGT | 2660380 | 1.2035769 | 6.436236 | 7 |
| GCTAT | 3325880 | 1.2033737 | 5.7178407 | 60-64 |
| CCAGG | 7232915 | 1.2008194 | 10.509103 | 110-114 |
| ACAGT | 3772290 | 1.1960834 | 6.2509465 | 15-19 |
| GCCTA | 5108065 | 1.1960138 | 9.464586 | 75-79 |
| GAGCT | 4661240 | 1.195859 | 5.3357105 | 115-119 |
| ACGGA | 5265290 | 1.1837587 | 9.305325 | 85-89 |
| GCCCG | 9523405 | 1.1675622 | 9.538279 | 125-129 |
| TTAGT | 2077955 | 1.1618342 | 30.234009 | 5 |
| GGTTC | 3955550 | 1.1580384 | 6.583727 | 65-69 |
| CGACA | 5603345 | 1.1497134 | 8.579008 | 105-109 |
| GTGCA | 4469145 | 1.1465763 | 11.642155 | 135-137 |
| GCGAC | 6871800 | 1.1408665 | 6.7541428 | 105-109 |
| ACGCA | 5556050 | 1.1400092 | 12.199525 | 40-44 |
| CGCGA | 6834055 | 1.1346 | 7.163753 | 100-104 |
| TAAAT | 1872990 | 1.1341848 | 6.129885 | 105-109 |
| CGGAC | 6809010 | 1.130442 | 11.242808 | 50-54 |
| CACCT | 5288140 | 1.1300147 | 14.242892 | 3 |
| CGTCT | 4214770 | 1.126137 | 16.556297 | 3 |
| ACCGA | 5474940 | 1.1233667 | 8.306409 | 65-69 |
| GTCCC | 6467820 | 1.1183072 | 32.392895 | 1 |
| AGTAG | 3207615 | 1.1143903 | 6.6691775 | 80-84 |
| AGTCT | 3068140 | 1.1101179 | 7.3901696 | 15-19 |
| CAACA | 4346675 | 1.1022427 | 5.0119524 | 10-14 |
| AATAG | 2560285 | 1.0993125 | 7.3034873 | 15-19 |
| CACGT | 4694200 | 1.0991105 | 5.6641016 | 135-137 |
| TTATC | 2147045 | 1.0955962 | 5.217999 | 50-54 |
| ACGCC | 7219160 | 1.0938363 | 9.608027 | 50-54 |
| CCGCT | 6302155 | 1.0896633 | 14.489904 | 10-14 |
| AAATT | 1792765 | 1.0856048 | 12.875254 | 3 |
| TCGTG | 3707915 | 1.08554 | 8.503658 | 130-134 |
| CGTAA | 3418515 | 1.0839117 | 7.6405935 | 6 |
| TACAG | 3403410 | 1.0791223 | 21.113401 | 7 |
| CTGCG | 5644025 | 1.0692785 | 8.869779 | 1 |
| TACGT | 2937090 | 1.0627012 | 5.8855586 | 60-64 |
| CACTA | 3672085 | 1.0626013 | 5.5046735 | 70-74 |
| ATTTG | 1898935 | 1.0617399 | 12.150583 | 5 |
| GCAGT | 4098030 | 1.0513653 | 8.851251 | 4 |
| ATCAT | 2332485 | 1.0430152 | 5.1848207 | 50-54 |
| AGGTT | 2627290 | 1.0415992 | 7.87326 | 65-69 |
| TATTG | 1859025 | 1.0394253 | 6.8373737 | 5 |
| TCGGT | 3536245 | 1.0352814 | 7.758885 | 7 |
| AACCG | 5039255 | 1.0339714 | 25.00149 | 9 |
| ACGGG | 5607690 | 1.02011 | 7.4637423 | 80-84 |
| TGCGC | 5368385 | 1.0170577 | 8.709105 | 2 |
| GAGTA | 2917285 | 1.0135237 | 5.7964163 | 75-79 |
| GTCAG | 3947655 | 1.012786 | 6.1793637 | 1 |
| TGCCC | 5833320 | 1.0086001 | 7.0149655 | 30-34 |
| GTAAC | 3180155 | 1.0083345 | 7.881654 | 7 |
| AATGT | 2039615 | 0.99935234 | 5.299587 | 5 |
| GCGCG | 7388290 | 0.9924997 | 5.944038 | 55-59 |
| GGATG | 3503685 | 0.9849228 | 5.478676 | 55-59 |
| GTCTG | 3320770 | 0.9721983 | 5.040979 | 15-19 |
| TTGGA | 2439965 | 0.9673334 | 5.1334586 | 95-99 |
| CCGCG | 7832935 | 0.960312 | 5.784277 | 30-34 |
| GATGC | 3700110 | 0.9492774 | 5.1995 | 55-59 |
| CAGCG | 5713995 | 0.948646 | 6.9369364 | 45-49 |
| TATCA | 2075710 | 0.92819345 | 5.539961 | 50-54 |
| GCTGG | 4425250 | 0.91862535 | 10.125161 | 5 |
| TCCCA | 4271625 | 0.9127971 | 37.942223 | 2 |
| CCCGT | 5270420 | 0.9112728 | 11.545486 | 125-129 |
| AGCTG | 3548190 | 0.9103016 | 5.384333 | 50-54 |
| CCCAT | 4186200 | 0.89454275 | 38.61642 | 3 |
| ACGAG | 3970145 | 0.8925802 | 5.011081 | 30-34 |
| ACTAC | 3081770 | 0.89178026 | 5.0065446 | 70-74 |
| GCGCA | 5285220 | 0.8774602 | 6.0619774 | 2 |
| GCCGC | 7122290 | 0.8731873 | 5.421912 | 70-74 |
| GGCCC | 7092270 | 0.8695069 | 7.870728 | 120-124 |
| GGCGA | 4716360 | 0.85796577 | 10.064307 | 1 |
| AAGCT | 2673620 | 0.8477271 | 5.3416343 | 45-49 |
| GAGGT | 2996620 | 0.84238154 | 5.2735496 | 65-69 |
| CAGAT | 2625895 | 0.8325949 | 6.7443757 | 3 |
| CTGGC | 4344500 | 0.82307935 | 9.037949 | 6 |
| GATGA | 2329625 | 0.80935884 | 9.268686 | 1 |
| TATAT | 1146295 | 0.79210424 | 7.5871153 | 3 |
| TGCGG | 3796205 | 0.7880437 | 6.1481633 | 50-54 |
| GCGAG | 4288915 | 0.78020805 | 24.667864 | 1 |
| TTATA | 1114060 | 0.76982945 | 7.60472 | 2 |
| TTCGG | 2620415 | 0.76716036 | 6.538504 | 70-74 |
| GTTAG | 1877890 | 0.74449664 | 21.203638 | 4 |
| CTTAT | 1451345 | 0.74059373 | 5.818265 | 1 |
| ACCTA | 2544480 | 0.7363032 | 18.610754 | 4 |
| ATAGC | 2309775 | 0.7323624 | 6.3738146 | 20-24 |
| CGCTG | 3800880 | 0.72008884 | 8.898866 | 4 |
| ATATG | 1450330 | 0.7106198 | 5.712175 | 5 |
| GGGCG | 4793785 | 0.70560837 | 5.1695986 | 2 |
| GTCGG | 3395985 | 0.7049631 | 5.1769814 | 1 |
| GAATG | 1929945 | 0.6705019 | 6.1086264 | 9 |
| CGAAT | 2028025 | 0.6430277 | 6.2236943 | 15-19 |
| TCAGA | 1974510 | 0.62605965 | 6.383848 | 2 |
| CTCGT | 2288095 | 0.61135215 | 7.2004 | 1 |
| AGCGC | 3196665 | 0.5307151 | 5.8201084 | 1 |
| CGCAG | 3090840 | 0.51314586 | 5.5776825 | 3 |
| AAGCC | 2063080 | 0.42330974 | 5.577453 | 5 |

Produced by FastQC (version 0.10.1)
