# Supplementary material for: Freshwater sponge hosts and their green algae symbionts: a tractable model to understand intracellular symbiosis
Source: PeerJ. 2021 Feb 11;9:e10654. doi: 10.7717/peerj.10654 (PMC7882143; doi:10.7717/peerj.10654)
Supplement: Supplemental Information 33 [file peerj-09-10654-s033.zip › EmInf2_Clean_Data2.fq_fastqc/fastqc_report.html]

EmInf2\_Clean\_Data2.fq.gz FastQC Report


FastQC Report

Tue 10 Sep 2019  
EmInf2\_Clean\_Data2.fq.gz

## Summary

- Basic Statistics
- Per base sequence quality
- Per sequence quality scores
- Per base sequence content
- Per base GC content
- Per sequence GC content
- Per base N content
- Sequence Length Distribution
- Sequence Duplication Levels
- Overrepresented sequences
- Kmer Content

## Basic Statistics

| Measure | Value |
| --- | --- |
| Filename | EmInf2\_Clean\_Data2.fq.gz |
| File type | Conventional base calls |
| Encoding | Sanger / Illumina 1.9 |
| Total Sequences | 28504992 |
| Filtered Sequences | 0 |
| Sequence length | 100-141 |
| %GC | 58 |

## Per base sequence quality

## Per sequence quality scores

## Per base sequence content

## Per base GC content

## Per sequence GC content

## Per base N content

## Sequence Length Distribution

## Sequence Duplication Levels

## Overrepresented sequences

| Sequence | Count | Percentage | Possible Source |
| --- | --- | --- | --- |
| CTCGGAGACGCCGGAGGGGACCCTGGGAAGAGTTCTCTTTTCTTCTTAAC | 772780 | 2.7110339129370744 | No Hit |
| CGGAGACGCCGGAGGGGACCCTGGGAAGAGTTCTCTTTTCTTCTTAACGG | 635356 | 2.2289288837548176 | No Hit |
| GGCAACTCCCGGTATGTCGCGAAGCGCGAATCTCCGTGGCCCGTAGGCGG | 581369 | 2.039533987590665 | No Hit |
| GCAGGTGCACACCACGAAGGGAGGCAACTCCCGGTATGTCGCGAAGCGCG | 414153 | 1.45291393170712 | No Hit |
| GTGCACACCACGAAGGGAGGCAACTCCCGGTATGTCGCGAAGCGCGAATC | 393614 | 1.3808598858754284 | No Hit |
| GTTTCGACGTGCCGGCACGCCGGCGAGGACTTCGGCCCTCGCAGGCGTAG | 335323 | 1.176365880053571 | No Hit |
| GGAGACGCCGGAGGGGACCCTGGGAAGAGTTCTCTTTTCTTCTTAACGGG | 283064 | 0.9930330799601699 | No Hit |
| AGCATATGTAGCCAGGCGTCGCCCCGCGTGAGGTTCAGGTTTCGACGTGC | 262141 | 0.9196319016683113 | No Hit |
| GGTGCACACCACGAAGGGAGGCAACTCCCGGTATGTCGCGAAGCGCGAAT | 261147 | 0.9161447931646499 | No Hit |
| GCCAGGCGTCGCCCCGCGTGAGGTTCAGGTTTCGACGTGCCGGCACGCCG | 231179 | 0.811012330752452 | No Hit |
| CAGGTTTCGACGTGCCGGCACGCCGGCGAGGACTTCGGCCCTCGCAGGCG | 221757 | 0.7779584712740842 | No Hit |
| CACGAAGGGAGGCAACTCCCGGTATGTCGCGAAGCGCGAATCTCCGTGGC | 213985 | 0.7506930715854964 | No Hit |
| GCCGGAGGGGACCCTGGGAAGAGTTCTCTTTTCTTCTTAACGGGCCATCA | 206614 | 0.7248344430336974 | No Hit |
| GTGCCGGCACGCCGGCGAGGACTTCGGCCCTCGCAGGCGTAGCCGACCGC | 189703 | 0.6655079924246251 | No Hit |
| GGGAAGAGTTCTCTTTTCTTCTTAACGGGCCATCACCCTGGAATCAGGTT | 180839 | 0.6344116848024374 | No Hit |
| GGAAGAGTTCTCTTTTCTTCTTAACGGGCCATCACCCTGGAATCAGGTTG | 174889 | 0.613538147984746 | No Hit |
| CTCTTTTCTTCTTAACGGGCCATCACCCTGGAATCAGGTTGGCTGGAGGT | 166210 | 0.5830908494905033 | No Hit |
| GGCGTCGCCCCGCGTGAGGTTCAGGTTTCGACGTGCCGGCACGCCGGCGA | 165861 | 0.5818665025410286 | No Hit |
| GGGACGTATAGCCGCGTCGTTCGGAGCGCGCCCGCGACCGAGGAGAGGGT | 165225 | 0.5796353144038771 | No Hit |
| GGGAAGCATATGTAGCCAGGCGTCGCCCCGCGTGAGGTTCAGGTTTCGAC | 162753 | 0.5709631491915521 | No Hit |
| GAGACGCCGGAGGGGACCCTGGGAAGAGTTCTCTTTTCTTCTTAACGGGC | 161487 | 0.5665218218619391 | No Hit |
| GGAAGCTCCCTGTAGCACGGTGCAACTCGCCATCTTGGCGACCGGCACCC | 160446 | 0.5628698299582052 | No Hit |
| CGACGTGCCGGCACGCCGGCGAGGACTTCGGCCCTCGCAGGCGTAGCCGA | 157555 | 0.552727746775021 | No Hit |
| CGTGCCGGCACGCCGGCGAGGACTTCGGCCCTCGCAGGCGTAGCCGACCG | 142124 | 0.498593369189509 | No Hit |
| CACATTTCCCCGCGGGCTGCAGGTGCACACCACGAAGGGAGGCAACTCCC | 141134 | 0.495120293315641 | No Hit |
| GACGTATAGCCGCGTCGTTCGGAGCGCGCCCGCGACCGAGGAGAGGGTCT | 136108 | 0.4774882939802263 | No Hit |
| GGCTGCAGGTGCACACCACGAAGGGAGGCAACTCCCGGTATGTCGCGAAG | 135270 | 0.4745484580385078 | No Hit |
| GTTCTCTTTTCTTCTTAACGGGCCATCACCCTGGAATCAGGTTGGCTGGA | 129118 | 0.45296627341624934 | No Hit |
| CCGGTATGTCGCGAAGCGCGAATCTCCGTGGCCCGTAGGCGGCCTTCGGT | 112965 | 0.39629900615302754 | No Hit |
| GCTGCAGGTGCACACCACGAAGGGAGGCAACTCCCGGTATGTCGCGAAGC | 112483 | 0.39460807426292205 | No Hit |
| GTCGGAAGCGAGGGTCGACGAAGCGGGCTGGCGGGGGGGCCCTCTCGGGG | 112353 | 0.3941520137946364 | No Hit |
| GAAGAGTTCTCTTTTCTTCTTAACGGGCCATCACCCTGGAATCAGGTTGG | 112332 | 0.394078342488221 | No Hit |
| GACGTGCCGGCACGCCGGCGAGGACTTCGGCCCTCGCAGGCGTAGCCGAC | 111371 | 0.39070700318035523 | No Hit |
| GGGGAAGCTCCCTGTAGCACGGTGCAACTCGCCATCTTGGCGACCGGCAC | 104669 | 0.36719533196150345 | No Hit |
| AGACGCCGGAGGGGACCCTGGGAAGAGTTCTCTTTTCTTCTTAACGGGCC | 101659 | 0.35663577804196545 | No Hit |
| TCGGAGACGCCGGAGGGGACCCTGGGAAGAGTTCTCTTTTCTTCTTAACG | 101385 | 0.3556745429011171 | No Hit |
| CCGGAGGGGACCCTGGGAAGAGTTCTCTTTTCTTCTTAACGGGCCATCAC | 98784 | 0.34654982537795487 | No Hit |
| GGCGAAGTTAGGGACGTATAGCCGCGTCGTTCGGAGCGCGCCCGCGACCG | 98574 | 0.345813112313801 | No Hit |
| GACGCCGGAGGGGACCCTGGGAAGAGTTCTCTTTTCTTCTTAACGGGCCA | 97633 | 0.3425119361549023 | No Hit |
| GGACGTATAGCCGCGTCGTTCGGAGCGCGCCCGCGACCGAGGAGAGGGTC | 96428 | 0.3382846064296387 | No Hit |
| TGCAGGTGCACACCACGAAGGGAGGCAACTCCCGGTATGTCGCGAAGCGC | 94463 | 0.331391077043628 | No Hit |
| CGGAGGGGACCCTGGGAAGAGTTCTCTTTTCTTCTTAACGGGCCATCACC | 89088 | 0.31253473075873867 | No Hit |
| CGTATAGCCGCGTCGTTCGGAGCGCGCCCGCGACCGAGGAGAGGGTCTCT | 87651 | 0.3074935085054576 | No Hit |
| CTGGAATCAGGTTGGCTGGAGGTAGGGTTGCATGCCCGGTAAAGCGCCAC | 86350 | 0.30292939566515226 | No Hit |
| GCAACTCCCGGTATGTCGCGAAGCGCGAATCTCCGTGGCCCGTAGGCGGC | 84180 | 0.2953166940022295 | No Hit |
| CTTTTCTTCTTAACGGGCCATCACCCTGGAATCAGGTTGGCTGGAGGTAG | 83194 | 0.29185765075815495 | No Hit |
| GAAGCGGGCTGGCGGGGGGGCCCTCTCGGGGGTCCTGCCGCCGGAGCGTG | 82489 | 0.2893843997570671 | No Hit |
| GTCCCGACTTTGCGGAAGGGATGTATTTATTAGATCCAAAGCCAATGCGG | 78490 | 0.27535527812110944 | No Hit |
| CATTTCCCCGCGGGCTGCAGGTGCACACCACGAAGGGAGGCAACTCCCGG | 77145 | 0.2706368063530767 | No Hit |
| CGCAACGACACATTTCCCCGCGGGCTGCAGGTGCACACCACGAAGGGAGG | 76444 | 0.26817758798178226 | No Hit |
| GTCGATTCAGACATTTGGCATTTGCGCTTGGCTGAAAAGCCAATGGCGCG | 75777 | 0.2658376469637318 | No Hit |
| CCCGCGTGAGGTTCAGGTTTCGACGTGCCGGCACGCCGGCGAGGACTTCG | 75772 | 0.26582010617649005 | No Hit |
| GGCGAATTGTAGCCGAGAGAGGCACCTGCGCTCGGCAGGCGGTCGACCAA | 74305 | 0.26067363919975844 | No Hit |
| TTCGACGTGCCGGCACGCCGGCGAGGACTTCGGCCCTCGCAGGCGTAGCC | 72906 | 0.25576572692951466 | No Hit |
| ATTTCCCCGCGGGCTGCAGGTGCACACCACGAAGGGAGGCAACTCCCGGT | 72291 | 0.2536082100987785 | No Hit |
| CCCGACTTTGCGGAAGGGATGTATTTATTAGATCCAAAGCCAATGCGGGG | 72180 | 0.25321880462201146 | No Hit |
| CAGGTGCACACCACGAAGGGAGGCAACTCCCGGTATGTCGCGAAGCGCGA | 70993 | 0.24905462173081824 | No Hit |
| CCCCGCGTGAGGTTCAGGTTTCGACGTGCCGGCACGCCGGCGAGGACTTC | 70549 | 0.24749699982375017 | No Hit |
| GTCGACGAAGCGGGCTGGCGGGGGGGCCCTCTCGGGGGTCCTGCCGCCGG | 68920 | 0.24178221134038558 | No Hit |
| AGCGAGGGTCGACGAAGCGGGCTGGCGGGGGGGCCCTCTCGGGGGTCCTG | 66264 | 0.23246454515756398 | No Hit |
| GGGGACCCTGGGAAGAGTTCTCTTTTCTTCTTAACGGGCCATCACCCTGG | 64035 | 0.22464486220518848 | No Hit |
| AGGCAACTCCCGGTATGTCGCGAAGCGCGAATCTCCGTGGCCCGTAGGCG | 63953 | 0.22435719329442363 | No Hit |
| CGAAGGGAGGCAACTCCCGGTATGTCGCGAAGCGCGAATCTCCGTGGCCC | 62796 | 0.22029825512668097 | No Hit |
| GTTCAGGTTTCGACGTGCCGGCACGCCGGCGAGGACTTCGGCCCTCGCAG | 61784 | 0.21674799978894924 | No Hit |
| GCCGCGTCGTTCGGAGCGCGCCCGCGACCGAGGAGAGGGTCTCTTCGACC | 57842 | 0.20291884312754763 | No Hit |
| GTCGCGAAGCGCGAATCTCCGTGGCCCGTAGGCGGCCTTCGGTGACCGCG | 57821 | 0.20284517182113226 | No Hit |
| GGAGGGGACCCTGGGAAGAGTTCTCTTTTCTTCTTAACGGGCCATCACCC | 57031 | 0.20007372743693458 | No Hit |
| GCATATGTAGCCAGGCGTCGCCCCGCGTGAGGTTCAGGTTTCGACGTGCC | 56988 | 0.19992287666665545 | No Hit |
| AGCGGGCTGGCGGGGGGGCCCTCTCGGGGGTCCTGCCGCCGGAGCGTGGA | 56316 | 0.19756539486136324 | No Hit |
| ACGAAGGGAGGCAACTCCCGGTATGTCGCGAAGCGCGAATCTCCGTGGCC | 55897 | 0.19609547689050394 | No Hit |
| CTTCGACCCGCCAGCGCAGGCCTTCGTGGCCGGAGCTCCCGCGTTCCGGT | 55873 | 0.19601128111174354 | No Hit |
| GCGAGGGTCGACGAAGCGGGCTGGCGGGGGGGCCCTCTCGGGGGTCCTGC | 55585 | 0.19500093176661829 | No Hit |
| GGCGGTGCTGTTACGGCGACCGGGTGGTGCCCTGACCCGCCTCTCGGGGC | 53783 | 0.1886792320446889 | No Hit |
| AAGCGAGGGTCGACGAAGCGGGCTGGCGGGGGGGCCCTCTCGGGGGTCCT | 53641 | 0.18818107368702297 | No Hit |
| TTTCGACGTGCCGGCACGCCGGCGAGGACTTCGGCCCTCGCAGGCGTAGC | 53315 | 0.18703741435886037 | No Hit |
| GGGAGGCAACTCCCGGTATGTCGCGAAGCGCGAATCTCCGTGGCCCGTAG | 52626 | 0.18462029387694617 | No Hit |
| CCCTGACCCGCCTCTCGGGGCGAAGTTAGGGACGTATAGCCGCGTCGTTC | 51277 | 0.17988778947912 | No Hit |
| AAGCGGGCTGGCGGGGGGGCCCTCTCGGGGGTCCTGCCGCCGGAGCGTGG | 49925 | 0.1751447606089488 | No Hit |
| GCCCCGCGTGAGGTTCAGGTTTCGACGTGCCGGCACGCCGGCGAGGACTT | 48060 | 0.1686020469677732 | No Hit |
| GGCGTGTGCCTGTAACCGTAGTGAATCAACGGGGCTTGATCTGGCGAATA | 47507 | 0.16666203589883483 | No Hit |
| AAGCATATGTAGCCAGGCGTCGCCCCGCGTGAGGTTCAGGTTTCGACGTG | 46168 | 0.16196461307549218 | No Hit |
| GAAGCTCCCTGTAGCACGGTGCAACTCGCCATCTTGGCGACCGGCACCCA | 45794 | 0.16065256218980872 | No Hit |
| GCCGGCACGCCGGCGAGGACTTCGGCCCTCGCAGGCGTAGCCGACCGCCG | 45622 | 0.16004915910869225 | No Hit |
| CTGGGAAGAGTTCTCTTTTCTTCTTAACGGGCCATCACCCTGGAATCAGG | 44649 | 0.15663572191144626 | No Hit |
| GTCTCTTCGACCCGCCAGCGCAGGCCTTCGTGGCCGGAGCTCCCGCGTTC | 43524 | 0.1526890447820508 | No Hit |
| GCGTGAGGTTCAGGTTTCGACGTGCCGGCACGCCGGCGAGGACTTCGGCC | 42993 | 0.15082621317697614 | No Hit |
| CCCGGTATGTCGCGAAGCGCGAATCTCCGTGGCCCGTAGGCGGCCTTCGG | 41860 | 0.1468514707879939 | No Hit |
| GAAGCATATGTAGCCAGGCGTCGCCCCGCGTGAGGTTCAGGTTTCGACGT | 41557 | 0.14578849908114339 | No Hit |
| CCTGACCCGCCTCTCGGGGCGAAGTTAGGGACGTATAGCCGCGTCGTTCG | 40874 | 0.14339242754391932 | No Hit |
| GGGACCCTGGGAAGAGTTCTCTTTTCTTCTTAACGGGCCATCACCCTGGA | 40829 | 0.1432345604587435 | No Hit |
| GGCGACCGGGTGGTGCCCTGACCCGCCTCTCGGGGCGAAGTTAGGGACGT | 40109 | 0.14070868709593043 | No Hit |
| CAGGCGTCGCCCCGCGTGAGGTTCAGGTTTCGACGTGCCGGCACGCCGGC | 39787 | 0.13957906039756124 | No Hit |
| CACCACGAAGGGAGGCAACTCCCGGTATGTCGCGAAGCGCGAATCTCCGT | 39226 | 0.13761098406903605 | No Hit |
| GCCCTGACCCGCCTCTCGGGGCGAAGTTAGGGACGTATAGCCGCGTCGTT | 38259 | 0.13421859581648016 | No Hit |
| GCGAACTCGGAGACGCCGGAGGGGACCCTGGGAAGAGTTCTCTTTTCTTC | 37927 | 0.13305388754362746 | No Hit |
| CTCTTCGACCCGCCAGCGCAGGCCTTCGTGGCCGGAGCTCCCGCGTTCCG | 37102 | 0.13015965764873744 | No Hit |
| GTATAGCCGCGTCGTTCGGAGCGCGCCCGCGACCGAGGAGAGGGTCTCTT | 37093 | 0.1301280842317023 | No Hit |
| GCTCCCTGTAGCACGGTGCAACTCGCCATCTTGGCGACCGGCACCCACCA | 36983 | 0.12974218691238362 | No Hit |
| CCCCGCGGGCTGCAGGTGCACACCACGAAGGGAGGCAACTCCCGGTATGT | 36670 | 0.1286441336310496 | No Hit |
| CAACTCCCGGTATGTCGCGAAGCGCGAATCTCCGTGGCCCGTAGGCGGCC | 35755 | 0.12543416956580797 | No Hit |
| GTGGGTTGCGGGCGGTGCTGTTACGGCGACCGGGTGGTGCCCTGACCCGC | 35658 | 0.1250938782933179 | No Hit |
| CGTCGCCCCGCGTGAGGTTCAGGTTTCGACGTGCCGGCACGCCGGCGAGG | 35014 | 0.12283462489657952 | No Hit |
| CGGCGACCGGGTGGTGCCCTGACCCGCCTCTCGGGGCGAAGTTAGGGACG | 34934 | 0.1225539723007114 | No Hit |
| CTCCGGCGCACAGCCGGCGAATTGTAGCCGAGAGAGGCACCTGCGCTCGG | 34772 | 0.12198565079407846 | No Hit |
| GCAACAAGTCCCGACTTTGCGGAAGGGATGTATTTATTAGATCCAAAGCC | 34006 | 0.1192984021886412 | No Hit |
| GTCGCCCCGCGTGAGGTTCAGGTTTCGACGTGCCGGCACGCCGGCGAGGA | 32999 | 0.11576568763815125 | No Hit |
| CGGTATGTCGCGAAGCGCGAATCTCCGTGGCCCGTAGGCGGCCTTCGGTG | 32883 | 0.11535874137414245 | No Hit |
| GACACATTTCCCCGCGGGCTGCAGGTGCACACCACGAAGGGAGGCAACTC | 32862 | 0.11528507006772709 | No Hit |
| CCTGGGAAGAGTTCTCTTTTCTTCTTAACGGGCCATCACCCTGGAATCAG | 32227 | 0.11305739008802389 | No Hit |
| CTCGGGGCGAAGTTAGGGACGTATAGCCGCGTCGTTCGGAGCGCGCCCGC | 32099 | 0.11260834593463488 | No Hit |
| CTTCTTAACGGGCCATCACCCTGGAATCAGGTTGGCTGGAGGTAGGGTTG | 31854 | 0.11174884735978877 | No Hit |
| CGGAAGCGAGGGTCGACGAAGCGGGCTGGCGGGGGGGCCCTCTCGGGGGT | 31738 | 0.11134190109578 | No Hit |
| GAGGCAACTCCCGGTATGTCGCGAAGCGCGAATCTCCGTGGCCCGTAGGC | 31533 | 0.11062272881886794 | No Hit |
| GTGAGGTTCAGGTTTCGACGTGCCGGCACGCCGGCGAGGACTTCGGCCCT | 31532 | 0.11061922066141958 | No Hit |
| GTAATTCTAGAGCTAATACATGCAACAAGTCCCGACTTTGCGGAAGGGAT | 31240 | 0.10959483868650094 | No Hit |
| AAGGGAGGCAACTCCCGGTATGTCGCGAAGCGCGAATCTCCGTGGCCCGT | 31021 | 0.10882655220531197 | No Hit |
| GCGTCGCCCCGCGTGAGGTTCAGGTTTCGACGTGCCGGCACGCCGGCGAG | 30871 | 0.10830032858805923 | No Hit |
| GTCGGGCTGCGGTCGGAAGCGAGGGTCGACGAAGCGGGCTGGCGGGGGGG | 30028 | 0.10534295185909892 | No Hit |
| CGCGAAGCGCGAATCTCCGTGGCCCGTAGGCGGCCTTCGGTGACCGCGCG | 29741 | 0.10433611067142204 | No Hit |
| CTTCGGCCCTCGCAGGCGTAGCCGACCGCCGCTTCCGCATTTCTCACCGG | 29585 | 0.10378883810947921 | No Hit |
| TCTTTTCTTCTTAACGGGCCATCACCCTGGAATCAGGTTGGCTGGAGGTA | 29520 | 0.10356080787533636 | No Hit |
| AGGGGACCCTGGGAAGAGTTCTCTTTTCTTCTTAACGGGCCATCACCCTG | 29189 | 0.10239960775993201 | No Hit |
| GCACACCACGAAGGGAGGCAACTCCCGGTATGTCGCGAAGCGCGAATCTC | 29031 | 0.10184531888309248 | No Hit |
| GGACCCTGGGAAGAGTTCTCTTTTCTTCTTAACGGGCCATCACCCTGGAA | 28869 | 0.10127699737645954 | No Hit |
| TCGACGTGCCGGCACGCCGGCGAGGACTTCGGCCCTCGCAGGCGTAGCCG | 28843 | 0.1011857852828024 | No Hit |

## Kmer Content

| Sequence | Count | Obs/Exp Overall | Obs/Exp Max | Max Obs/Exp Position |
| --- | --- | --- | --- | --- |
| TCTCT | 15219150 | 5.2816386 | 19.49941 | 30-34 |
| TTCTC | 13673650 | 4.7452903 | 23.405483 | 130-134 |
| TTCTT | 10325755 | 4.673373 | 33.415146 | 40-44 |
| TTTCT | 9808695 | 4.4393544 | 25.214128 | 35-39 |
| GAATC | 11677395 | 4.117865 | 16.904808 | 60-64 |
| ATCTC | 10417005 | 3.8211048 | 10.05562 | 120-124 |
| CTTCT | 10761410 | 3.734629 | 18.456888 | 110-114 |
| CTCTT | 10732615 | 3.7246363 | 12.982817 | 30-34 |
| ATCAA | 6695925 | 3.578677 | 11.913528 | 85-89 |
| CATTT | 7040080 | 3.3678598 | 11.200472 | 3 |
| TCAAA | 6107395 | 3.2641337 | 11.797128 | 85-89 |
| GTTCT | 10267325 | 3.240768 | 18.813543 | 130-134 |
| AATCA | 5965690 | 3.1883986 | 19.3685 | 60-64 |
| CTTCG | 12846405 | 3.1091511 | 13.145342 | 70-74 |
| AGGTT | 10010345 | 3.037524 | 11.227955 | 65-69 |
| CATCA | 7770180 | 3.0126235 | 14.997808 | 50-54 |
| CTGGA | 12730360 | 2.9619722 | 12.235707 | 60-64 |
| GCGAA | 12017715 | 2.955493 | 9.34005 | 40-44 |
| TCTTC | 8436545 | 2.92781 | 13.089143 | 35-39 |
| TTGAA | 6331825 | 2.91196 | 15.078064 | 135-137 |
| AAGAT | 5934200 | 2.8846054 | 10.67006 | 110-114 |
| TATGT | 6618035 | 2.8795044 | 23.88584 | 5 |
| AATCT | 5651035 | 2.8574073 | 10.615327 | 50-54 |
| GGTTG | 14226645 | 2.8483105 | 11.238042 | 85-89 |
| GCCAA | 10524485 | 2.845749 | 9.509834 | 80-84 |
| ATTTC | 5872340 | 2.8092318 | 10.462813 | 4 |
| CCATC | 9978265 | 2.8065367 | 19.150082 | 50-54 |
| GTAAA | 5752955 | 2.7965026 | 17.696642 | 95-99 |
| AATCC | 7203405 | 2.7928758 | 9.25499 | 105-109 |
| CCGGT | 16520055 | 2.788391 | 13.619106 | 9 |
| CCAAT | 7189330 | 2.787419 | 9.905616 | 100-104 |
| CTTAA | 5485350 | 2.77363 | 18.343176 | 40-44 |
| TATCA | 5483030 | 2.7724566 | 11.473625 | 65-69 |
| TTTTC | 6070855 | 2.7476313 | 22.979849 | 35-39 |
| CGAAG | 11065950 | 2.7214272 | 13.974295 | 3 |
| TCACC | 9594800 | 2.698681 | 12.401015 | 55-59 |
| AAGCG | 10933570 | 2.6888714 | 12.625565 | 100-104 |
| TAAAG | 5529990 | 2.6881196 | 17.672026 | 95-99 |
| TCTTT | 5907030 | 2.6734853 | 16.59262 | 30-34 |
| TGGCC | 15768800 | 2.6615877 | 10.262791 | 75-79 |
| ACCCT | 9361515 | 2.6330664 | 15.800387 | 55-59 |
| TTAAC | 5200070 | 2.62938 | 18.191032 | 40-44 |
| AAAGC | 7046470 | 2.6264277 | 16.342993 | 100-104 |
| CAACT | 6760020 | 2.6209683 | 32.462402 | 3 |
| ACACA | 6386315 | 2.6171699 | 6.3908477 | 8 |
| TCTTA | 5455565 | 2.6098535 | 26.243176 | 40-44 |
| CTTTT | 5728275 | 2.5925817 | 19.79594 | 35-39 |
| GGCAA | 10507050 | 2.5839782 | 21.238136 | 1 |
| AACTC | 6628145 | 2.5698383 | 34.474724 | 4 |
| ACGCT | 9911260 | 2.5354605 | 10.393192 | 105-109 |
| CGGTA | 10809145 | 2.5149634 | 12.079859 | 95-99 |
| CAGGT | 10742605 | 2.4994812 | 13.637708 | 2 |
| GACCG | 13974085 | 2.4930632 | 5.830967 | 80-84 |
| TTCGG | 11281680 | 2.483398 | 6.891489 | 70-74 |
| AAAAT | 3328965 | 2.4525578 | 11.569195 | 125-129 |
| GGCCA | 13716280 | 2.4470694 | 7.8116093 | 50-54 |
| GCTGG | 15928495 | 2.4452837 | 8.988633 | 75-79 |
| TGGAA | 7603610 | 2.4386992 | 16.936523 | 60-64 |
| ATCAC | 6141930 | 2.381325 | 14.236582 | 50-54 |
| AGCGC | 13322200 | 2.3767629 | 10.321172 | 100-104 |
| GACTT | 7086945 | 2.3643806 | 6.1080713 | 20-24 |
| GCTTC | 9748935 | 2.3594859 | 10.790052 | 110-114 |
| GGAAT | 7297895 | 2.3406477 | 21.686686 | 60-64 |
| CACAC | 7813315 | 2.322837 | 17.567322 | 8 |
| CAAAC | 5666095 | 2.3220172 | 10.253918 | 90-94 |
| CTCTA | 6306360 | 2.313262 | 18.730762 | 135-137 |
| GGAGG | 15653520 | 2.3101838 | 8.607468 | 75-79 |
| CAAGA | 6140730 | 2.2888317 | 8.575773 | 115-119 |
| CCTTC | 8512520 | 2.2651975 | 9.706496 | 70-74 |
| TTGCA | 6771685 | 2.2592022 | 18.56952 | 85-89 |
| TGCAT | 6760655 | 2.255522 | 12.682382 | 85-89 |
| GAAGA | 6641170 | 2.2513902 | 15.112445 | 25-29 |
| CGTAT | 6748145 | 2.2513487 | 8.407748 | 85-89 |
| TGAAA | 4624685 | 2.2480524 | 8.18692 | 120-124 |
| CGCTT | 9276130 | 2.2450554 | 8.885857 | 105-109 |
| CGGTG | 14548910 | 2.2334945 | 9.120827 | 125-129 |
| CGCGA | 12389255 | 2.21032 | 8.361851 | 40-44 |
| ATCAG | 6210170 | 2.1899266 | 15.227692 | 65-69 |
| CCCTG | 11759110 | 2.182249 | 7.7278514 | 15-19 |
| GGTGC | 14069845 | 2.1599503 | 12.413499 | 125-129 |
| TGGAG | 10199140 | 2.1583216 | 11.589967 | 75-79 |
| TCTAC | 5883925 | 2.1583064 | 30.810856 | 135-137 |
| GCAAC | 7952755 | 2.1503708 | 25.284615 | 2 |
| CCGCG | 16608610 | 2.1495378 | 5.5266643 | 85-89 |
| GAAGC | 8731895 | 2.1474178 | 8.5532055 | 3 |
| AGATC | 6084040 | 2.145449 | 8.965951 | 115-119 |
| GGGTT | 10545945 | 2.1113992 | 8.710517 | 85-89 |
| GTTGC | 9579380 | 2.1086764 | 13.544713 | 85-89 |
| CGTTC | 8710855 | 2.1082447 | 12.444484 | 130-134 |
| TGACC | 8240840 | 2.10814 | 6.2039237 | 80-84 |
| GCCAT | 8054220 | 2.0603995 | 13.404932 | 50-54 |
| GAAAA | 3967970 | 2.038733 | 5.8630557 | 120-124 |
| ATGTC | 6110535 | 2.0386262 | 7.2016144 | 10-14 |
| TTCTG | 6455160 | 2.0375001 | 17.997648 | 110-114 |
| TGGTG | 10152165 | 2.0325606 | 12.127166 | 115-119 |
| CAATC | 5242150 | 2.0324657 | 12.629222 | 100-104 |
| CGAAT | 5744295 | 2.0256429 | 11.958399 | 45-49 |
| TTCGA | 6045260 | 2.0168488 | 15.630244 | 3 |
| GTAGG | 9506095 | 2.0116608 | 12.31182 | 80-84 |
| ACTCC | 7084505 | 1.9926231 | 23.386883 | 5 |
| AAGAG | 5845075 | 1.9815098 | 17.479239 | 25-29 |
| TCTCA | 5392995 | 1.9782267 | 7.04269 | 70-74 |
| TAACG | 5607910 | 1.9775484 | 15.019836 | 45-49 |
| GGCTG | 12806945 | 1.9660746 | 7.643151 | 70-74 |
| ACTTC | 5320535 | 1.9516475 | 7.1897874 | 25-29 |
| GCATT | 5845890 | 1.950334 | 6.381546 | 65-69 |
| ACGAA | 5201540 | 1.938768 | 14.540878 | 2 |
| CGTAG | 8270580 | 1.9243156 | 8.981904 | 60-64 |
| AGGCG | 11757465 | 1.9078135 | 5.292421 | 4 |
| AGGGA | 8488685 | 1.8987193 | 7.335628 | 6 |
| CTTGA | 5680830 | 1.8952658 | 13.077504 | 135-137 |
| CGGAG | 11643635 | 1.889343 | 22.795654 | 3 |
| GTGGC | 12271160 | 1.8838229 | 9.237214 | 115-119 |
| CGCCA | 9600720 | 1.8832216 | 8.1991415 | 105-109 |
| GCGTA | 8029450 | 1.8682119 | 7.182211 | 80-84 |
| AGGTA | 5822820 | 1.8675481 | 11.628143 | 75-79 |
| AATAT | 2675000 | 1.8645157 | 6.336035 | 70-74 |
| GGTAA | 5800195 | 1.8602916 | 17.883095 | 95-99 |
| GCCGG | 15742550 | 1.8531015 | 11.179129 | 8 |
| GTATC | 5549950 | 1.8516011 | 11.028432 | 85-89 |
| TCAGG | 7938030 | 1.8469411 | 11.987887 | 65-69 |
| TTGTA | 4234975 | 1.842636 | 8.178599 | 125-129 |
| CTGGC | 10731270 | 1.8113121 | 6.6755238 | 95-99 |
| CACGC | 9221985 | 1.8089312 | 11.105242 | 105-109 |
| TAGCC | 7052925 | 1.8042521 | 16.04459 | 9 |
| CACCA | 6043520 | 1.7966911 | 16.927809 | 6 |
| AGGGT | 8465460 | 1.7914436 | 8.091565 | 80-84 |
| AAACG | 4801760 | 1.789758 | 12.077409 | 90-94 |
| AGTTC | 5301765 | 1.7688005 | 12.28268 | 25-29 |
| TCGAC | 6895740 | 1.7640415 | 12.230928 | 4 |
| TCCGC | 9469470 | 1.7573389 | 7.130559 | 100-104 |
| CAGGC | 9794835 | 1.7474592 | 6.145782 | 3 |
| GCCTT | 7190605 | 1.7403063 | 7.054779 | 70-74 |
| ATATT | 2636780 | 1.738795 | 6.1927867 | 70-74 |
| GAGGT | 8210820 | 1.7375572 | 7.780797 | 75-79 |
| CCACG | 8785005 | 1.7232157 | 11.039275 | 8 |
| ACGTG | 7397745 | 1.721233 | 10.897102 | 7 |
| AGAGT | 5366525 | 1.721201 | 17.645887 | 25-29 |
| TGAAT | 3728795 | 1.7148454 | 7.6891847 | 135-137 |
| GACCC | 8738540 | 1.7141012 | 11.207897 | 15-19 |
| GTATG | 5636910 | 1.7104554 | 10.0222845 | 30-34 |
| CTCCC | 8364395 | 1.7066799 | 21.29482 | 6 |
| CGCCG | 13165875 | 1.7039684 | 14.5361805 | 9 |
| AACGC | 6299080 | 1.7032282 | 6.619728 | 95-99 |
| GATCT | 5063400 | 1.6892759 | 10.865665 | 115-119 |
| GCAGG | 10409680 | 1.6891165 | 9.712104 | 1 |
| CAACG | 6245775 | 1.688815 | 7.1678996 | 125-129 |
| ACCGC | 8608565 | 1.6886063 | 5.5810637 | 75-79 |
| GGCGA | 10322490 | 1.6749687 | 5.4093747 | 1 |
| TGTAG | 5479440 | 1.662673 | 14.271128 | 7 |
| CCTGG | 9850495 | 1.6626477 | 7.75612 | 20-24 |
| TCTCC | 6237510 | 1.6598129 | 6.766721 | 50-54 |
| GGAAG | 7382730 | 1.6513432 | 13.117014 | 2 |
| CGAGG | 10129500 | 1.6436534 | 5.0368557 | 130-134 |
| AAATC | 3070630 | 1.6411165 | 9.6982155 | 125-129 |
| TTGGC | 7365910 | 1.6214329 | 11.323412 | 70-74 |
| CATGC | 6322005 | 1.6172711 | 11.136308 | 90-94 |
| GGTAT | 5317010 | 1.6133854 | 7.5313134 | 30-34 |
| CCAAC | 5390860 | 1.6026603 | 5.809899 | 85-89 |
| ATTCA | 3168705 | 1.602234 | 6.7793493 | 5 |
| ATCCG | 6228640 | 1.5933868 | 5.559172 | 100-104 |
| TGTTA | 3644235 | 1.5856053 | 7.3858356 | 105-109 |
| AACGT | 4460840 | 1.5730507 | 6.0877113 | 80-84 |
| CCCGC | 11041070 | 1.5711247 | 7.089269 | 9 |
| TCTTG | 4973450 | 1.5698144 | 6.4331946 | 120-124 |
| GCATG | 6739560 | 1.5680932 | 8.665168 | 85-89 |
| ATGCC | 6126475 | 1.5672513 | 13.096041 | 90-94 |
| AAGTT | 3354580 | 1.5427465 | 7.782101 | 5 |
| GACGT | 6613125 | 1.5386753 | 10.796064 | 6 |
| TCTGG | 6960245 | 1.5321351 | 9.039558 | 110-114 |
| GAGGG | 10378405 | 1.5316699 | 8.90209 | 10-14 |
| TGCCC | 8241085 | 1.5293759 | 10.313436 | 90-94 |
| TCCCG | 8155650 | 1.513521 | 15.656325 | 7 |
| TGGCT | 6865450 | 1.5112681 | 12.558904 | 70-74 |
| GGCGT | 9841045 | 1.5107607 | 5.8249907 | 115-119 |
| AACGG | 6121725 | 1.5055038 | 12.413169 | 45-49 |
| GAGTT | 4958480 | 1.5045936 | 11.063287 | 25-29 |
| GCGTT | 6834005 | 1.5043463 | 9.189382 | 125-129 |
| CTGTT | 4760755 | 1.5026797 | 5.4781127 | 100-104 |
| ACGCC | 7634575 | 1.4975538 | 21.551565 | 8 |
| GCGTC | 8858255 | 1.4951692 | 7.972372 | 120-124 |
| TACGG | 6411620 | 1.4917914 | 15.741902 | 135-137 |
| CCGGA | 8303860 | 1.4814601 | 15.963492 | 9 |
| GGTGA | 6978105 | 1.4766927 | 6.2307305 | 75-79 |
| TAATA | 2113500 | 1.4731417 | 7.8187776 | 70-74 |
| GGAGA | 6533720 | 1.4614397 | 24.831776 | 4 |
| GTAGC | 6256455 | 1.4556892 | 10.743521 | 8 |
| TCCGG | 8544070 | 1.4421386 | 8.735819 | 120-124 |
| GGGAA | 6434725 | 1.4392968 | 12.902809 | 1 |
| TTAAT | 2173880 | 1.4335408 | 7.4036274 | 70-74 |
| GGTAG | 6760270 | 1.4305948 | 9.41425 | 80-84 |
| TTCAT | 2961600 | 1.4167813 | 5.1092534 | 130-134 |
| GGTGG | 10133020 | 1.4148346 | 9.817338 | 115-119 |
| TTCAG | 4235465 | 1.4130561 | 5.212794 | 20-24 |
| CTTCC | 5264665 | 1.4009371 | 5.191439 | 60-64 |
| CTGGT | 6355200 | 1.3989486 | 8.377364 | 110-114 |
| AATGG | 4353920 | 1.396429 | 5.712324 | 135-137 |
| CACCC | 6465830 | 1.3944714 | 11.0304 | 55-59 |
| CCGTA | 5448735 | 1.3938744 | 6.214175 | 60-64 |
| AAGGG | 6229650 | 1.3934263 | 7.4711313 | 5 |
| CGTCC | 7493050 | 1.390556 | 10.353391 | 120-124 |
| AGACG | 5629330 | 1.38441 | 26.712059 | 6 |
| TAGGG | 6529080 | 1.3816708 | 11.66473 | 80-84 |
| AGGCA | 5527255 | 1.3593069 | 5.6507616 | 15-19 |
| GCAAG | 5525980 | 1.3589934 | 5.8509307 | 115-119 |
| ACCAC | 4569555 | 1.3584929 | 16.799307 | 7 |
| TCGGA | 5819805 | 1.3540938 | 29.384552 | 2 |
| ATTTT | 2166375 | 1.3515759 | 6.8821883 | 135-137 |
| ACACC | 4524075 | 1.3449719 | 17.329046 | 9 |
| TGGGA | 6321770 | 1.3378004 | 11.872542 | 20-24 |
| GTGCC | 7830340 | 1.3216692 | 8.169766 | 9 |
| GGCCT | 7764395 | 1.3105386 | 5.0389504 | 65-69 |
| GAAGG | 5833625 | 1.3048449 | 7.54548 | 4 |
| CCCGG | 10063100 | 1.3023976 | 10.517853 | 8 |
| CTTGT | 4116445 | 1.2993103 | 8.244863 | 120-124 |
| GTGCA | 5580360 | 1.2983822 | 14.155521 | 1 |
| GTGAC | 5514455 | 1.283048 | 7.656021 | 75-79 |
| GCACA | 4744380 | 1.282848 | 15.881977 | 7 |
| GTTGG | 6400915 | 1.2815244 | 8.679122 | 70-74 |
| CTCCG | 6901050 | 1.2806929 | 6.091111 | 50-54 |
| GTCGC | 7456285 | 1.2585331 | 8.281406 | 8 |
| ACATT | 2452975 | 1.2403301 | 10.5219345 | 2 |
| TGGCG | 8050845 | 1.2359358 | 5.864916 | 115-119 |
| CTACG | 4809565 | 1.2303644 | 14.514794 | 135-137 |
| TATAG | 2645135 | 1.2164781 | 11.048607 | 7 |
| GGGAC | 7494315 | 1.2160578 | 7.286515 | 15-19 |
| TAGAC | 3426320 | 1.2082423 | 6.957211 | 125-129 |
| ATGTA | 2623275 | 1.206425 | 17.988735 | 6 |
| ATATG | 2621470 | 1.2055948 | 17.462038 | 4 |
| TCCGT | 4980050 | 1.2052966 | 5.36188 | 55-59 |
| ACGGC | 6678130 | 1.1914197 | 8.40558 | 135-137 |
| TCATT | 2489800 | 1.1910799 | 5.103785 | 130-134 |
| GTAGA | 3680245 | 1.1803619 | 5.345816 | 120-124 |
| GCCAC | 6012770 | 1.17943 | 8.711249 | 105-109 |
| TGCGT | 5275465 | 1.1612701 | 8.872367 | 125-129 |
| TGCAC | 4512105 | 1.1542693 | 14.749939 | 6 |
| GCGCC | 8747935 | 1.1321849 | 5.4655414 | 100-104 |
| GACTA | 3209660 | 1.1318402 | 7.6949964 | 115-119 |
| GTATA | 2450220 | 1.1268382 | 10.936426 | 6 |
| GTTTC | 3547840 | 1.1198364 | 15.714454 | 1 |
| GTTAC | 3348220 | 1.1170493 | 5.1487427 | 110-114 |
| GTCCG | 6582315 | 1.1110173 | 10.018701 | 120-124 |
| CACAT | 2858635 | 1.1083388 | 8.57058 | 1 |
| GAAGT | 3439650 | 1.103196 | 6.5846453 | 4 |
| TTACG | 3301390 | 1.1014256 | 5.561426 | 110-114 |
| GAGGC | 6773365 | 1.0990733 | 5.331349 | 9 |
| ACTTG | 3234465 | 1.0790977 | 7.382016 | 130-134 |
| ACGAC | 3955900 | 1.0696484 | 5.084726 | 130-134 |
| CTCGG | 6312565 | 1.0654868 | 19.461946 | 1 |
| GTGCG | 6818365 | 1.04673 | 10.17572 | 125-129 |
| TAAAT | 1501095 | 1.0462861 | 5.2823644 | 3 |
| ATCTT | 2170540 | 1.038351 | 5.458411 | 30-34 |
| CACGA | 3817025 | 1.0320976 | 14.877408 | 9 |
| TTTCG | 3218685 | 1.0159423 | 14.802628 | 2 |
| AAGCT | 2878145 | 1.0149362 | 8.631576 | 3 |
| GAGCG | 6090650 | 0.98829335 | 5.559933 | 135-137 |
| GGACC | 5535745 | 0.98761123 | 9.156684 | 15-19 |
| AGCTC | 3847630 | 0.98428595 | 6.3155255 | 4 |
| AGTTA | 2132905 | 0.9809073 | 7.0960717 | 6 |
| AGGTG | 4586415 | 0.97056794 | 12.320527 | 3 |
| ATAGC | 2712520 | 0.9565309 | 9.049377 | 8 |
| TCCCT | 3592510 | 0.9559735 | 6.428758 | 7 |
| AATTG | 2072490 | 0.9531229 | 6.8920503 | 5 |
| CATAT | 1866435 | 0.94375014 | 19.264236 | 3 |
| GAGAC | 3816535 | 0.9385929 | 26.872086 | 5 |
| GGTTT | 3215505 | 0.92310715 | 9.021761 | 3 |
| AAGCA | 2383090 | 0.8882481 | 8.909613 | 4 |
| ACGTA | 2498020 | 0.8808906 | 8.228662 | 4 |
| CGTGC | 5183665 | 0.8749417 | 8.076564 | 8 |
| GCCAG | 4851005 | 0.8654493 | 6.547563 | 1 |
| ACGGG | 5305505 | 0.8608926 | 8.867774 | 45-49 |
| TCGCC | 4618110 | 0.8570262 | 6.1332173 | 9 |
| ACTTT | 1785890 | 0.85434073 | 5.68487 | 5 |
| CTGCA | 3309270 | 0.84656465 | 5.033048 | 3 |
| TCGCG | 4977365 | 0.8401206 | 5.4222145 | 35-39 |
| CGTCG | 4842700 | 0.81739086 | 5.7407126 | 7 |
| TTCCC | 3009845 | 0.8009254 | 5.945322 | 6 |
| CGACG | 4421270 | 0.7887819 | 8.45728 | 5 |
| GGGGA | 5329375 | 0.7865219 | 5.4800706 | 10-14 |
| CTGGG | 5000945 | 0.7677265 | 7.8386574 | 20-24 |
| ATTGT | 1729875 | 0.75266796 | 5.566462 | 6 |
| TTTCC | 2159180 | 0.7493196 | 7.6213136 | 5 |
| AGCAT | 2059560 | 0.72627413 | 13.424394 | 1 |
| GAATT | 1546415 | 0.7111849 | 6.320358 | 4 |
| AGGGG | 4815585 | 0.71069556 | 5.4670944 | 10-14 |
| GACGC | 3678935 | 0.6563448 | 19.48394 | 7 |
| GTCGA | 2784080 | 0.6477718 | 6.4373894 | 1 |
| CCTGT | 2631585 | 0.63690937 | 8.010204 | 9 |
| CCAGG | 3474960 | 0.6199544 | 5.9363203 | 2 |
| GCATA | 1492020 | 0.52613926 | 13.218887 | 2 |
| CCCCG | 3656350 | 0.5202921 | 6.1969004 | 8 |
| TCGAT | 1473020 | 0.49143595 | 5.06595 | 2 |

Produced by FastQC (version 0.10.1)
