# Supplementary material for: Freshwater sponge hosts and their green algae symbionts: a tractable model to understand intracellular symbiosis
Source: PeerJ. 2021 Feb 11;9:e10654. doi: 10.7717/peerj.10654 (PMC7882143; doi:10.7717/peerj.10654)
Supplement: Supplemental Information 34 [file peerj-09-10654-s034.zip › EmInf3_Clean_Data1.fq_fastqc/fastqc_report.html]

EmInf3\_Clean\_Data1.fq.gz FastQC Report


FastQC Report

Tue 10 Sep 2019  
EmInf3\_Clean\_Data1.fq.gz

## Summary

- Basic Statistics
- Per base sequence quality
- Per sequence quality scores
- Per base sequence content
- Per base GC content
- Per sequence GC content
- Per base N content
- Sequence Length Distribution
- Sequence Duplication Levels
- Overrepresented sequences
- Kmer Content

## Basic Statistics

| Measure | Value |
| --- | --- |
| Filename | EmInf3\_Clean\_Data1.fq.gz |
| File type | Conventional base calls |
| Encoding | Sanger / Illumina 1.9 |
| Total Sequences | 24542165 |
| Filtered Sequences | 0 |
| Sequence length | 100-141 |
| %GC | 58 |

## Per base sequence quality

## Per sequence quality scores

## Per base sequence content

## Per base GC content

## Per sequence GC content

## Per base N content

## Sequence Length Distribution

## Sequence Duplication Levels

## Overrepresented sequences

| Sequence | Count | Percentage | Possible Source |
| --- | --- | --- | --- |
| GTCCCATTCAAGTCGTCTACAAGAGATCTTGCCCCGCGGATTGGCCAGCG | 1078470 | 4.394355591692909 | No Hit |
| GCGAGAAAATGAACCGCTCCCTCGGATTTTCAAGGGCCGTAGAGAACGCA | 785865 | 3.2021013631030515 | No Hit |
| GAGAAAATGAACCGCTCCCTCGGATTTTCAAGGGCCGTAGAGAACGCACC | 511085 | 2.0824772386625225 | No Hit |
| CGGGCGAGAAAATGAACCGCTCCCTCGGATTTTCAAGGGCCGTAGAGAAC | 432960 | 1.76414753955081 | No Hit |
| AGAAAATGAACCGCTCCCTCGGATTTTCAAGGGCCGTAGAGAACGCACCG | 426461 | 1.7376665832048641 | No Hit |
| GGCGAGAAAATGAACCGCTCCCTCGGATTTTCAAGGGCCGTAGAGAACGC | 392940 | 1.6010812412026405 | No Hit |
| GCCACCTACAGCCAACAGTCTGAAGCGCAGTCGCGAACCCCGCGCACGGC | 353720 | 1.441274638973375 | No Hit |
| GTCGTCTACAAGAGATCTTGCCCCGCGGATTGGCCAGCGTTTGATACGCG | 328425 | 1.3382071223137812 | No Hit |
| CTGCGCTGGCGGGTCGAAGAGACCCTCTCCTCGGTCGCGGGCGCGCTCCG | 297566 | 1.2124684191472106 | No Hit |
| AAGAGATCTTGCCCCGCGGATTGGCCAGCGTTTGATACGCGCGGTCACCG | 260524 | 1.061536339601661 | No Hit |
| GCCGTTAGTCGCCTGCCGAATAGCCGCCGACCACGAGGGACGGCGACCAA | 259518 | 1.0574372717321394 | No Hit |
| ATTCAAGTCGTCTACAAGAGATCTTGCCCCGCGGATTGGCCAGCGTTTGA | 225174 | 0.9174985173475934 | No Hit |
| GTCGCCGTAACAGCACCGCCCGCAACCCACGTTGGCCAGCCCCGGTGAGA | 201550 | 0.8212396909563602 | No Hit |
| CTCTCCTCGGTCGCGGGCGCGCTCCGAACGACGCGGCTATACGTCCCTAA | 201471 | 0.8209177959646186 | No Hit |
| AGCGCAGTCGCGAACCCCGCGCACGGCGGAGGGATGCGCCGGCCTCGCAC | 191635 | 0.7808398321826946 | No Hit |
| GGCCGTTAGTCGCCTGCCGAATAGCCGCCGACCACGAGGGACGGCGACCA | 183762 | 0.7487603477525312 | No Hit |
| GTCAGATGAAGCCACCTACAGCCAACAGTCTGAAGCGCAGTCGCGAACCC | 163767 | 0.667288317880676 | No Hit |
| CTCCTCGGTCGCGGGCGCGCTCCGAACGACGCGGCTATACGTCCCTAACT | 153658 | 0.6260979827981761 | No Hit |
| GATGAAGCCACCTACAGCCAACAGTCTGAAGCGCAGTCGCGAACCCCGCG | 151600 | 0.6177124145322958 | No Hit |
| GGGCGAGAAAATGAACCGCTCCCTCGGATTTTCAAGGGCCGTAGAGAACG | 145308 | 0.5920749045571163 | No Hit |
| GAAAATGAACCGCTCCCTCGGATTTTCAAGGGCCGTAGAGAACGCACCGG | 141648 | 0.5771617948131308 | No Hit |
| GTCTACAAGAGATCTTGCCCCGCGGATTGGCCAGCGTTTGATACGCGCGG | 123091 | 0.5015490687150054 | No Hit |
| CTCGTCCCATTCAAGTCGTCTACAAGAGATCTTGCCCCGCGGATTGGCCA | 118426 | 0.4825409657216468 | No Hit |
| GCGCTGGCGGGTCGAAGAGACCCTCTCCTCGGTCGCGGGCGCGCTCCGAA | 116406 | 0.4743102330214144 | No Hit |
| GCAGAAATTTGAATGCACCATCGCCGGCACGAGGCCATGCGATTCGAGCA | 115287 | 0.4697507330750975 | No Hit |
| CGAGAAAATGAACCGCTCCCTCGGATTTTCAAGGGCCGTAGAGAACGCAC | 107797 | 0.4392318281618594 | No Hit |
| CCGCCCGCAACCCACGTTGGCCAGCCCCGGTGAGAAATGCGGAAGCGGCG | 105551 | 0.4300802313080366 | No Hit |
| GTCGGCCGTTAGTCGCCTGCCGAATAGCCGCCGACCACGAGGGACGGCGA | 94926 | 0.3867873922288437 | No Hit |
| AGAAATTTGAATGCACCATCGCCGGCACGAGGCCATGCGATTCGAGCAGT | 93977 | 0.3829205777077939 | No Hit |
| CCTGACTCTCCAAAGACACCTAATATCTAGGCAGGCGGTCGGCCGCGTAC | 89610 | 0.36512671151872705 | No Hit |
| AGAGATCTTGCCCCGCGGATTGGCCAGCGTTTGATACGCGCGGTCACCGA | 87890 | 0.3581183648630836 | No Hit |
| CACCCGGTCGCCGTAACAGCACCGCCCGCAACCCACGTTGGCCAGCCCCG | 87345 | 0.3558976968820803 | No Hit |
| CAAGAGATCTTGCCCCGCGGATTGGCCAGCGTTTGATACGCGCGGTCACC | 84447 | 0.34408944769135075 | No Hit |
| GAGATCTTGCCCCGCGGATTGGCCAGCGTTTGATACGCGCGGTCACCGAA | 81662 | 0.33274163057741646 | No Hit |
| CCCGCAACCCACGTTGGCCAGCCCCGGTGAGAAATGCGGAAGCGGCGGTC | 81179 | 0.3307735890456282 | No Hit |
| GCCCGCAACCCACGTTGGCCAGCCCCGGTGAGAAATGCGGAAGCGGCGGT | 78793 | 0.3210515453709972 | No Hit |
| CATTCAAGTCGTCTACAAGAGATCTTGCCCCGCGGATTGGCCAGCGTTTG | 77912 | 0.3174618050200543 | No Hit |
| CTTATATTGGTCGGGCTAGGAGCTGAGTCTACTCACAGGCACTATCCCAT | 77609 | 0.3162271951150194 | No Hit |
| CCCATTCAAGTCGTCTACAAGAGATCTTGCCCCGCGGATTGGCCAGCGTT | 75898 | 0.3092555200407136 | No Hit |
| CCTGCGCTGGCGGGTCGAAGAGACCCTCTCCTCGGTCGCGGGCGCGCTCC | 74624 | 0.3040644539713591 | No Hit |
| CCGGGCGAGAAAATGAACCGCTCCCTCGGATTTTCAAGGGCCGTAGAGAA | 74092 | 0.301896756052288 | No Hit |
| CCCGGTCGCCGTAACAGCACCGCCCGCAACCCACGTTGGCCAGCCCCGGT | 71262 | 0.2903655810316653 | No Hit |
| GGCAGAAATTTGAATGCACCATCGCCGGCACGAGGCCATGCGATTCGAGC | 69745 | 0.2841843822661937 | No Hit |
| TTCAAGTCGTCTACAAGAGATCTTGCCCCGCGGATTGGCCAGCGTTTGAT | 69549 | 0.28338575671706223 | No Hit |
| CGCGGATTGGCCAGCGTTTGATACGCGCGGTCACCGAAGGCCGCCTACGG | 65847 | 0.2683015129268343 | No Hit |
| CAAGTCGTCTACAAGAGATCTTGCCCCGCGGATTGGCCAGCGTTTGATAC | 64595 | 0.2632000885007496 | No Hit |
| GCCTGCGCTGGCGGGTCGAAGAGACCCTCTCCTCGGTCGCGGGCGCGCTC | 64390 | 0.2623647913702805 | No Hit |
| CCGCAACCCACGTTGGCCAGCCCCGGTGAGAAATGCGGAAGCGGCGGTCG | 61512 | 0.2506380345825236 | No Hit |
| CTGCTTACAACACCTCGTCCCATTCAAGTCGTCTACAAGAGATCTTGCCC | 59356 | 0.24185315354207748 | No Hit |
| GCGGGAGCTCCGGCCACGAAGGCCTGCGCTGGCGGGTCGAAGAGACCCTC | 57336 | 0.23362242084184504 | No Hit |
| AGATGAAGCCACCTACAGCCAACAGTCTGAAGCGCAGTCGCGAACCCCGC | 57245 | 0.23325163040831973 | No Hit |
| GAAGCCACCTACAGCCAACAGTCTGAAGCGCAGTCGCGAACCCCGCGCAC | 55482 | 0.22606807508628515 | No Hit |
| CCGAGATGGCGCCCTCCACCGGAACGCGGGAGCTCCGGCCACGAAGGCCT | 54985 | 0.2240429888724161 | No Hit |
| CTCCACCGGAACGCGGGAGCTCCGGCCACGAAGGCCTGCGCTGGCGGGTC | 54210 | 0.2208851582572279 | No Hit |
| CTACAAGAGATCTTGCCCCGCGGATTGGCCAGCGTTTGATACGCGCGGTC | 52996 | 0.2159385693967912 | No Hit |
| CAGATGAAGCCACCTACAGCCAACAGTCTGAAGCGCAGTCGCGAACCCCG | 50146 | 0.20432590197319592 | No Hit |
| GAAATTTGAATGCACCATCGCCGGCACGAGGCCATGCGATTCGAGCAGTT | 49692 | 0.20247602442571797 | No Hit |
| GGCGGGTCGAAGAGACCCTCTCCTCGGTCGCGGGCGCGCTCCGAACGACG | 49505 | 0.20171407045792414 | No Hit |
| CACCTACAGCCAACAGTCTGAAGCGCAGTCGCGAACCCCGCGCACGGCGG | 49121 | 0.20014941632085026 | No Hit |
| GCCGTAACAGCACCGCCCGCAACCCACGTTGGCCAGCCCCGGTGAGAAAT | 49106 | 0.2000882970186208 | No Hit |
| GGCCTGCGCTGGCGGGTCGAAGAGACCCTCTCCTCGGTCGCGGGCGCGCT | 48672 | 0.19831991187411543 | No Hit |
| GCCAACAGTCTGAAGCGCAGTCGCGAACCCCGCGCACGGCGGAGGGATGC | 47444 | 0.1933162783315979 | No Hit |
| CCGGTCGCCGTAACAGCACCGCCCGCAACCCACGTTGGCCAGCCCCGGTG | 46797 | 0.19067999909543434 | No Hit |
| CGAGATGGCGCCCTCCACCGGAACGCGGGAGCTCCGGCCACGAAGGCCTG | 45799 | 0.18661352818710167 | No Hit |
| AAAATGAACCGCTCCCTCGGATTTTCAAGGGCCGTAGAGAACGCACCGGA | 45600 | 0.18580267877752432 | No Hit |
| TGACTCTCCAAAGACACCTAATATCTAGGCAGGCGGTCGGCCGCGTACGG | 45533 | 0.1855296792275661 | No Hit |
| AAGCGCAGTCGCGAACCCCGCGCACGGCGGAGGGATGCGCCGGCCTCGCA | 44819 | 0.18262040044144434 | No Hit |
| GCCACGAAGGCCTGCGCTGGCGGGTCGAAGAGACCCTCTCCTCGGTCGCG | 44575 | 0.18162619312517864 | No Hit |
| GTCTACTTATATTGGTCGGGCTAGGAGCTGAGTCTACTCACAGGCACTAT | 43303 | 0.1764432762961214 | No Hit |
| CGCCGTAACAGCACCGCCCGCAACCCACGTTGGCCAGCCCCGGTGAGAAA | 43146 | 0.17580356093278648 | No Hit |
| GACCGGGCGAGAAAATGAACCGCTCCCTCGGATTTTCAAGGGCCGTAGAG | 42992 | 0.1751760694298975 | No Hit |
| CGTTAGTCGCCTGCCGAATAGCCGCCGACCACGAGGGACGGCGACCAAGC | 42655 | 0.1738029224398092 | No Hit |
| GCGCAGTCGCGAACCCCGCGCACGGCGGAGGGATGCGCCGGCCTCGCACT | 42514 | 0.1732284009988524 | No Hit |
| CACCGGAACGCGGGAGCTCCGGCCACGAAGGCCTGCGCTGGCGGGTCGAA | 42206 | 0.17197341799307436 | No Hit |
| GGGAAATGTGTCGTTGCGTTCTAGCGTGGATTCTGACTTAGAGGCGTTCA | 40251 | 0.1640075356025029 | No Hit |
| CTACTGCTTACAACACCTCGTCCCATTCAAGTCGTCTACAAGAGATCTTG | 38929 | 0.15862088776601413 | No Hit |
| CTCGGATTTTCAAGGGCCGTAGAGAACGCACCGGACGCCACCAGAAGCGT | 38863 | 0.15835196283620456 | No Hit |
| CTCGTCCCGGTTCGGGAATATTAACCCGATTCCCTTTCGATGGTGGGTGC | 38705 | 0.15770817285272104 | No Hit |
| GTTAGTCGCCTGCCGAATAGCCGCCGACCACGAGGGACGGCGACCAAGCT | 37568 | 0.1530753297437288 | No Hit |
| GCTCCCTCGGATTTTCAAGGGCCGTAGAGAACGCACCGGACGCCACCAGA | 36557 | 0.14895588877346394 | No Hit |
| CTCCAAAGACACCTAATATCTAGGCAGGCGGTCGGCCGCGTACGGGGTTC | 36307 | 0.1479372337363065 | No Hit |
| CTCGGTCGCGGGCGCGCTCCGAACGACGCGGCTATACGTCCCTAACTTCG | 35917 | 0.1463481318783408 | No Hit |
| CCTCGGTCGCGGGCGCGCTCCGAACGACGCGGCTATACGTCCCTAACTTC | 35505 | 0.14466938837710527 | No Hit |
| CAGAAATTTGAATGCACCATCGCCGGCACGAGGCCATGCGATTCGAGCAG | 35416 | 0.1443067471838772 | No Hit |
| AGATCTTGCCCCGCGGATTGGCCAGCGTTTGATACGCGCGGTCACCGAAG | 34231 | 0.13947832230775076 | No Hit |
| ACCGCCCGCAACCCACGTTGGCCAGCCCCGGTGAGAAATGCGGAAGCGGC | 34211 | 0.13939682990477817 | No Hit |
| GTCCGAGATGGCGCCCTCCACCGGAACGCGGGAGCTCCGGCCACGAAGGC | 33939 | 0.13828853322435083 | No Hit |
| CTCCAGCCAACCTGATTCCAGGGTGATGGCCCGTTAAGAAGAAAAGAGAA | 33774 | 0.1376162208998269 | No Hit |
| GCCCACTGGTGTTAGTTTTAGTACAGCCGAGCCCAATTTATTGGGCTGAA | 33721 | 0.13740026603194952 | No Hit |
| TACAAGAGATCTTGCCCCGCGGATTGGCCAGCGTTTGATACGCGCGGTCA | 33454 | 0.1363123424522653 | No Hit |
| CTCTACTGCTTACAACACCTCGTCCCATTCAAGTCGTCTACAAGAGATCT | 31851 | 0.12978072635401156 | No Hit |
| CCATTCAAGTCGTCTACAAGAGATCTTGCCCCGCGGATTGGCCAGCGTTT | 31264 | 0.1273889243267658 | No Hit |
| ACAAGAGATCTTGCCCCGCGGATTGGCCAGCGTTTGATACGCGCGGTCAC | 31113 | 0.12677365668432267 | No Hit |
| AAATGAACCGCTCCCTCGGATTTTCAAGGGCCGTAGAGAACGCACCGGAC | 31096 | 0.12670438814179596 | No Hit |
| CTACAGCCAACAGTCTGAAGCGCAGTCGCGAACCCCGCGCACGGCGGAGG | 30871 | 0.12578759860835423 | No Hit |
| GCGGGGAAATGTGTCGTTGCGTTCTAGCGTGGATTCTGACTTAGAGGCGT | 30781 | 0.12542088279497754 | No Hit |
| CCGCGGATTGGCCAGCGTTTGATACGCGCGGTCACCGAAGGCCGCCTACG | 30211 | 0.1230983493102585 | No Hit |
| CCACCTACAGCCAACAGTCTGAAGCGCAGTCGCGAACCCCGCGCACGGCG | 30182 | 0.12298018532594822 | No Hit |
| GCTTACAACACCTCGTCCCATTCAAGTCGTCTACAAGAGATCTTGCCCCG | 29533 | 0.1203357568494874 | No Hit |
| CAACAGTCTGAAGCGCAGTCGCGAACCCCGCGCACGGCGGAGGGATGCGC | 29362 | 0.11963899680407168 | No Hit |
| CCCTCTCCTCGGTCGCGGGCGCGCTCCGAACGACGCGGCTATACGTCCCT | 28761 | 0.1171901500947451 | No Hit |
| CGCCCGCAACCCACGTTGGCCAGCCCCGGTGAGAAATGCGGAAGCGGCGG | 28495 | 0.11610630113520955 | No Hit |
| CGCGGGAGCTCCGGCCACGAAGGCCTGCGCTGGCGGGTCGAAGAGACCCT | 28137 | 0.11464758712200003 | No Hit |
| GGTCGCCGTAACAGCACCGCCCGCAACCCACGTTGGCCAGCCCCGGTGAG | 28065 | 0.11435421447129868 | No Hit |
| CATTATTCAACCTGGATACAGCCGGACTCCTCCGGCGAACCCATATTGAT | 28063 | 0.11434606523100142 | No Hit |
| GTCCCGGTTCGGGAATATTAACCCGATTCCCTTTCGATGGTGGGTGCCGG | 27933 | 0.11381636461167953 | No Hit |
| CTGACTCTCCAAAGACACCTAATATCTAGGCAGGCGGTCGGCCGCGTACG | 27700 | 0.11286697811704875 | No Hit |
| ACCCGGTCGCCGTAACAGCACCGCCCGCAACCCACGTTGGCCAGCCCCGG | 27492 | 0.11201945712613375 | No Hit |
| CCGTTAGTCGCCTGCCGAATAGCCGCCGACCACGAGGGACGGCGACCAAG | 27455 | 0.11186869618063443 | No Hit |
| GCCAGCGTTTGATACGCGCGGTCACCGAAGGCCGCCTACGGGCCACGGAG | 26702 | 0.1088005072087161 | No Hit |
| CGGCCGTTAGTCGCCTGCCGAATAGCCGCCGACCACGAGGGACGGCGACC | 26449 | 0.10776962831111274 | No Hit |
| CCGGCGTCTCCGAGTTCGCTCGCGTTGCCGCCTCCGGCCCCTTGTTGAAG | 26249 | 0.10695470428138676 | No Hit |
| GCCCTCTCCTCGGTCGCGGGCGCGCTCCGAACGACGCGGCTATACGTCCC | 24904 | 0.1014743401814795 | No Hit |
| CACCACCCGGTCGCCGTAACAGCACCGCCCGCAACCCACGTTGGCCAGCC | 24895 | 0.10143766860014185 | No Hit |

## Kmer Content

| Sequence | Count | Obs/Exp Overall | Obs/Exp Max | Max Obs/Exp Position |
| --- | --- | --- | --- | --- |
| ATTTT | 4880920 | 4.6333985 | 50.131733 | 25-29 |
| TTCAA | 8316240 | 4.4273663 | 88.50353 | 7 |
| AGAAA | 9376055 | 4.2573223 | 54.32179 | 4 |
| GAGAT | 10154365 | 4.1780624 | 19.210276 | 90-94 |
| AGAGA | 11521940 | 4.1720576 | 21.833162 | 40-44 |
| GATTC | 9578635 | 4.06659 | 23.950424 | 110-114 |
| GGATT | 8447670 | 3.9496307 | 29.23225 | 20-24 |
| AAATG | 7438480 | 3.8379393 | 58.76531 | 7 |
| TTGAT | 5642910 | 3.759343 | 25.032408 | 50-54 |
| GAGAA | 10263460 | 3.716366 | 40.050613 | 3 |
| TTTTC | 5301555 | 3.6443214 | 37.92167 | 25-29 |
| TTTGA | 5465370 | 3.6410642 | 25.501833 | 50-54 |
| TTTCA | 5928580 | 3.5864651 | 38.357765 | 25-29 |
| AAGAA | 7858100 | 3.568075 | 48.06965 | 130-134 |
| TTCGC | 11072010 | 3.4038353 | 15.852519 | 95-99 |
| ATCTT | 5449000 | 3.296345 | 25.156921 | 6 |
| TGATT | 4818055 | 3.2098193 | 34.865738 | 110-114 |
| GATTT | 4761545 | 3.1721718 | 34.439014 | 25-29 |
| CTTCG | 10291565 | 3.1639054 | 12.230995 | 100-104 |
| AGAAG | 8678385 | 3.1424153 | 23.064344 | 60-64 |
| AAAAT | 4686860 | 3.0324078 | 70.607254 | 6 |
| ATGAA | 5807685 | 2.996518 | 56.884132 | 9 |
| TTTAC | 4862615 | 2.9416149 | 32.161716 | 75-79 |
| ATTGG | 6269750 | 2.931364 | 18.055145 | 40-44 |
| ACCGG | 15385590 | 2.9212556 | 13.926693 | 75-79 |
| TCCCT | 10456150 | 2.918912 | 17.843271 | 15-19 |
| TGATA | 4905095 | 2.8758006 | 22.113834 | 50-54 |
| TGATG | 6150565 | 2.8756406 | 26.1112 | 120-124 |
| CTTTA | 4722520 | 2.8568652 | 31.024181 | 75-79 |
| GATGG | 8689665 | 2.8512375 | 19.730661 | 120-124 |
| TCCAG | 10508695 | 2.8431082 | 16.005308 | 95-99 |
| TTGCC | 9176350 | 2.821058 | 13.026711 | 25-29 |
| TTGGC | 8268465 | 2.799367 | 14.13184 | 40-44 |
| CGAAG | 10663205 | 2.7959397 | 11.514175 | 65-69 |
| GAAAA | 6156095 | 2.7952569 | 49.654602 | 5 |
| GCTTT | 5765410 | 2.781342 | 27.098091 | 70-74 |
| AGATT | 4741230 | 2.7797284 | 21.75575 | 90-94 |
| CGCTT | 9040000 | 2.7791407 | 19.286774 | 70-74 |
| GCGCT | 12704325 | 2.7409708 | 12.379495 | 70-74 |
| TCAAG | 7232680 | 2.7022686 | 61.36823 | 8 |
| TACCG | 9893985 | 2.6767995 | 19.25851 | 75-79 |
| AGTTG | 5718865 | 2.6738033 | 16.848606 | 115-119 |
| TCGCG | 12302835 | 2.6543493 | 10.981616 | 95-99 |
| AAGAG | 7270805 | 2.6327353 | 14.773409 | 20-24 |
| GGCCA | 13812635 | 2.622599 | 8.239945 | 40-44 |
| CAACC | 12104675 | 2.617022 | 14.304472 | 105-109 |
| TTAAG | 4463495 | 2.6168957 | 34.165596 | 130-134 |
| GAGTT | 5586185 | 2.61177 | 16.406818 | 115-119 |
| ATACG | 6945245 | 2.5948772 | 14.523563 | 55-59 |
| GTTTG | 4864250 | 2.5842404 | 19.54067 | 50-54 |
| TCTTG | 5350180 | 2.581027 | 19.408382 | 7 |
| GTTAA | 4321295 | 2.5335255 | 33.237366 | 130-134 |
| AATGA | 4877760 | 2.5167167 | 56.374615 | 8 |
| TTCCA | 6497225 | 2.5047348 | 25.170816 | 110-114 |
| AGCGT | 8368390 | 2.4933279 | 16.271046 | 65-69 |
| CGGTC | 11521990 | 2.485881 | 10.660044 | 60-64 |
| GAAGA | 6861545 | 2.4845436 | 27.096748 | 135-137 |
| ACATA | 5176035 | 2.425036 | 17.878414 | 105-109 |
| GGTGA | 7329805 | 2.4050426 | 18.91383 | 115-119 |
| CGGAT | 7981880 | 2.378169 | 20.487144 | 20-24 |
| GGTCG | 9927730 | 2.3588245 | 10.76181 | 9 |
| ATTCC | 6098780 | 2.3511307 | 21.90995 | 110-114 |
| GAACG | 8957640 | 2.348733 | 15.019616 | 40-44 |
| CGCGG | 15476730 | 2.3433807 | 9.03027 | 35-39 |
| TGGCC | 10858475 | 2.3427272 | 13.011782 | 120-124 |
| GATCT | 5452190 | 2.314716 | 17.40393 | 5 |
| CTCCA | 9419125 | 2.3139942 | 18.92725 | 95-99 |
| GTGAT | 4929950 | 2.3049533 | 25.215014 | 120-124 |
| GAAGG | 7957505 | 2.2977874 | 11.043815 | 70-74 |
| CCTAC | 9320205 | 2.2896926 | 16.087439 | 90-94 |
| AACCT | 6694675 | 2.271255 | 20.126474 | 105-109 |
| CACCG | 13099240 | 2.2584403 | 11.917356 | 45-49 |
| GGTCA | 7522240 | 2.241221 | 12.002264 | 60-64 |
| TAAGA | 4341970 | 2.2402718 | 31.426731 | 130-134 |
| GCTCC | 11421035 | 2.2375124 | 12.421877 | 15-19 |
| CCAGC | 12884510 | 2.2214186 | 9.169811 | 100-104 |
| GAAGC | 8455875 | 2.217168 | 16.49559 | 60-64 |
| GGAGT | 6715590 | 2.2035077 | 11.782696 | 115-119 |
| CTGAT | 5161760 | 2.1914146 | 23.917086 | 105-109 |
| GATTG | 4677960 | 2.1871376 | 18.474045 | 35-39 |
| CCACG | 12637540 | 2.1788385 | 6.794626 | 85-89 |
| CGAGA | 8238640 | 2.1602077 | 30.583887 | 2 |
| GCCAC | 12403585 | 2.1385024 | 10.469956 | 1 |
| CCGAA | 8970410 | 2.1357944 | 10.750607 | 65-69 |
| ATGGC | 7155775 | 2.1320343 | 19.882109 | 120-124 |
| ATTCA | 4000730 | 2.1298926 | 85.93383 | 6 |
| AACTT | 3994195 | 2.1264136 | 8.865579 | 70-74 |
| ATTCG | 5004380 | 2.124599 | 16.886583 | 90-94 |
| TGTGT | 3990475 | 2.1200283 | 5.15728 | 7 |
| ATGCA | 5668025 | 2.117683 | 19.74145 | 85-89 |
| AGAAC | 6408720 | 2.107187 | 20.941463 | 40-44 |
| GAAAT | 4077410 | 2.1037703 | 10.795537 | 4 |
| TAGAG | 5105085 | 2.1005116 | 22.298973 | 40-44 |
| CGTTA | 4924840 | 2.0908308 | 25.060354 | 125-129 |
| TACAA | 4462470 | 2.090722 | 24.27133 | 7 |
| AACCC | 9668110 | 2.0902386 | 11.298928 | 90-94 |
| GTTGC | 6168020 | 2.0882416 | 12.6116085 | 115-119 |
| GTAGA | 5066780 | 2.084751 | 22.976105 | 35-39 |
| TGGCG | 8768140 | 2.0833068 | 12.728571 | 70-74 |
| CCTCG | 10629925 | 2.0825248 | 10.66315 | 20-24 |
| TGCAA | 5563615 | 2.0786736 | 20.597607 | 85-89 |
| GTGTG | 5556565 | 2.0717351 | 14.391336 | 135-137 |
| CGTTT | 4260295 | 2.0552464 | 18.799719 | 45-49 |
| TGAAC | 5410065 | 2.0213044 | 27.27407 | 8 |
| CAGAA | 6139820 | 2.0187728 | 18.185925 | 60-64 |
| GCACC | 11700605 | 2.0173013 | 10.974181 | 45-49 |
| CACCA | 9318415 | 2.0146348 | 12.462867 | 55-59 |
| CCTCC | 11239100 | 1.9993951 | 12.622259 | 95-99 |
| CTACA | 5884100 | 1.9962568 | 32.898357 | 6 |
| TTCGT | 4100980 | 1.9783896 | 20.234037 | 125-129 |
| GCTTC | 6417820 | 1.9730116 | 11.536998 | 100-104 |
| ACCTG | 7239595 | 1.9586592 | 16.056147 | 105-109 |
| AAGCG | 7392420 | 1.9383254 | 14.122837 | 65-69 |
| GATAC | 5158350 | 1.9272588 | 14.902705 | 50-54 |
| TACCT | 4985240 | 1.9218518 | 19.744911 | 95-99 |
| AAGTC | 5138970 | 1.9200182 | 13.950888 | 10-14 |
| AGGGC | 9154890 | 1.9142623 | 13.188946 | 30-34 |
| GACAT | 5116075 | 1.9114641 | 13.7192135 | 105-109 |
| TTACC | 4949920 | 1.9082357 | 21.379503 | 75-79 |
| GCAAC | 7877480 | 1.8755752 | 14.972112 | 85-89 |
| AGATG | 4473220 | 1.8405279 | 13.44812 | 4 |
| AGATC | 4924975 | 1.8400655 | 14.816623 | 20-24 |
| GGGAG | 7952130 | 1.8311529 | 10.107241 | 115-119 |
| AGGGT | 5570205 | 1.8276861 | 18.57836 | 115-119 |
| TTTTT | 1690175 | 1.82317 | 5.7515793 | 110-114 |
| ACCCT | 7413795 | 1.8213454 | 12.873739 | 90-94 |
| CCGTA | 6724140 | 1.8192039 | 14.784097 | 35-39 |
| CAGCC | 10514760 | 1.8128498 | 9.495539 | 100-104 |
| AACAG | 5509125 | 1.8114002 | 11.467737 | 9 |
| ACGCG | 9540055 | 1.8113662 | 9.602648 | 55-59 |
| TGGTG | 4836785 | 1.803369 | 10.111404 | 130-134 |
| AAGGC | 6860510 | 1.7988563 | 9.967299 | 70-74 |
| GCGTT | 5271300 | 1.7846484 | 13.519626 | 45-49 |
| GCGGG | 10676010 | 1.7801865 | 8.775222 | 9 |
| TCCTC | 6371720 | 1.778713 | 8.373695 | 4 |
| AGCCA | 7440025 | 1.7714199 | 13.29555 | 100-104 |
| GCGGA | 8465120 | 1.7700332 | 10.574161 | 35-39 |
| AGTCG | 5921230 | 1.7642066 | 18.726448 | 6 |
| GTCGC | 8158510 | 1.7602069 | 14.337993 | 8 |
| TTATT | 1844335 | 1.750805 | 5.9394917 | 3 |
| CGGGC | 11556180 | 1.7497579 | 13.566864 | 80-84 |
| GGGCC | 11542945 | 1.7477539 | 8.55356 | 30-34 |
| ATATT | 2090905 | 1.7467664 | 11.152786 | 4 |
| TGAAG | 4225020 | 1.7384048 | 11.260998 | 7 |
| GAACC | 7230345 | 1.7214966 | 17.336927 | 9 |
| CCCTC | 9665140 | 1.7193934 | 10.447425 | 15-19 |
| CCATT | 4455125 | 1.717488 | 61.236656 | 4 |
| GCCAA | 7183185 | 1.7102681 | 15.240832 | 100-104 |
| CGCGC | 12398360 | 1.7046492 | 5.4265018 | 55-59 |
| CGTGG | 7121845 | 1.6921476 | 14.951499 | 65-69 |
| TCGCC | 8507345 | 1.6666869 | 7.6234875 | 9 |
| GCCGT | 7716610 | 1.6648664 | 11.340703 | 35-39 |
| GCCAG | 8753605 | 1.6620433 | 8.551216 | 40-44 |
| AAGGG | 5722400 | 1.6523846 | 15.979574 | 30-34 |
| CATAC | 4864825 | 1.6504545 | 13.250862 | 105-109 |
| CCACC | 10541215 | 1.6502901 | 9.93484 | 55-59 |
| CAAGA | 4995870 | 1.6426418 | 15.762318 | 9 |
| CGGAG | 7850940 | 1.6416099 | 9.5968275 | 85-89 |
| GGCCG | 10839815 | 1.6412907 | 8.495398 | 35-39 |
| CCCTA | 6666765 | 1.6378226 | 14.301496 | 90-94 |
| CCGGA | 8609200 | 1.6346252 | 11.46333 | 50-54 |
| CGGGA | 7754340 | 1.6214111 | 10.729957 | 110-114 |
| TCGAA | 4332960 | 1.6188773 | 5.7357845 | 10-14 |
| TGAAT | 2751040 | 1.612903 | 8.55473 | 8 |
| GTCTA | 3781125 | 1.6052688 | 20.362349 | 4 |
| CCTGA | 5893955 | 1.5945988 | 17.765608 | 105-109 |
| CGCTC | 8137625 | 1.5942545 | 11.832057 | 15-19 |
| GGAGA | 5514500 | 1.592352 | 10.831047 | 90-94 |
| CTACC | 6481245 | 1.5922459 | 13.575231 | 90-94 |
| CTATA | 2983105 | 1.5881335 | 8.211537 | 60-64 |
| CAGGG | 7592495 | 1.5875695 | 12.364683 | 115-119 |
| TAACT | 2962025 | 1.576911 | 9.269011 | 70-74 |
| ACTTC | 4086240 | 1.5752797 | 5.808107 | 70-74 |
| CCGTT | 5116935 | 1.5730844 | 20.325062 | 125-129 |
| TAACA | 3355240 | 1.5719712 | 14.137623 | 8 |
| GGCGG | 9416235 | 1.5701234 | 7.3609495 | 8 |
| AATTT | 1874865 | 1.5662842 | 13.991178 | 6 |
| TCGTC | 5091020 | 1.5651174 | 21.881317 | 2 |
| ATACC | 4563280 | 1.5481515 | 12.356871 | 110-114 |
| CTTGC | 4995365 | 1.5357102 | 11.918096 | 25-29 |
| GGCAT | 5140830 | 1.5316895 | 18.787973 | 80-84 |
| TACGC | 5658925 | 1.5310117 | 12.1577 | 55-59 |
| GGGCA | 7316815 | 1.5299257 | 11.540572 | 80-84 |
| AACGC | 6395845 | 1.5228076 | 12.238768 | 45-49 |
| TATAC | 2852390 | 1.518544 | 9.284665 | 60-64 |
| CGCCT | 7737230 | 1.5158125 | 8.379602 | 75-79 |
| CCAGA | 6349865 | 1.5118603 | 12.804344 | 60-64 |
| TCTAC | 3920715 | 1.5114685 | 18.569628 | 5 |
| CATGC | 5585500 | 1.5111467 | 14.147203 | 85-89 |
| GGCTA | 5059820 | 1.5075529 | 5.099063 | 55-59 |
| CTACG | 5552105 | 1.5021119 | 12.165365 | 75-79 |
| TCTGA | 3535435 | 1.5009614 | 6.061107 | 15-19 |
| GCATG | 5026250 | 1.4975508 | 16.699087 | 80-84 |
| TTGAA | 2542975 | 1.490917 | 10.55851 | 9 |
| CTCGG | 6910330 | 1.4909108 | 11.735509 | 20-24 |
| AAATA | 2286285 | 1.4792311 | 5.940195 | 105-109 |
| ACCTC | 5982205 | 1.4696469 | 13.098189 | 95-99 |
| CCAAC | 6784025 | 1.4667014 | 12.241584 | 100-104 |
| TCACC | 5966705 | 1.465839 | 9.1700735 | 65-69 |
| TCCAC | 5963965 | 1.4651659 | 6.8580527 | 75-79 |
| AATGC | 3907875 | 1.4600574 | 5.4483027 | 135-137 |
| TGCCT | 4734150 | 1.4554058 | 9.757817 | 120-124 |
| ACGTT | 3415715 | 1.4501346 | 10.572905 | 135-137 |
| GCGTG | 6060540 | 1.439982 | 12.950031 | 65-69 |
| TCCGA | 5312765 | 1.4373589 | 5.2226796 | 45-49 |
| CCCGC | 11502250 | 1.4360206 | 5.1067147 | 30-34 |
| ACAAG | 4363945 | 1.434865 | 15.8027115 | 8 |
| CCCTT | 5105250 | 1.4251684 | 8.392591 | 125-129 |
| GCGGT | 5980695 | 1.421011 | 11.186933 | 60-64 |
| GACGC | 7478700 | 1.4199774 | 13.107474 | 50-54 |
| TGAGA | 3450870 | 1.419877 | 5.4698787 | 130-134 |
| CTCTC | 5073330 | 1.4162577 | 9.640939 | 1 |
| ATGCG | 4749820 | 1.4151896 | 6.3984313 | 55-59 |
| CATTC | 3669295 | 1.4145439 | 61.968807 | 5 |
| GTGGT | 3785080 | 1.4112467 | 9.620123 | 130-134 |
| TACGG | 4734045 | 1.4104896 | 10.726499 | 80-84 |
| CGTAG | 4708975 | 1.4030201 | 18.659723 | 35-39 |
| AGGCC | 7388775 | 1.4029036 | 7.154914 | 70-74 |
| CCTTC | 4999640 | 1.3956866 | 8.203441 | 125-129 |
| CAAGG | 5322095 | 1.3954767 | 13.853375 | 30-34 |
| GGTGT | 3711850 | 1.3839431 | 10.018194 | 130-134 |
| ACCAG | 5793070 | 1.3792909 | 12.406313 | 60-64 |
| CTCCT | 4930485 | 1.3763815 | 8.578156 | 3 |
| GGGTG | 5240910 | 1.3713405 | 16.764421 | 115-119 |
| CGCAC | 7881525 | 1.3588539 | 12.110881 | 45-49 |
| TAGTC | 3186405 | 1.3527814 | 15.976591 | 6 |
| CTCCC | 7589080 | 1.3500699 | 9.799318 | 15-19 |
| CTGAA | 3609150 | 1.3484479 | 5.2997065 | 20-24 |
| GTCAC | 4963325 | 1.3428185 | 10.735163 | 60-64 |
| CCGGG | 8812205 | 1.3342838 | 8.188522 | 80-84 |
| ACAGC | 5593960 | 1.3318841 | 13.739111 | 8 |
| GTAGT | 2809520 | 1.3135654 | 6.6150146 | 80-84 |
| TCTCC | 4695440 | 1.3107669 | 8.807542 | 2 |
| GGACG | 6256485 | 1.3082135 | 13.787998 | 50-54 |
| AAAAG | 2878725 | 1.3071234 | 25.5987 | 135-137 |
| CACGT | 4822770 | 1.3047916 | 6.2409377 | 135-137 |
| GCTAT | 3033070 | 1.2876836 | 6.38585 | 60-64 |
| GGCGC | 8497990 | 1.2867074 | 9.347329 | 70-74 |
| GTCGT | 3792495 | 1.283985 | 17.59866 | 1 |
| CGTAA | 3431700 | 1.2821492 | 10.887907 | 6 |
| AAAGA | 2821315 | 1.2810556 | 15.327756 | 135-137 |
| ACCGC | 7404450 | 1.2766014 | 11.37637 | 10-14 |
| CAAGT | 3415645 | 1.2761506 | 59.96437 | 9 |
| GCCTC | 6501560 | 1.2737305 | 5.8762183 | 120-124 |
| TTGGT | 2378910 | 1.2638487 | 7.6332026 | 7 |
| GAATA | 2441850 | 1.2598908 | 9.087552 | 15-19 |
| TCGGA | 4222885 | 1.2581916 | 16.215803 | 20-24 |
| ACGAA | 3773945 | 1.2408731 | 6.0673227 | 125-129 |
| TTTGG | 2319075 | 1.23206 | 5.4296613 | 95-99 |
| ACAGT | 3224600 | 1.2047725 | 5.311795 | 15-19 |
| TGTGC | 3554475 | 1.2034011 | 13.261008 | 135-137 |
| GTAAC | 3220490 | 1.203237 | 11.1846285 | 7 |
| CAGTC | 4433330 | 1.1994293 | 7.669192 | 5 |
| TAAAT | 1615105 | 1.1874187 | 7.2194295 | 105-109 |
| CGGCT | 5470600 | 1.1802875 | 5.1184683 | 70-74 |
| CGCCA | 6841265 | 1.1795026 | 9.461989 | 55-59 |
| CACGG | 6160600 | 1.1697105 | 6.926573 | 85-89 |
| GTGGC | 4901245 | 1.1645341 | 13.240783 | 65-69 |
| GCCCG | 8414170 | 1.1568633 | 10.557162 | 125-129 |
| GGTTC | 3411365 | 1.1549499 | 5.8183084 | 65-69 |
| TACGT | 2720010 | 1.1547745 | 5.9883156 | 60-64 |
| GCCTA | 4262900 | 1.1533198 | 9.832187 | 75-79 |
| ACGGA | 4396840 | 1.1528708 | 9.73924 | 85-89 |
| AAATT | 1564290 | 1.1500597 | 12.910005 | 5 |
| TTAGT | 1722070 | 1.147254 | 25.238852 | 5 |
| CCAGG | 6037130 | 1.1462673 | 10.323891 | 115-119 |
| GTCCC | 5839550 | 1.1440351 | 31.772598 | 1 |
| CGTCT | 3720350 | 1.1437362 | 15.559341 | 3 |
| AGTCT | 2668530 | 1.132919 | 6.667498 | 15-19 |
| AGTCC | 4177640 | 1.1302528 | 5.096233 | 90-94 |
| AACGA | 3410970 | 1.1215267 | 5.0886884 | 50-54 |
| AGTAG | 2723055 | 1.120414 | 5.6796117 | 80-84 |
| CGACA | 4694865 | 1.1178157 | 9.071613 | 105-109 |
| TTATC | 1841880 | 1.114236 | 6.311303 | 50-54 |
| TCGGT | 3275870 | 1.1090767 | 9.868932 | 7 |
| CACCT | 4474850 | 1.0993354 | 12.489788 | 3 |
| CTGCG | 5081520 | 1.0963432 | 9.35941 | 1 |
| GCGAC | 5772945 | 1.0961066 | 7.0243287 | 105-109 |
| AATAG | 2124315 | 1.0960562 | 6.0976934 | 15-19 |
| ACGCC | 6355585 | 1.0957664 | 10.472293 | 55-59 |
| GTCAG | 3673175 | 1.0944077 | 8.910279 | 1 |
| CGCGA | 5739535 | 1.089763 | 7.395349 | 100-104 |
| ATTTG | 1630095 | 1.0859797 | 11.98613 | 5 |
| CCTAA | 3197820 | 1.0849017 | 5.040855 | 70-74 |
| TACAG | 2892855 | 1.0808263 | 18.964859 | 7 |
| ACCGA | 4536710 | 1.0801603 | 8.667361 | 65-69 |
| TCGTG | 3182280 | 1.077391 | 9.310372 | 130-134 |
| TATTG | 1608235 | 1.0714164 | 8.313365 | 5 |
| ACGCA | 4493720 | 1.0699245 | 12.4486065 | 45-49 |
| ATCAT | 2006430 | 1.0681752 | 5.359276 | 50-54 |
| GTGCA | 3579050 | 1.0663636 | 12.052486 | 135-137 |
| CGGAC | 5589105 | 1.061201 | 10.9307575 | 50-54 |
| ATAAA | 1632445 | 1.0561953 | 5.6461573 | 105-109 |
| GCAGT | 3475155 | 1.0354084 | 8.475636 | 4 |
| AGGTT | 2205340 | 1.0310867 | 7.5738363 | 65-69 |
| GAGTA | 2461950 | 1.0129812 | 5.449575 | 75-79 |
| CTAAC | 2977270 | 1.0100772 | 5.262781 | 70-74 |
| AATGT | 1715910 | 1.0060183 | 5.0084634 | 5 |
| CCGCT | 5129805 | 1.004988 | 10.823002 | 10-14 |
| ACGGG | 4744890 | 0.9921432 | 7.8774524 | 80-84 |
| TGCGC | 4596835 | 0.9917719 | 9.120279 | 2 |
| TTGGA | 2108395 | 0.98576087 | 5.8080773 | 95-99 |
| GCGCG | 6380050 | 0.96602356 | 6.0776176 | 55-59 |
| AACCG | 4054270 | 0.9652944 | 14.299828 | 9 |
| TGCCC | 4890480 | 0.9581014 | 7.238789 | 30-34 |
| TATCA | 1776480 | 0.9457553 | 6.2232533 | 50-54 |
| GGCGA | 4517130 | 0.9445193 | 12.897893 | 1 |
| TCCCA | 3802590 | 0.9341814 | 38.19932 | 2 |
| GCGAG | 4452585 | 0.93102324 | 22.845852 | 1 |
| CCCAT | 3742700 | 0.9194683 | 38.977604 | 3 |
| GCTGG | 3858165 | 0.9166985 | 10.679346 | 5 |
| CAGCG | 4823290 | 0.91579604 | 7.243552 | 45-49 |
| CCGCG | 6581450 | 0.90488285 | 5.7842603 | 30-34 |
| AGCTG | 3003895 | 0.8949984 | 5.3372865 | 50-54 |
| TACTT | 1467145 | 0.88754195 | 5.050472 | 4 |
| CAGAT | 2335660 | 0.8726476 | 9.925003 | 3 |
| TGCGG | 3630070 | 0.86250323 | 6.0440903 | 50-54 |
| GCGCA | 4523785 | 0.85892916 | 5.7516007 | 2 |
| TATAT | 1022075 | 0.8538534 | 9.41139 | 3 |
| GATGA | 2072450 | 0.8527195 | 10.459956 | 5 |
| CCCGT | 4339275 | 0.850114 | 11.342164 | 125-129 |
| TTATA | 998450 | 0.8341168 | 9.459061 | 2 |
| CTGGC | 3837620 | 0.82797045 | 9.490056 | 6 |
| GCCGC | 5958020 | 0.81916755 | 5.469348 | 70-74 |
| TGCTT | 1697150 | 0.81873703 | 5.720693 | 2 |
| GAGGT | 2484735 | 0.81528693 | 5.168099 | 65-69 |
| GGCCC | 5856455 | 0.8052033 | 7.63286 | 125-129 |
| GTCGG | 3380470 | 0.8031984 | 5.1412454 | 1 |
| ACTCT | 2070115 | 0.79804665 | 5.839112 | 5 |
| GGGCG | 4744450 | 0.79112 | 10.237628 | 2 |
| CTTAT | 1281115 | 0.7750042 | 7.0668435 | 1 |
| TTCGG | 2230315 | 0.75509423 | 6.5090723 | 70-74 |
| ACCTA | 2218340 | 0.7526005 | 16.603485 | 4 |
| GTTAG | 1538750 | 0.71942854 | 17.452637 | 4 |
| ATAGC | 1897440 | 0.7089201 | 6.3960967 | 20-24 |
| CGCTG | 3284215 | 0.70857275 | 9.340581 | 4 |
| TGACT | 1668765 | 0.70847076 | 5.8296213 | 3 |
| TCTAA | 1306670 | 0.6956397 | 5.539476 | 100-104 |
| GAATG | 1655825 | 0.6812971 | 5.896224 | 9 |
| TCAGA | 1756190 | 0.6561464 | 9.578118 | 2 |
| CTCGT | 2083040 | 0.6403829 | 9.82907 | 1 |
| CGAAT | 1707410 | 0.6379213 | 5.9996986 | 15-19 |
| AGCGC | 2784995 | 0.52878577 | 5.500622 | 1 |
| CGCAG | 2660270 | 0.50510436 | 5.276794 | 3 |
| AAGCC | 1801255 | 0.4288667 | 5.961192 | 9 |

Produced by FastQC (version 0.10.1)
