# Supplementary figures and images for: Freshwater sponge hosts and their green algae symbionts: a tractable model to understand intracellular symbiosis (part 2 of 2)
Source: PeerJ. 2021 Feb 11;9:e10654. doi: 10.7717/peerj.10654 (PMC7882143; doi:10.7717/peerj.10654)

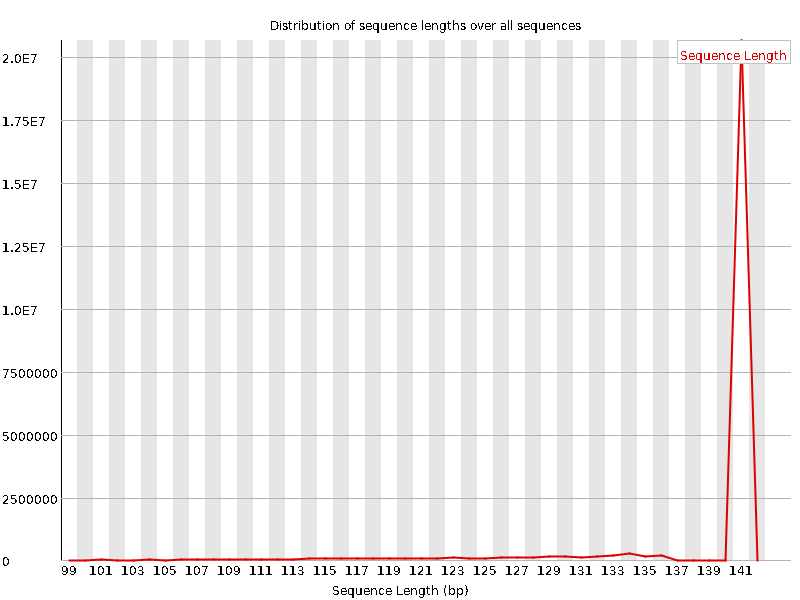

Supplement: Supplemental Information 34 [file peerj-09-10654-s034.zip › EmInf3_Clean_Data1.fq_fastqc/Images/sequence_length_distribution.png]

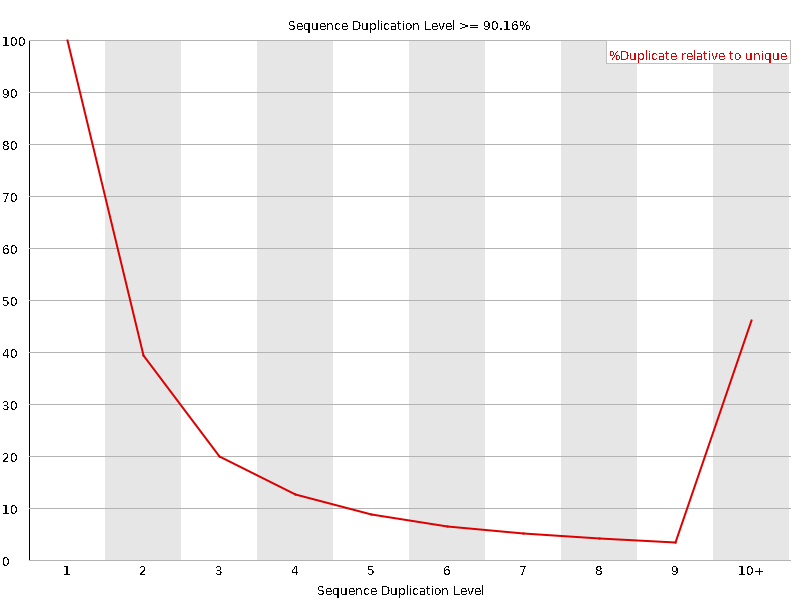

Supplement: Supplemental Information 34 [file peerj-09-10654-s034.zip › EmInf3_Clean_Data1.fq_fastqc/Images/duplication_levels.png]

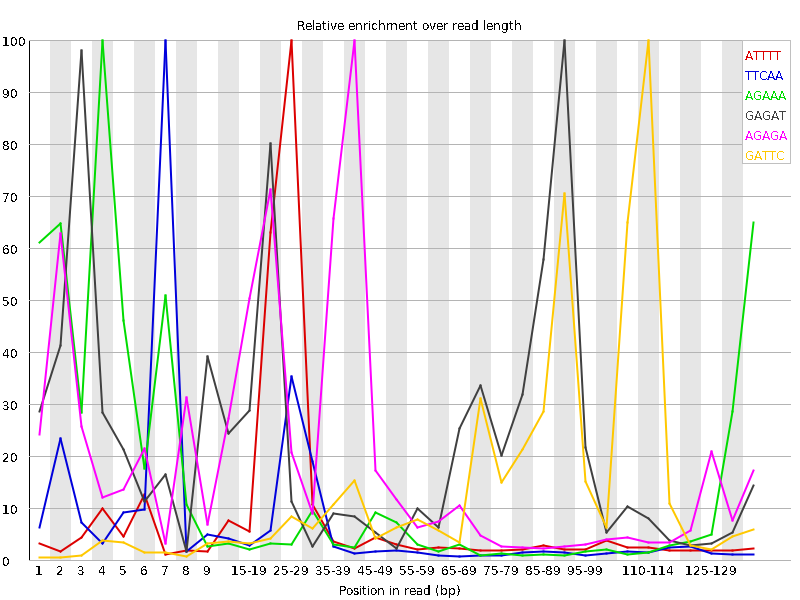

Supplement: Supplemental Information 34 [file peerj-09-10654-s034.zip › EmInf3_Clean_Data1.fq_fastqc/Images/kmer_profiles.png]

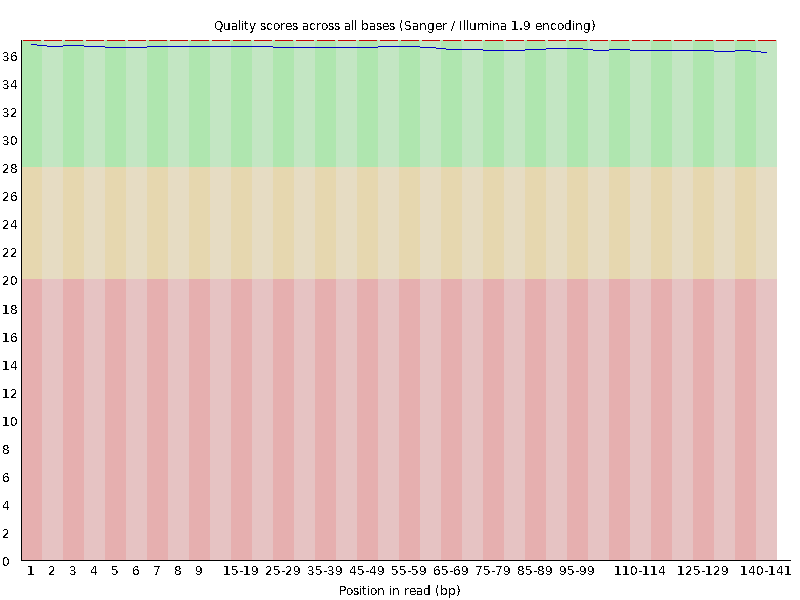

Supplement: Supplemental Information 35 [file peerj-09-10654-s035.zip › EmInf3_Clean_Data2.fq_fastqc/Images/per_base_quality.png]

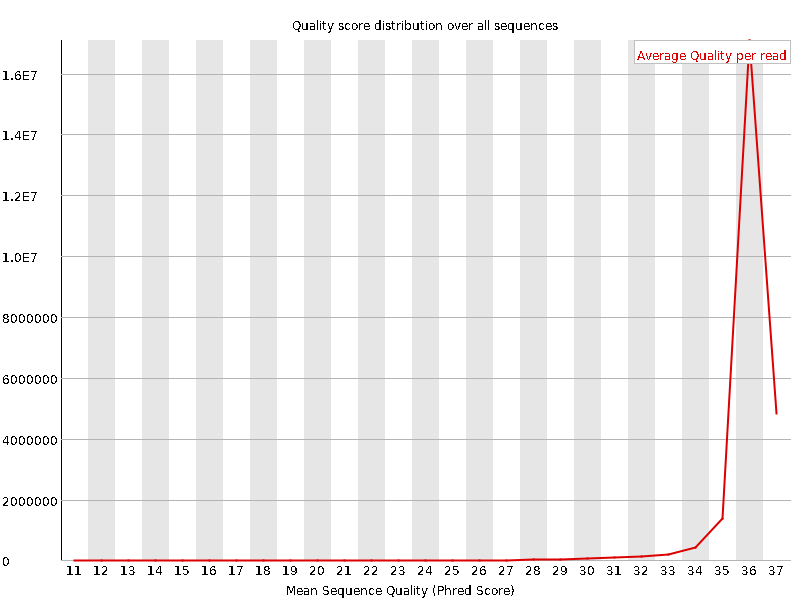

Supplement: Supplemental Information 35 [file peerj-09-10654-s035.zip › EmInf3_Clean_Data2.fq_fastqc/Images/per_sequence_quality.png]

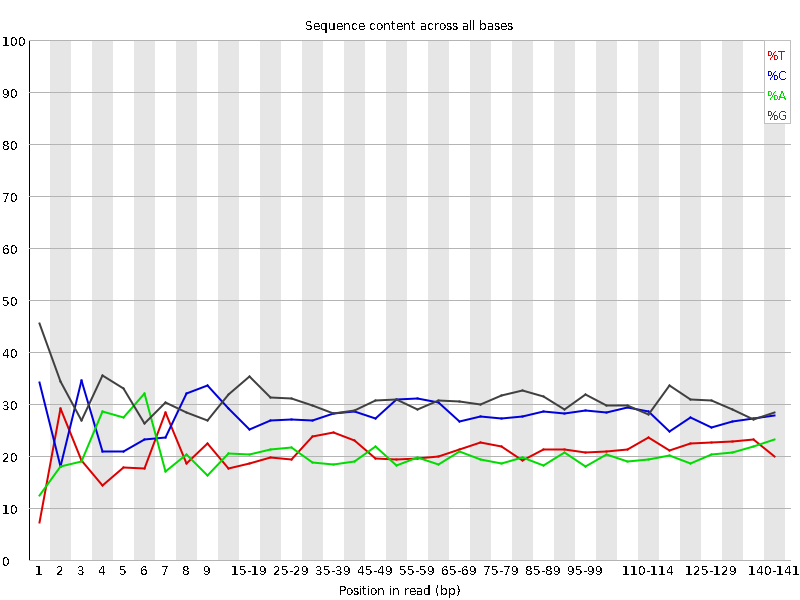

Supplement: Supplemental Information 35 [file peerj-09-10654-s035.zip › EmInf3_Clean_Data2.fq_fastqc/Images/per_base_sequence_content.png]

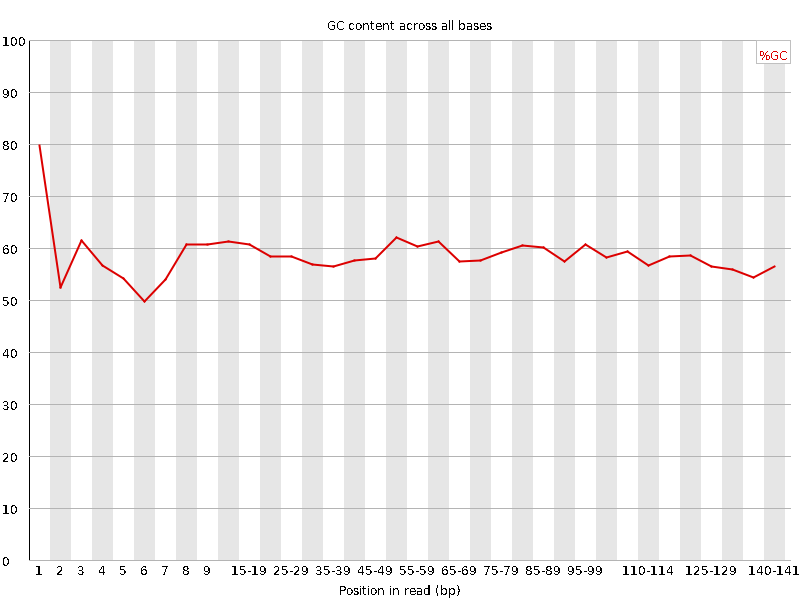

Supplement: Supplemental Information 35 [file peerj-09-10654-s035.zip › EmInf3_Clean_Data2.fq_fastqc/Images/per_base_gc_content.png]

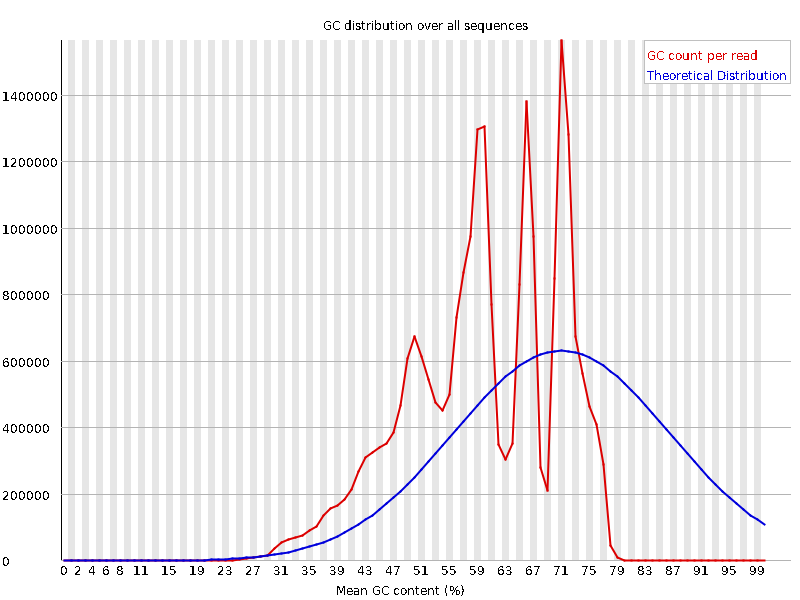

Supplement: Supplemental Information 35 [file peerj-09-10654-s035.zip › EmInf3_Clean_Data2.fq_fastqc/Images/per_sequence_gc_content.png]

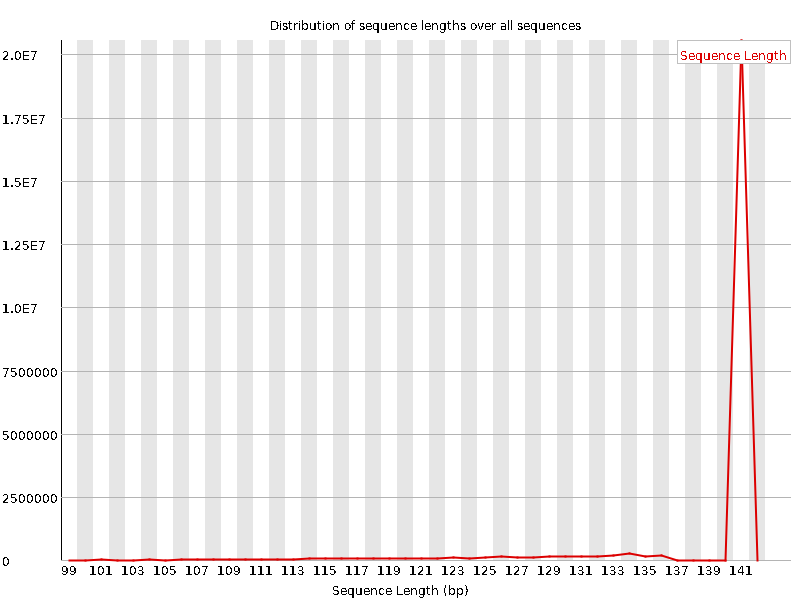

Supplement: Supplemental Information 35 [file peerj-09-10654-s035.zip › EmInf3_Clean_Data2.fq_fastqc/Images/sequence_length_distribution.png]

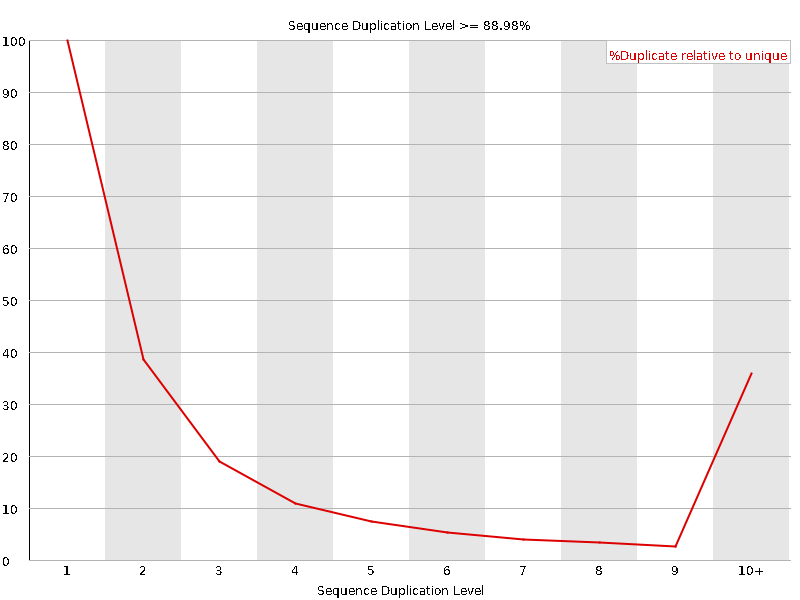

Supplement: Supplemental Information 35 [file peerj-09-10654-s035.zip › EmInf3_Clean_Data2.fq_fastqc/Images/duplication_levels.png]

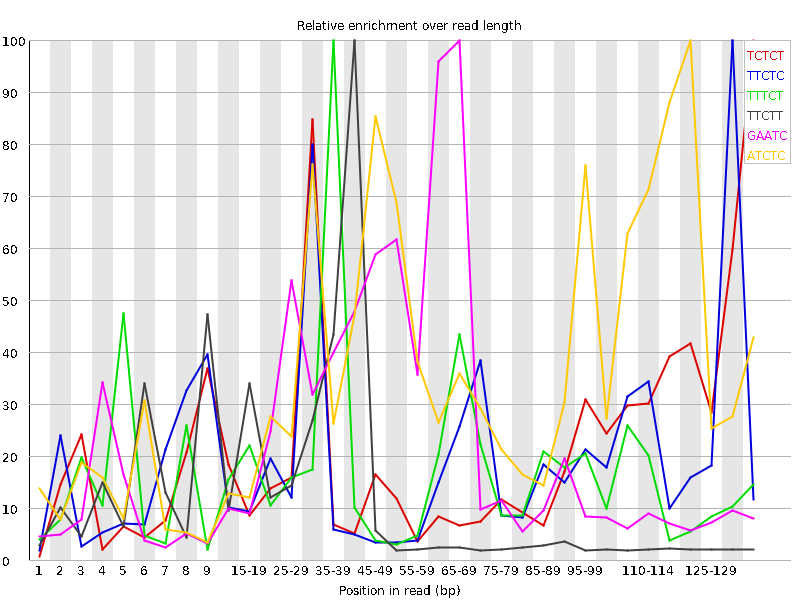

Supplement: Supplemental Information 35 [file peerj-09-10654-s035.zip › EmInf3_Clean_Data2.fq_fastqc/Images/kmer_profiles.png]
