# Supplementary material for: Freshwater sponge hosts and their green algae symbionts: a tractable model to understand intracellular symbiosis
Source: PeerJ. 2021 Feb 11;9:e10654. doi: 10.7717/peerj.10654 (PMC7882143; doi:10.7717/peerj.10654)
Supplement: Supplemental Information 35 [file peerj-09-10654-s035.zip › EmInf3_Clean_Data2.fq_fastqc/fastqc_report.html]

EmInf3\_Clean\_Data2.fq.gz FastQC Report


FastQC Report

Tue 10 Sep 2019  
EmInf3\_Clean\_Data2.fq.gz

## Summary

- Basic Statistics
- Per base sequence quality
- Per sequence quality scores
- Per base sequence content
- Per base GC content
- Per sequence GC content
- Per base N content
- Sequence Length Distribution
- Sequence Duplication Levels
- Overrepresented sequences
- Kmer Content

## Basic Statistics

| Measure | Value |
| --- | --- |
| Filename | EmInf3\_Clean\_Data2.fq.gz |
| File type | Conventional base calls |
| Encoding | Sanger / Illumina 1.9 |
| Total Sequences | 24542165 |
| Filtered Sequences | 0 |
| Sequence length | 100-141 |
| %GC | 58 |

## Per base sequence quality

## Per sequence quality scores

## Per base sequence content

## Per base GC content

## Per sequence GC content

## Per base N content

## Sequence Length Distribution

## Sequence Duplication Levels

## Overrepresented sequences

| Sequence | Count | Percentage | Possible Source |
| --- | --- | --- | --- |
| CTCGGAGACGCCGGAGGGGACCCTGGGAAGAGTTCTCTTTTCTTCTTAAC | 874746 | 3.564257676533427 | No Hit |
| CGGAGACGCCGGAGGGGACCCTGGGAAGAGTTCTCTTTTCTTCTTAACGG | 514993 | 2.098400854203368 | No Hit |
| GGCAACTCCCGGTATGTCGCGAAGCGCGAATCTCCGTGGCCCGTAGGCGG | 396773 | 1.6166992602323391 | No Hit |
| GTTTCGACGTGCCGGCACGCCGGCGAGGACTTCGGCCCTCGCAGGCGTAG | 347031 | 1.4140195047991895 | No Hit |
| GTGCACACCACGAAGGGAGGCAACTCCCGGTATGTCGCGAAGCGCGAATC | 289793 | 1.1807963967319102 | No Hit |
| CAGGTTTCGACGTGCCGGCACGCCGGCGAGGACTTCGGCCCTCGCAGGCG | 277460 | 1.1305441064388573 | No Hit |
| GCAGGTGCACACCACGAAGGGAGGCAACTCCCGGTATGTCGCGAAGCGCG | 267240 | 1.0889014885198596 | No Hit |
| GGTGCACACCACGAAGGGAGGCAACTCCCGGTATGTCGCGAAGCGCGAAT | 263136 | 1.0721792474298824 | No Hit |
| AGCATATGTAGCCAGGCGTCGCCCCGCGTGAGGTTCAGGTTTCGACGTGC | 243392 | 0.9917299472153333 | No Hit |
| CACGAAGGGAGGCAACTCCCGGTATGTCGCGAAGCGCGAATCTCCGTGGC | 242108 | 0.9864981349444925 | No Hit |
| GCCAGGCGTCGCCCCGCGTGAGGTTCAGGTTTCGACGTGCCGGCACGCCG | 188556 | 0.768294076745063 | No Hit |
| CGACGTGCCGGCACGCCGGCGAGGACTTCGGCCCTCGCAGGCGTAGCCGA | 187701 | 0.7648102765179844 | No Hit |
| GGGACGTATAGCCGCGTCGTTCGGAGCGCGCCCGCGACCGAGGAGAGGGT | 180744 | 0.7364631441439661 | No Hit |
| GGGAAGAGTTCTCTTTTCTTCTTAACGGGCCATCACCCTGGAATCAGGTT | 157943 | 0.6435577301350554 | No Hit |
| GGGAAGCATATGTAGCCAGGCGTCGCCCCGCGTGAGGTTCAGGTTTCGAC | 153055 | 0.6236409868485523 | No Hit |
| GGAGACGCCGGAGGGGACCCTGGGAAGAGTTCTCTTTTCTTCTTAACGGG | 152799 | 0.6225978840905031 | No Hit |
| CTCTTTTCTTCTTAACGGGCCATCACCCTGGAATCAGGTTGGCTGGAGGT | 152559 | 0.6216199752548318 | No Hit |
| GGCGTCGCCCCGCGTGAGGTTCAGGTTTCGACGTGCCGGCACGCCGGCGA | 147383 | 0.6005297413655234 | No Hit |
| GGAAGCTCCCTGTAGCACGGTGCAACTCGCCATCTTGGCGACCGGCACCC | 143019 | 0.5827480990369024 | No Hit |
| TGCAGGTGCACACCACGAAGGGAGGCAACTCCCGGTATGTCGCGAAGCGC | 137487 | 0.5602073003746817 | No Hit |
| GCCGGAGGGGACCCTGGGAAGAGTTCTCTTTTCTTCTTAACGGGCCATCA | 135089 | 0.5504363612582671 | No Hit |
| GGAAGAGTTCTCTTTTCTTCTTAACGGGCCATCACCCTGGAATCAGGTTG | 130462 | 0.5315830938305565 | No Hit |
| CGTGCCGGCACGCCGGCGAGGACTTCGGCCCTCGCAGGCGTAGCCGACCG | 119319 | 0.48617960151437334 | No Hit |
| CCCCGCGTGAGGTTCAGGTTTCGACGTGCCGGCACGCCGGCGAGGACTTC | 114850 | 0.4679701240701462 | No Hit |
| GGCTGCAGGTGCACACCACGAAGGGAGGCAACTCCCGGTATGTCGCGAAG | 113409 | 0.4620985964359705 | No Hit |
| CACATTTCCCCGCGGGCTGCAGGTGCACACCACGAAGGGAGGCAACTCCC | 111932 | 0.4560803824764441 | No Hit |
| GACGTATAGCCGCGTCGTTCGGAGCGCGCCCGCGACCGAGGAGAGGGTCT | 110190 | 0.44898239417753083 | No Hit |
| CCCGCGTGAGGTTCAGGTTTCGACGTGCCGGCACGCCGGCGAGGACTTCG | 98062 | 0.3995654010149471 | No Hit |
| GTTCAGGTTTCGACGTGCCGGCACGCCGGCGAGGACTTCGGCCCTCGCAG | 97837 | 0.39864861148150543 | No Hit |
| GGACGTATAGCCGCGTCGTTCGGAGCGCGCCCGCGACCGAGGAGAGGGTC | 97356 | 0.39668871919001436 | No Hit |
| GTCGGAAGCGAGGGTCGACGAAGCGGGCTGGCGGGGGGGCCCTCTCGGGG | 96642 | 0.3937794404038926 | No Hit |
| GACGTGCCGGCACGCCGGCGAGGACTTCGGCCCTCGCAGGCGTAGCCGAC | 96449 | 0.3929930387152071 | No Hit |
| GTGCCGGCACGCCGGCGAGGACTTCGGCCCTCGCAGGCGTAGCCGACCGC | 96261 | 0.3922270101272647 | No Hit |
| CCGGAGGGGACCCTGGGAAGAGTTCTCTTTTCTTCTTAACGGGCCATCAC | 95195 | 0.38788346504882515 | No Hit |
| CCGGTATGTCGCGAAGCGCGAATCTCCGTGGCCCGTAGGCGGCCTTCGGT | 89681 | 0.3654160095492798 | No Hit |
| GTTCTCTTTTCTTCTTAACGGGCCATCACCCTGGAATCAGGTTGGCTGGA | 86564 | 0.3527154185460003 | No Hit |
| TTTCGACGTGCCGGCACGCCGGCGAGGACTTCGGCCCTCGCAGGCGTAGC | 83952 | 0.34207251071777894 | No Hit |
| TCGGAGACGCCGGAGGGGACCCTGGGAAGAGTTCTCTTTTCTTCTTAACG | 78934 | 0.32162606681195405 | No Hit |
| GAGACGCCGGAGGGGACCCTGGGAAGAGTTCTCTTTTCTTCTTAACGGGC | 78905 | 0.32150790282764374 | No Hit |
| GTCCCGACTTTGCGGAAGGGATGTATTTATTAGATCCAAAGCCAATGCGG | 76880 | 0.3132567970266682 | No Hit |
| CTGGGAAGAGTTCTCTTTTCTTCTTAACGGGCCATCACCCTGGAATCAGG | 72968 | 0.29731688300522796 | No Hit |
| CGGAGGGGACCCTGGGAAGAGTTCTCTTTTCTTCTTAACGGGCCATCACC | 72812 | 0.2966812422620417 | No Hit |
| GCTGCAGGTGCACACCACGAAGGGAGGCAACTCCCGGTATGTCGCGAAGC | 72391 | 0.2949658271794685 | No Hit |
| GAAGAGTTCTCTTTTCTTCTTAACGGGCCATCACCCTGGAATCAGGTTGG | 72266 | 0.2944564996608897 | No Hit |
| CATTTCCCCGCGGGCTGCAGGTGCACACCACGAAGGGAGGCAACTCCCGG | 72241 | 0.294354634157174 | No Hit |
| CGCAACGACACATTTCCCCGCGGGCTGCAGGTGCACACCACGAAGGGAGG | 71953 | 0.2931811435543686 | No Hit |
| GGGGAAGCTCCCTGTAGCACGGTGCAACTCGCCATCTTGGCGACCGGCAC | 70905 | 0.2889109416386044 | No Hit |
| GAAGCGGGCTGGCGGGGGGGCCCTCTCGGGGGTCCTGCCGCCGGAGCGTG | 70239 | 0.2861972446196169 | No Hit |
| TTCGACGTGCCGGCACGCCGGCGAGGACTTCGGCCCTCGCAGGCGTAGCC | 70172 | 0.2859242450696587 | No Hit |
| GGCGAAGTTAGGGACGTATAGCCGCGTCGTTCGGAGCGCGCCCGCGACCG | 68922 | 0.2808309698838713 | No Hit |
| CCCGACTTTGCGGAAGGGATGTATTTATTAGATCCAAAGCCAATGCGGGG | 66924 | 0.2726898788269087 | No Hit |
| AGGCAACTCCCGGTATGTCGCGAAGCGCGAATCTCCGTGGCCCGTAGGCG | 65715 | 0.26776366306721516 | No Hit |
| CGTATAGCCGCGTCGTTCGGAGCGCGCCCGCGACCGAGGAGAGGGTCTCT | 61199 | 0.24936267847600244 | No Hit |
| AGCGAGGGTCGACGAAGCGGGCTGGCGGGGGGGCCCTCTCGGGGGTCCTG | 57911 | 0.23596532742730722 | No Hit |
| CTGGAATCAGGTTGGCTGGAGGTAGGGTTGCATGCCCGGTAAAGCGCCAC | 57823 | 0.2356067608542278 | No Hit |
| GGCGAATTGTAGCCGAGAGAGGCACCTGCGCTCGGCAGGCGGTCGACCAA | 56951 | 0.23205369208462254 | No Hit |
| ATTTCCCCGCGGGCTGCAGGTGCACACCACGAAGGGAGGCAACTCCCGGT | 56577 | 0.2305297841490349 | No Hit |
| CTTTTCTTCTTAACGGGCCATCACCCTGGAATCAGGTTGGCTGGAGGTAG | 56073 | 0.22847617559412547 | No Hit |
| CGAAGGGAGGCAACTCCCGGTATGTCGCGAAGCGCGAATCTCCGTGGCCC | 55739 | 0.22711525246448308 | No Hit |
| GTCGACGAAGCGGGCTGGCGGGGGGGCCCTCTCGGGGGTCCTGCCGCCGG | 55500 | 0.22614141824896053 | No Hit |
| GTCGATTCAGACATTTGGCATTTGCGCTTGGCTGAAAAGCCAATGGCGCG | 54948 | 0.22389222792691682 | No Hit |
| CCTGGGAAGAGTTCTCTTTTCTTCTTAACGGGCCATCACCCTGGAATCAG | 53777 | 0.2191208477328712 | No Hit |
| GCAACTCCCGGTATGTCGCGAAGCGCGAATCTCCGTGGCCCGTAGGCGGC | 51704 | 0.21067416016476134 | No Hit |
| ACGAAGGGAGGCAACTCCCGGTATGTCGCGAAGCGCGAATCTCCGTGGCC | 51451 | 0.20964328126715798 | No Hit |
| CCCTGACCCGCCTCTCGGGGCGAAGTTAGGGACGTATAGCCGCGTCGTTC | 50656 | 0.20640395824899718 | No Hit |
| AAGCATATGTAGCCAGGCGTCGCCCCGCGTGAGGTTCAGGTTTCGACGTG | 50557 | 0.20600057085428283 | No Hit |
| CACCACGAAGGGAGGCAACTCCCGGTATGTCGCGAAGCGCGAATCTCCGT | 50325 | 0.20505525897980068 | No Hit |
| AGACGCCGGAGGGGACCCTGGGAAGAGTTCTCTTTTCTTCTTAACGGGCC | 49266 | 0.2007402362424016 | No Hit |
| GGCGGTGCTGTTACGGCGACCGGGTGGTGCCCTGACCCGCCTCTCGGGGC | 49229 | 0.2005894752969023 | No Hit |
| CAGGTGCACACCACGAAGGGAGGCAACTCCCGGTATGTCGCGAAGCGCGA | 48675 | 0.19833213573456132 | No Hit |
| GTCTCTTCGACCCGCCAGCGCAGGCCTTCGTGGCCGGAGCTCCCGCGTTC | 47679 | 0.1942738140665259 | No Hit |
| GACGCCGGAGGGGACCCTGGGAAGAGTTCTCTTTTCTTCTTAACGGGCCA | 47513 | 0.19359742712185335 | No Hit |
| CGCCCCGCGTGAGGTTCAGGTTTCGACGTGCCGGCACGCCGGCGAGGACT | 47229 | 0.19244023499964247 | No Hit |
| AAGCGAGGGTCGACGAAGCGGGCTGGCGGGGGGGCCCTCTCGGGGGTCCT | 47146 | 0.19210204152730617 | No Hit |
| CCCGGTATGTCGCGAAGCGCGAATCTCCGTGGCCCGTAGGCGGCCTTCGG | 47036 | 0.19165383331095687 | No Hit |
| GCATATGTAGCCAGGCGTCGCCCCGCGTGAGGTTCAGGTTTCGACGTGCC | 46279 | 0.18856934585844404 | No Hit |
| CGTCGCCCCGCGTGAGGTTCAGGTTTCGACGTGCCGGCACGCCGGCGAGG | 44531 | 0.18144690983863893 | No Hit |
| GGGAGGCAACTCCCGGTATGTCGCGAAGCGCGAATCTCCGTGGCCCGTAG | 43955 | 0.1790999286330281 | No Hit |
| GCCCTGACCCGCCTCTCGGGGCGAAGTTAGGGACGTATAGCCGCGTCGTT | 43768 | 0.17833797466523432 | No Hit |
| CTTCGACCCGCCAGCGCAGGCCTTCGTGGCCGGAGCTCCCGCGTTCCGGT | 43293 | 0.1764025300946351 | No Hit |
| AGCCAGGCGTCGCCCCGCGTGAGGTTCAGGTTTCGACGTGCCGGCACGCC | 41756 | 0.1701398389261909 | No Hit |
| GGCGTGTGCCTGTAACCGTAGTGAATCAACGGGGCTTGATCTGGCGAATA | 41637 | 0.16965495912850395 | No Hit |
| GAAGCATATGTAGCCAGGCGTCGCCCCGCGTGAGGTTCAGGTTTCGACGT | 41583 | 0.16943492964047793 | No Hit |
| CTCTTCGACCCGCCAGCGCAGGCCTTCGTGGCCGGAGCTCCCGCGTTCCG | 41511 | 0.1691415569897766 | No Hit |
| GCCCCGCGTGAGGTTCAGGTTTCGACGTGCCGGCACGCCGGCGAGGACTT | 39753 | 0.16197837476848517 | No Hit |
| CAACTCCCGGTATGTCGCGAAGCGCGAATCTCCGTGGCCCGTAGGCGGCC | 39547 | 0.1611390030178674 | No Hit |
| TCGACGTGCCGGCACGCCGGCGAGGACTTCGGCCCTCGCAGGCGTAGCCG | 39378 | 0.16045039221274895 | No Hit |
| GGGGACCCTGGGAAGAGTTCTCTTTTCTTCTTAACGGGCCATCACCCTGG | 38193 | 0.15562196733662248 | No Hit |
| GTCGCGAAGCGCGAATCTCCGTGGCCCGTAGGCGGCCTTCGGTGACCGCG | 37208 | 0.15160846649022203 | No Hit |
| AGCGGGCTGGCGGGGGGGCCCTCTCGGGGGTCCTGCCGCCGGAGCGTGGA | 35542 | 0.1448201493226046 | No Hit |
| TGGGAAGAGTTCTCTTTTCTTCTTAACGGGCCATCACCCTGGAATCAGGT | 35530 | 0.14477125388082104 | No Hit |
| GGACCCTGGGAAGAGTTCTCTTTTCTTCTTAACGGGCCATCACCCTGGAA | 35359 | 0.14407449383540533 | No Hit |
| GCGAGGGTCGACGAAGCGGGCTGGCGGGGGGGCCCTCTCGGGGGTCCTGC | 34867 | 0.14206978072227938 | No Hit |
| CTCCGGCGCACAGCCGGCGAATTGTAGCCGAGAGAGGCACCTGCGCTCGG | 34326 | 0.13986541122187063 | No Hit |
| CTCGGGGCGAAGTTAGGGACGTATAGCCGCGTCGTTCGGAGCGCGCCCGC | 34175 | 0.13925014357942747 | No Hit |
| GGAGGGGACCCTGGGAAGAGTTCTCTTTTCTTCTTAACGGGCCATCACCC | 33934 | 0.13826816012360768 | No Hit |
| GAAGCTCCCTGTAGCACGGTGCAACTCGCCATCTTGGCGACCGGCACCCA | 33719 | 0.13739211679165225 | No Hit |
| GAGGCAACTCCCGGTATGTCGCGAAGCGCGAATCTCCGTGGCCCGTAGGC | 32844 | 0.13382682416160108 | No Hit |
| GGGACCCTGGGAAGAGTTCTCTTTTCTTCTTAACGGGCCATCACCCTGGA | 31292 | 0.12750301369092745 | No Hit |
| CAGGCGTCGCCCCGCGTGAGGTTCAGGTTTCGACGTGCCGGCACGCCGGC | 31249 | 0.12732780502453636 | No Hit |
| CGGCGACCGGGTGGTGCCCTGACCCGCCTCTCGGGGCGAAGTTAGGGACG | 30875 | 0.12580389708894874 | No Hit |
| TTCCCCGCGGGCTGCAGGTGCACACCACGAAGGGAGGCAACTCCCGGTAT | 30855 | 0.12572240468597617 | No Hit |
| CTCCCGGTATGTCGCGAAGCGCGAATCTCCGTGGCCCGTAGGCGGCCTTC | 30520 | 0.12435740693618512 | No Hit |
| CCCCGCGGGCTGCAGGTGCACACCACGAAGGGAGGCAACTCCCGGTATGT | 30309 | 0.12349766208482423 | No Hit |
| AAGCGGGCTGGCGGGGGGGCCCTCTCGGGGGTCCTGCCGCCGGAGCGTGG | 30307 | 0.12348951284452696 | No Hit |
| GTAGCCAGGCGTCGCCCCGCGTGAGGTTCAGGTTTCGACGTGCCGGCACG | 30240 | 0.12321651329456876 | No Hit |
| GCGTGAGGTTCAGGTTTCGACGTGCCGGCACGCCGGCGAGGACTTCGGCC | 29892 | 0.12179854548284555 | No Hit |
| CCTGACCCGCCTCTCGGGGCGAAGTTAGGGACGTATAGCCGCGTCGTTCG | 29745 | 0.12119957632099694 | No Hit |
| GGCCAACGTGGGTTGCGGGCGGTGCTGTTACGGCGACCGGGTGGTGCCCT | 29168 | 0.11884852049523749 | No Hit |
| CGAAGCGGGCTGGCGGGGGGGCCCTCTCGGGGGTCCTGCCGCCGGAGCGT | 28744 | 0.1171208815522184 | No Hit |
| GCCGAGAGAGGCACCTGCGCTCGGCAGGCGGTCGACCAAAGTTGACCTGG | 28537 | 0.116277435181452 | No Hit |
| GACACATTTCCCCGCGGGCTGCAGGTGCACACCACGAAGGGAGGCAACTC | 28504 | 0.11614297271654722 | No Hit |
| CATGCAACAAGTCCCGACTTTGCGGAAGGGATGTATTTATTAGATCCAAA | 28197 | 0.11489206433091784 | No Hit |
| GCCGCGTCGTTCGGAGCGCGCCCGCGACCGAGGAGAGGGTCTCTTCGACC | 28148 | 0.11469240794363496 | No Hit |
| CTTCGGCCCTCGCAGGCGTAGCCGACCGCCGCTTCCGCATTTCTCACCGG | 27570 | 0.11233727749772686 | No Hit |
| TGCACACCACGAAGGGAGGCAACTCCCGGTATGTCGCGAAGCGCGAATCT | 27516 | 0.11211724800970085 | No Hit |
| CCTGGAATCAGGTTGGCTGGAGGTAGGGTTGCATGCCCGGTAAAGCGCCA | 26884 | 0.10954208807576675 | No Hit |
| CGAACTCGGAGACGCCGGAGGGGACCCTGGGAAGAGTTCTCTTTTCTTCT | 26801 | 0.10920389460343045 | No Hit |
| CTTTGCGGAAGGGATGTATTTATTAGATCCAAAGCCAATGCGGGGGGCAA | 26567 | 0.10825043348865107 | No Hit |
| ATTCGACTCAGTACGAGAGGAACCGTCGATTCAGACATTTGGCATTTGCG | 26291 | 0.1071258383276292 | No Hit |
| GTAATTCTAGAGCTAATACATGCAACAAGTCCCGACTTTGCGGAAGGGAT | 26222 | 0.10684468953737374 | No Hit |
| GGTTTCGACGTGCCGGCACGCCGGCGAGGACTTCGGCCCTCGCAGGCGTA | 25895 | 0.10551228874877176 | No Hit |
| GTGAGGTTCAGGTTTCGACGTGCCGGCACGCCGGCGAGGACTTCGGCCCT | 25851 | 0.10533300546223204 | No Hit |
| TGTAGCCAGGCGTCGCCCCGCGTGAGGTTCAGGTTTCGACGTGCCGGCAC | 25330 | 0.10321012836479586 | No Hit |
| TGGGGAAGCTCCCTGTAGCACGGTGCAACTCGCCATCTTGGCGACCGGCA | 25330 | 0.10321012836479586 | No Hit |
| GCAACAAGTCCCGACTTTGCGGAAGGGATGTATTTATTAGATCCAAAGCC | 25189 | 0.10263560692383904 | No Hit |
| GGGCTGCAGGTGCACACCACGAAGGGAGGCAACTCCCGGTATGTCGCGAA | 25148 | 0.10246854749774521 | No Hit |
| CGACTCAGTACGAGAGGAACCGTCGATTCAGACATTTGGCATTTGCGCTT | 25087 | 0.10221999566867879 | No Hit |
| CTTAAATCTCCGGCGCACAGCCGGCGAATTGTAGCCGAGAGAGGCACCTG | 24839 | 0.10120948987181856 | No Hit |
| CTGCAGGTGCACACCACGAAGGGAGGCAACTCCCGGTATGTCGCGAAGCG | 24787 | 0.10099760962408982 | No Hit |
| GCACACCACGAAGGGAGGCAACTCCCGGTATGTCGCGAAGCGCGAATCTC | 24687 | 0.1005901476092268 | No Hit |
| GCGAACTCGGAGACGCCGGAGGGGACCCTGGGAAGAGTTCTCTTTTCTTC | 24666 | 0.10050458058610558 | No Hit |
| GTCGCCCCGCGTGAGGTTCAGGTTTCGACGTGCCGGCACGCCGGCGAGGA | 24573 | 0.10012564091228301 | No Hit |

## Kmer Content

| Sequence | Count | Obs/Exp Overall | Obs/Exp Max | Max Obs/Exp Position |
| --- | --- | --- | --- | --- |
| TCTCT | 12784910 | 5.205406 | 23.503986 | 135-137 |
| TTCTC | 11871615 | 4.8335567 | 23.689371 | 130-134 |
| TTTCT | 8716950 | 4.681366 | 26.088575 | 35-39 |
| TTCTT | 8487090 | 4.5579214 | 38.733574 | 40-44 |
| GAATC | 9711140 | 4.0190063 | 15.248438 | 65-69 |
| ATCTC | 8864295 | 3.81713 | 9.365715 | 120-124 |
| CTCTT | 9187505 | 3.7407143 | 11.679616 | 1 |
| CATTT | 6408795 | 3.640158 | 10.548589 | 3 |
| ATCAA | 5672935 | 3.6043189 | 12.122954 | 85-89 |
| CTTCT | 8813820 | 3.5885677 | 19.477503 | 110-114 |
| TCAAA | 5170120 | 3.2848537 | 11.855409 | 85-89 |
| AGGTT | 9043665 | 3.2157118 | 14.294719 | 2 |
| CTTCG | 11402975 | 3.1984828 | 12.808516 | 70-74 |
| ATTTC | 5529395 | 3.1406639 | 9.955209 | 4 |
| GTTCT | 8485710 | 3.1395333 | 19.888575 | 130-134 |
| AATCA | 4890515 | 3.1072052 | 17.812014 | 65-69 |
| GCCAA | 9535215 | 2.9917676 | 9.802158 | 80-84 |
| TTGAA | 5458000 | 2.9794295 | 15.043991 | 135-137 |
| CATCA | 6446370 | 2.935917 | 12.846144 | 55-59 |
| AAGAT | 5066430 | 2.925077 | 10.85416 | 110-114 |
| GCGAA | 10108710 | 2.8821268 | 8.804522 | 45-49 |
| TCTTC | 7037330 | 2.8652654 | 12.314858 | 40-44 |
| TATGT | 5546935 | 2.8629704 | 25.303421 | 5 |
| GGTTG | 12318835 | 2.853221 | 11.883721 | 85-89 |
| CTGGA | 10541460 | 2.841727 | 13.42012 | 60-64 |
| AATCT | 4707780 | 2.8281062 | 10.816852 | 45-49 |
| CCAAT | 6123200 | 2.7887332 | 10.039904 | 100-104 |
| TCACC | 8499740 | 2.7749 | 14.006974 | 55-59 |
| AATCC | 6044370 | 2.752831 | 9.055006 | 100-104 |
| TATCA | 4580435 | 2.7516065 | 11.33439 | 85-89 |
| GTAAA | 4706800 | 2.717447 | 15.272685 | 100-104 |
| CCATC | 8252630 | 2.6942263 | 19.062992 | 50-54 |
| CTTAA | 4483840 | 2.693579 | 15.867796 | 45-49 |
| CGAAG | 9431825 | 2.689138 | 15.019815 | 3 |
| TGGCC | 13879425 | 2.6820462 | 9.689166 | 75-79 |
| TTTTC | 4952885 | 2.6599061 | 24.724127 | 35-39 |
| CCGGT | 13692680 | 2.6459596 | 10.844805 | 95-99 |
| TCTTT | 4907280 | 2.635414 | 15.229547 | 35-39 |
| TAAAG | 4529220 | 2.614922 | 16.063328 | 100-104 |
| GACTT | 6668495 | 2.6093993 | 6.613227 | 25-29 |
| GACCG | 12753300 | 2.6064723 | 5.9037514 | 55-59 |
| AAGCG | 9074420 | 2.587237 | 13.289133 | 100-104 |
| CAACT | 5674005 | 2.5841532 | 26.409369 | 3 |
| AAAGC | 5863755 | 2.566611 | 18.63664 | 100-104 |
| TTCGG | 10032430 | 2.557125 | 7.0467615 | 70-74 |
| TTAAC | 4249645 | 2.5528908 | 16.698763 | 45-49 |
| GGCAA | 8911300 | 2.5407295 | 16.973957 | 1 |
| CTTTT | 4729300 | 2.5398314 | 22.826447 | 35-39 |
| AACTC | 5574815 | 2.5389783 | 27.804354 | 4 |
| TCTTA | 4466165 | 2.536755 | 27.227013 | 40-44 |
| ACCCT | 7714625 | 2.5185845 | 16.036947 | 55-59 |
| CAGGT | 9319805 | 2.5123978 | 13.240365 | 65-69 |
| CGACC | 10957565 | 2.464476 | 5.4398246 | 50-54 |
| GGCCA | 12036775 | 2.4600315 | 8.711591 | 50-54 |
| ACGCT | 8209690 | 2.4355016 | 10.188192 | 110-114 |
| GCTGG | 13822740 | 2.4272184 | 9.689495 | 75-79 |
| GCTTC | 8646785 | 2.4253843 | 12.097843 | 110-114 |
| AAAAT | 2733605 | 2.4229136 | 13.406363 | 125-129 |
| CGGTA | 8976385 | 2.4198198 | 12.68216 | 95-99 |
| TGGAA | 6335315 | 2.3825207 | 18.16588 | 60-64 |
| CGCTT | 8283840 | 2.3235795 | 7.949102 | 110-114 |
| CAAGA | 5241950 | 2.2944422 | 8.383194 | 110-114 |
| CAAAC | 4743575 | 2.2849152 | 10.293628 | 90-94 |
| ATCAC | 4999795 | 2.277093 | 12.932197 | 55-59 |
| GGAAT | 6043500 | 2.2727776 | 21.84838 | 60-64 |
| AGCGC | 11096665 | 2.2678952 | 10.294677 | 100-104 |
| CGTAT | 5767830 | 2.2569666 | 10.150727 | 5 |
| GAAGA | 5670900 | 2.2555695 | 17.281883 | 25-29 |
| TTCGA | 5711670 | 2.2349913 | 19.044626 | 3 |
| CTCTA | 5163815 | 2.2236347 | 23.532663 | 135-137 |
| TGAAA | 3843900 | 2.2192562 | 7.4797125 | 120-124 |
| ACACA | 4599925 | 2.215721 | 6.48846 | 8 |
| GGAGG | 13115480 | 2.213373 | 8.774104 | 10-14 |
| CCTTC | 7170345 | 2.213329 | 10.048513 | 70-74 |
| CGGTG | 12596305 | 2.211861 | 10.003343 | 125-129 |
| AGATC | 5284045 | 2.1868298 | 9.063236 | 115-119 |
| GGTGC | 12387930 | 2.1752715 | 13.402216 | 125-129 |
| GGGTT | 9354385 | 2.1666112 | 9.868269 | 85-89 |
| ACTTC | 5029100 | 2.165624 | 9.278704 | 25-29 |
| GCATT | 5532955 | 2.1650596 | 8.498813 | 65-69 |
| GTTGC | 8493720 | 2.1649294 | 13.488967 | 85-89 |
| TTGCA | 5529265 | 2.1636157 | 19.014118 | 85-89 |
| TCTCA | 5022365 | 2.1627235 | 9.411727 | 70-74 |
| TGCAT | 5524230 | 2.1616454 | 10.976134 | 90-94 |
| CGCGA | 10422235 | 2.1300576 | 7.775183 | 40-44 |
| GAGGA | 8181740 | 2.1197422 | 5.9399805 | 25-29 |
| GAAGC | 7429640 | 2.1182888 | 8.839068 | 3 |
| ATCAG | 5105865 | 2.113089 | 17.387373 | 65-69 |
| TGACC | 7106655 | 2.1082733 | 5.9674735 | 75-79 |
| CCCTG | 9897780 | 2.1048086 | 6.856275 | 20-24 |
| GCAAC | 6691760 | 2.0996058 | 21.12799 | 2 |
| TGGAG | 8534050 | 2.0905316 | 12.118353 | 75-79 |
| TCTAC | 4784680 | 2.060372 | 35.019886 | 135-137 |
| CGTTC | 7279075 | 2.0417478 | 13.94725 | 130-134 |
| CGAAT | 4887025 | 2.022521 | 10.946417 | 45-49 |
| CGTAG | 7493790 | 2.0201476 | 8.8643875 | 60-64 |
| CAATC | 4425345 | 2.015467 | 11.521415 | 100-104 |
| TGGTG | 8687395 | 2.0121267 | 12.77717 | 115-119 |
| GAAAA | 3290680 | 2.0093553 | 6.9185038 | 125-129 |
| ACGTG | 7441080 | 2.0059383 | 13.113354 | 7 |
| ATGTC | 5108855 | 1.9991081 | 7.219719 | 30-34 |
| TAGCC | 6699955 | 1.9876208 | 18.09522 | 9 |
| CACAC | 5735815 | 1.9804897 | 14.375845 | 4 |
| GGCTG | 11260700 | 1.9773343 | 7.068567 | 75-79 |
| TTCTG | 5343270 | 1.9768969 | 18.352192 | 110-114 |
| GCCAT | 6660110 | 1.9758 | 14.15611 | 50-54 |
| AGGCG | 10624150 | 1.9730799 | 5.2878246 | 45-49 |
| GCGTA | 7280565 | 1.9626671 | 7.3347154 | 80-84 |
| ACGAA | 4475030 | 1.9587554 | 17.548079 | 2 |
| AAGAG | 4916695 | 1.9555883 | 18.625216 | 25-29 |
| ACTCC | 5962425 | 1.9465458 | 18.719074 | 5 |
| TCGAC | 6491430 | 1.9257594 | 14.660019 | 4 |
| TTGTA | 3730720 | 1.9255573 | 8.515212 | 120-124 |
| GCCCT | 9043050 | 1.9230461 | 5.510994 | 35-39 |
| GTAGG | 7846190 | 1.922031 | 12.767518 | 80-84 |
| AATAT | 2279895 | 1.9106506 | 6.1362457 | 70-74 |
| GCCGG | 14320795 | 1.9064733 | 10.553523 | 8 |
| TAACG | 4595890 | 1.9020331 | 17.209272 | 45-49 |
| CTTGA | 4838580 | 1.8933488 | 12.501195 | 135-137 |
| CGGAG | 10046770 | 1.8658507 | 26.691692 | 3 |
| AGGGA | 7199195 | 1.8651823 | 9.268714 | 6 |
| TCAGG | 6907105 | 1.8619913 | 12.61352 | 65-69 |
| CTGGC | 9625200 | 1.8599639 | 6.4778657 | 95-99 |
| CAACG | 5910015 | 1.8543257 | 7.5025816 | 125-129 |
| CAGGC | 9066615 | 1.8530012 | 5.8498044 | 3 |
| CACGC | 8138335 | 1.8304007 | 11.066069 | 105-109 |
| GTATC | 4676600 | 1.8299656 | 9.953132 | 85-89 |
| TCCGC | 8552095 | 1.8186423 | 7.2903485 | 100-104 |
| AGGTA | 4803805 | 1.8065659 | 10.431366 | 80-84 |
| CGCCA | 8031865 | 1.8064542 | 9.608043 | 105-109 |
| TGTTA | 3494715 | 1.8037467 | 9.968209 | 105-109 |
| AACGT | 4347075 | 1.7990599 | 6.7697344 | 85-89 |
| GTGGC | 10244955 | 1.7989734 | 9.209909 | 115-119 |
| TGAAT | 3288850 | 1.7953272 | 7.043077 | 130-134 |
| GGTAA | 4766195 | 1.7924219 | 18.304165 | 95-99 |
| CGCCG | 12194330 | 1.7864941 | 18.451086 | 9 |
| CCAAC | 5152820 | 1.7791904 | 7.6495566 | 85-89 |
| ATATT | 2237130 | 1.7726452 | 5.903828 | 70-74 |
| AAACG | 4037225 | 1.7671247 | 10.865942 | 90-94 |
| ACCGC | 7826260 | 1.7602116 | 5.664164 | 50-54 |
| GCAGG | 9378685 | 1.7417763 | 7.1605062 | 1 |
| AGTTC | 4443035 | 1.738571 | 11.112503 | 30-34 |
| GAGGT | 7083350 | 1.735163 | 6.5080495 | 80-84 |
| AGGGT | 7040110 | 1.7245708 | 6.8134346 | 85-89 |
| CACCA | 4989145 | 1.7226759 | 14.58412 | 6 |
| TGTAG | 4836650 | 1.7197975 | 14.365098 | 7 |
| AGAGT | 4540375 | 1.7074978 | 18.11082 | 25-29 |
| GCCTT | 6075335 | 1.704104 | 7.104993 | 70-74 |
| GACGT | 6308945 | 1.7007415 | 13.037747 | 6 |
| GATCT | 4344245 | 1.6999142 | 10.041821 | 115-119 |
| GACCC | 7530350 | 1.6936582 | 11.257908 | 15-19 |
| ATTCA | 2792305 | 1.6774224 | 5.9896097 | 5 |
| AACGC | 5324120 | 1.6704953 | 6.0443206 | 95-99 |
| CTGTT | 4493420 | 1.6624703 | 5.955734 | 105-109 |
| GTATG | 4646890 | 1.6523234 | 9.040629 | 30-34 |
| CTCCC | 7033225 | 1.6459223 | 17.867306 | 6 |
| CCACG | 7290050 | 1.6396118 | 10.756781 | 105-109 |
| GGAAG | 6290465 | 1.6297466 | 13.340974 | 2 |
| CCCTC | 6959705 | 1.6287172 | 5.965693 | 35-39 |
| TCTCC | 5226095 | 1.6131815 | 6.6933784 | 50-54 |
| CATCT | 3740035 | 1.6105285 | 5.487492 | 110-114 |
| TCTTG | 4333870 | 1.6034403 | 6.934638 | 120-124 |
| GTAGC | 5946115 | 1.6029313 | 10.700282 | 8 |
| AAATC | 2514770 | 1.5977675 | 9.957417 | 125-129 |
| CCTGG | 8201745 | 1.5848967 | 8.486834 | 20-24 |
| AAGTT | 2902920 | 1.5846546 | 6.7030816 | 5 |
| CTCAC | 4826305 | 1.575638 | 5.352338 | 70-74 |
| CCCGC | 9752955 | 1.5723889 | 7.0791388 | 9 |
| GGTAT | 4421140 | 1.5720521 | 7.550845 | 30-34 |
| ACGCC | 6964715 | 1.5664407 | 27.80274 | 8 |
| GGCGT | 8857345 | 1.5553147 | 5.2824683 | 1 |
| TTGGC | 6087000 | 1.5514905 | 11.916101 | 70-74 |
| ATCCG | 5213165 | 1.546547 | 5.5087676 | 100-104 |
| TTCAG | 3928730 | 1.5373222 | 6.6477785 | 2 |
| CTTCC | 4975675 | 1.5358824 | 6.8271284 | 60-64 |
| CGCAT | 5149280 | 1.5275944 | 6.1674385 | 65-69 |
| ACCGG | 7473470 | 1.5274001 | 5.234017 | 115-119 |
| CATGC | 5109595 | 1.5158218 | 12.542646 | 90-94 |
| TGCCC | 7003450 | 1.489316 | 10.33839 | 90-94 |
| GCATG | 5523210 | 1.4889259 | 8.069833 | 90-94 |
| GGAGA | 5739230 | 1.4869314 | 32.38096 | 4 |
| ATGCC | 5011255 | 1.4866481 | 13.772695 | 90-94 |
| TACGG | 5510190 | 1.4854162 | 13.1529 | 135-137 |
| GTTCA | 3788430 | 1.4824225 | 7.2945223 | 1 |
| GAGTT | 4154280 | 1.477163 | 9.467019 | 30-34 |
| CCGCC | 9142720 | 1.4740057 | 5.147601 | 55-59 |
| TCCCG | 6924435 | 1.472513 | 12.370834 | 7 |
| TCTGG | 5768135 | 1.4702162 | 7.4099483 | 110-114 |
| GAGGG | 8681845 | 1.4651513 | 8.914445 | 10-14 |
| GGTGA | 5980500 | 1.4650047 | 6.0370855 | 75-79 |
| GCGTC | 7550995 | 1.4591466 | 8.840129 | 120-124 |
| AATGG | 3876565 | 1.4578589 | 6.28457 | 135-137 |
| TGGCT | 5681760 | 1.4482006 | 12.708846 | 70-74 |
| GTGCC | 7475435 | 1.4445455 | 9.651514 | 9 |
| GCGTT | 5665590 | 1.4440789 | 8.393674 | 130-134 |
| AACGG | 5058330 | 1.4421968 | 13.084887 | 45-49 |
| CCGGA | 7047595 | 1.4403614 | 14.973299 | 9 |
| TAATA | 1718090 | 1.4398338 | 7.3175216 | 70-74 |
| AGGAC | 4964150 | 1.4153448 | 6.3175287 | 25-29 |
| GGGAA | 5452730 | 1.4127045 | 13.144166 | 1 |
| TCGGA | 5219865 | 1.4071515 | 37.221638 | 2 |
| AGACG | 4908915 | 1.3995965 | 35.00064 | 6 |
| TTAAT | 1761115 | 1.3954632 | 7.239987 | 70-74 |
| TCCGG | 7191550 | 1.3896877 | 7.3459783 | 125-129 |
| GGTGG | 8679845 | 1.3849899 | 9.970876 | 115-119 |
| GGTAG | 5631095 | 1.3794134 | 10.63653 | 80-84 |
| AGGCA | 4829025 | 1.3768189 | 5.5887094 | 15-19 |
| CCGTA | 4634500 | 1.3748792 | 6.167439 | 60-64 |
| AAGGG | 5297595 | 1.3725117 | 9.386573 | 5 |
| CGGCA | 6666115 | 1.3623958 | 5.560872 | 9 |
| TGCTG | 5284845 | 1.3470325 | 5.3922977 | 105-109 |
| TGGGA | 5475255 | 1.3412381 | 12.014902 | 20-24 |
| CTTGT | 3606385 | 1.3342862 | 8.024502 | 120-124 |
| TCGCA | 4494805 | 1.3334371 | 6.1460214 | 35-39 |
| CTGGT | 5225500 | 1.3319063 | 7.5721035 | 115-119 |
| GCAAG | 4663020 | 1.3294886 | 5.446543 | 110-114 |
| TAGGG | 5413100 | 1.3260125 | 11.822798 | 80-84 |
| CGTCC | 6156940 | 1.3093017 | 10.733615 | 120-124 |
| GAAGG | 5037255 | 1.3050623 | 9.4774885 | 4 |
| ACCAC | 3772805 | 1.3026922 | 14.494462 | 7 |
| CACCC | 5256210 | 1.3009583 | 11.487682 | 55-59 |
| GTTTC | 3488840 | 1.290797 | 18.845333 | 1 |
| ATTTT | 1711010 | 1.2818801 | 6.8698177 | 135-137 |
| ACACC | 3697215 | 1.2765923 | 14.262558 | 5 |
| GTTAC | 3256210 | 1.2741634 | 6.9295654 | 110-114 |
| TAGAC | 3067535 | 1.2695155 | 7.459839 | 125-129 |
| CGACT | 4262120 | 1.264408 | 5.0726194 | 130-134 |
| TTACG | 3230225 | 1.2639953 | 7.7207303 | 110-114 |
| GCGAC | 6139955 | 1.2548612 | 5.555888 | 115-119 |
| GTGAC | 4643810 | 1.2518607 | 6.8384643 | 75-79 |
| GTAGA | 3308060 | 1.2440615 | 5.8828216 | 120-124 |
| CTCCG | 5847960 | 1.2435956 | 5.389572 | 50-54 |
| GTGCA | 4610940 | 1.2429998 | 12.017016 | 1 |
| ATATG | 2272200 | 1.2403553 | 19.14486 | 4 |
| TATAG | 2269220 | 1.2387285 | 14.185583 | 7 |
| ACATT | 2056760 | 1.2355582 | 9.910668 | 2 |
| GTCGC | 6377930 | 1.2324648 | 7.7047186 | 8 |
| ATGTA | 2248280 | 1.2272978 | 19.674484 | 6 |
| CCCGG | 8377145 | 1.2272689 | 8.170711 | 8 |
| TTGCG | 4814590 | 1.2271712 | 5.416568 | 95-99 |
| GTTGG | 5287805 | 1.2247324 | 9.771079 | 70-74 |
| GCACA | 3868425 | 1.2137564 | 12.845422 | 3 |
| GCCGA | 5930475 | 1.2120484 | 5.058213 | 50-54 |
| TTTCG | 3243180 | 1.199908 | 18.034607 | 2 |
| GGGAC | 6425585 | 1.1933371 | 8.149501 | 15-19 |
| ACGGC | 5756775 | 1.1765484 | 6.3857923 | 135-137 |
| TCCGT | 4168790 | 1.1693267 | 5.1459556 | 50-54 |
| GTATA | 2113715 | 1.153841 | 14.030651 | 6 |
| GACTA | 2745350 | 1.1361775 | 7.851917 | 115-119 |
| ACTTG | 2849075 | 1.1148504 | 7.2195406 | 130-134 |
| GCCAC | 4930645 | 1.1089559 | 9.676065 | 105-109 |
| GAAGT | 2948525 | 1.1088512 | 5.667832 | 4 |
| CTACG | 3712590 | 1.1013837 | 12.48559 | 135-137 |
| TGCGT | 4306825 | 1.097749 | 7.7032676 | 130-134 |
| GAGGC | 5865295 | 1.089282 | 6.7458515 | 9 |
| TGCAC | 3671210 | 1.0891079 | 12.148408 | 2 |
| TGCCG | 5610935 | 1.0842514 | 5.1009526 | 6 |
| CTCGG | 5587435 | 1.0797101 | 25.05695 | 1 |
| GCGCC | 7296405 | 1.0689381 | 5.5590262 | 105-109 |
| GGTTT | 3139145 | 1.0553784 | 13.110375 | 3 |
| GTCCG | 5454220 | 1.053968 | 9.957436 | 120-124 |
| TAAAT | 1236605 | 1.0363284 | 6.112742 | 3 |
| CACGA | 3283425 | 1.030207 | 12.813213 | 9 |
| AGCCA | 3278180 | 1.0285614 | 5.410248 | 10-14 |
| CCTGA | 3457340 | 1.0256606 | 5.681861 | 130-134 |
| CACAT | 2249195 | 1.0243671 | 7.95471 | 1 |
| ATCTT | 1782710 | 1.0125688 | 5.0828657 | 30-34 |
| AAGCT | 2432055 | 1.0065187 | 8.948043 | 3 |
| GTGCG | 5685360 | 0.99832666 | 9.939848 | 125-129 |
| AGTTA | 1815610 | 0.99111056 | 6.049292 | 6 |
| CGTGC | 5111285 | 0.98769957 | 9.524969 | 8 |
| AGCTC | 3285615 | 0.97471654 | 6.513308 | 4 |
| CATAT | 1620415 | 0.97343254 | 21.17041 | 3 |
| GGACC | 4706590 | 0.9619154 | 9.521335 | 15-19 |
| ATAGC | 2322820 | 0.9613113 | 11.289185 | 8 |
| AATTG | 1751895 | 0.95632964 | 6.7349467 | 5 |
| GAGAC | 3351700 | 0.95561403 | 35.176067 | 5 |
| AGGTG | 3849390 | 0.94296044 | 9.1682825 | 3 |
| GCACG | 4508910 | 0.9215143 | 5.4392385 | 15-19 |
| TCCCT | 2966090 | 0.91556716 | 6.6093693 | 7 |
| AAGCA | 2084825 | 0.912544 | 9.792691 | 4 |
| ACGTA | 2199330 | 0.9102043 | 10.49168 | 4 |
| CGACG | 4381465 | 0.8954675 | 10.037325 | 5 |
| GCCAG | 4324530 | 0.8838314 | 6.0609136 | 1 |
| TTAAA | 1051825 | 0.8814749 | 5.6763988 | 2 |
| GGGAG | 5172285 | 0.87287664 | 5.8746076 | 7 |
| TGCAG | 3210630 | 0.8655095 | 5.488211 | 1 |
| TCGCC | 4057680 | 0.86288434 | 5.8429055 | 9 |
| ACTTT | 1518465 | 0.8624792 | 6.296607 | 7 |
| CGTCG | 4310950 | 0.8330436 | 5.4053864 | 7 |
| TTTGC | 2244955 | 0.83058596 | 5.01724 | 7 |
| TACTC | 1902055 | 0.8190602 | 5.4480405 | 120-124 |
| ACGGG | 4366430 | 0.8109181 | 8.880263 | 45-49 |
| TTCCC | 2508970 | 0.7744643 | 5.45037 | 6 |
| ATTGT | 1459365 | 0.75323015 | 5.1987925 | 6 |
| AGCAT | 1794915 | 0.742835 | 14.533791 | 1 |
| TTTCC | 1817175 | 0.73986715 | 7.09216 | 5 |
| CTGGG | 4203040 | 0.73803717 | 8.138173 | 20-24 |
| GAATT | 1328325 | 0.72511 | 5.9764047 | 4 |
| CCAGG | 3200850 | 0.65417784 | 5.5819902 | 2 |
| GTCGA | 2425565 | 0.65387464 | 5.4003043 | 1 |
| GACGC | 3101755 | 0.6339252 | 25.169008 | 7 |
| GTAGT | 1772265 | 0.63017523 | 5.041035 | 125-129 |
| CCTGT | 2223965 | 0.6238121 | 8.439576 | 9 |
| CCCCG | 3363725 | 0.54230577 | 5.9745717 | 8 |
| GCATA | 1306825 | 0.5408364 | 14.361742 | 2 |

Produced by FastQC (version 0.10.1)
